# Exploration of Hydrazide-Based HDAC8 PROTACs for the Treatment of Hematological Malignancies and Solid Tumors

**DOI:** 10.1021/acs.jmedchem.4c00836

**Published:** 2024-08-01

**Authors:** Chunlong Zhao, Jianqiu Zhang, Hangyu Zhou, Rita Setroikromo, Gerrit J. Poelarends, Frank J. Dekker

**Affiliations:** Department of Chemical and Pharmaceutical Biology, Groningen Research Institute of Pharmacy (GRIP), University of Groningen, Antonius Deusinglaan 1, 9713 AV Groningen, The Netherlands

## Abstract

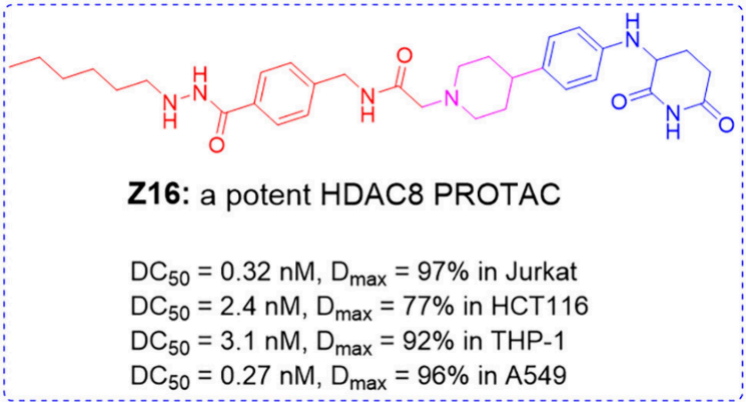

HDAC8 can mediate signals by using its enzymatic or nonenzymatic functions, which are expected to be critical for various types of cancer. Herein, we employed proteolysis targeting chimera (PROTAC) technology to target the enzymatic as well as the nonenzymatic functions of HDAC8. A potent and selective HDAC8 PROTAC **Z16 (CZH-726)** with low nanomolar DC_50_ values in various cell lines was identified. Interestingly, **Z16** induced structural maintenance of chromosomes protein 3 (SMC3) hyperacetylation at low concentrations and histone hyperacetylation at high concentrations, which can be explained by HDAC8 degradation and off-target HDAC inhibition, respectively. Notably, **Z16** potently inhibited proliferation of various cancer cell lines and the antiproliferative mechanisms proved to be cell-type-dependent, which, to a large extent, is due to off-target HDAC inhibition. In conclusion, we report a hydrazide-based HDAC8 PROTAC **Z16**, which can be used as a probe to investigate the biological functions of HDAC8.

## Introduction

In recent years, insight in different types of cell death has developed quickly. Currently, distinct mechanisms such as apoptosis, necroptosis, and ferroptosis have been described.^1,2^ Development of potent small molecule modulators of these mechanisms holds promise for cancer therapy.

Histone deacetylases (HDACs) play important roles in regulation of gene transcription and protein function by exerting both enzymatic and nonenzymatic functions.^3−5^ The family of HDACs comprises 11 zinc-dependent HDACs grouped into four enzyme classes: class I (HDAC1, 2, 3, and 8), class IIa (HDAC4, 5, 7, and 9), class IIb (HDAC6 and 10), and class IV (HDAC11).^3,5^ Although HDAC inhibitors can induce apoptosis in cancer cells, their molecular mechanisms of action are not fully understood.^6−8^ In addition, the HDAC inhibitors that are currently FDA-approved have serious side effects, probably owing to their nonselectivity among HDAC isoenzymes.^9,10^ Therefore, HDAC research is shifting toward targeting specific HDAC isoenzymes and nonenzymatic functions.^11−16^

The HDAC isoenzyme HDAC8 has been connected to processes that are important for oncology. HDAC8 is a unique class I HDAC, which contains 377 amino acids and localizes in both nucleus and cytoplasm.^12−14^ Although it has been shown that HDAC8 deacetylates core histones in vitro, it remains unclear if histones are the bona fide HDAC8 substrates in vivo.^13,14,17^ Interestingly, some nonhistone substrates have also been described for HDAC8, such as Structural Maintenance of Chromosomes protein 3 (SMC3), Estrogen-Related Receptor alpha (ERRα), cortical actin-binding protein (cortactin), and p53.^12,14,17^ Besides functions that require its deacetylase enzymatic activity, nonenzymatic functions have also been described for HDAC8.^18−20^ These include aberrant expression levels or dysregulated interactions with transcription factors, which proved to be critical for HDAC8 functions in various cancers mainly including T-cell leukemia, acute myeloid leukemia (AML), childhood neuroblastoma, colon cancer, breast cancer, and lung cancer.^9,10,14,17,19−21^ Therefore, development of small molecule HDAC8 modulators has potential for the treatment of these cancers.

Although potent HDAC8 inhibitors have been reported, most of them showed modest selectivity over other HDAC isoforms and had limited antiproliferative activities in various cancer models.^21−25^ This indicates the need for novel modalities to increase the HDAC8 inhibitory potency and also raises interest in targeting its nonenzymatic functions.

**Figure 1:**
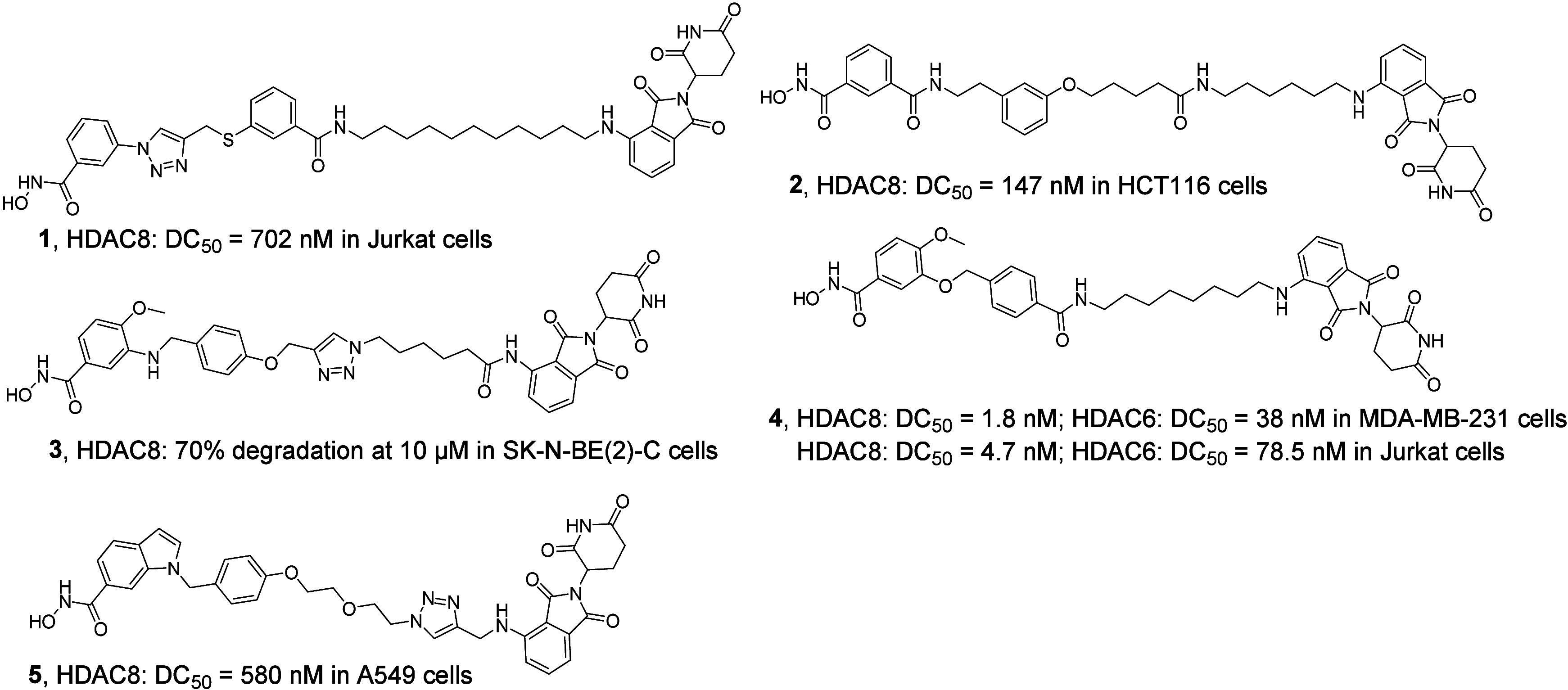
Chemical structures of representative HDAC8 PROTACs.

The development of proteolysis targeting chimeras (PROTACs) has emerged as a powerful strategy in drug discovery.^26−29^ PROTACs consist of a protein of interest (POI) binder, a E3 ligase recruiter, and a linker and trigger the degradation of the POI by hijacking the ubiquitin-proteasome system (UPS), which enables targeting previously undruggable proteins or inhibiting nonenzymatic functions of enzymes.^30−33^ In addition, PROTACs can also offer possibilities to gain higher selectivity for protein degradation compared to their small molecule counterparts.^34,35^ Nevertheless, currently reported HDAC8 PROTACs either lack potency or selectivity among HDACs (Figure 1). The HDAC8-directed PROTACs **1**,^36^**2**,^37^**3**,^38^ and **5**(39) have modest potency, while PROTAC **4**(40) lacks selectivity between HDAC8 and HDAC6 degradation. This indicates the need for improved HDAC8 PROTACs.

In this study, we aimed for the development of a potent and selective HDAC8 PROTAC. For this, we set out to design several novel HDAC8 PROTACs, followed by the evaluation of their potential to degrade HDAC8 and explore the antiproliferative activities in various cell lines from hematological malignancies and solid cancers by the use of Western blot analysis, caspase 3/7 activity assay, flow cytometry, and MTS assay. This enabled the discovery of a potent and selective HDAC8 PROTAC **Z16** of which we characterized the effect on different antiproliferative mechanisms in hematological malignancies and solid cancers. Our results indicate that the antiproliferative activities of **Z16** is not due to HDAC8 degradation and **Z16** can be used as a chemical tool to investigate the functions of HDAC8.

## Results and Discussion

### Design of Hydrazide-based HDAC8 PROTACs

**Figure 2:**
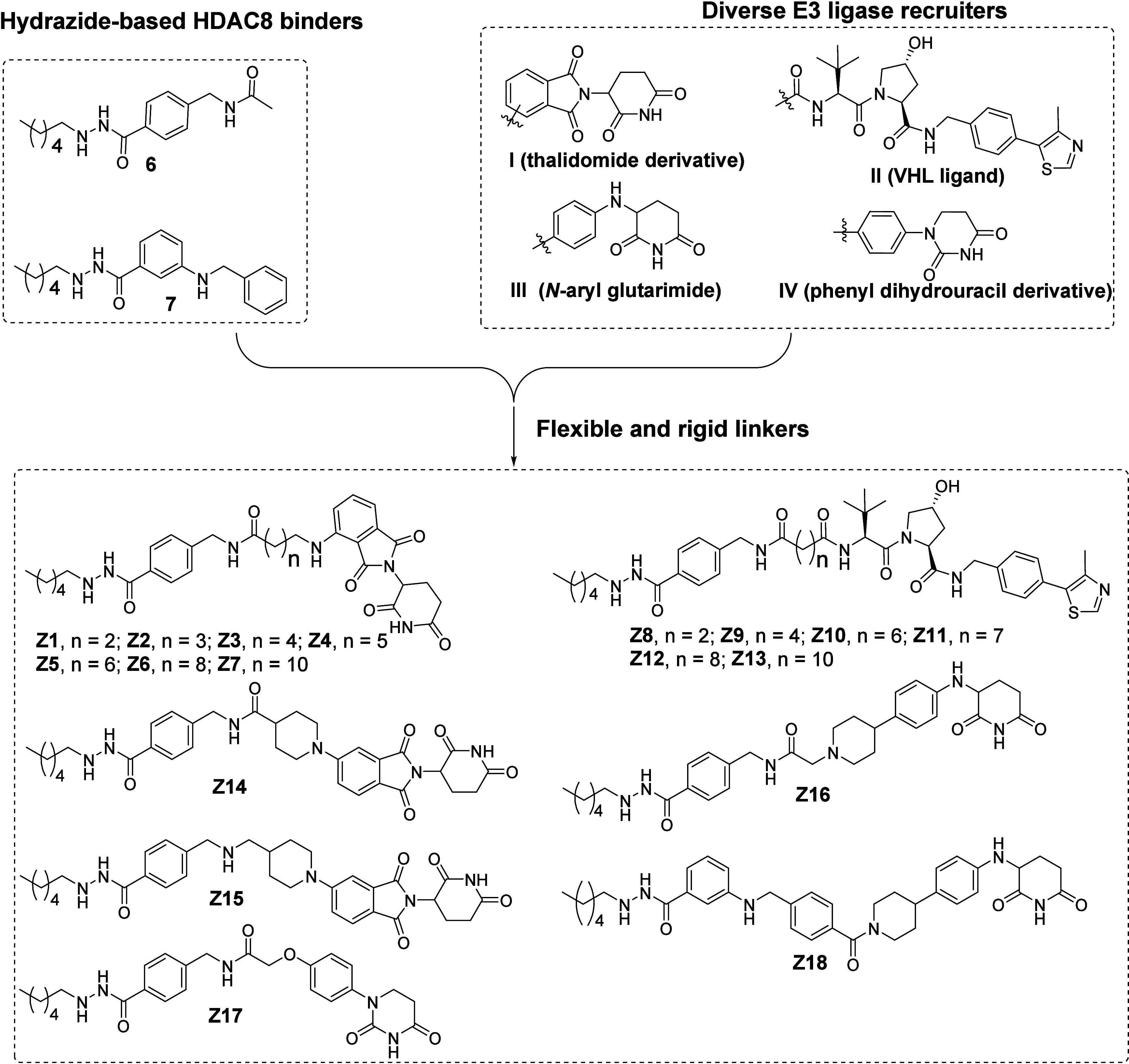
Design strategy of HDAC8 PROTACs. Compounds **Z1**–**Z7** and **Z8**–**Z13** were designed by linking compound **6** to CRBN ligand **I** and VHL ligand **II** via aliphatic linkers, respectively. Compounds **Z14** and **Z15** were designed by linking compound **6** to CRBN ligand **I** via rigid linkers. Connecting new CRBN ligand **III** and **IV** to compound **6** gave compounds **Z16** and **Z17**, respectively. Compound **Z18** was obtained by linking compound **7** to CRBN ligand **III**.

Currently reported HDAC8 PROTACs contain hydroxamic acids as zinc binding groups, which is connected to limited selectivity and concerns about the pharmacokinetic (PK) and safety profiles.^41−43^ Therefore, we employed the recently reported hydrazide-based HDAC8 inhibitor **6** as a starting point, because it shows potent HDAC8 inhibitory activity and excellent selectivity among other HDACs.^44^ Molecular docking studies of compound **6** in HDAC8 (PDB Code: 1T69),^45^ HDAC6 (PDB Code: 5EDU),^46^ and HDAC2 (PDB Code: 4LXZ)^47^ provided docking poses in which the hexyl-substituted hydrazide group can occupy the active pocket of HDAC8 and the acetyl group is solvent-exposed (Figure S1A in the Supporting Information). However, the hexyl-substituted hydrazide group protrudes from the active pockets of both HDAC6 and HDAC2 (Figure S1B and S1C in the Supporting Information, respectively), thus indicating that this functionality renders selective binding to HDAC8. These binding modes indicate that introduction of a linker to the acetyl group of compound **6** might be tolerated for HDAC8 binding. The cocrystal complex of thalidomide in cereblon (CRBN) (PDB Code: 4CL1)^48^ shows that the imide moiety of thalidomide occupies the active site of CRBN, meanwhile leaving the aromatic ring solvent-exposed (Figure S1D in the Supporting Information). In addition, the VHL ligand/VHL E3 ligase cocrystal complex (PDB Code: 4W9H)^49^ shows that the acetyl group protrudes out of the pocket and is solvent-exposed (Figure S1E in the Supporting Information). Based on these binding modes, compounds **Z1**–**Z7** were designed by linking compound **6** to the widely used CRBN ligand **I** via flexible aliphatic linkers as a starting point for exploration (Figure 2).^50^ Similarly, compounds **Z8**–**Z13** were designed by linking compound **6** to VHL E3 ligase ligand **II** (Figure 2), which is another widely used E3 ligand.^51^

It has been shown that application of rigid linkers enables improvement of the physicochemical properties, optimization of the degradation potency and reduction of the off-target effects.^52−54^ This inspired the design of compounds **Z14** and **Z15** by including rigid linkers between compound **6** and CRBN ligand **I** (Figure 2). Recently, *N*-aryl glutarimide **III** and phenyl dihydrouracil derivative **IV** have been successfully utilized as alternative CRBN ligands with improved resistance to hydrolysis and reduced off-target effects in PROTAC design.^55−58^ This also inspired us to employ these two new CRBN ligands in the design of **Z16** and **Z17**, respectively (Figure 2). As an alternative for HDAC8 ligand **6**, ligand **7**(44) was included in compound **Z18** (Figure 2).

### Chemistry

**Scheme 1:**
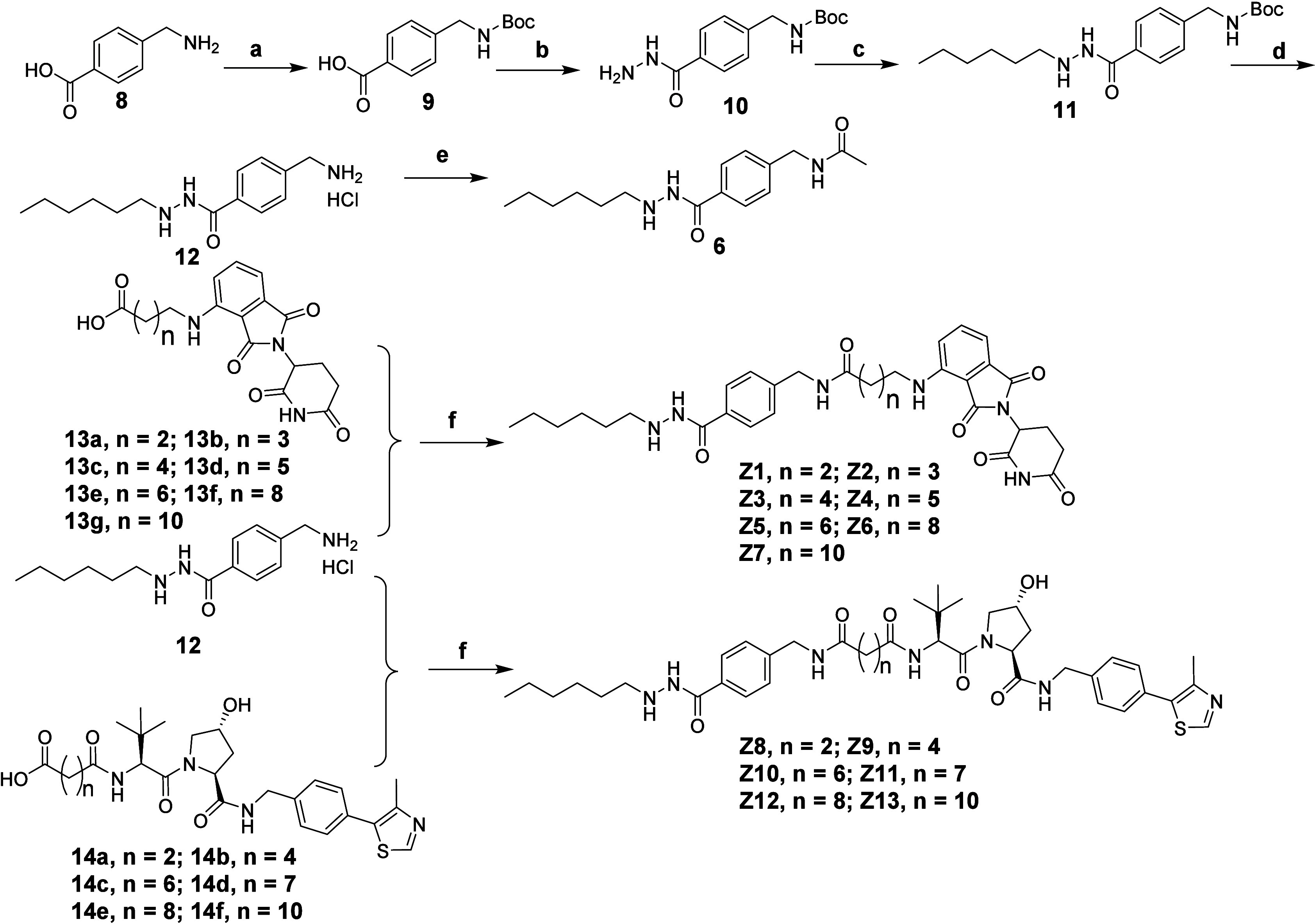
Synthetic Route of Compounds **Z1**–**Z13** Reagents and conditions: a) (Boc)_2_O, 1 M NaOH, THF/H_2_O, 94%; b) Hydrazine monohydrate, CDI, THF, 63%; c) Hexanal, NaBH_4_, MeOH, 57%; d) 4 N HCl in dioxane, DCM, 87%; e) Acetic acid, HATU, DIPEA, DMF, 44%; f) for **Z1**–**Z3**, EDCI, HOBT, TEA, DMF, 10–54%; for **Z4**–**Z13**, HATU, DIPEA, DMF, 17–49%.

The synthetic steps of compound **6**, CRBN-based PROTACs **Z1**–**Z7** and VHL-based PROTACs **Z8**–**Z13** were shown in Scheme 1. Boc-protection of starting material **8** under basic condition led to compound **9**, which provided compound **10** via CDI-mediated amide formation with hydrazine. Then, after reductive amination, compound **10** gave compound **11** followed by boc-deprotection under acidic condition to give intermediate **12**. Compound **6** was obtained by HATU-mediated amine formation between compound **12** and acetic acid. The intermediates **13a**–**13g** and **14a**–**14f** were synthesized according to reported methods.^59^ Compounds **Z1**–**Z7** and **Z8**–**Z13** were obtained via amide formation between compound **12** and intermediates **13a**–**13g** and **14a**–**14f**, respectively.

**Scheme 2:**
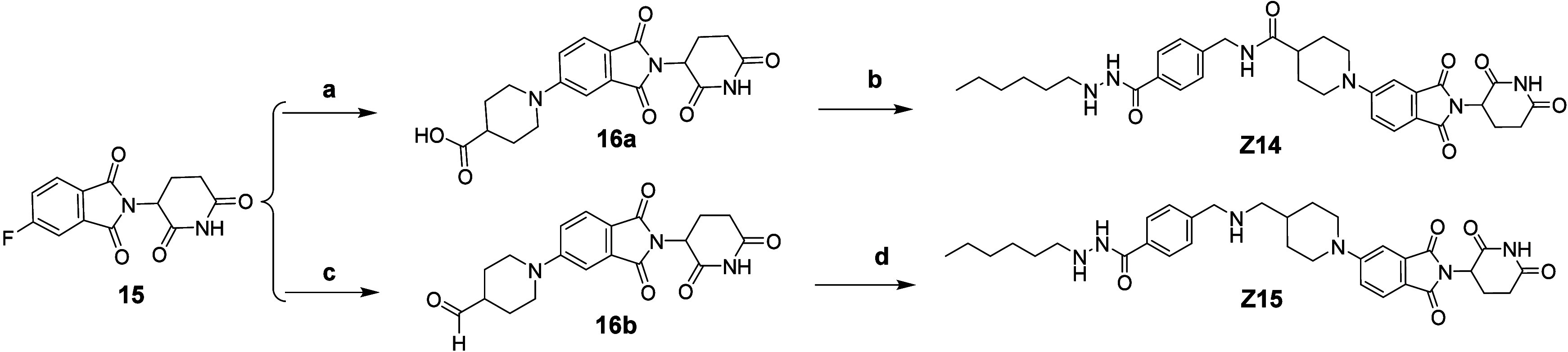
Synthetic Route of Compounds **Z14** and **Z15**a Reagents and conditions: a) (1) *tert*-butyl piperidine-4-carboxylate, DIPEA, DMSO, 90 °C; (2) TFA/DCM, 57% (two steps); b) **12**, HATU, DIPEA, DMF, 25%; c) (1) piperidin-4-ylmethanol, DIPEA, DMSO, 90 °C; (2) Dess-Martin Oxidant, DCM, 53% (two steps); d) **12**, NaBH(OAc)_3_, DIPEA, MeOH, 19%.

The synthetic steps for compounds **Z14** and **Z15** were shown in Scheme 2. Intermediate **15** reacted with *tert*-butyl piperidine-4-carboxylate, followed by the removal of *tert*-butyl group in the presence of TFA/DCM to give compound **16a**. Then, target compound **Z14** was obtained by amide formation between compounds **16a** and **12**. In addition, compound **15** reacted with piperidin-4-ylmethanol, followed by Dess–Martin oxidation to afford compound **16b**. Then, compound **16b** condensed with compound **12** to give target compound **Z15**.

**Scheme 3:**
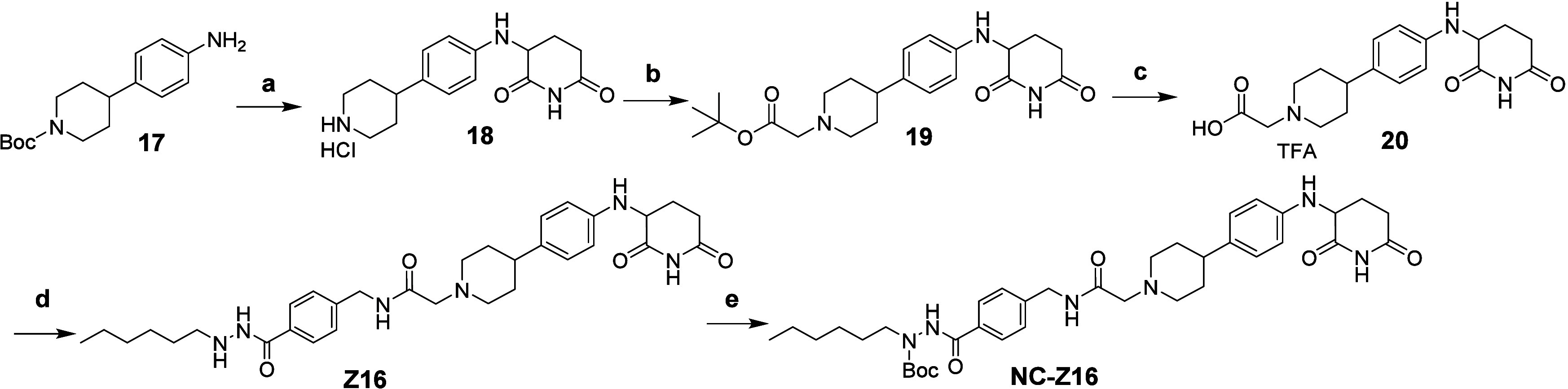
Synthetic Route of Compounds **Z16** and **NC-Z16** Reagents and conditions: a) (1) 3-bromopiperidine-2,6-dione, NaHCO_3_, DMF, 65 °C; (2) 4 N HCl in dioxane, DCM, 55% (two steps); b) *tert*-butyl 2-bromoacetate, DIPEA, ACN, 70 °C, 53%; c) TFA/DCM, 51%; d) **12**, HATU, DIPEA, DMF, 38%; e) (Boc)_2_O, TEA, THF, 50 °C, 38%.

The synthetic steps for compounds **Z16** and **NC-Z16** were shown in Scheme 3. After nucleophilic substitution reaction with 3-bromopiperidine-2,6-dione and boc-deprotection, compound **17** afforded compound **18**, which led to compound **19** via reaction with *tert*-butyl 2-bromoacetate. The removal of *tert*-butyl group of compound **19** afforded compound **20**, which was converted into target compound **Z16** via HATU-mediated amide formation with compound **12**. Then, boc-protection of compound **Z16** led to target compound **NC-Z16**.

**Scheme 4:**
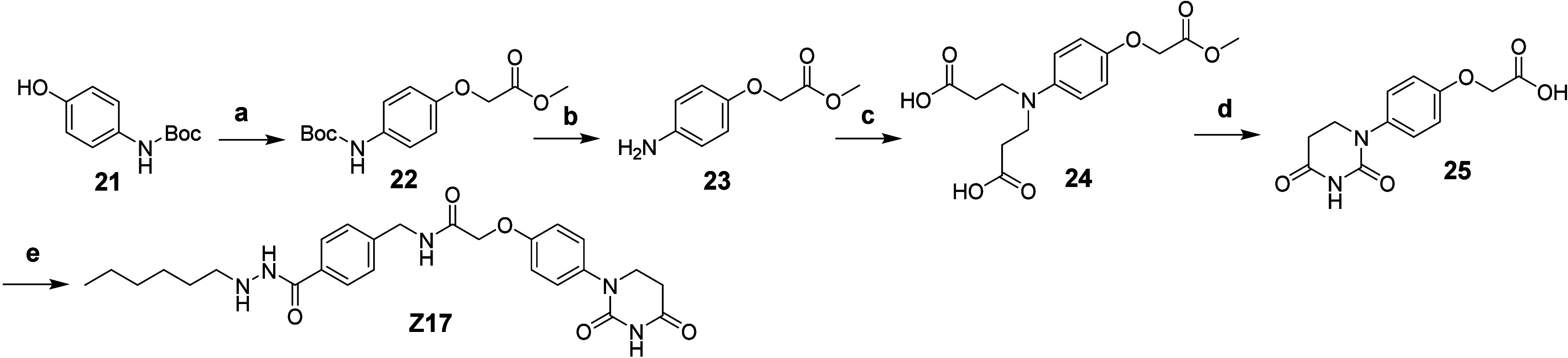
Synthetic Route of Compound **Z17** Reagents and conditions: a) methyl 2-bromoacetate, Cs_2_CO_3_, NaI, acetone, 60 °C; b) TFA/DCM, 97%; c) acrylic acid, H_2_O, 70 °C, 26%; d) (1) urea, acetic acid, 120 °C, (2) 4 N HCl (aq), 120 °C, 23% (two steps); e) **12**, HATU, DIPEA, DMF, 57%.

**Scheme 5:**
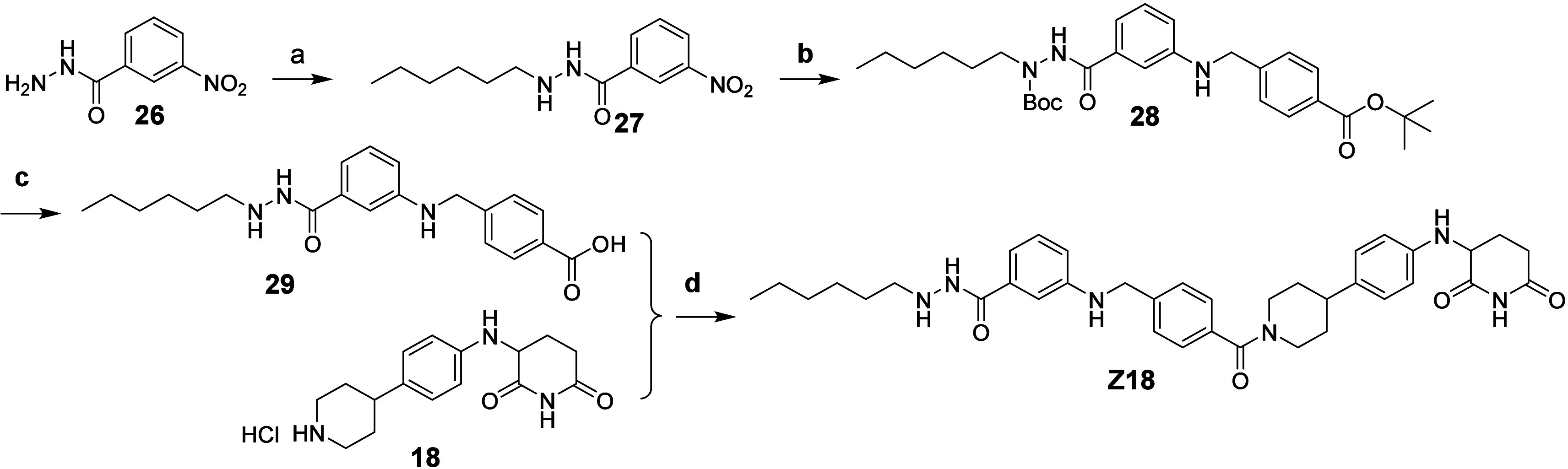
Synthetic route of compound **Z18** Reagents and conditions: a) Hexanal, NaBH_4_, MeOH, 72%; b) (1) (Boc)_2_O, TEA, THF, 50 °C; (2) Pd/C, H_2_, MeOH/EtOAc; (3) *tert*-butyl 4-(bromomethyl)benzoate, K_2_CO_3_, DMF, 51% (three steps); c) TFA/DCM, 88%; d) HATU, DIPEA, DMF, 28%.

**Scheme 6:**
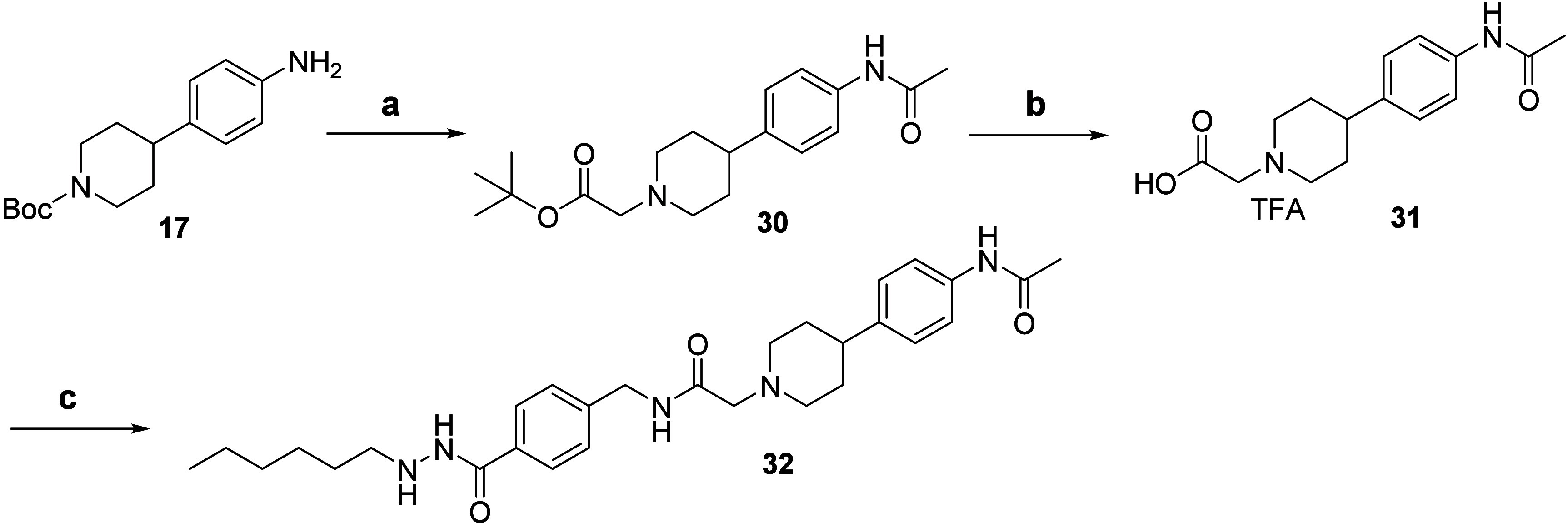
Synthetic route of compound **32** Reagents and conditions: a) (1) Acetyl chloride, TEA, THF; (2) TFA/DCM; (3) *tert*-butyl 2-bromoacetate, DIPEA, ACN, 70 °C, 47% (three steps); b) TFA/DCM, 73%; c) **12**, HATU, DIPEA, DMF, 26%.

The synthetic steps for compound **Z17**, **Z18**, and **32** were shown in Scheme 4, Scheme 5, and Scheme 6, respectively. In Scheme 4, after nucleophilic substitution reaction with methyl 2-bromoacetate, compound **21** afforded compound **22**, which provided compound **23** via boc-deprotection. Then, compound **23** reacted with acrylic acid to give compound **24**, which was converted into intermediate **25**. Afterward, target compound **Z17** was obtained via amide formation between compounds **25** and **12**. In Scheme 5, compound **26** gave compound **27** after reductive amination. After boc-protection, the reduction of nitro group and reaction with *tert*-butyl 4-(bromomethyl)benzoate, compound **27** afforded compound **28**. The removal of *tert*-butyl group of compound **28** led to the carboxylic acid **29**, which was converted into target compound **Z18** via amide formation. In Scheme 6, compound **17** gave compound **30** after condensation reaction with acetyl chloride, boc-deprotection and substitution reaction with *tert*-butyl 2-bromoacetate. The removal of *tert*-butyl group of compound **30** led to compound **31**, which was converted into final product **32** via HATU-mediated amide formation with intermediate **12**.

### Screening of HDAC8 PROTACs with Flexible Linkers

**Figure 3:**
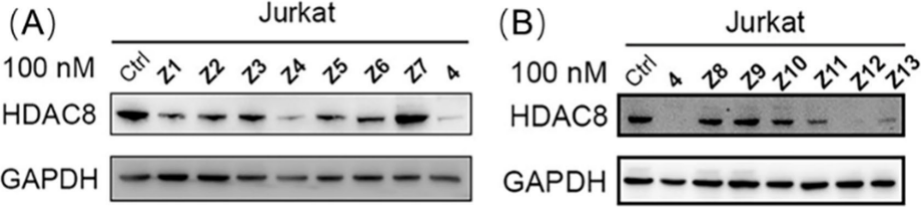
Jurkat cells were treated with 100 nM of PROTACs **Z1-Z7** and **4** (A), and **Z8**–**Z13** and **4** (B) for 6 h. HDAC8 level was detected using Western blot. GAPDH was used as a loading control.

**Figure 4:**
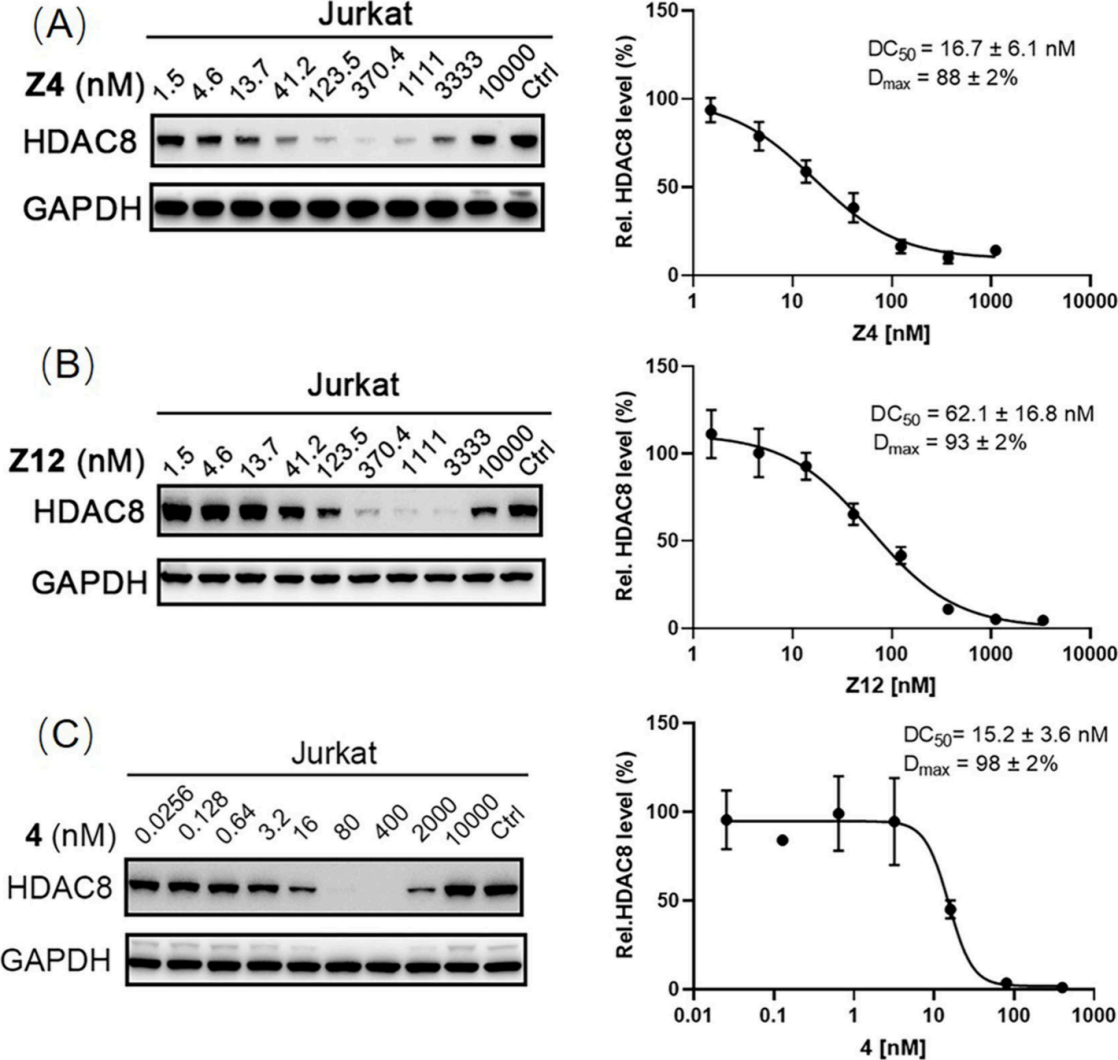
Jurkat cells were treated with indicated concentrations of compounds **Z4** (A), **Z12** (B), and **4** (C) for 6 h. HDAC8 levels were detected using Western blot. GAPDH was used as a loading control. Data were normalized to the DMSO-treated group and the dot plots were shown as mean ± SEM of at least two independent experiments. Nonlinear fitting was generated by the GraphPad Prism.

After synthesizing the potential HDAC8 PROTACs, their ability to trigger HDAC8 degradation was evaluated at a single concentration and compared to the previously developed HDAC8 PROTAC **4**.^40^ T-cell leukemia Jurkat cells were treated with the CRBN-based PROTACs **Z1**–**Z7** and the VHL-based PROTACs **Z8**–**Z13** at a concentration of 100 nM for 6 h, respectively and the HDAC8 levels were analyzed using Western blot. The results show that the CRBN-based PROTACs **Z1**–**Z6** provided HDAC8 degradation ranging from 43% to 90%, while the CRBN-based PROTAC **Z7** did not trigger visible degradation (Table 1 and Figure 3). Also, the PROTACs **Z8**–**Z13** decreased HDAC8 levels by 14% to 83% compared to the DMSO-treated group (Table 1 and Figure 3B). Taking this together, the initial screening indicates that compounds **Z4** and **Z12** demonstrated a HDAC8 degradation efficiency that is comparable with PROTAC **4** at 100 nM. For these compounds, the concentration dependence of HDAC8 degradation was investigated. Western blot analysis and quantification showed DC_50_ values in the low nanomolar range. **Z4** provided a DC_50_ value of 16.7 nM and a *D*_max_ of 88%, whereas compound **Z12** gave a DC_50_ value of 62.1 nM and a *D*_max_ of 93% as shown in Figure 4.

**Table 1:** HDAC8 Degradation Efficiency for PROTACs **Z1**–**Z13**

ID	Degradation (%) at 100 nM^a^	ID	Degradation (%) at 100 nM^a^
**Z1**	73 ± 1	**Z8**	20 ± 1
**Z2**	62 ± 2	**Z9**	17 ± 8
**Z3**^c^	44 ± 13	**Z10**	14 ± 5
**Z4**	90 ± 10	**Z11**	48 ± 4
**Z5**	43 ± 17	**Z12**	83 ± 2
**Z6**	43 ± 3	**Z13**	65 ± 5
**Z7**^c^	n.d^b^	**4**	88 ± 2

a HDAC8 degradation percentage is represented as mean ± standard error of mean (SEM) of at least two independent experiments.

b No degradation.

c The impurities of **Z3** and **Z7** (both 93.8% pure) might affect their degradation potency.

### Screening of HDAC8 PROTACs with Rigid Linkers

**Figure 5:**
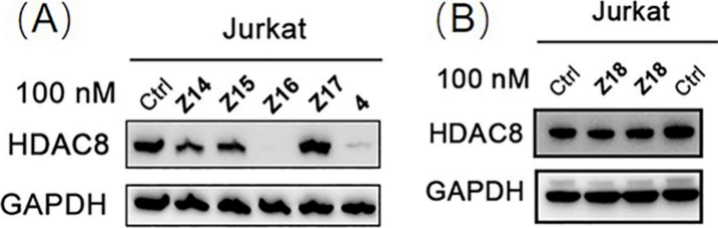
Jurkat cells were treated with 100 nM of PROTACs **Z14**–**Z17** and **4** (A), and **Z18** (B) for 6 h. HDAC8 level was detected using Western blot. GAPDH was used as a loading control.

To obtain more potent HDAC8 PROTACs, a new round of optimization was performed by synthesis of compounds **Z14**–**Z18** that include rigid linkers and novel CRBN ligands (Figure 2). Similarly, Jurkat cells were treated with compounds **Z14**–**Z18** at 100 nM for 6 h and the HDAC8 levels were determined using Western blot. However, only *N*-aryl glutarimide-based compound **Z16** decreased HDAC8 levels by 92%, while the other compounds showed less HDAC8 degradation (Table 2 and Figure 5).

**Table 2:** HDAC8 Degradation Efficiency for PROTACs **Z14**–**Z18**

ID	Degradation (%) at 100 nM^a^
**Z14**	34 ± 3
**Z15**	25 ± 2
**Z16** (**CZH-726**)	92 ± 8
**Z17**	n.d^b^
**Z18**	13 ± 4
**4**	88 ± 2

a HDAC8 degradation percentage is represented as mean ± SEM of at least two independent experiments.

b No degradation.

Because compound **Z16** stood out as the most potent HDAC8 PROTAC among these series of compounds, the HDAC inhibition profile of **Z16** was explored. **Z16** showed much less potent HDAC1/2/3 inhibitory activities than SAHA, approximately 4-fold less and much less in HDAC8 inhibition compared to **6** and PCI-34051, respectively (Table 3 and Figure S2 in the Supporting Information).

**Table 3:** HDAC1/2/3/8 Inhibitory Activities of PROTAC **Z16**^a^

	IC_50_ (μM)			
ID	HDAC1	HDAC2	HDAC3	HDAC8
**Z16**	1.7 ± 0.3	20.1 ± 4.6	1.2 ± 0.2	2.7 ± 0.9
**6**	0.94 ± 0.05	8.0 ± 1.0	9.5 ± 0.7	0.60 ± 0.08
SAHA	0.016 ± 0.002	0.14 ± 0.02	0.017 ± 0.001	–
PCI-34051	–	–	–	0.04 ± 0.003

a Data are shown as mean ± SEM of three independent experiments.

**Figure 6:**
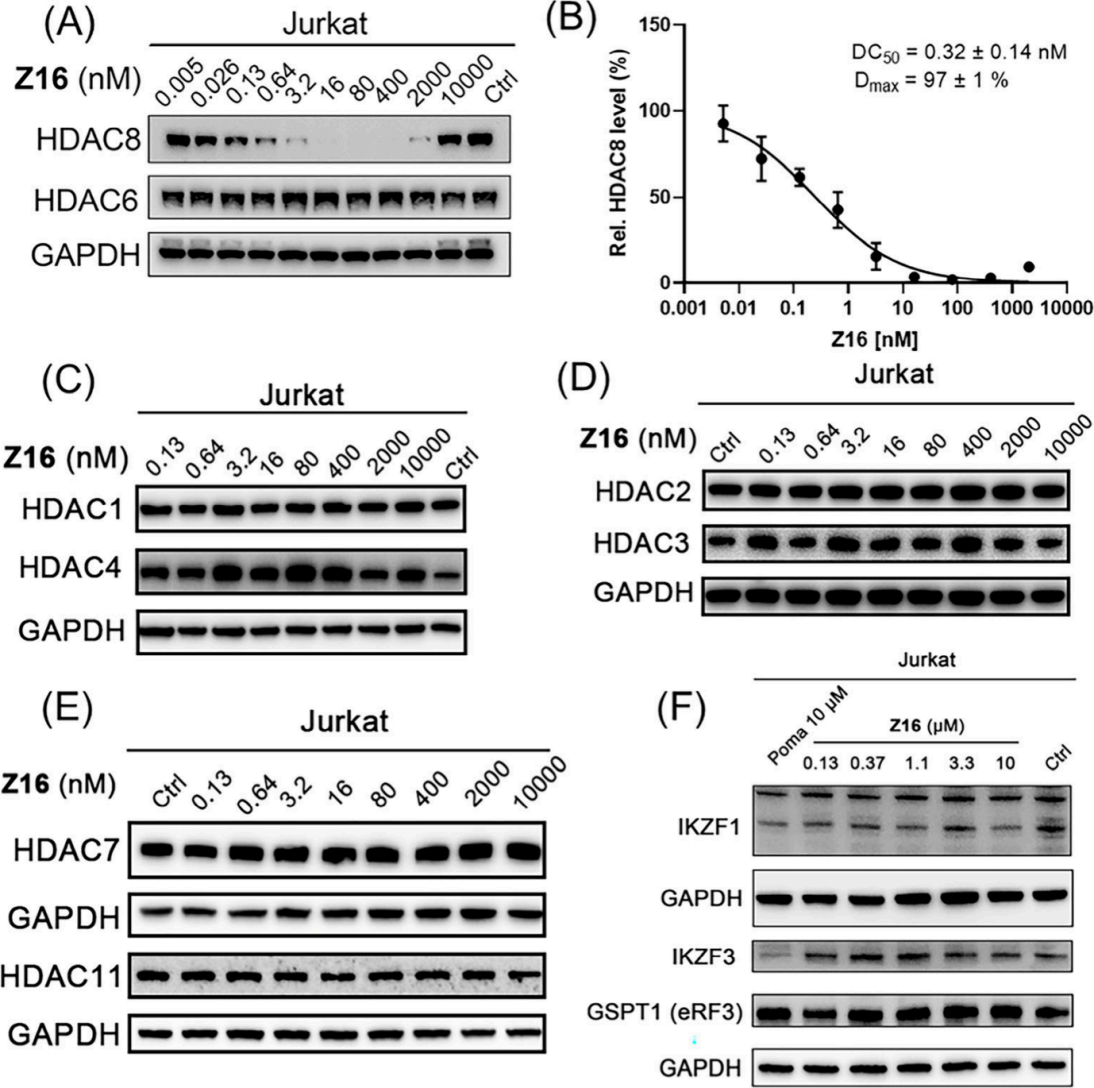
Jurkat cells were treated with indicated concentrations of **Z16**, and 10 μM pomalidomide (Poma) for 6 h. HDAC1/2/3/4/6/7/8/11 levels and IKZF1/IKZF3/GSPT1 levels were detected using Western blot. GAPDH was used as a loading control. HDAC8 levels were normalized to the DMSO-treated group and the dot plots were shown as mean ± SEM of at least two independent experiments. Nonlinear fitting was generated by the GraphPad Prism.

Compound **Z16** with potent HDAC8 degradation at 100 nM was selected for further investigation. The dose-dependency, as shown in Figure 6A and 6B, demonstrates that PROTAC **Z16** effectively induced HDAC8 degradation with a DC_50_ of 0.32 nM and a *D*_max_ of 97% in Jurkat cells after 6 h treatment. In addition, an obvious hook effect was observed at a concentration of 10 μM in Jurkat cells (Figure 6A). Importantly, no obvious HDAC1 (Figure 6C), HDAC2 (Figure 6D), HDAC3 (Figure 6D), HDAC4 (Figure 6C), HDAC6 (Figure 6A), HDAC7 (Figure 6E), and HDAC11 (Figure 6E) degradation was observed on Western blot upon **Z16** treatment, thus indicating good selectivity among HDAC isoenzymes. Additionally, **Z16** did not significantly affect the intracellular levels of IKZF1, IKZF3, and GSPT1 in Jurkat cells (Figure 6F), which are the neo-substrates of CRBN.^60,61^

**Figure 7:**
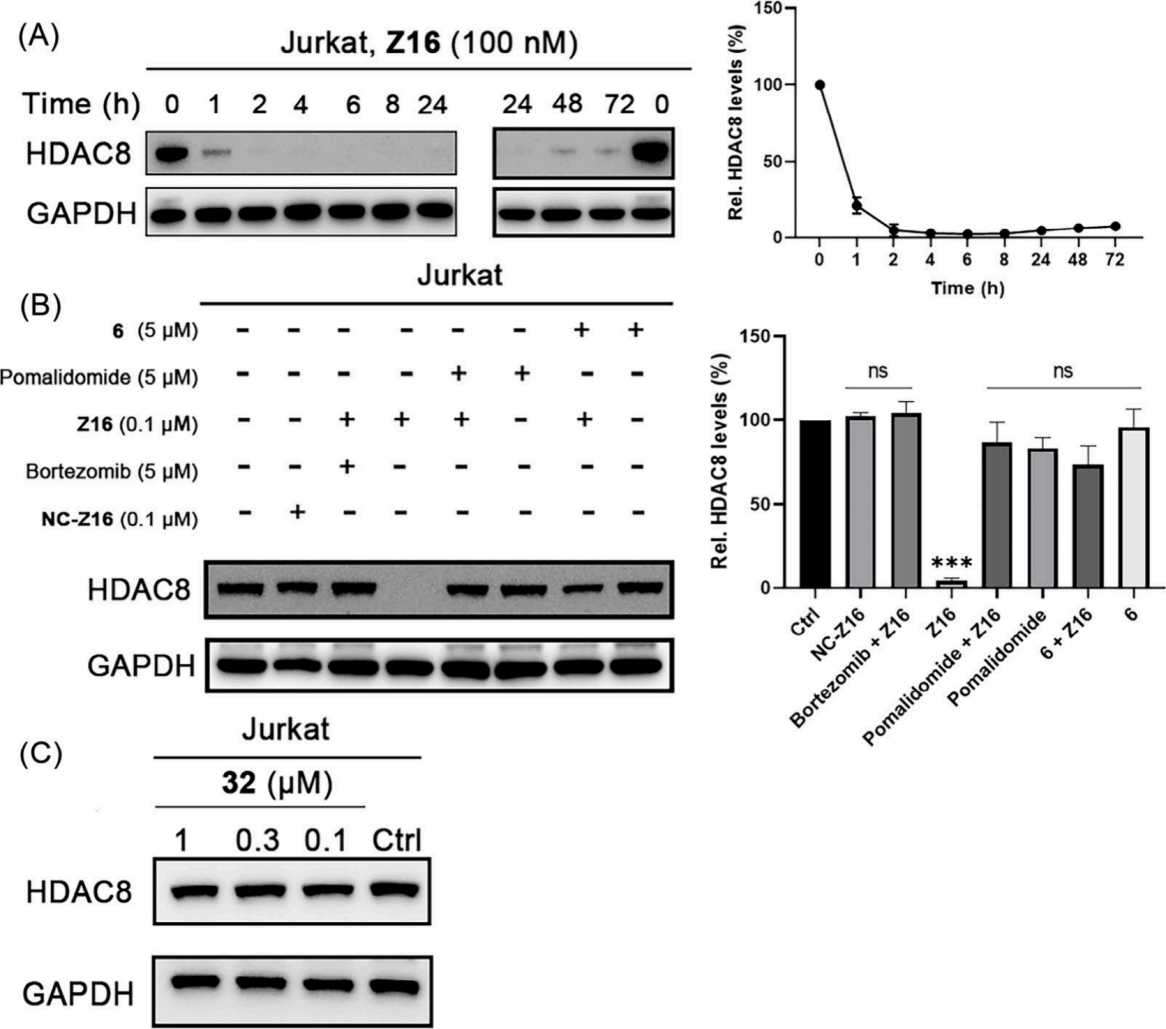
(A) Degradation kinetics of HDAC8 by PROTAC **Z16** in Jurkat cells. Jurkat cells were treated with 100 nM of **Z16** for the indicated time. HDAC8 levels were detected using Western blot and quantified using ImageJ. GAPDH was used as a loading control. Data are shown as mean ± SEM of two independent experiments. (B) Mechanistic investigation of HDAC8 degradation induced by PROTAC **Z16** in Jurkat cells. Jurkat cells were pretreated with 5 μM of HDAC8 inhibitor **6**, CRBN ligand pomalidomide, and proteasome inhibitor bortezomib for 1 h, followed by treatment of **Z16** at 100 nM for 6 h. Jurkat cells were treated with 100 nM of **Z16** and **NC-Z16** for 6 h. (C) Jurkat cells were treated with indicated concentrations of **32** for 6 h. HDAC8 levels were detected using Western blot. GAPDH was used as a loading control. Data are represented as mean ± SEM of two independent experiments. ns: not significant, *P < 0.05, **P < 0.01, ***P < 0.001, and ****P < 0.0001 vs DMSO-treated group, one-way analysis of variance (ANOVA).

The ability of **Z16** to induce HDAC8 degradation was explored further in Jurkat cells. Analysis of the kinetics of HDAC8 degradation upon treatment with 100 nM **Z16** showed rapid and efficient degradation after one or 2 h, which remained for up to 3 days (Figure 7A). The effect of **Z16**-induced HDAC8 degradation was diminished upon pretreatment with HDAC8 inhibitor **6** (5 μM), CRBN ligand pomalidomide (5 μM), or the proteasome inhibitor bortezomib (5 μM). Furthermore, control compound **NC-Z16** (Scheme 3) was synthesized in which the HDAC8 ligand was Boc-protected to block HDAC8 binding, which showed over 6-fold decreased HDAC8 binding affinity (HDAC8 inhibition IC_50_ = 16.6 μM, Figure S2 in the Supporting Information) compared to **Z16**. In addition, we also synthesized another control compound **32** (Scheme 6) in which the glutarimide group of **Z16** was replaced by an acetyl group to block CRBN binding. Neither **NC-Z16** nor **32** induced HDAC8 degradation, thus confirming the critical role for both HDAC8 and CRBN binding in the activity of **Z16**-induced HDAC8 degradation (Figure 7B and 7C). Based on these results, we can conclude that HDAC8 degradation induced by **Z16** proceeds via formation of a ternary complex and depends on proteasomal activity.

**Figure 8:**
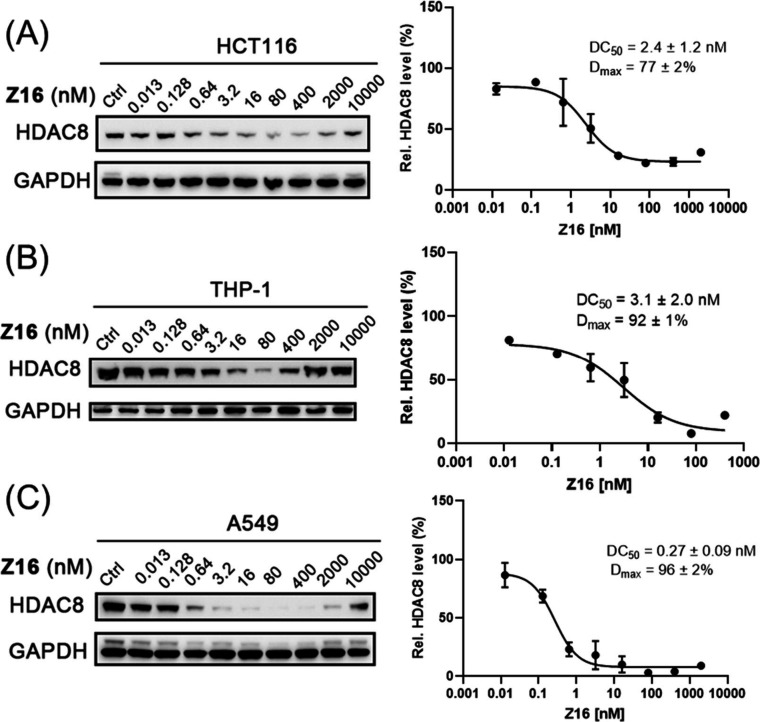
HCT116 (A), THP-1 (B), and A549 (C) cells were treated with the indicated concentrations of compound **Z16** for 6 h. HDAC8 levels were detected using Western blot. GAPDH was used as a loading control. Data were normalized to the DMSO-treated group and the dot plots were shown as mean ± SEM of at least two independent experiments. Nonlinear fitting was generated by the GraphPad Prism.

In addition, **Z16** treatment for 6 h effectively induced HDAC8 degradation in colon cancer cells HCT116 (DC_50_ = 2.4 nM, *D*_max_ = 77%) without significant impact on HDAC1 and HDAC6 (Figure 8A and S3 in the Supporting Information). Furthermore, similar effects were observed in AML THP-1 cells (DC_50_ = 3.1 nM, *D*_max_ = 92%) (Figure 8B), and lung cancer A549 cells (DC_50_ = 0.27 nM, *D*_max_ = 96%) (Figure 8C). Taken together, we conclude that **Z16** is an effective and potent HDAC8 PROTAC with good selectivity over HDAC1/2/3/4/6/7/11.

### HDAC8 PROTAC Z16 is Active in Hematological and Solid Cancer Cell Lines

Because **Z16** could effectively induce HDAC8 degradation in Jurkat, THP-1, HCT116, and A549 cells, the antiproliferative activities of **Z16** against these four cell lines were investigated. **Z16** has antiproliferative activity against Jurkat, THP-1, HCT116, and A549 cells with IC_50_ values of 1.7 μM, 1.8 μM, 1.4 μM, and 7.7 μM, respectively, as shown in Table 4 and Figure S4 in the Supporting Information. Importantly, **Z16** showed more potent antiproliferative activities against all the four cell lines than PROTAC **4**. Nevertheless, the pan-HDAC inhibitor SAHA and the HDAC1/2/3 inhibitor MS-275 are about 2-fold more potent. In comparison, the HDAC8 inhibitor **6**, CRBN ligand pomalidomide and the combination treatment of HDAC8 inhibitor **6** plus CRBN ligand **19** provided IC_50_ values of over 33.3 μM against all the four cell lines, which implicates that the hydrazide HDAC inhibitor **6** and the CRBN ligand are not cytotoxic on their own. To further confirm the importance of the HDAC8 binding, the cells were also treated with **NC-Z16**, which provided much less potency compared to PROTAC **Z16**. However, control compound **32** without effective CRBN ligand showed slightly less potent antiproliferative activities against Jurkat, HCT116, THP-1 and A549 cells. Interestingly, the well-known HDAC8 selective inhibitor PCI-34051 specially demonstrated antiproliferative activities against Jurkat, but not THP-1, HCT116, and A549 cells. In addition, we also investigated the antiproliferative activity of **Z16** against HEL cells, which are less HDAC8-dependent leukemia cells (depmap.org). As shown in Figure S4 in the Supporting Information, **Z16** provided an IC_50_ value of 7.7 μM, which was about 10-fold less potent than SAHA (IC_50_ = 0.72 μM). Overall, HDAC8 PROTAC **Z16** demonstrates potent antiproliferative activities against cell lines of both hematological malignancies and solid cancers and is more potent than the HDAC8 inhibitor **6**, previously reported PROTAC **4**, comparable with compound **32**, SAHA, and MS-275.

**Table 4:** Antiproliferative Activities of PROTAC **Z16** against Both Hematological Malignancies and Solid Cancers^a^

	IC_50_ (μM)			
	Hematological malignancies		Solid cancers	
ID	Jurkat	THP-1	HCT116	A549
**Z16**	1.7 ± 0.2	1.8 ± 0.3	1.4 ± 0.1	7.7 ± 0.8
**4**	4.0 ± 0.8	10.8 ± 0.9	8.0 ± 0.5	15.2 ± 3.3
**6**	>33.3	>33.3	>33.3	>33.3
**32**	3.0 ± 0.3	3.4 ± 0.6	2.8 ± 0.3	12.0 ± 1.6
**6 + 19**(1:1)	>33.3	>33.3	>33.3	>33.3
**PCI-34051**	4.5 ± 0.7	>33.3	21.9 ± 2.8	>33.3
**Poma**	>33.3	>33.3	>33.3	>33.3
**NC-Z16**	19.5 ± 2.9	>33.3	30.1 ± 8.6	32.9 ± 0.4
**SAHA**	0.38 ± 0.05	0.62 ± 0.05	0.72 ± 0.05	3.5 ± 0.3
**MS-275**	0.28 ± 0.05	2.3. ± 0.3	0.56 ± 0.02	4.9 ± 0.5

a Cells were treated with indicated compounds for 72 h. Cell viability was determined by MTS assay. Data are shown as mean ± SEM of three independent experiments.

### HDAC8 PROTAC Z16 Induced Both SMC3 and Histone Hyperacetylation

**Figure 9:**
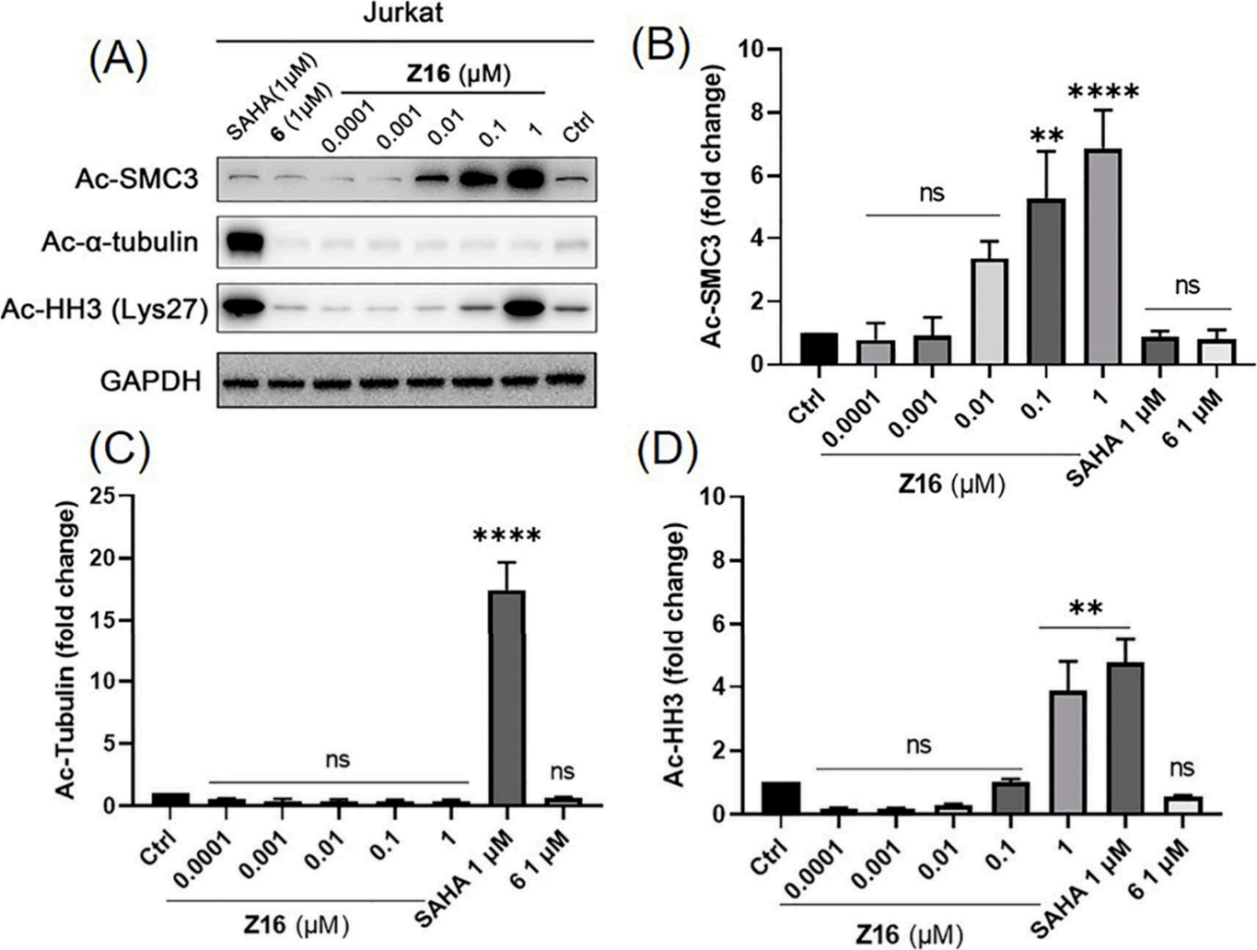
(A) Jurkat cells were treated with indicated concentrations of compound **Z16**, **6**, and SAHA for 6 h. The levels of Ac-SMC3, Ac-Tubulin, and Ac-HH3 were detected using Western blot. GAPDH was used as a loading control. The levels of Ac-SMC3 (B), Ac-Tubulin (C), and Ac-HH3 (D) were quantified using ImageJ and normalized to the DMSO-treated group. Data are shown as mean ± SEM of two independent experiments. ns: not significant, *P < 0.05, **P < 0.01, ***P < 0.001, and ****P < 0.0001 vs DMSO-treated group, one-way analysis of variance (ANOVA).

After confirming that **Z16** is a potent HDAC8 PROTAC, we investigated the effects on acetylation of HDAC intracellular substrates. **Z16** dose-dependently increased the levels of HDAC8 substrate acetyl-SMC3 (Ac-SMC3) as shown in Figure 9A and 9B after 6 h treatment. It is worth noting that 0.01 μM of **Z16** could lead to obvious increased Ac-SMC3, while neither 1 μM of pan-HDAC inhibitor SAHA nor 1 μM of HDAC8 inhibitor **6** increased the level of Ac-SMC3 (Figure 9A and 9B). In contrast, increased levels of the HDAC6 substrate acetyl-α-tubulin (Ac-Tubulin) were only observed in the SAHA-treated group but not in the **Z16**-treated group, suggesting that **Z16** did not significantly impact HDAC6 activity (Figure 9A and 9C). It is worth noting both SAHA and **Z16** at 1 μM significantly increase the levels of class I HDAC substrate acetyl-histone H3 (Ac-HH3) (Figure 9A and 9D).

**Figure 10:**
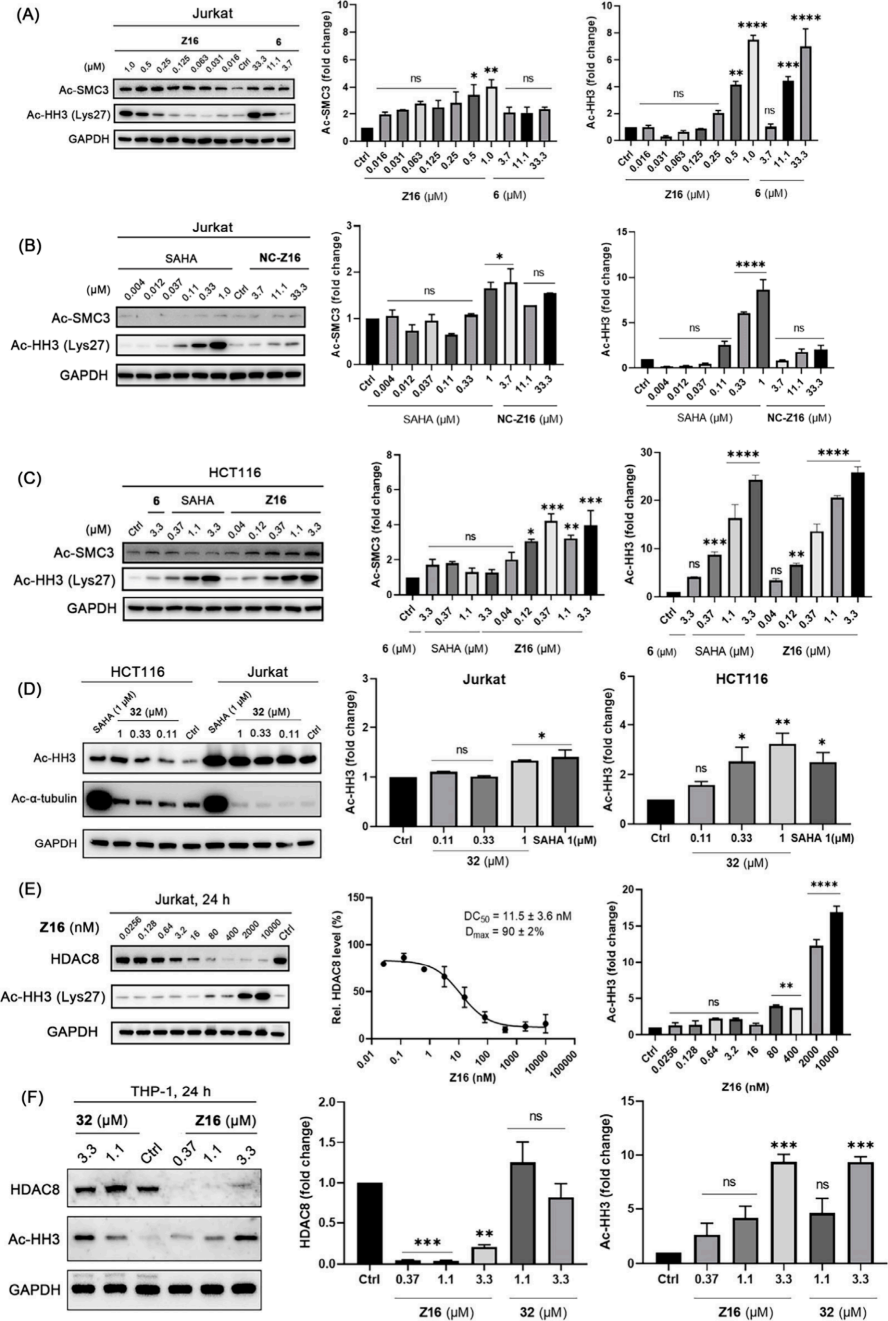
Jurkat cells were treated with indicated concentrations of compound **Z16**, **6** (A) and SAHA, **NC-Z16** (B) for 6 h, respectively. (C) HCT116 cells were treated with indicated concentrations of compound **Z16**, **6** and SAHA for 6 h. (D) Jurkat and HCT116 cells were treated with indicated concentrations of **32** for 6 h. (E) Jurkat cells were treated with indicated concentrations of compound **Z16** for 24 h. (F) THP-1 cells were treated with indicated concentrations of **Z16** and **32** for 24 h. The levels of Ac-SMC3, Ac-HH3, Ac-tubulin, and HDAC8 were detected using Western blot. GAPDH was used as a loading control. The levels of Ac-SMC3, Ac-HH3 and HDAC8 were quantified using ImageJ and normalized to the DMSO-treated group. Data are shown as mean ± SEM of two independent experiments. Nonlinear fitting was generated by the GraphPad Prism.ns: not significant, *P < 0.05, **P < 0.01, ***P < 0.001, and ****P < 0.0001 vs DMSO-treated group, one-way analysis of variance (ANOVA).

In order to further investigate the effects of **Z16** on the levels of Ac-SMC3 and Ac-HH3, Jurkat cells were treated with multiple concentration of **Z16**, **6**, SAHA, and **NC-Z16** for 6 h. The results are shown in Figure 10A. **Z16** treatment increased the levels of both Ac-SMC3 and Ac-HH3 in a dose-dependent manner. Similarly, HDAC8 inhibitor **6** treatment increased the levels of both Ac-SMC3 and Ac-HH3 at higher concentrations (Figure 10A). The increased Ac-HH3 levels were also observed in SAHA-treated group but not in **NC-Z16**-treated group (Figure 10B). Additionally, **Z16** could also increase the levels of both Ac-SMC3 and Ac-HH3 in a dose-dependent manner in HCT116 cell lines (Figure 10C). However, the dose-dependent increase of Ac-HH3 levels and no significant impact on Ac-tubulin levels were also observed in both Jurkat and HCT116 cells upon compound **32** treatment (Figure 10D). However, neither Ac-HH3 levels nor Ac-HH4 levels were significantly increased in Jurkat cells upon selective HDAC8 inhibitor PCI-34051 treatment (0.4 μM – 33.3 μM), which indicates that the antiproliferative activity of PCI-34051 against Jurkat cells is not due to histone hyperacetylation (Figure S5A in the Supporting Information). Next, in order to investigate if the antiproliferative activity of PCI-34051 against Jurkat is due to HDAC8 inhibition, Jurkat cells were pretreated with 50 nM **Z16** for 2 h to decrease HDAC8 levels before PCI-34051 treatment. Interestingly, the pretreatment of **Z16** led to about 5-fold decreased potency compared to PCI-34051 single treatment. (Figure S5B in the Supporting Information), which suggests that HDAC8 inhibition by PCI-34051 can inhibit Jurkat cells growth. PCI-34051 might affect the nonhistone substrates of HDAC8, leading to the antiproliferative activity against Jurkat cells. However, the low concentrations of **Z16** in which effectively triggers HDAC8 degradation did not affect cell viability as assessed by MTS assay. Taken together, **Z16** treatment affect both SMC3 and histone H3 acetylation, while the **Z16** concentrations that induce SMC3 acetylation are much lower than the concentrations that induce histone H3 acetylation in Jurkat cells.

As a next step, we compared the concentration dependence of **Z16**-induced HDAC8 degradation to the concentration of **Z16**-induced histone H3 hyperacetylation. We observed a DC_50_ value of 11.5 nM and a *D*_max_ of 90% (Figure 10E) for HDAC8 degradation after 24 h treatment of **Z16** in Jurkat cells. In addition, **Z16** also effectively triggered HDAC8 degradation in THP-1 cells after 24 h treatment. (Figure 10F). Interestingly, we did not observe an obvious hook effect at the concentration of >2 μM in both Jurkat and THP-1 cells compared to 6 h treatment (Figure 6A and 8B). This indicates that **Z16** might not be stable after 24 h treatment, which can explain the time-dependence of the hook effect. In comparison, **Z16** dose-dependently increased the level of Ac-HH3 in Jurkat cells (Figure 10E) at concentration of about 8-fold higher. Similarly, the Ac-HH3 levels increased in both THP-1 (Figure 10F) and HCT116 cells (Figure S6A in the Supporting Information) upon **Z16** treatment. Moreover, compound **32** showed comparable ability to induce histone H3 hyperacetylation in THP-1 cells (Figure 10F). In order to investigate if **Z16** could still effectively trigger HDAC8 degradation after 48 h treatment, Jurkat cells were treated with **Z16**, SAHA, and **6** for 48 h. As shown in Figure S6B in the Supporting Information, **Z16** could still effectively trigger HDAC8 degradation, while **SAHA** and **6** did not show significant impact on HDAC8 levels. It is worth noting that both **Z16** and SAHA could lead to histone hyperacetylation after 48 h treatment, while HDAC8 inhibitor **6** did not show significant impact on both Ac-HH3 and Ac-HH4 (Figure S6B and S6C in the Supporting Information). Overall, these results indicate that **Z16** induces histone hyperacetylation.

**Figure 11:**
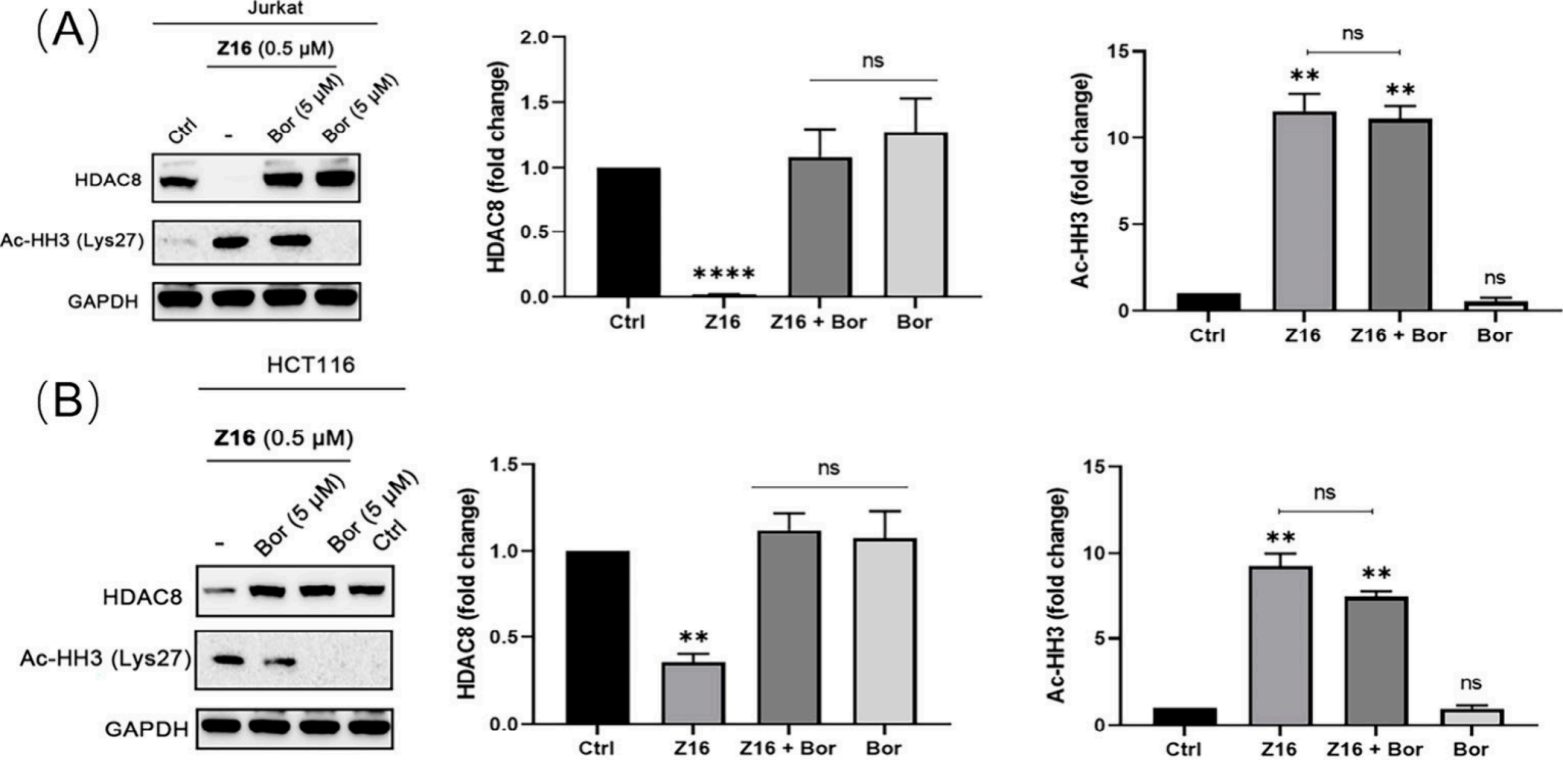
Jurkat (A) and HCT116 (B) were either pretreated with 5 μM bortezomib (Bor) for 1 h, followed by treatment with 0.5 μM **Z16** or treated with 0.5 μM **Z16** for 6 h. The levels of Ac-HH3 and HDAC8 were detected using Western blot. GAPDH was used as a loading control. The levels of Ac-HH3 and HDAC8 were quantified using ImageJ and normalized to the DMSO-treated group. Data are shown as mean ± SEM of two independent experiments. ns: not significant, *P < 0.05, **P < 0.01, ***P < 0.001, and ****P < 0.0001 vs DMSO-treated group, one-way analysis of variance (ANOVA).

Generally, histones are considered as class I HDAC substrates, but it remains controversial if histones are the bona fide HDAC8 substrates in vivo.^12,14^ The HDAC8 degradation efficiency of the **Z16** has a DC_50_ value of 0.32 nM, which means that 90% efficiency is reached at 10–100 nM concentrations after 6 h treatment in Jurkat cells (Figure 6A). If we inspect substrate selectivity profile in Jurkat cells, we observed that the increased acetylation of SMC3 started around 10 nM (Figure 9A and 10A), whereas increased acetylation of histone H3 started at concentration of about factor 10 higher (Figure 10A). **Z16** provided a DC_50_ value of 2.4 nM and *D*_max_ of 77% at the concentration of about 80 nM in HCT116 cells (Figure 8A). If we inspect the substrate selectivity profile in HCT116 cells, we observed that the increased acetylation of both SMC3 and histone H3 started at concentration of 40 nM after 6 h treatment (Figure 10C). In order to further confirm whether HDAC8 degradation is involved in histone hyperacetylation, Jurkat and HCT116 cells were pretreated with 5 μM proteasome inhibitor bortezomib, followed by treatment of 0.5 μM **Z16**. As shown in Figure 11, pretreatment with bortezomib could effectively block HDAC8 degradation induced by **Z16**, but failed to inhibit the histone hyperacetylation in both Jurkat and HCT116 cells even though **Z16** treatment group showed slightly higher Ac-HH3 levels than **Z16** plus bortezomib treatment group. Similar results were also observed in A549 cells upon **Z16** treatment (Figure S7 in the Supporting Information). Based on the results above, we can conclude that the SMC3 acetylation, as an HDAC8 specific effect, can be observed at low concentration around 10 nM, whereas higher concentrations of **Z16** might also start to affect the activity of other HDACs, which can be explained by the observed effects on histone hyperacetylation of control compound **32**, selective HDAC8 inhibitor PCI-34051 and **Z16** plus bortezomib cotreatment. Taken together, the HDAC1/2/3/8 deacetylase inhibition data (Table 3) and Western blot results for HDACs, acetylation of SMC3, histone H3 and H4 (Figure 6, 9,10 and 11, S5A and S6 in the Supporting Information), we can conclude that **Z16** induced SMC3 hyperacetylation at low concentrations and histone hyperacetylation at high concentrations, which can be explained by HDAC8 degradation and off-target HDAC inhibition, respectively.

We also explored a potential by-stander effect of HDAC8 degradation. It has been reported that HDAC8 can form a corepressor complex with STAT3 and release of the HDAC8 corepressor complex can recruit p300, Sp3, and other factors that activate gene transcription in colon cancer HCT116 cells.^20^ Next, we investigated if **Z16** could also trigger STAT3 degradation. However, Western blot results showed that **Z16** did not induce STAT3 degradation in both Jurkat and HCT116 cells (Figure S8 in the Supporting Information).

### Antiproliferative Mechanism of Z16 is Cell Type Dependent

**Figure 12:**
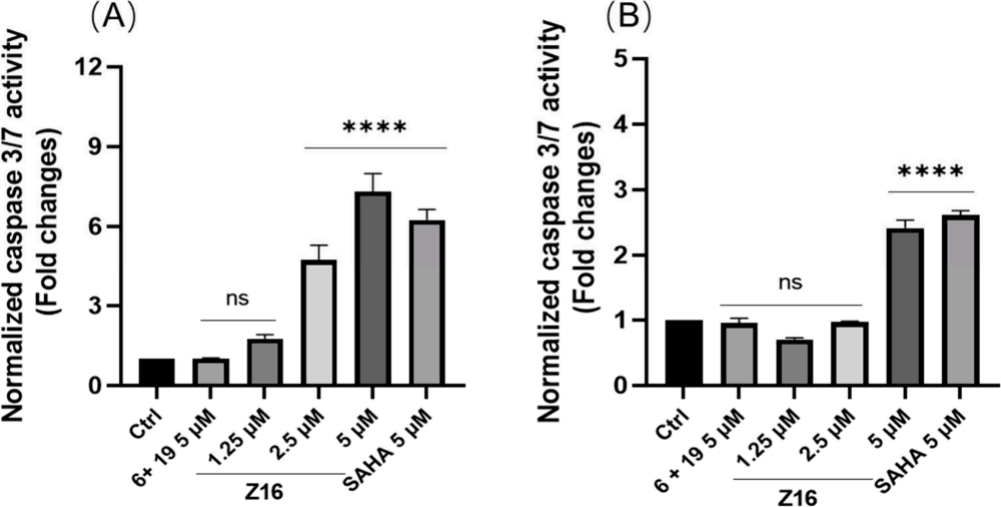
Caspase 3/7 activity was measured on Jurkat (A) and HCT116 (B) treated with the indicated concentrations of **Z16**, **6 + 19**, and SAHA for 48 h. Data were shown as mean ± SEM of three independent experiments. ns: not significant, *P < 0.05, **P < 0.01, ***P < 0.001, and ****P < 0.0001 vs DMSO-treated group, one-way analysis of variance (ANOVA).

**Figure 13:**
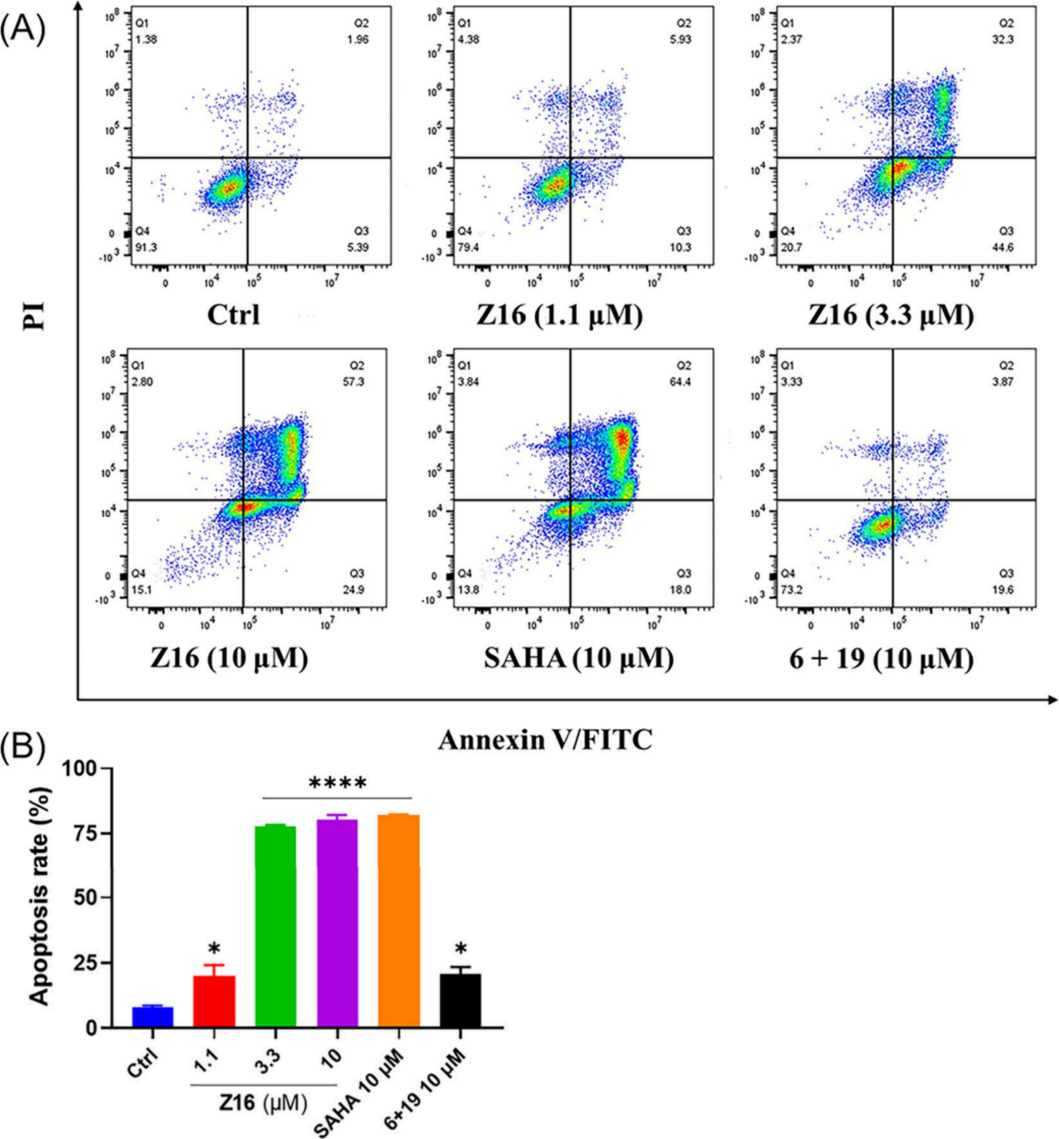
HCT116 cells treated with the indicated concentrations of **Z16**, **6 + 19**, and SAHA for 48 h. (A) Apoptosis was analyzed using flow cytometry. (B) Apoptosis rates were shown as mean ± SEM of three independent experiments. ns: not significant, *P < 0.05, **P < 0.01, ***P < 0.001, and ****P < 0.0001 vs DMSO-treated group, one-way analysis of variance (ANOVA).

After having observed that **Z16** showed similar substrate acetylation and antiproliferative activities as pan-HDAC inhibitor SAHA, we were interested to study the antiproliferative mechanisms of **Z16** in comparison to SAHA. First, the effect of **Z16** on induction of apoptosis was explored in Jurkat and HCT116 cell lines. **Z16** significantly increased the caspase 3/7 activity in a dose-dependent manner. Notably, 5 μM of **Z16** and SAHA has similar ability to increase the caspase 3/7 activity in both Jurkat and HCT116 cell lines, while 5 μM of HDAC8 inhibitor **6** plus CRBN ligand **19** did not demonstrate significant impact on caspase 3/7 activity (Figure 12A and 12B). Moreover, the pro-apoptotic potency of **Z16** was further evaluated on Jurkat and HCT116 cell lines by flow cytometry. However, neither **Z16** nor SAHA could effectively induce apoptosis in Jurkat cells after 24 and 48 h treatment (Figure S9A and S9B in the Supporting Information), whereas **Z16** significantly induced apoptosis in dose-dependent manner in HCT116 cells after 48 h treatment (Figure 13). Notably, 3.3 μM of **Z16** induced over 70% apoptosis, while 10 μM of **6** plus **19** induced just over 20% apoptosis in HCT116 cells (Figure 13). Interestingly, in 2001, Ruefli et al. reported that HDAC inhibitor SAHA initiates cell death by inducing mitochondria-mediated death pathways characterized by the production of reactive oxygen species (ROS), which is independent of the activation of key caspases, in T-cell leukemia CEM cells.^62^ Although **Z16** and SAHA could increase the caspase 3/7 activity in Jurkat cells (Figure 12A), the low apoptosis rate detected by flow cytometry in Jurkat cells (Figure S9 in the Supporting Information) indicates that **Z16** and SAHA affect cell viability via a nonapoptotic mechanism at low micromolar concentrations in Jurkat cells.

In order to further unravel the antiproliferative mechanisms of **Z16** in these four cell lines, they were pretreated with 20 μM apoptosis inhibitor Z-VAD-FMK,^63^ followed by treatment with **Z16**. The apoptosis inhibitor Z-VAD-FMK did not affect the antiproliferative activity against Jurkat cells upon **Z16** treatment, while Z-VAD-FMK nearly completely diminished the antiproliferative activity against THP-1 cells and partly attenuated the antiproliferative activities against both HCT116 and A549 cells upon **Z16** treatment (Table 5 and Figure S10A in the Supporting Information). However, 10 μM of necroptosis inhibitor necrostatin-1 (Nec-1)^64^ did not show significant impact on the antiproliferative activities against Jurkat, HCT116 and A549 cells upon **Z16** treatment (Table 5 and Figure S10A in the Supporting Information). Based on these results, we conclude that **Z16** did not induce apoptosis or necroptosis in Jurkat cells, even though it activated caspase 3/7 (Figure 12A). In contrast, the antiproliferative activity of **Z16** in THP-1 cells is likely due to apoptosis because it can be inhibited by an apoptosis inhibitor. Moreover, treatment with the apoptosis inhibitor demonstrates that the antiproliferative activities against HCT116 and A549 cells upon **Z16** treatment can be partly ascribed to apoptosis, but not necroptosis.

**Table 5:** Antiproliferative Activity of **Z16** in the Absence and Presence of 20 μM Z-VAD-FMK, 10 μM of Nec-1, 1 μM of Fer-1, and 1 μM or 15 μM of CQ^a^

	IC_50_ (μM)			
	Hematological malignancies		Solid tumors	
ID	Jurkat	THP-1	HCT116	A549
**Z16**	1.7 ± 0.2	1.8 ± 0.3	1.4 ± 0.1	7.7 ± 0.8
**Z16 + Z-VAD-FMK**	1.5 ± 0.2	>33.3	3.8 ± 0.6	17.5 ± 2.5
**Z16 + Nec-1**	2.0 ± 0.3	–	1.8 ± 0.3	7.5 ± 1.0
**Z16 + Fer-1**	2.8 ± 0.5	–	1.7 ± 0.2	–
**Z16 + CQ**	2.5 ± 0.5	–	1.5 ± 0.1	–

a Cells were pretreated with 20 μM of caspase inhibitor Z-VAD-FMK, 10 μM of necroptosis inhibitor necrostatin-1 (Nec-1),1 μM of ferroptosis inhibitor ferrostain-1 (Fer-1), 1 μM and 15 μM of autophagy inhibitor chloroquine (CQ) for Jurkat and HCT116, respectively, for 1 h, followed by treatment of **Z16** for 72 h. Data are shown as mean ± SEM of three independent experiments.

**Figure 14:**
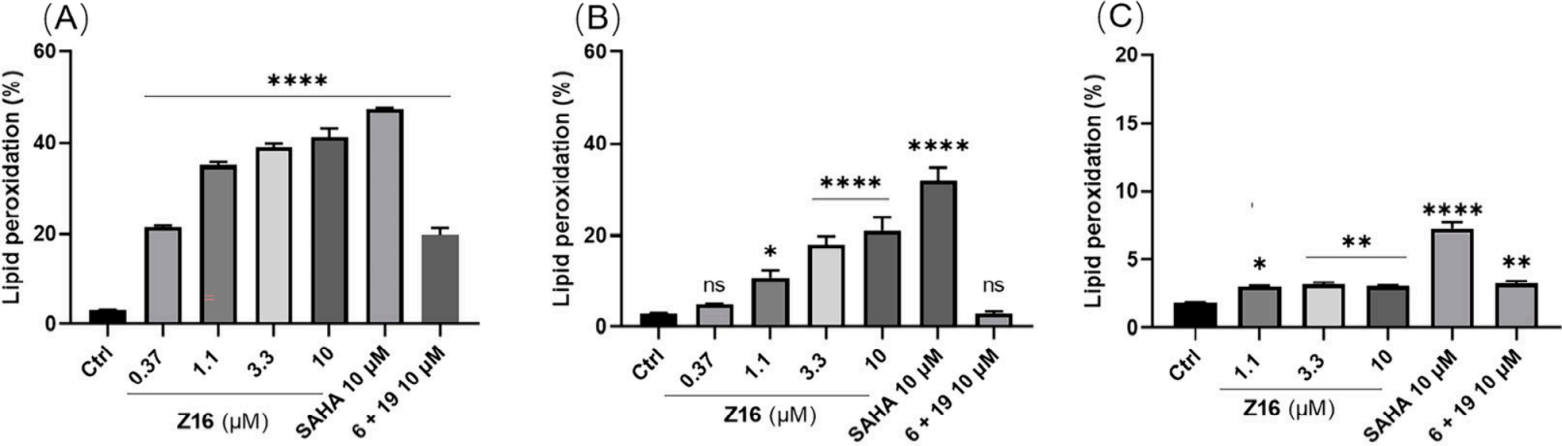
Cells were treated with indicated concentrations of **Z16**, SAHA, and **6 + 19** for 24 h. The lipid peroxidation level in Jurkat (A), HCT116 (B), and THP-1 (C) were detected by BODIPY 581/591 C11 staining determined by flow cytometry. Data are shown as mean ± SEM of two independent experiments. ns: not significant, *P < 0.05, **P < 0.01, ***P < 0.001, and ****P < 0.0001 vs vehicle group, one-way analysis of variance (ANOVA).

**Figure 15:**
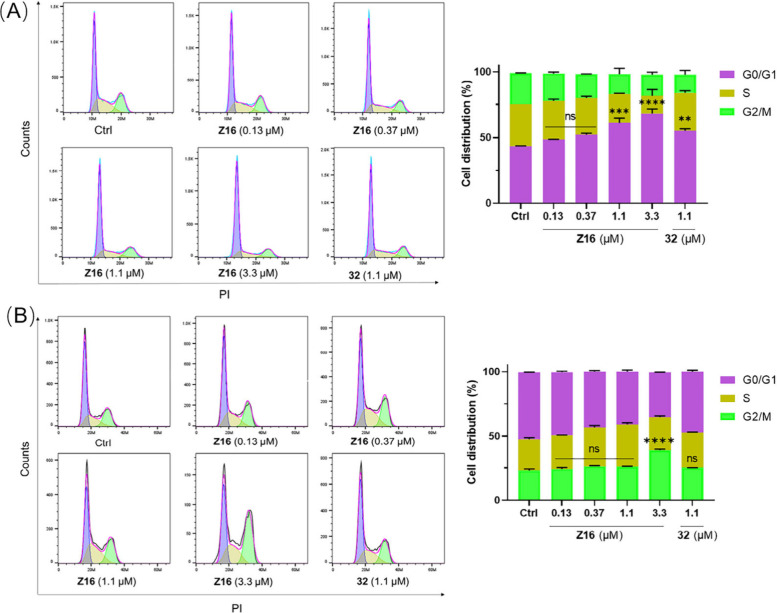
Induction of cell cycle arrest by **Z16** in Jurkat and HCT116 cells. Jurkat (A) and HCT116 (B) cells were treated with indicated concentrations of **Z16** and **32** for 48 h. Cell distributions were analyzed using flow cytometry and shown as mean ± SEM of two independent experiments. ns: not significant, *P < 0.05, **P < 0.01, ***P < 0.001, and ****P < 0.0001 vs DMSO-treated group, two-way analysis of variance (ANOVA).

In addition to apoptosis and necroptosis, ferroptosis is another regulated cell death, which is characterized by the accumulation of lipid ROS.^65^ Next, we explored if **Z16** induced ferroptosis in Jurkat, THP-1 and HCT116 cells. Toward this aim, the lipid peroxidation levels in Jurkat cells were assessed by stained with the BODIPY 581/591 C11 dye. **Z16** induced lipid peroxidation in a dose-dependent manner in both Jurkat cells (Figure 14A and S11A in the Supporting Information) and HCT116 cells at low micromolar concentrations (Figure 14B and S11B in the Supporting Information), but not in THP-1 cells (Figure 14C and S11C in the Supporting Information). It is worth noting that 10 μM of **Z16** induced over 40% lipid peroxidation in Jurkat cells, but to a lower extent in HCT116 cells after 24 h treatment, which is comparable with SAHA treatment. This indicates that lipid peroxidation might play an important role in antiproliferation against Jurkat cells. Interestingly, **Z16** showed a sharp decrease of lipid peroxidation in Jurkat cells after 48 h treatment (Figure S11D in the Supporting Information). Additionally, the ferroptosis inhibitor Ferrostatin-1 (Fer-1)^66^ could slightly attenuate the antiproliferative activity against Jurkat cells, but failed to attenuate the antiproliferative activity against HCT116 cells (Table 5). In addition, we also explored if the autophagy is involved in the antiproliferative mechanisms of **Z16** in Jurkat and HCT116 cells. Jurkat cells were pretreated with 1 μM of autophagy inhibitor chloroquine (CQ),^67^ which was not toxic to Jurkat cells, followed by treatment with **Z16**, while HCT116 cells were pretreated with 15 μM of autophagy inhibitor CQ, which was not toxic to HCT116 cells, followed by treatment with **Z16**. However, pretreatment with CQ did not significantly affect the antiproliferative activities of **Z16** against both Jurkat and HCT116 cells (Table 5). It is worth noting that these four cell lines showed similar responses to cell death inhibitors and SAHA cotreatment as they responded to cell death inhibitors and **Z16** cotreatment (Figure S10B in Supporting Information). Next, flow cytometry was used to detect the cell death in Jurkat cells upon **Z16** treatment. As shown in Figure S12 in the Supporting Information, 5 μM of **Z16** could only induced 10% cell death in Jurkat cells and various cell death inhibitors cannot rescue Jurkat cells death, which is consistent with results from the MTS test (Table 5). These results indicate that other mechanisms except cell death might be responsible for the antiproliferative activity of **Z16** against Jurkat cells. It has been reported that HDAC inhibitors can also induce cell cycle arrest in cancer cells.^8^ To investigate if **Z16** can induce cell cycle arrest in Jurkat and HCT116 cells, flow cytometry was used to analyze cell cycle arrest in Jurkat cells upon **Z16** and **32** treatment. **Z16** dose-dependently induced cell cycle arrest at the G0/G1 phase in Jurkat cells and G2/M phase in HCT116, respectively (Figure 15). The proportion of Jurkat cells at G0/G1 phase is 43.5% for the control group, which increases to 48.4%, 52.2%, 61%, and 68.2% upon treatment with 0.13 μM, 0.37 μM, 1.1 μM, and 3.3 μM of **Z16**, respectively, while the proportion of HCT116 cells at G2/M phase is 23.0% for the control group (Figure 15A), which increases to 24.1%, 26.4%, 26.2%, and 38.8% upon treatment with 0.13 μM, 0.37 μM, 1.1 μM, and 3.3 μM of **Z16**, respectively (Figure 15B). These results indicate that **Z16** induces inhibition of cell cycle progression in Jurkat and HCT116 cells, which can explain the observed inhibition of cell proliferation. Based on these results above, we can conclude that **Z16** induces lipid peroxidation and cell cycle arrest in Jurkat cells, lipid peroxidation, cell cycle arrest and apoptosis in HCT116 cells, and apoptosis in THP-1 cells. However, the contributions of lipid peroxidation to antiproliferative activities warrant further investigation. Taken together, this demonstrates that **Z16** shows different antiproliferative mechanisms in different cell lines.

## Conclusion

Based on investigation of the structure–activity relationship (SAR), a potent and selective HDAC8 PROTAC **Z16** was identified and characterized with low nanomolar level DC_50_ values in various cell lines, whereas no visible degradation of HDAC 1,2,3,4,6,7 and 11 as well as the CRBN neo-substrates IKZF1, IKZF3, and GSPT1 occurred. **Z16** induced SMC3 hyperacetylation at low concentrations and histone hyperacetylation at high concentrations, which can be explained by HDAC8 degradation and off-target HDAC inhibition, respectively. Importantly, **Z16** potently inhibited proliferation of various cancer cells at low micromolar concentrations via different antiproliferative mechanisms, which can, to a large extent, be attributed to off-target HDAC inhibition instead of HDAC8 degradation. Interestingly, the various antiproliferative mechanisms proved to be highly cell type dependent. This sets the stage for further exploration of the biological roles of HDACs and their connection to different antiproliferative mechanisms in different cell types. In conclusion, we report a potent and selective HDAC8 PROTAC, which can be used as a chemical tool to further investigate the biological roles of HDAC8.

## Experimental Section

### Chemistry

Unless otherwise noted, the chemical reagents and solvents were purchased from commercial sources, such as Sigma-Aldrich, BLD pharm, Fluorochem as well as Acros, and were used without further purification. All reactions were monitored by thin-layer chromatography (TLC) on 0.25 mm silica gel plates (60GF-254). UV light, iodine stain, and potassium permanganate staining were used to visualize the spots. ^1^H and ^13^C NMR spectra were recorded on a Bruker DRX spectrometer at 500 or 600 MHz, with δ given in parts per million (ppm) and *J* in hertz (Hz) and using TMS an internal standard. Multiplicity of ^1^H NMR signals was reported as singlet (s), doublet (d), triplet (t), quartet (q), and multiplet (m). High-resolution mass spectra (HRMS) were recorded using Fourier Transform Mass Spectrometry (FTMS) and Orbitrap XL Hybrid Ion Trap-Orbitrap Mass Spectrometer. Silica gel was used for column chromatography purification. C18 reverse-phase high performance liquid chromatography (HPLC) analysis was performed to determine the purity of target compounds. All the final products have at least 95% purity except compounds **Z3** and **Z7**, which both have higher than 93% purity.

#### 4-(((*tert*-Butoxycarbonyl)amino)methyl)benzoic acid (**9**)

A solution of compound **8** (1.0 g, 6.6 mmol) and 8 mL 1 M NaOH (aq) in 20 mL THF/H_2_O (V:V = 1:1) was cooled to 0 °C. Then, (Boc)_2_O (1.5 g, 6.8 mmol) solubilized in 10 mL THF was added to the solution dropwise. The resulting solution was stirred at room temperature overnight. Then the organic solvents were removed under reduced pressure. The pH value of the resulting residue was adjusted to 4 by the addition of 1 M HCl (aq) dropwise at 0 °C. The desired product was collected by filtration and dried to give 1.6 g of compound **9** as a white solid in a yield of 94%.^1^H NMR (500 MHz, DMSO-*d*_6_) δ 12.90 (s, 1H), 7.90 (d, *J* = 7.9 Hz, 2H), 7.49 (t, *J* = 6.2 Hz, 1H), 7.35 (d, *J* = 8.0 Hz, 2H), 4.19 (d, *J* = 6.2 Hz, 2H), 1.40 (s, 7H).

#### *tert*-Butyl (4-(hydrazinecarbonyl)benzyl)carbamate (**10**)

CDI (carbonyldiimidazole, 0.51 g, 3.2 mmol) was added to a stirred mixture of compound **9** (0.75 g, 3.0 mmol) in 10 mL of THF and the resulting solution was stirred at room temperature for additional 30 min. Then, hydrazine monohydrate (1.5 mL, 30.0 mmol) was added to the reaction solution dropwise. The resulting solution was allowed to stirred overnight. Afterward, the solution was evaporated and 50 mL water was added. The formed precipitate was collected and dried to give 0.50 g of compound **10** as a white solid in a yield of 63%. ^1^H NMR (500 MHz, DMSO-*d*_6_) δ 9.73 (s, 1H), 7.77 (d, *J* = 7.9 Hz, 2H), 7.46 (t, *J* = 6.3 Hz, 1H), 7.29 (d, *J* = 7.9 Hz, 2H), 4.46 (s, 2H), 4.16 (d, *J* = 6.2 Hz, 2H), 1.40 (s, 9H).

#### *tert*-Butyl (4-(2-hexylhydrazine-1-carbonyl)benzyl)carbamate (**11**)

A solution of compound **10** (0.40 g, 1.5 mmol) and hexanal (195 μL, 1.6 mmol) in 5 mL of MeOH was stirred at room temperature for 1 h. Then, NaBH_4_ (115 mg, 3.0 mmol) was added to the reaction mixture in two portions. The resulting solution was evaporated and diluted with EtOAc, washed with water and brine. The organic layer was concentrated and triturated with Pentane/EtOAc to give 0.30 g of compound **11** as a white solid in a yield of 57%. ^1^H NMR (500 MHz, DMSO-*d*_6_) δ 9.97 (d, *J* = 6.6 Hz, 1H), 7.77 (d, *J* = 8.1 Hz, 2H), 7.47 (t, *J* = 6.3 Hz, 1H), 7.30 (d, *J* = 7.9 Hz, 2H), 5.06 (q, *J* = 6.1 Hz, 1H), 4.16 (d, *J* = 6.3 Hz, 2H), 2.77 (q, *J* = 6.7 Hz, 2H), 1.48–1.24 (m, 17H), 0.94–0.80 (m, 3H).

#### 4-(Aminomethyl)-*N*′-hexylbenzohydrazide hydrochloride acid (**12**)

To a solution of compound **11** (0.25 g, 0.71 mmol) in 3 mL of DCM was added 4 N HCl in dioxane (1.8 mL, 7.1 mmol) dropwise at room temperature. The resulting solution was stirred at room temperature for 1 h. The desired product was collected by filtration to give 0.20 g of compound **12** as a white solid in a yield of 87%. ^1^H NMR (500 MHz, DMSO-*d*_6_) δ 11.95 (s, 1H), 8.51 (s, 3H), 8.00 (d, *J* = 8.0 Hz, 2H), 7.65 (d, *J* = 8.0 Hz, 2H), 4.12 (q, *J* = 5.9 Hz, 2H), 3.17 (t, *J* = 7.8 Hz, 2H), 1.64 (q, *J* = 7.8 Hz, 2H), 1.39–1.23 (m, 6H), 0.87 (t, *J* = 6.7 Hz, 3H).

### Method A for Amide Formation

A solution of respective carboxylic acids (0.15 mmol, 1.0 equiv), DIPEA (4.0 equiv) and HATU (1.2 equiv) in 3 mL of DMF was cooled to 0 °C. Then, amine (1.0 equiv) was added to the solution. The reaction solution was warmed to room temperature and stirred overnight. Afterward, the solution was diluted with EtOAc, washed with water and brine. The organic layer was concentrated and further purified by column chromatography (DCM/MeOH = 100:1–10:1).

### Method B for Amide Formation

A solution of respective carboxylic acids (0.11 mmol, 1.0 equiv), EDCI (1.2 equiv) and HOBT (1.2 equiv) in 2 mL of DMF was cooled to 0 °C. Then, TEA (3.0 equiv) was added to the solution, followed by the addition of compound **12** (1.1 equiv). The reaction solution was warmed to room temperature and stirred overnight. Afterward, the solution was diluted with EtOAc, washed with water and brine. The organic layer was concentrated and further purified by column chromatography (DCM/MeOH = 100:1–10:1).

#### N-(4-(2-Hexylhydrazine-1-carbonyl)benzyl)acetamide (6)

Using the method A for amide formation, compound **12** gave 40 mg of **6** as a white solid in a yield of 44%. ^1^H NMR (500 MHz, DMSO-*d*_6_) δ 9.98 (s, 1H), 8.40 (t, *J* = 6.1 Hz, 1H), 7.77 (d, *J* = 8.0 Hz, 2H), 7.31 (d, *J* = 8.0 Hz, 2H), 5.07 (s, 1H), 4.29 (d, *J* = 6.0 Hz, 2H), 2.77 (t, *J* = 7.1 Hz, 2H), 1.89 (s, 3H), 1.45 (p, *J* = 7.1 Hz, 2H), 1.38–1.22 (m, 6H), 0.87 (t, *J* = 6.8 Hz, 3H). HRMS, calculated 292.2020 for C_16_H_26_N_3_O_2_ [M + H]^+^, found 292.2017. Purity: 95.9%.

Intermediates **13a**–**13h** were synthesized according to previously reported methods.^59^

#### 4-((2-(2,6-Dioxopiperidin-3-yl)-1,3-dioxoisoindolin-4-yl)amino)-N-(4-(2-hexylhydrazine-1-carbonyl)benzyl)butanamide (**Z1**)

Using the method B for amide formation, compound **13a** and compound **12** gave 35 mg of **Z1** as a yellow solid in a yield of 54%. ^1^H NMR (500 MHz, DMSO-*d*_6_) δ 11.12 (s, 1H), 9.98 (s, 1H), 8.46 (t, *J* = 6.2 Hz, 1H), 7.76 (d, *J* = 7.9 Hz, 2H), 7.58 (t, *J* = 7.8 Hz, 1H), 7.31 (d, *J* = 8.0 Hz, 2H), 7.11 (d, *J* = 8.7 Hz, 1H), 7.03 (d, *J* = 7.1 Hz, 1H), 6.68–6.63 (m, 1H), 5.06 (dd, *J* = 12.7, 5.6 Hz, 2H), 4.31 (d, *J* = 6.1 Hz, 2H), 3.32–3.30 (m, 2H), 2.94–2.84 (m, 1H), 2.76 (s, 2H), 2.64–2.52 (m, 2H), 2.26 (d, *J* = 7.3 Hz, 2H), 2.05–2.00 (m, 1H), 1.85–1.81 (m, 2H), 1.44 (q, *J* = 7.3 Hz, 2H), 1.36–1.24 (m, 6H), 0.91–0.85 (m, 3H). ^13^C NMR (126 MHz, DMSO-*d*_6_) δ 173.3, 172.2, 170.6, 169.3, 167.8, 165.5, 146.8, 143.5, 136.8, 136.6, 132.7, 132.1, 127.6, 127.4, 117.6, 110.9, 109.6, 51.7, 49.0, 42.3, 42.0, 40.6, 32.9, 31.7, 31.5, 28.1, 26.8, 22.6, 14.4. HRMS, calculated 591.2926 for C_31_H_39_N_6_O_6_ [M + H]^+^, found 591.2923. Purity: 95.8%.

#### 5-((2-(2,6-Dioxopiperidin-3-yl)-1,3-dioxoisoindolin-4-yl)amino)-N-(4-(2-hexylhydrazine-1-carbonyl)benzyl)pentanamide (**Z2**)

Using the method B for amide formation, compound **13b** and compound **12** gave 26 mg of **Z2** as a yellow solid in a yield of 32%. ^1^H NMR (500 MHz, DMSO-*d*_6_) δ 11.11 (s, 1H), 9.97 (s, 1H), 8.40 (t, *J* = 6.0 Hz, 1H), 7.76 (d, *J* = 8.0 Hz, 2H), 7.58 (t, *J* = 7.8 Hz, 1H), 7.30 (d, *J* = 7.9 Hz, 2H), 7.10 (d, *J* = 8.6 Hz, 1H), 7.02 (d, *J* = 7.0 Hz, 1H), 6.59 (t, *J* = 6.0 Hz, 1H), 5.10–5.02 (m, 2H), 4.30 (d, *J* = 6.1 Hz, 2H), 3.33–3.30 (m, 2H), 2.96–2.83 (m, 1H), 2.77 (t, *J* = 7.1 Hz, 2H), 2.67–2.54 (m, 2H), 2.21 (t, *J* = 7.0 Hz, 2H), 2.08–2.00 (m, 1H), 1.64–1.56 (m, 4H), 1.49–1.42 (m, 2H), 1.37–1.22 (m, 6H), 0.87 (t, *J* = 6.7 Hz, 3H). ^13^C NMR (126 MHz, DMSO-*d*_6_) δ 173.3, 172.5, 170.6, 169.4, 167.8, 165.5, 146.8, 143.5, 136.8, 136.7, 132.7, 132.1, 127.4, 117.6, 110.8, 109.5, 51.7, 49.0, 42.2, 42.0, 40.6, 35.4, 31.7, 31.5, 28.1, 26.8, 23.1, 22.6, 14.4. HRMS, calculated 605.3083 for C_32_H_41_N_6_O_6_ [M + H]^+^, found 605.3079. Purity: 96.0%.

#### 6-((2-(2,6-Dioxopiperidin-3-yl)-1,3-dioxoisoindolin-4-yl)amino)-N-(4-(2-hexylhydrazine-1-carbonyl)benzyl)hexanamide (**Z3**)

Using the method B for amide formation, compound **13c** and compound **12** gave 10 mg of **Z3** as a yellow solid in a yield of 10%. ^1^H NMR (500 MHz, DMSO-*d*_6_) δ 11.11 (s, 1H), 8.41 (t, *J* = 6.0 Hz, 1H), 7.83–7.77 (m, 2H), 7.58 (dd, *J* = 9.7, 6.1 Hz, 1H), 7.39–7.25 (m, 2H), 7.09 (dd, *J* = 8.6, 4.3 Hz, 1H), 7.03 (dd, *J* = 7.3, 3.6 Hz, 1H), 6.55 (t, *J* = 6.3 Hz, 1H), 5.10–5.02 (m, 2H), 4.35–4.29 (m, 2H), 3.33–3.25 (m, 2H), 3.00–2.83 (m, 3H), 2.63–2.53 (m, 2H), 2.18 (t, *J* = 7.3 Hz, 2H), 2.06–2.01 (m, 1H), 1.63–1.48 (m, 6H), 1.39–1.29 (m, 4H), 1.28–1.24 (m, 4H), 0.91–0.83 (m, 3H). ^13^C NMR (151 MHz, DMSO-*d*_6_) δ 173.3, 172.6, 170.6, 170.5, 169.4, 167.8, 165.7, 146.9, 136.8, 132.7, 127.8, 127.5, 117.6, 110.9, 109.5, 51.1, 49.0, 42.2, 42.2, 40.5, 35.7, 31.5, 28.9, 26.4, 26.4, 25.5, 22.6, 22.5, 14.4. HRMS, calculated 619.3239 for C_33_H_43_N_6_O_6_ [M + H]^+^, found 619.3238. Purity: 93.8%.

#### 7-((2-(2,6-Dioxopiperidin-3-yl)-1,3-dioxoisoindolin-4-yl)amino)-N-(4-(2-hexylhydrazine-1-carbonyl)benzyl)heptanamide (**Z4**)

Using the method A for amide formation, compound **13d** and compound **12** gave 40 mg of **Z4** as a yellow solid in a yield of 42%. ^1^H NMR (500 MHz, DMSO-*d*_6_) δ 11.11 (s, 1H), 9.97 (s, 1H), 8.36 (t, *J* = 6.1 Hz, 1H), 7.77 (dd, *J* = 8.0, 1.3 Hz, 2H), 7.58 (t, *J* = 7.8 Hz, 1H), 7.30 (d, *J* = 8.0 Hz, 2H), 7.09 (d, *J* = 8.6 Hz, 1H), 7.03 (d, *J* = 7.0 Hz, 1H), 6.55 (t, *J* = 6.0 Hz, 1H), 5.06 (dd, *J* = 12.7, 5.5 Hz, 2H), 4.30 (d, *J* = 6.1 Hz, 2H), 3.31–3.27 (m, 2H), 2.94–2.84 (m, 1H), 2.76 (t, *J* = 7.2 Hz, 2H), 2.63–2.51 (m, 2H), 2.16 (t, *J* = 7.4 Hz, 2H), 2.07–1.99 (m, 1H), 1.60–1.52 (m, 7.2 Hz, 4H), 1.47–1.42 (m, 2H), 1.39–1.17 (m, 10H), 0.87 (t, *J* = 6.7 Hz, 3H). ^13^C NMR (126 MHz, DMSO-*d*_6_) δ 173.3, 172.6, 170.6, 169.4, 167.8, 165.5, 146.9, 143.6, 136.8, 136.7, 132.7, 132.1, 127.6, 127.4, 117.6, 110.8, 109.5, 51.7, 49.0, 42.3, 42.2, 40.6, 35.7, 31.7, 31.4, 29.0, 28.9, 28.1, 26.8, 26.6, 25.7, 22.6, 14.4. HRMS, calculated 632.3396 for C_34_H_44_N_6_O_6_ [M + H]^+^, found 632.3394. Purity: 95.4%.

#### 8-((2-(2,6-Dioxopiperidin-3-yl)-1,3-dioxoisoindolin-4-yl)amino)-N-(4-(2-Hexylhydrazine-1-carbonyl)benzyl)octanamide (**Z5**)

Using the method A for amide formation, compound **13e** and compound **12** gave 30 mg of **Z5** as a yellow solid in a yield of 30%. ^1^H NMR (500 MHz, DMSO-*d*_6_) δ 11.11 (s, 1H), 9.98 (d, *J* = 5.9 Hz, 1H), 8.36 (t, *J* = 6.0 Hz, 1H), 7.77 (d, *J* = 7.9 Hz, 2H), 7.60–7.55 (m, 1H), 7.30 (d, *J* = 8.0 Hz, 2H), 7.10 (d, *J* = 8.6 Hz, 1H), 7.02 (d, *J* = 7.0 Hz, 1H), 6.54 (t, *J* = 5.9 Hz, 1H), 5.06 (dd, *J* = 12.8, 5.4 Hz, 2H), 4.29 (d, *J* = 6.0 Hz, 2H), 3.29 (q, *J* = 6.7 Hz, 2H), 2.89 (ddd, *J* = 17.5, 13.5, 5.4 Hz, 1H), 2.76 (q, *J* = 6.6 Hz, 2H), 2.65–2.53 (m, 2H), 2.15 (t, *J* = 7.4 Hz, 2H), 2.04–2.00 (m, 1H), 1.56–1.49 (m, 4H), 1.46–1.41 (m, 2H), 1.36–1.24 (m, 12H), 0.90–0.81 (m, 3H). ^13^C NMR (126 MHz, DMSO-*d*_6_) δ 173.3, 172.7, 170.6, 169.4, 167.8, 165.5, 146.9, 143.6, 136.8, 136.7, 132.7, 132.1, 127.6, 127.4, 117.6, 110.8, 109.5, 51.7, 49.0, 42.3, 42.1, 40.6, 35.8, 31.7, 31.4, 29.1, 29.1, 29.0, 28.1, 26.8, 26.7, 25.7, 22.6, 14.4. HRMS, calculated 647.3552 for C_35_H_47_N_6_O_6_ [M + H]^+^, found 647.3552. Purity: 95.5%.

#### 10-((2-(2,6-Dioxopiperidin-3-yl)-1,3-dioxoisoindolin-4-yl)amino)-N-(4-(2-hexylhydrazine-1-carbonyl)benzyl)decanamide (**Z6**)

Using the method A for amide formation, compound **13f** and compound **12** gave 24 mg of **Z6** as a yellow solid in a yield of 26%. ^1^H NMR (500 MHz, DMSO-*d*_6_) δ 11.11 (s, 1H), 9.98 (s, 1H), 8.35 (t, *J* = 6.0 Hz, 1H), 7.77 (d, *J* = 8.0 Hz, 2H), 7.58 (t, *J* = 7.8 Hz, 1H), 7.30 (d, *J* = 8.0 Hz, 2H), 7.09 (d, *J* = 8.6 Hz, 1H), 7.02 (d, *J* = 7.0 Hz, 1H), 6.54 (t, *J* = 6.0 Hz, 1H), 5.06 (dd, *J* = 12.7, 5.5 Hz, 2H), 4.29 (d, *J* = 6.0 Hz, 2H), 3.29 (q, *J* = 6.7 Hz, 2H), 2.90–2.85 (m, 1H), 2.77 (d, *J* = 7.8 Hz, 2H), 2.63–2.53 (m, 2H), 2.14 (t, *J* = 7.4 Hz, 2H), 2.08–2.00 (m, 1H), 1.59–1.56 (m, 2H), 1.55–1.49 (m, 2H), 1.47–1.41 (m, 2H), 1.38–1.23 (m, 16H), 0.87 (t, *J* = 6.7 Hz, 3H). ^13^C NMR (126 MHz, DMSO-*d*_6_) δ 173.3, 172.7, 170.6, 169.4, 167.8, 165.5, 146.9, 143.6, 136.8, 136.7, 132.6, 132.1, 127.6, 127.4, 117.6, 110.8, 109.4, 51.7, 49.0, 42.3, 42.1, 40.6, 35.8, 31.7, 31.4, 29.4, 29.2, 28.1, 26.8, 25.7, 22.6, 14.4. HRMS, calculated 675.3865 for C_37_H_51_N_6_O_6_ [M + H]^+^, found 675.3866. Purity: 95.8%.

#### 12-((2-(2,6-Dioxopiperidin-3-yl)-1,3-dioxoisoindolin-4-yl)amino)-N-(4-(2-Hexylhydrazine-1-carbonyl)benzyl)dodecanamide (**Z7**)

Using the method A for amide formation, compound **13g** and compound **12** gave 20 mg of **Z7** as a yellow solid in a yield of 17%. ^1^H NMR (500 MHz, DMSO-*d*_6_) δ 11.11 (s, 1H), 9.98 (d, *J* = 5.9 Hz, 1H), 8.35 (t, *J* = 6.1 Hz, 1H), 7.77 (d, *J* = 7.9 Hz, 2H), 7.58 (t, *J* = 7.8 Hz, 1H), 7.30 (d, *J* = 8.0 Hz, 2H), 7.09 (d, *J* = 8.6 Hz, 1H), 7.02 (d, *J* = 7.0 Hz, 1H), 6.54 (t, *J* = 5.9 Hz, 1H), 5.06 (dd, *J* = 12.8, 5.4 Hz, 2H), 4.29 (d, *J* = 6.0 Hz, 2H), 3.29 (q, *J* = 6.7 Hz, 2H), 2.95–2.84 (m, 1H), 2.76 (q, *J* = 6.4, 5.8 Hz, 2H), 2.64–2.51 (m, 2H), 2.14 (t, *J* = 7.4 Hz, 2H), 2.07–2.01 (m, 1H), 1.59–1.56 (m, 2H), 1.55–1.46 (m, 2H), 1.48–1.39 (m, 2H), 1.36–1.22 (m, 20H), 0.87 (t, *J* = 6.9 Hz, 3H). ^13^C NMR (126 MHz, DMSO-*d*_6_) δ 173.3, 172.7, 170.6, 169.4, 167.8, 165.5, 146.9, 143.6, 136.7, 136.6, 132.6, 132.1, 127.5, 127.4, 117.6, 110.8, 109.4, 51.7, 49.0, 42.3, 42.1, 40.6, 35.8, 31.7, 31.4, 29.5, 29.4, 29.2, 29.1, 28.0, 26.8, 25.8, 22.6, 14.4. HRMS, calculated 703.4178 for C_39_H_55_N_6_O_6_ [M + H]^+^, found 703.4178. Purity: 93.8%.

Intermediates **14a**–**14f** were synthesized according to previously reported methods.^59^

#### N^1^-(4-(2-Hexylhydrazine-1-carbonyl)benzyl)-N^4^-((S)-1-((2*S*,4*R*)-4-hydroxy-2-((4-(4-methylthiazol-5-yl)benzyl)carbamoyl)pyrrolidin-1-yl)-3,3-dimethyl-1-oxobutan-2-yl)succinimide (**Z8**)

Using the method A for amide formation, compound **14a** and compound **12** gave 26 mg of **Z8** as a white solid in a yield of 23%. ^1^H NMR (500 MHz, DMSO-*d*_6_) δ 9.00 (d, *J* = 1.8 Hz, 1H), 8.59 (t, *J* = 6.4 Hz, 1H), 8.42 (t, *J* = 10.9 Hz, 1H), 7.96 (d, *J* = 9.1 Hz, 1H), 7.84–7.73 (m, 2H), 7.44–7.38 (m, 4H), 7.37–7.30 (m, 2H), 5.15 (d, *J* = 3.4 Hz, 1H), 4.55 (d, *J* = 9.2 Hz, 1H), 4.44 (dd, *J* = 14.8, 6.6 Hz, 2H), 4.38–4.27 (m, 3H), 4.23 (dd, *J* = 15.6, 5.6 Hz, 1H), 3.69–3.62 (m, 2H), 2.79–2.75 (m, 1H), 2.55 (d, *J* = 3.2 Hz, 1H), 2.45 (d, *J* = 1.9 Hz, 3H), 2.45–2.33 (m, 3H), 2.30–2.22 (m, 1H), 2.09–2.00 (m, 1H), 1.96–1.85 (m, 1H), 1.56–1.40 (m, 2H), 1.37–1.23 (m, 6H), 0.94–0.87 (m, 12H). ^13^C NMR (151 MHz, DMSO-*d*_6_) δ 172.4, 172.0, 172.0, 171.7, 170.0, 165.6, 151.9, 148.2, 143.4, 140.0, 132.1, 131.6, 130.1, 129.1, 127.9, 127.5, 127.4, 69.4, 59.2, 56.9, 56.79, 51.7, 42.2, 42.1, 40.5, 38.4, 35.8, 31.7, 28.1, 26.8, 26.8, 22.6, 16.4, 14.4. HRMS, calculated 762.4008 for C_40_H_56_N_7_O_6_S [M + H]^+^, found 762.4009. Purity: 96.6%.

#### N^1^-(4-(2-Hexylhydrazine-1-carbonyl)benzyl)-N^6^-((S)-1-((2*S*,4*R*)-4-hydroxy-2-((4-(4-methylthiazol-5-yl)benzyl)carbamoyl)pyrrolidin-1-yl)-3,3-dimethyl-1-oxobutan-2-yl)adipamide (**Z9**)

Using the method A for amide formation, compound **14b** and compound **12** gave 40 mg of **Z9** as a white solid in a yield of 34%. ^1^H NMR (600 MHz, DMSO-*d*_6_) δ 9.98 (s, 1H), 8.98 (s, 1H), 8.57 (t, *J* = 6.1 Hz, 1H), 8.35 (t, *J* = 6.0 Hz, 1H), 7.86 (d, *J* = 9.4 Hz, 1H), 7.78–7.73 (m, 2H), 7.42 (d, *J* = 8.3 Hz, 2H), 7.39 (d, *J* = 8.3 Hz, 2H), 7.31–7.28 (m, 2H), 5.15 (d, *J* = 3.5 Hz, 1H), 4.55 (d, *J* = 9.4 Hz, 1H), 4.46–4.40 (m, 2H), 4.38–4.34 (m, 2H), 4.33–4.27 (m, 2H), 4.22 (dd, *J* = 15.8, 5.5 Hz, 1H), 3.69–3.64 (m, 2H), 2.77 (t, *J* = 7.2 Hz, 2H), 2.45 (s, 3H), 2.31–2.23 (m, 1H), 2.17–2.14 (m, 3H), 2.06–2.02 (m, 1H), 1.93–1.89 (m, 1H), 1.54–1.43 (m, 6H), 1.37–1.21 (m, 6H), 0.94 (s, 9H), 0.86 (t, *J* = 6.9 Hz, 3H). ^13^C NMR (151 MHz, DMSO-*d*_6_) δ 172.6, 172.4, 170.2, 165.6, 151.9, 148.2, 143.6, 140.0, 132.1, 131.6, 130.1, 129.1, 127.9, 127.5, 127.4, 69.3, 59.2, 56.8, 56.8, 51.7, 42.2, 42.1, 40.5, 38.4, 35.7, 35.6, 35.2, 31.7, 28.0, 26.8, 26.8, 25.9, 25.7, 25.5, 22.5, 16.4, 14.4. HRMS, calculated 790.4321 for C_42_H_60_N_7_O_6_S [M + H]^+^, found 790.4316. Purity: 95.2%.

#### N^1^-(4-(2-Hexylhydrazine-1-carbonyl)benzyl)-N^8^-((S)-1-((2*S*,4*R*)-4-hydroxy-2-((4-(4-methylthiazol-5-yl)benzyl)carbamoyl)pyrrolidin-1-yl)-3,3-dimethyl-1-oxobutan-2-yl)octanediamide (**Z10**)

Using the method A for amide formation, compound **14c** and compound **12** gave 60 mg of **Z10** as a white solid in a yield of 49%. ^1^H NMR (600 MHz, DMSO-*d*_6_) δ 9.99 (s, 1H), 8.98 (s, 1H), 8.57 (t, *J* = 6.1 Hz, 1H), 8.35 (t, *J* = 6.0 Hz, 1H), 7.85 (d, *J* = 9.3 Hz, 1H), 7.76 (d, *J* = 8.3 Hz, 2H), 7.42 (d, *J* = 8.1 Hz, 2H), 7.39 (d, *J* = 8.3 Hz, 2H), 7.30 (d, *J* = 8.1 Hz, 2H), 5.15 (d, *J* = 3.5 Hz, 1H), 4.55 (d, *J* = 9.4 Hz, 1H), 4.45–4.42 (m, 2H), 4.38–4.34 (m, 1H), 4.29 (d, *J* = 6.0 Hz, 2H), 4.22 (dd, *J* = 15.8, 5.5 Hz, 1H), 3.70–3.63 (m, 2H), 2.77 (t, *J* = 7.1 Hz, 2H), 2.45 (s, 3H), 2.29–2.23 (m, 1H), 2.15–2.10 (m, 3H), 2.06–2.01 (m, 1H), 1.93–1.89 (m, 1H), 1.53–1.42 (m, 6H), 1.36–1.23 (m, 10H), 0.94 (s, 9H), 0.86 (t, *J* = 6.9 Hz, 3H). ^13^C NMR (151 MHz, DMSO-*d*_6_) δ 172.8, 172.6, 172.3, 170.2, 165.6, 151.9, 148.2, 143.6, 139.9, 132.1, 131.6, 130.1, 129.1, 127.9, 127.5, 127.4, 69.3, 59.2, 56.8, 56.8, 51.7, 42.2, 42.1, 40.5, 38.4, 35.8, 35.7, 35.3, 31.7, 28.9, 28.9, 28.0, 26.8, 26.8, 25.8, 25.7, 22.5, 16.4, 14.4. HRMS, calculated 818.4634 for C_44_H_64_N_7_O_6_S [M + H]^+^, found 818.4636. Purity: 95.3%.

#### N^1^-(4-(2-Hexylhydrazine-1-carbonyl)benzyl)-N^9^-((S)-1-((2*S*,4*R*)-4-hydroxy-2-((4-(4-methylthiazol-5-yl)benzyl)carbamoyl)pyrrolidin-1-yl)-3,3-dimethyl-1-oxobutan-2-yl)nonanediamide (**Z11**)

Using the method A for amide formation, compound **14d** and compound **12** gave 60 mg of **Z11** as a white solid in a yield of 48%. ^1^H NMR (600 MHz, DMSO-*d*_6_) δ 9.97 (s, 1H), 8.98 (s, 1H), 8.57 (t, *J* = 6.1 Hz, 1H), 8.35 (t, *J* = 6.0 Hz, 1H), 7.85 (d, *J* = 9.4 Hz, 1H), 7.79–7.74 (m, 2H), 7.42 (d, *J* = 8.2 Hz, 2H), 7.39 (d, *J* = 8.3 Hz, 2H), 7.29 (d, *J* = 8.1 Hz, 2H), 5.13 (d, *J* = 3.6 Hz, 1H), 4.55 (dd, *J* = 9.5, 1.7 Hz, 1H), 4.47–4.40 (m, 2H), 4.38–4.33 (m, 1H), 4.29 (d, *J* = 6.0 Hz, 2H), 4.22 (dd, *J* = 15.8, 5.5 Hz, 1H), 3.70–3.63 (m, 2H), 2.77 (t, *J* = 7.1 Hz, 2H), 2.45 (s, 3H), 2.30–2.23 (m, 1H), 2.16–2.09 (m, 3H), 2.07–2.01 (m, 1H), 1.9–1.89 (m, 1H), 1.55–1.41 (m, 6H), 1.36–1.21 (m, 12H), 0.94 (s, 9H), 0.89–0.83 (m, 3H). ^13^C NMR (151 MHz, DMSO-*d*_6_) δ 172.8, 172.6, 172.4, 170.2, 165.6, 151.9, 148.2, 143.6, 140.0, 132.1, 131.6, 130.1, 129.1, 127.9, 127.5, 127.4, 69.3, 59.2, 56.8, 56.7, 51.7, 42.2, 42.1, 40.5, 38.4, 35.8, 35.7, 35.3, 31.7, 29.1, 29.0, 29.0, 28.0, 26.8, 26.8, 25.9, 25.7, 22.5, 16.4, 14.4. HRMS, calculated 832.4790 for C_45_H_66_N_7_O_6_S [M + H]^+^, found 832.4790. Purity: 96.0%.

#### N^1^-(4-(2-Hexylhydrazine-1-carbonyl)benzyl)-N^10^-((S)-1-((2*S*,4*R*)-4-hydroxy-2-((4-(4-methylthiazol-5-yl)benzyl)carbamoyl)pyrrolidin-1-yl)-3,3-dimethyl-1-oxobutan-2-yl)decanediamide (**Z12**)

Using the method A for amide formation, compound **14e** and compound **12** gave 30 mg of **Z12** as a white solid in a yield of 33%. ^1^H NMR (600 MHz, DMSO-*d*_6_) δ 9.96 (d, *J* = 6.0 Hz, 1H), 8.99 (s, 1H), 8.56 (t, *J* = 6.1 Hz, 1H), 8.34 (t, *J* = 6.0 Hz, 1H), 7.84 (d, *J* = 9.3 Hz, 1H), 7.78–7.74 (m, 2H), 7.42 (d, *J* = 8.3 Hz, 2H), 7.41–7.37 (m, 2H), 7.29 (d, *J* = 8.2 Hz, 2H), 5.12 (d, *J* = 3.6 Hz, 1H), 5.08–5.01 (m, 1H), 4.55 (d, *J* = 9.4 Hz, 1H), 4.45–4.42 (m, 2H), 4.36 (s, 1H), 4.29 (d, *J* = 6.0 Hz, 2H), 4.22 (dd, *J* = 15.8, 5.5 Hz, 1H), 3.71–3.61 (m, 2H), 2.77 (q, *J* = 6.6 Hz, 2H), 2.45 (s, 3H), 2.30–2.23 (m, 1H), 2.15–2.09 (m, 3H), 2.06–2.01 (m, 1H), 1.93–1.89 (m, 1H), 1.53–1.42 (m, 6H), 1.37–1.19 (m, 14H), 0.94 (s, 9H), 0.89–0.83 (m, 3H). ^13^C NMR (151 MHz, DMSO-*d*_6_) δ 172.7, 172.6, 172.4, 170.2, 165.6, 151.9, 148.2, 143.6, 140.0, 132.0, 131.6, 130.1, 129.1, 127.9, 127.5, 127.4, 69.3, 59.2, 56.8, 56.7, 51.7, 42.2, 42.1, 40.5, 38.4, 35.8, 35.7, 35.3, 31.7, 29.2, 29.1, 29.1, 28.0, 26.8, 26.8, 25.9, 25.8, 22.5, 16.4, 14.4. HRMS, calculated 846.4947 for C_46_H_68_N_7_O_6_S [M + H]^+^, found 846.4947. Purity: 95.2%.

#### N^1^-(4-(2-Hexylhydrazine-1-carbonyl)benzyl)-N^12^-((S)-1-((2*S*,4*R*)-4-hydroxy-2-((4-(4-methylthiazol-5-yl)benzyl)carbamoyl)pyrrolidin-1-yl)-3,3-dimethyl-1-oxobutan-2-yl)dodecanediamide (**Z13**)

Using the method A for amide formation, compound **14f** and compound **12** gave 45 mg of **Z13** as a white solid in a yield of 48%. ^1^H NMR (600 MHz, DMSO-*d*_6_) δ 9.97 (s, 1H), 8.99 (s, 1H), 8.56 (t, *J* = 6.1 Hz, 1H), 8.34 (t, *J* = 6.0 Hz, 1H), 7.84 (d, *J* = 9.4 Hz, 1H), 7.79–7.73 (m, 2H), 7.42 (d, *J* = 8.3 Hz, 2H), 7.39 (d, *J* = 8.3 Hz, 2H), 7.29 (d, *J* = 8.2 Hz, 2H), 5.12 (d, *J* = 3.6 Hz, 1H), 5.07 (s, 1H), 4.55 (d, *J* = 9.4 Hz, 1H), 4.46–4.41 (m, 2H), 4.37–4.34 (m, 1H), 4.29 (d, *J* = 6.0 Hz, 2H), 4.22 (dd, *J* = 15.8, 5.5 Hz, 1H), 3.70–3.62 (m, 2H), 2.77 (t, *J* = 7.1 Hz, 2H), 2.45 (s, 3H), 2.30–2.23 (m, 1H), 2.15–2.08 (m, 3H), 2.07–2.01 (m, 1H), 1.93–1.89 (m, 1H), 1.54–1.42 (m, 6H), 1.36–1.21 (m, 18H), 0.94 (s, 9H), 0.89–0.86 (m, 3H). ^13^C NMR (151 MHz, DMSO-*d*_6_) δ 172.7, 172.5, 172.4, 170.2, 165.5, 151.9, 148.2, 143.6, 140.0, 132.1, 131.6, 130.1, 129.1, 127.9, 127.5, 127.4, 69.3, 59.1, 56.8, 56.7, 51.7, 42.2, 42.1, 40.5, 40.4, 38.4, 35.8, 35.7, 35.3, 31.7, 29.4, 29.4, 29.2, 29.1, 28.1, 26.8, 26.8, 25.9, 25.8, 22.6, 16.4, 14.4. HRMS, calculated 437.7666 for C_48_H_73_N_7_O_6_S [M + 2H]^2+^, found 437.7665. Purity: 96.1%.

#### 1-(2-(2,6-Dioxopiperidin-3-yl)-1,3-dioxoisoindolin-5-yl)piperidine-4-carboxylic acid (**16a**)

A solution of **15** (200 mg, 0.72 mmol), *tert*-butyl piperidine-4-carboxylate (193 mg, 0.87 mmol) and DIPEA (515 μL, 2.9 mmol) in 3 mL of DMSO was heated at 90 °C overnight. Then, the solution was diluted with EtOAc, washed with water and brine. The organic layer was concentrated to give crude product, which was dissolved in 3 mL of DCM. Then, 1.5 mL of TFA was added dropwise at 0 °C. The resulting solution was stirred at room temperature for 4 h. Then, the solvents were removed to give 160 mg of **16a** as a yellow solid in a yield of 57%. ^1^H NMR (500 MHz, DMSO-*d*_6_) δ 11.09 (s, 1H), 7.67 (dd, *J* = 8.5, 2.9 Hz, 1H), 7.34 (d, *J* = 2.5 Hz, 1H), 7.26 (dd, *J* = 8.6, 2.5 Hz, 1H), 5.08 (dd, *J* = 12.8, 5.4 Hz, 1H), 4.00–3.96 (m, 2H), 3.16–3.05 (m, 2H), 2.89 (ddd, *J* = 16.5, 13.7, 5.5 Hz, 1H), 2.63–2.53 (m, 3H), 2.05–1.98 (m, 1H), 1.91 (dd, *J* = 13.7, 3.9 Hz, 2H), 1.66–1.54 (m, 2H).

#### 1-(2-(2,6-Dioxopiperidin-3-yl)-1,3-dioxoisoindolin-5-yl)-N-(4-(2-hexylhydrazine-1-carbonyl)benzyl)piperidine-4-carboxamide (**Z14**)

Using the method A for amide formation, compound **16a** and compound **12** gave 29 mg of **Z14** as a yellow solid in a yield of 25%. ^1^H NMR (600 MHz, DMSO-*d*_6_) δ 11.08 (s, 1H), 9.96 (d, *J* = 5.4 Hz, 1H), 8.44 (t, *J* = 5.9 Hz, 1H), 7.76 (d, *J* = 8.3 Hz, 2H), 7.67 (d, *J* = 8.6 Hz, 1H), 7.34 (d, *J* = 2.3 Hz, 1H), 7.30 (d, *J* = 8.3 Hz, 2H), 7.26 (dd, *J* = 8.7, 2.4 Hz, 1H), 5.07 (dt, *J* = 14.7, 7.4 Hz, 2H), 4.31 (d, *J* = 6.0 Hz, 2H), 4.09 (dd, *J* = 10.5, 6.7 Hz, 2H), 3.05–3.00 (m, 2H), 2.92–2.86 (m, 1H), 2.77 (q, *J* = 6.6 Hz, 2H), 2.63–2.53 (m, 2H), 2.04–2.00 (m, 1H), 1.84–1.81 (m, 2H), 1.68–1.61 (m, 2H), 1.47–1.42 (m, 2H), 1.36–1.24 (m, 6H), 0.89–0.85 (m, 3H). ^13^C NMR (151 MHz, DMSO-*d*_6_) δ 174.4, 173.3, 170.6, 168.1, 167.4, 165.5, 155.3, 143.5, 134.5, 132.1, 127.5, 127.3, 125.5, 118.2, 118.1, 108.4, 51.7, 49.2, 47.4, 42.1, 42.0, 40.5, 31.7, 31.4, 28.1, 28.0, 26.8, 22.7, 22.6, 14.4. HRMS, calculated 617.3083 for C_33_H_41_N_6_O_6_ [M + H]^+^, found 617.3081. Purity: 95.3%.

#### 1-(2-(2,6-Dioxopiperidin-3-yl)-1,3-dioxoisoindolin-5-yl)piperidine-4-carbaldehyde (**16b**)

A solution of **15** (500 mg, 1.8 mmol), piperidin-4-ylmethanol (250 mg, 2.2 mmol) and DIPEA (964 μL, 5.4 mmol) in 3 mL of DMSO was heated at 90 °C overnight. Then, the solution was diluted with EtOAc, washed with water and brine. The organic layer was concentrated to give crude product, which was dissolved in 5 mL of DCM at 0 °C. Then, 5 mL of 10% Dess–Martin oxidant in DCM was added dropwise. The resulting solution was warmed to room temperature and stirred for 3 h. Then, the solution was concentrated and dissolved in EtOAc, washed with NaHCO_3_(aq) and brine. The organic layer was concentrated and purified by column chromatography (DCM/MeOH = 120:1–80:1) to give 350 mg of **16b** as a yellow solid in a yield of 53%. ^1^H NMR (500 MHz, DMSO-*d*_6_) δ 11.10 (d, *J* = 3.7 Hz, 1H), 9.63 (d, *J* = 3.8 Hz, 1H), 7.67 (dt, *J* = 5.4, 2.8 Hz, 1H), 7.35 (t, *J* = 3.0 Hz, 1H), 7.28–7.24 (m, 1H), 5.10–5.06 (m, 1H), 3.98–3.93 (m, 2H), 3.22–3.12 (m, 2H), 2.95–2.83 (m, 1H), 2.72–2.53 (m, 3H), 2.05–2.02 (m, 1H), 1.97–1.89 (m, 2H), 1.61–1.53 (m, 2H).

#### 4-((((1-(2-(2,6-Dioxopiperidin-3-yl)-1,3-dioxoisoindolin-5-yl)piperidin-4-yl)methyl)amino)methyl)-*N*′-hexylbenzohydrazide (**Z15**)

A solution of compound **12** (40 mg, 0.12 mmol), **16b** (46 mg, 0.12 mmol), DIPEA (54 μL, 0.31 mmol), and NaBH(OAc)_3_ (53 mg, 0.25 mmol) in 3 mL of MeOH was stirred at room temperature for 4 h. Then, the solvents were removed and the resulting residue was diluted with EtOAc, washed with water. Then, the organic layer was concentrated and further purified by column chromatography (DCM/MeOH = 100:1–20:1) to give 15 mg of **Z15** as a yellow solid in a yield of 19%. ^1^H NMR (600 MHz, DMSO-*d*_6_) δ 11.07 (s, 1H), 9.95 (s, 1H), 7.78–7.74 (m, 2H), 7.65 (d, *J* = 8.6 Hz, 1H), 7.40 (d, *J* = 8.0 Hz, 2H), 7.30 (d, *J* = 2.3 Hz, 1H), 7.23 (dd, *J* = 8.7, 2.4 Hz, 1H), 5.10–4.97 (m, 2H), 4.05 (dt, *J* = 13.2, 3.4 Hz, 2H), 3.75 (s, 2H), 2.95 (td, *J* = 12.9, 2.7 Hz, 2H), 2.88 (ddd, *J* = 17.0, 13.8, 5.5 Hz, 1H), 2.77 (t, *J* = 7.2 Hz, 2H), 2.64–2.53 (m, 2H), 2.39 (dd, *J* = 5.1, 3.1 Hz, 2H), 2.03–1.99 (m, 1H), 1.86–1.79 (m, 2H), 1.78–1.68 (m, 1H), 1.45 (p, *J* = 7.2 Hz, 2H), 1.37–1.22 (m, 7H), 1.22–1.12 (m, 2H), 0.87 (t, *J* = 6.9 Hz, 3H). ^13^C NMR (151 MHz, DMSO-*d*_6_) δ 173.3, 170.6, 168.1, 167.4, 165.7, 155.4, 134.5, 128.1, 127.3, 125.5, 118.0, 117.8, 108.2, 51.7, 49.2, 47.8, 40.5, 31.7, 31.4, 29.8, 28.1, 26.8, 22.7, 22.6, 14.4. HRMS, calculated 603.3290 for C_33_H_43_N_6_O_5_ [M + H]^+^, found 603.3288. Purity: 97.9%.

#### 3-((4-(Piperidin-4-yl)phenyl)amino)piperidine-2,6-dione hydrochloride (**18**)

A solution of compound **17** (756 mg, 2.7 mmol) and 3-bromopiperidine-2,6-dione (500 mg, 2.6 mmol) in 5 mL of DMF was cooled to 0 °C. Then, NaHCO_3_ (655 mg, 7.8 mmol) was added to the solution. The resulting mixture was stirred at 65 °C overnight. Then, the solution was diluted with EtOAc, washed with water and brine. The organic layer was concentrated to give the crude product, which was dissolved in 4 mL of DCM/MeOH (3:1), followed by the addition 2 mL of 4 N HCl in dioxane dropwise. The resulting solution was stirred at room temperature for 4 h. Then, the resulting mixture was filtered to give 400 mg of **18** as a pink solid in a yield of 45%. ^1^H NMR (500 MHz, DMSO-*d*_6_) δ 10.87 (s, 1H), 9.08 (d, *J* = 11.2 Hz, 1H), 8.94 (d, *J* = 11.4 Hz, 1H), 7.00 (d, *J* = 8.2 Hz, 2H), 6.76 (d, *J* = 8.1 Hz, 2H), 4.35 (dd, *J* = 11.6, 4.8 Hz, 1H), 3.30 (d, *J* = 12.5 Hz, 2H), 2.96–2.89 (m, 2H), 2.78–2.66 (m, 2H), 2.58 (dt, *J* = 17.6, 4.2 Hz, 1H), 2.10–2.02 (m, 1H), 1.96–1.75 (m, 5H).

#### *tert*-Butyl 2-(4-(4-((2,6-dioxopiperidin-3-yl)amino)phenyl)piperidin-1-yl)acetate (**19**)

A solution of compound **18** (300 mg, 0.93 mmol), *tert*-butyl 2-bromacate (151 μL, 1.0 mmol) and DIPEA (494 μL, 2.8 mmol) in 5 mL of ACN was heated at 70 °C overnight. Then, the solvents were removed and diluted with EtOAc, washed with water. The organic layer was concentrated and the resulting residue was triturated with Pentane/EtOAc to give 200 mg of **19** as an off-white solid in a yield of 53%. ^1^H NMR (500 MHz, DMSO-*d*_6_) δ 10.79 (s, 1H), 6.96 (d, *J* = 8.0 Hz, 2H), 6.61 (d, *J* = 8.1 Hz, 2H), 5.66 (d, *J* = 7.4 Hz, 1H), 4.33–4.23 (m, 1H), 3.10 (s, 2H), 2.89 (d, *J* = 10.7 Hz, 2H), 2.75 (ddd, *J* = 17.4, 12.0, 5.3 Hz, 1H), 2.63–2.54 (m, 1H), 2.32–2.21 (m, 3H), 2.13–2.08 (m, 1H), 1.90–1.82 (m, 1H), 1.67–1.65 (m, 2H), 1.63–1.52 (m, 2H), 1.43 (s, 9H). HRMS, calculated 402.2388 for C_22_H_32_N_3_O_4_ [M + H]^+^, found 402.2383.

#### 2-(4-(4-((2,6-Dioxopiperidin-3-yl)amino)phenyl)piperidin-1-yl)acetic acid (**20**)

A solution of compound **19** (200 mg, 0.50 mmol) in 2 mL of DCM was cooled to 0 °C. One mL of TFA was added dropwise to the solution. The resulting solution was stirred at room temperature overnight. Then, the solvents were removed to give 211 mg of **20** as a pale-green solid in a yield of 51%. ^1^H NMR (500 MHz, DMSO-*d*_6_) δ 10.80 (s, 1H), 9.83 (s, 1H), 6.97 (d, *J* = 8.0 Hz, 2H), 6.65 (d, *J* = 8.0 Hz, 2H), 4.30 (dd, *J* = 11.5, 4.6 Hz, 1H), 4.16 (d, *J* = 7.7 Hz, 2H), 3.57 (d, *J* = 12.8 Hz, 2H), 3.18–3.11 (m, 2H), 2.75 (ddd, *J* = 17.5, 12.1, 5.3 Hz, 1H), 2.64–2.57 (m, 2H), 2.10 (d, *J* = 12.7 Hz, 1H), 1.98–1.86 (m, 5H).

#### 2-(4-(4-((2,6-Dioxopiperidin-3-yl)amino)phenyl)piperidin-1-yl)-N-(4-(2-hexylhydrazine-1-carbonyl)benzyl)acetamide (**Z16**)

Using the method A for amide formation, compound **20** and compound **12** gave 22 mg of **Z16** as a pale-green solid in a yield of 38%. ^1^H NMR (600 MHz, DMSO-*d*_6_) δ 10.77 (s, 1H), 9.96 (s, 1H), 8.37 (t, *J* = 6.3 Hz, 1H), 7.77 (d, *J* = 8.3 Hz, 2H), 7.33 (d, *J* = 8.3 Hz, 2H), 7.00–6.92 (m, 2H), 6.66–6.55 (m, 2H), 5.65 (d, *J* = 7.5 Hz, 1H), 5.05 (s, 1H), 4.36 (d, *J* = 6.3 Hz, 2H), 4.29–4.25 (m, 1H), 3.00 (s, 2H), 2.91–2.89 (m, 2H), 2.80–2.68 (m, 3H), 2.58 (dt, *J* = 17.5, 4.3 Hz, 1H), 2.36–2.30 (m, 1H), 2.21–2.07 (m, 3H), 1.92–1.82 (m, 1H), 1.75–1.64 (m, 4H), 1.47–1.42 (m, 2H), 1.37–1.22 (m, 6H), 0.87 (t, *J* = 6.9 Hz, 3H). ^13^C NMR (151 MHz, DMSO-*d*_6_) δ 174.2, 173.6, 170.3, 165.5, 146.5, 143.7, 134.6, 132.1, 127.5, 127.4, 127.4, 113.1, 62.2, 55.4, 54.7, 53.1, 51.7, 42.1, 40.9, 40.5, 33.7, 31.7, 31.2, 28.1, 26.8, 25.2, 22.6, 14.4. HRMS, calculated 577.3497 for C_32_H_45_N_6_O_4_ [M + H]^+^, found 577.3495. Purity: 99.2%.

#### *tert*-Butyl 2-(4-((2-(4-(4-((2,6-dioxopiperidin-3-yl)amino)phenyl)piperidin-1-yl)acetamido)methyl)benzoyl)-1-hexylhydrazine-1-carboxylate (**NC-Z16**)

A solution of **Z16** (89 mg, 0.16 mmol), (Boc)_2_O (41 mg, 0.19 mmol) and TEA (33 μL, 0.23 mmol) in 3 mL of THF was stirred at 50 °C overnight. Afterward, the organic solvents were removed and further purified by column chromatography (DCM/MeOH = 100:1–30:1) give 40 mg of **NC-Z16** as a brown solid in a yield of 38%. ^1^H NMR (600 MHz, DMSO-*d*_6_) δ 10.77 (s, 1H), 10.46 (d, *J* = 11.2 Hz, 1H), 8.41 (s, 1H), 7.79 (dd, *J* = 25.6, 7.8 Hz, 2H), 7.37 (d, *J* = 7.8 Hz, 2H), 6.97 (d, *J* = 7.9 Hz, 2H), 6.62 (d, *J* = 7.9 Hz, 2H), 5.66 (d, *J* = 7.4 Hz, 1H), 4.43–4.33 (m, 2H), 4.30–4.20 (m, 1H), 3.41 (s, 2H), 3.06 (d, *J* = 24.1 Hz, 3H), 2.92 (s, 2H), 2.77–2.71 (m, 1H), 2.62–2.55 (m, 1H), 2.34 (brs, 1H), 2.24–2.05 (m, 3H), 1.89–1.83 (m, 1H), 1.72–1.69 (m, 4H), 1.49–1.41 (m, 6H), 1.32–1.27 (m, 11H), 0.86 (t, *J* = 7.7 Hz, 3H). ^13^C NMR (151 MHz, DMSO-*d*_6_) δ 174.2, 173.6, 165.9, 155.3, 146.5, 144.3, 134.5, 131.6, 128.0, 127.8, 127.5, 127.4, 113.2, 113.1, 79.8, 54.6, 53.1, 48.8, 46.2, 42.1, 40.5, 33.6, 31.5, 31.3, 31.2, 28.4, 28.3, 27.8, 27.6, 26.3, 26.1, 25.2, 22.5, 14.4. HRMS, calculated 677.4022 for C_37_H_53_N_6_O_6_ [M + H]^+^, found 677.4016. Purity: 97.9%.

#### Methyl 2-(4-((*tert*-butoxycarbonyl)amino)phenoxy)acetate (**22**)

A mixture of compound **21** (500 mg, 2.4 mmol), methyl bromoacetate (272 μL, 2.9 mmol), Cs_2_CO_3_ (935 mg, 2.9 mmol), and NaI (36 mg, 0.24 mmol) in 10 mL of acetone was heated at 60 °C overnight. Then, the mixture was concentrated and the resulting residue was diluted with EtOAc, washed with water and brine. The organic layer was concentrated and purified by column chromatography (Pentane/EtOAc = 20:1–5:1) to give 500 mg of **22** as a white solid in a yield of 74%. ^1^H NMR (500 MHz, DMSO-*d*_6_) δ 9.17 (s, 1H), 7.34 (d, *J* = 8.5 Hz, 2H), 6.87–6.80 (m, 2H), 4.73 (d, *J* = 1.1 Hz, 2H), 3.70 (d, *J* = 1.1 Hz, 3H), 1.47 (d, *J* = 1.1 Hz, 9H).

#### Methyl 2-(4-aminophenoxy)acetate (**23**)

A solution of compound **22** (462 mg, 2.0 mmol) in 2 mL of DCM was cooled to 0 °C. Then, 2 mL of TFA was added to the solution dropwise. The resulting solution was stirred at room temperature for 2 h. Then, the pH value of the solution was adjusted to 7 by the addition of saturated NaHCO_3_ (aq). The resulting mixture was diluted with DCM, washed with water and brine. The organic layer was concentrated to give 350 mg of **23** as a brown oil in a yield of 97%. ^1^H NMR (500 MHz, DMSO-*d*_6_) δ 6.68–6.62 (m, 2H), 6.50 (dd, *J* = 8.8, 1.0 Hz, 2H), 4.68 (s, 2H), 4.60 (s, 2H), 3.68 (d, *J* = 1.0 Hz, 3H).

#### 3,3′-((4-(2-Methoxy-2-oxoethoxy)phenyl)azanediyl)dipropionic acid (**24**)

A solution of compound **23** (1.5 g, 8.3 mmol) and acrylic acid (2.8 mL, 41.4 mmol) in 3 mL of H_2_O was heated at 70 °C overnight. Then, the solution was extracted with EtOAc, washed with water and brine. The organic layer was concentrated and purified by column chromatography (DCM/MeOH = 100:1–20:1) to give 700 mg of **24** as a brown oil in a yield of 26%. ^1^H NMR (500 MHz, DMSO-*d*_6_) δ 12.33 (s, 2H), 6.82 (d, *J* = 8.6 Hz, 2H), 6.67 (d, *J* = 8.6 Hz, 2H), 4.67 (s, 2H), 3.70 (d, *J* = 1.1 Hz, 3H), 2.57 (t, *J* = 6.3 Hz, 2H), 2.47 (t, *J* = 6.8 Hz, 2H), 2.40 (t, *J* = 7.1 Hz, 2H).

#### 2-(4-(2,4-Dioxotetrahydropyrimidin-1(2H)-yl)phenoxy)acetic acid (**25**)

A solution of compound **24** (700 mg, 2.1 mmol) and urea (194 mg, 3.2 mmol) in 5 mL of acetic acid was heated at 120 °C overnight. Then, the solvents were removed and the resulting residue was added to 5 mL of 4 N HCl (aq). The resulting mixture was heated at 120 °C for 1 h. Then, the reaction solution was cooled to ambient temperature and the precipitate was collected by filtration to give 130 mg of **25** as an off-white solid in a yield of 23%. ^1^H NMR (500 MHz, DMSO-*d*_6_) δ 13.03 (s, 1H), 10.33 (s, 1H), 7.27–7.17 (m, 2H), 6.98–6.90 (m, 2H), 4.69 (d, *J* = 1.1 Hz, 2H), 3.78–3.65 (m, 2H), 2.70 (t, *J* = 6.6 Hz, 2H).

#### 2-(4-(2,4-Dioxotetrahydropyrimidin-1(2H)-yl)phenoxy)-N-(4-(2-hexylhydrazine-1-carbonyl)benzyl)acetamide (**Z17**)

Using the method A for amide formation, compound **25** and compound **12** gave 35 mg of **Z17** as a brown solid in a yield of 57%. ^1^H NMR (500 MHz, DMSO-*d*_6_) δ 10.34 (s, 1H), 9.98 (d, *J* = 4.7 Hz, 1H), 8.73 (t, *J* = 6.2 Hz, 1H), 7.76 (d, *J* = 7.8 Hz, 2H), 7.32 (d, *J* = 7.8 Hz, 2H), 7.27 (d, *J* = 8.4 Hz, 2H), 7.00 (d, *J* = 8.5 Hz, 2H), 5.06 (s, 1H), 4.59 (s, 2H), 4.39 (d, *J* = 6.1 Hz, 2H), 3.73 (t, *J* = 6.6 Hz, 2H), 2.71 (t, *J* = 6.6 Hz, 2H), 1.44 (q, *J* = 7.2 Hz, 2H), 1.36–1.25 (m, 6H), 0.87 (t, *J* = 6.7 Hz, 3H). ^13^C NMR (151 MHz, DMSO-*d*_6_) δ 173.3, 170.6, 168.1, 167.4, 165.6, 155.4, 134.5, 128.2, 127.3, 125.5, 118.0, 117.8, 108.2, 51.7, 49.2, 47.8, 40.5, 31.7, 29.8, 28.1, 26.8, 22.6, 14.4. HRMS, calculated 496.2555 for C_26_H_34_N_5_O_5_ [M + H]^+^, found 496.2549. Purity: 97.0%.

#### *N*′-Hexyl-3-nitrobenzohydrazide (**27**)

A solution of compound **26** (500 mg, 2.76 mmol) and hexanal (356 μL, 2.90 mmol) in 5 mL of MeOH was stirred at room temperature for 2 h. Then, NaBH_4_ (209 mg, 5.52 mmol) was added to the mixture at 0 °C. Then, the resulting solution was further stirred at room temperature for 1 h. Afterward, the solvents were removed and the resulting residue was diluted with EtOAc, washed with water. The organic layer was concentrated and triturated by Pentane/EtOAc to give 522 mg of **27** as a yellow solid in a yield of 72%. ^1^H NMR (600 MHz, DMSO-*d*_6_) δ 10.38 (d, *J* = 5.9 Hz, 1H), 8.65 (t, *J* = 2.0 Hz, 1H), 8.39–8.37 (m, 1H), 8.27 (dt, *J* = 7.8, 1.3 Hz, 1H), 7.78 (t, *J* = 8.0 Hz, 1H), 5.20–5.17 (m, 1H), 2.85–2.75 (m, 2H), 1.49–1.44 (m, 2H), 1.37–1.26 (m, 6H), 0.87 (t, *J* = 6.9 Hz, 3H).

#### *tert*-Butyl 2-(3-((4-(tert-butoxycarbonyl)benzyl)amino)benzoyl)-1-hexylhydrazine-1-carboxylate (**28**)

A solution of compound **27** (450 mg, 1.7 mmol), (Boc)_2_O (444 mg, 2.0 mmol) and TEA (355 μL, 2.6 mmol) in 10 mL of THF was stirred at 50 °C overnight. Then, the solution was concentrated and purified by column chromatography (Pentane/EtOAc = 10:1–3:1) to give 350 mg colorless oil, which was dissolved in 6 mL of MeOH/EtOAc (1:1). Then, Pd/C (35 mg, 10 wt %) was added to the solution. The resulting mixture was stirred at room temperature under H_2_ atmosphere overnight. Then, the mixture was filtered over Celite and the resulting filtrate was concentrated to give a white solid. A mixture of the above white solid, K_2_CO_3_ (199 mg, 1.4 mmol) and *tert*-butyl 4-(bromomethyl)benzoate (272 mg, 1.0 mmol) in 5 mL of DMF was stirred at room temperature overnight. Afterward, the mixture was diluted with EtOAc, washed with water and brine. The organic layer was concentrated to give 260 mg of **28** as a colorless oil in a yield of 51%, which was used directly for next step.

#### 4-(((3-(2-Hexylhydrazine-1-carbonyl)phenyl)amino)methyl)benzoic acid (**29**)

To a solution of compound **28** (260 mg, 0.49 mmol) in 3 mL of DCM was added 1.5 mL of TFA dropwise. Then, the resulting solution was stirred at room temperature for 4 h. Afterward, the solvents were removed and the resulting residue was dried to give 160 mg of **29** as an off-white solid in a yield of 88%. ^1^H NMR (600 MHz, DMSO-*d*_6_) δ 11.09 (s, 1H), 7.93–7.87 (m, 2H), 7.47 (d, *J* = 8.1 Hz, 2H), 7.19 (t, *J* = 7.9 Hz, 1H), 7.08–7.03 (m, 1H), 7.00 (d, *J* = 7.6 Hz, 1H), 6.79 (dd, *J* = 8.1, 2.3 Hz, 1H), 4.41 (s, 2H), 3.10–3.00 (m, 2H), 1.59–1.53 (m, 2H), 1.35–1.24 (m, 6H), 0.87 (t, *J* = 6.9 Hz, 3H).

#### 3-((4-(4-(4-((2,6-Dioxopiperidin-3-yl)amino)phenyl)piperidine-1-carbonyl)benzyl)amino)-*N*′-hexylbenzohydrazide (**Z18**)

Using the method A for amide formation, compound **29** and compound **18** gave 24 mg of **Z18** as an off-white solid in a yield of 28%. ^1^H NMR (600 MHz, DMSO-*d*_6_) δ 10.77 (s, 1H), 9.87 (s, 1H), 7.42 (d, *J* = 8.0 Hz, 2H), 7.38 (d, *J* = 8.1 Hz, 2H), 7.10 (t, *J* = 7.8 Hz, 1H), 7.05 (t, *J* = 2.0 Hz, 1H), 7.00–6.93 (m, 3H), 6.71 (dd, *J* = 8.2, 2.4 Hz, 1H), 6.62 (d, *J* = 8.4 Hz, 2H), 6.50 (t, *J* = 6.2 Hz, 1H), 5.68 (d, *J* = 7.5 Hz, 1H), 4.35 (d, *J* = 5.3 Hz, 2H), 4.29–4.25 (m, 1H), 2.88–2.69 (m, 4H), 2.66–2.55 (m, 2H), 2.12–2.08 (m, 1H), 1.90–1.60 (m, 3H), 1.58–1.47 (m, 2H), 1.46–1.41 (m, 2H), 1.36–1.22 (m, 6H), 0.87 (t, *J* = 6.9 Hz, 3H). ^13^C NMR (151 MHz, DMSO-*d*_6_) δ 174.2, 173.6, 169.3, 166.4, 149.0, 146.6, 141.9, 135.3, 134.4, 133.9, 129.2, 127.5, 127.3, 115.4, 114.8, 113.1, 111.4, 55.4, 53.0, 51.6, 46.5, 41.5, 40.5, 31.7, 31.2, 27.9, 26.8, 25.2, 22.5, 14.4. HRMS, calculated 639.3654 for C_37_H_47_N_6_O_4_ [M + H]^+^, found 639.3650. Purity: 96.7%.

#### *tert*-Butyl 2-(4-(4-acetamidophenyl)piperidin-1-yl)acetate (**30**)

A solution of **17** (500 mg, 1.8 mmol) and TEA (631 μL, 4.5 mmol) in 5 mL of THF was cooled to 0 °C, followed by the addition of acetyl chloride (192 μL, 2.7 mmol) dropwise. The resulting mixture was stirred at room temperature for 1 h. Then, the solvents were removed and the resulting residue was diluted with EtOAc, washed with water. The organic layer was concentrated to give a pink solid, which was directly dissolved in 4 mL of DCM. Then, 2 mL of TFA was added to the solution dropwise. The resulting solution was stirred at room temperature overnight. Afterward, the solvents were removed and the resulting residue was dissolve in 10 mL of ACN, followed by the addition of DIPEA (629 μL, 3.6 mmol) and *tert*-butyl 2-bromoacetate (265 μL, 1.8 mmol). The resulting solution was heated at 70 °C for 2 h. Then, the solvents were removed and the resulting residue was diluted with EtOAc, washed with water. The organic layer was concentrated to give 280 mg of **30** as a brown solid in a yield of 47% (three steps). ^1^H NMR (500 MHz, DMSO-*d*_6_) δ 9.85 (s, 1H), 7.48 (d, *J* = 7.8 Hz, 2H), 7.16 (d, *J* = 8.2 Hz, 2H), 3.12 (s, 2H), 2.92 (d, *J* = 10.5 Hz, 2H), 2.44–2.35 (m, 1H), 2.26 (q, *J* = 14.1, 12.9 Hz, 2H), 2.02 (s, 2H), 1.71–1.58 (m, 4H), 1.43 (s, 9H).

#### 2-(4-(4-Acetamidophenyl)piperidin-1-yl)acetic acid (**31**)

Using the synthetic method for compound **20**, compound **30** gave 228 mg of **31** as an off-white solid in a yield of 73%. ^1^H NMR (500 MHz, DMSO-*d*_6_) δ 9.92 (s, 1H), 7.53 (t, *J* = 5.4 Hz, 2H), 7.18 (d, *J* = 7.9 Hz, 2H), 4.17 (s, 2H), 3.63–3.52 (m, 2H), 3.17 (d, *J* = 12.1 Hz, 2H), 2.78–2.72 (m, 1H), 2.03 (s, 3H), 1.96 (brs, 4H). HRMS, calculated 277.1547 for C_16_H_21_N_2_O_3_ [M + H]^+^, found 277.1541.

#### 2-(4-(4-Acetamidophenyl)piperidin-1-yl)-N-(4-(2-hexylhydrazine-1-carbonyl)benzyl)acetamide (**32**)

Using the method A for amide formation, compound **31** and compound **12** gave 36 mg of **32** as a white solid in a yield of 26%. ^1^H NMR (500 MHz, DMSO-*d*_6_) δ 9.96 (d, *J* = 5.8 Hz, 1H), 9.84 (s, 1H), 8.38 (t, *J* = 6.1 Hz, 1H), 7.77 (d, *J* = 7.7 Hz, 2H), 7.49 (d, *J* = 7.9 Hz, 2H), 7.34 (d, *J* = 7.6 Hz, 2H), 7.16 (d, *J* = 8.0 Hz, 2H), 5.05 (q, *J* = 6.0 Hz, 1H), 4.36 (d, *J* = 6.1 Hz, 2H), 3.01 (s, 2H), 2.91 (d, *J* = 10.9 Hz, 2H), 2.76 (t, *J* = 6.2 Hz, 2H), 2.43 (dq, *J* = 11.0, 5.0 Hz, 1H), 2.17 (d, *J* = 11.1 Hz, 2H), 2.02 (d, *J* = 3.6 Hz, 3H), 1.80–1.68 (m, 4H), 1.44 (q, *J* = 7.1 Hz, 2H), 1.35–1.26 (m, 6H), 0.87 (t, *J* = 6.6 Hz, 3H). ^13^C NMR (151 MHz, DMSO-*d*_6_) δ 170.3, 168.5, 165.5, 143.7, 141.3, 137.8, 132.1, 127.5, 127.4, 127.2, 119.6, 62.2, 54.5, 51.7, 42.1, 41.2, 40.5, 33.4, 31.7, 28.1, 26.8, 24.4, 22.5, 14.4. HRMS, calculated 508.3283 for C_29_H_42_N_5_O_3_ [M + H]^+^, found 508.3274. Purity: 99.4%.

### Molecular Docking Study

Molecular docking studies were performed using Maestro 13.9 module in the Schrödinger 2024–1. The crystal structures of HDAC8 (PDB code: 1T69), HDAC6 (PDB Code: 5EDU), and HDAC2 (PDB code: 4LXZ) were downloaded from protein data bank (https://www.rcsb.org/) and subjected to Maestro’s protein preparation module. The protein structures were prepared and optimized by protein preparation workflow module using OPLS4 force field. The molecular structure of compound **6** was imported to Maestro 13.9 module and optimized using the LigPrep module. The Grid files were generated using Receptor Grid Generation. Molecular docking was carried out via Ligand Docking module with Standard-Precision (SP) mode. Other parameters were set as default values. The docking results were visualized by PyMOL(https://www.pymol.org/).

### Cell Culture

Jurkat, THP-1, HCT116, HEL, and A549 cell lines were cultured in RPMI 1640 medium containing 10% (v/v) fetal bovine serum (FBS), 100 U/ml penicillin/streptomycin (Gibco by life Technologies, Bleiswijk, The Netherlands) at 37 °C with 5% CO_2_ in humidified air.

### Preparation of Compound Solutions

All the compounds were dissolved in DMSO to give 10 mM of stock solutions. Treatment groups were treated with indicated concentration of compounds diluted in assay buffer or cell culture media, while the vehicle groups were treated with DMSO which is diluted to the corresponding highest concentration of comppounds. The DMSO concenration for all the treatment groups is below 0.5%.

### Western Blot

After treated with different concentration of compounds for indicated time, cells were harvested and washed with cold 1X PBS twice, and subsequently lysed with RIPA lysis buffer supplemented with complete protease inhibitor cocktail, EDTA-free (Roche, Basel, Switzerland) inside. For acetylated proteins, 10 mM SAHA was also added to RIPA lysis buffer. Protein concentrations were determined by a BCA Protein Assay Kit (Thermo Fisher Scientific, USA) according to the manufacturer’s protocol. Samples were separated by NuPAGE 4–12% Bis-Tris gels (Invitrogen, Carlsbad, Canada), and transferred onto polyvinylidene fluoride (PVDF) or nitrocellulose (NC) membranes. The membranes were blocked at room temperature for 1–2 h in 0.1% PBST solution containing 5% skimmed milk, and subsequently incubated at 4 °C overnight with one of the following primary antibodies: anti-HDAC1 (1:1000 Cell Signaling, #5356), anti-HDAC2 (1:1000 Cell Signaling, #5113), anti-HDAC3 (1:1000 Cell Signaling, #3949), anti-HDAC4 (1:2000 Cell Signaling, #7628), anti-HDAC6 (1:1000, Cell Signaling, #7612), anti-HDAC7 (1:1000 Cell Signaling, #33418), anti-HDAC8 (1:1000 Cell Signaling, #66042), anti-HDAC11 (1:1000 Cell Signaling, #58442), anti-Acetyl-Histone H3 (Lys27) (1:1000 Cell Signaling, #8173), anti-Acetyl-tubulin (1:1000 Cell Signaling, #5335), anti-Acetyl SMC3 (1:1000 Sigma-Aldrich, MABE1073), anti-Acetyl-Histone H3 (Lys9/Lys14/LysK18/Lys23/Lys27) (1:1000 abcam, ab47915), anti-Acetyl-Histone H4 (Lys12/Lys16/Lys5/Lys8) (1:1000 abcam, ab177790), anti-IKZF1 (1:2000 Invitrogen, PA5-98433), anti-IKZF3 (1:500 Invitrogen, 720418), anti-GSPT1 (eRF3) (1:1000 Cell Signaling, #14980), anti-GAPDH (1:10000 Cell Signaling, #5174) in 0.1% PBST solution containing 5% BSA. Membranes were washed three times in 0.1% PBST and incubated at room temperature for 1 h with corresponding secondary antibodies: goat antirabbit HRP-conjugated secondary antibody (1:2000 DAKO, P0448) or rabbit antimouse HRP-conjugated secondary antibody (1:2000 DAKO, P0260). The bands were visualized via enhanced chemiluminescence (ECL) Western blot detection (cytiva, RPN2232). All expression levels were analyzed by the ImageJ software and normalized to GAPDH expression.

### Cell Viability Assay

MTS assay was used to measure the cell viability. For single treatment, Jurkat, THP-1 and HEL (8000 cells/well), HCT116 and A549 (2000 cells/well) were seeded in 96-well plates in 100 μL complete medium. After overnight incubation, 50 μL of media containing various concentrations of compounds or the vehicle were added to each well. For cotreatment, cells were seeded in 96-well plates in 50 μL complete medium. After overnight incubation, 50 μL of Z-VAD-FMK, Nec-1, Fer-1, CQ, and other indicated compounds were added to each well. One hour later, 50 μL of media containing various concentrations of compounds were added to each well. After 72 h of treatment, 20 μL of CellTiter 96 AQueous One Solution reagent (Promega, Madison, USA) was added to each well according to manufacturer’s protocol. Plates were incubated at 37 °C for 1–2 h. The absorbance was determined at a wavelength of 490 nm using a Synergy H1 plate reader (Biotek, Winooski, VT, USA). Nonlinear regression was used for the determination of the IC_50_ of each compound.

### In Vitro HDAC1/2/3/8 Deacetylase Inhibition Assay

Human recombinant C-terminal FLAG-tag, C-terminal His-tag HDAC1 (BPS Bioscience, Catalog#: 50051), Human recombinant C-terminal FLAG-tag HDAC2 (BPS Bioscience, Catalog#: 50052), human recombinant C-terminal His-tag HDAC3/NcoR2 (BPS Bioscience, Catalog#: 50003) and human recombinant C-terminal His-tag HDAC8 (BPS Bioscience, Catalog#: 50008) were diluted in assay buffer (25 mM Tris-HCl, pH 8.0, 137 mM NaCl, 2.7 mM KCl, 1 mM MgCl_2_, and 0.1 mg/mL BSA), HDAC1(1.87 ng/μL), HDAC2 (1.87 ng/μL), HDAC3 (0.89 ng/μL), and HDAC8 (1.16 ng/μL). 40 μL of enzyme solution was incubated with 10 μL of different concentrations of inhibitors dissolved in assay buffer for 10 min at 37 °C. Then, 50 μL of the HDAC1/2/3 fluorogenic substrate Boc-Lys(Ac)-AMC (20 mM, Bachem, Germany) or the HDAC8 fluorogenic substrate Boc-Lys(TFA)-AMC (20 mM, Bachem, Germany) was added to the plates and incubated at 37 °C for 30 min. Then, 50 μL of the stop solution (25 mM Tris-HCl (pH 8), 137 mM NaCl, 2.7 mM KCl, 1 mM MgCl_2_, 6.0 mg/mL trypsin (porcine pancreas Type IX-S, Sigma-Aldrich) and 10 mM SAHA) was added to each well. After a following incubation at 37 °C for 20 min, the fluorescence was measured on a Synergy H1 Platereader (BioTek, USA) with an excitation wavelength of 370 nm and an emission wavelength of 460 nm. Nonlinear regression was used for the determination of the IC_50_ of each compound.

### Caspase 3/7 Activity Assay

Jurkat cells (10000 cells/well) and HCT116 (2000 cells/well) were seeded in white-walled 96-well plates (Greiner bio-one, Alphen a/d Rijn, The Netherlands) in 50 μL complete medium and cultured overnight. Then, 50 μL of compounds were added to the plates and incubated for 48 h. The Caspase-Glo 3/7 Reagent (Promega Corporation, Madison, WI, USA) was equilibrated to room temperature, and 100 μL of the reagent was added to each well. After incubation at room temperature for 1 h, the luminescence was collected using a Synergy H1 plate reader (Biotek, Winooski, VT, USA).

### Apoptosis Assay

The cell apoptosis assay was performed using eBioscience Annexin V-FITC/PI Apoptosis Detection Kit (Thermo Fisher Scientific, Carlsbad, CA, USA). Jurkat cells (5 × 10^5^) and HCT116 cells (3 × 10^5^) were treated with different concentrations of test compounds for 24 or 48 h. Cells were harvested after incubation, washed twice in cold PBS, centrifuged, and resuspended in 1X annexin-binding buffer. According to the manufacturer’s instructions, cells were incubated with Annexin V and Propidium iodide (PI) sequentially. All samples were analyzed by NovoCyte Quanteon Flow Cytometer (Agilent Technologies, CA, USA).

### Lipid Peroxidation Detection

Jurkat cells (5 × 10^5^), HCT116 cells (3 × 10^5^) and THP-1 cells (5 × 10^5^) were seeded on 6-well plates. The next day, cells were treated with indicated concentrations of compounds for 24 or 48 h. Then, cells were stained with 1.3 μM of BODIPY 581/591 C11 (Invitrogen, D3861) for another 1 h at 37 °C. Later, cells were harvested and washed three times with ice-cold PBS, followed by resuspended in FACS buffer (1% BSA in PBS). Lipid peroxidation was determined by NovoCyte Quanteon Flow Cytometer (Agilent Technologies, CA, USA).

### Cell Death Determination by Flow Cytometry

Jurkat cells (5 × 10^5^) were seeded on 6-well plates. The next day, cells were treated with indicated concentrations of compounds for 48 h. Cells were harvested after incubation, washed twice in cold PBS, centrifuged, and stained with 7 μM Hoechst 33342 and 2 μM of Propidium iodide (PI) sequentially, and then determined by NovoCyte Quanteon Flow Cytometer (Agilent Technologies, CA, USA). Cells stained only with Hoechst 33342 were counted as live cells, while cells stained with both Hoechst 33342 and PI were counted as dead cells.

### Cell Cycle Analysis

Jurkat cells (5 × 10^5^) and HCT116 cells (3 × 10^5^) were seeded on 6-well plates. The next day, cells were treated with indicated concentrations of compounds for 48 h. Cells were harvested after incubation, washed twice in cold PBS, centrifuged, and stained with a solution containing 20 μg/mL of Propidium iodide (PI) and 0.1% (v/v) Triton-X100 for 15 min at room temperature, followed by detection with NovoCyte Quanteon Flow Cytometer (Agilent Technologies, CA, USA).

## Supporting Information Available

The in vitro HDAC1/2/3/8 inhibition results of **Z16**. The in vitro antiproliferative activities of **Z16**, **32**, and SAHA. The effects of **Z16** on HDAC1 and HDAC6 levels in HCT116 cells, and STAT3 levels in Jurkat and HCT116 cells. The effects of **Z16** and **Z16**+Bortezomib on HDAC8 and Ac-HH3 in A549 cells. The effects of PCI-34051 on Ac-HH3 and Ac-HH4 in Jurkat cells. Apoptosis results of **Z16** in Jurkat cells. Lipid peroxidation results of **Z16** in Jurkat, HCT116, and THP-1 cells after 24 and 48 h treatment. Cell death determined by Flow cytometry. ^1^H NMR, ^13^C NMR, HRMS spectra, and HPLC spectra of representative target compounds. Uncropped Western blots. (PDF)
The proposed binding modes of compound **6** in HDAC8 (PDB)
The proposed binding modes of compound **6** in HDAC6 (PDB)
The proposed binding modes of compound **6** in HDAC2 (PDB)
Molecular formula strings (CSV)

## Supplementary Material

jm4c00836_si_001.pdf

jm4c00836_si_002.pdb

jm4c00836_si_003.pdb

jm4c00836_si_004.pdb

## ABBREVIATIONS

AML: acute myeloid leukemia
CRBN: cereblon
CDI: carbonyldiimidazole
DCM: dichloromethane
DIPEA: *N,N*-diisopropylethylamine
DMF: *N,N*-dimethylformamide
EDCI: 1-Ethyl-3-(3-(dimethylamino)propyl)carbodiimide
ERRα: estrogen-related receptor alpha
HDAC: histone deacetylase
HATU: 1-[Bis(dimethylamino)methylene]-1H-1,2,3-triazolo[4,5-*b*]pyridinium 3-oxide hexafluorophosphate
HOBT: hydroxybenzotriazole
MTS: 3-(4,5-dimethylthiazol-2-yl)-5-(3-carboxymethoxyphenyl)-2-(4-sulfophenyl)-2H-tetrazolium
MeOH: methanol
PROTAC: proteolysis targeting chimera
ROS: reactive oxygen species
SMC3: structural maintenance of chromosomes protein 3
STAT3: signal transducer and activator of transcription 3
TFA: trifluoroacetic acid
THF: tetrahydrofuran
VHL: von Hippel–Lindau
