# Supplementary material for: Exploration of Hydrazide-Based HDAC8 PROTACs for the Treatment of Hematological Malignancies and Solid Tumors
Source: J Med Chem. 2024 Aug 1;67(16):14016–39. doi: 10.1021/acs.jmedchem.4c00836 (PMC11345830; doi:10.1021/acs.jmedchem.4c00836)
Supplement: Supplementary file 1 — jm4c00836_si_001.pdf [file jm4c00836_si_001.pdf]

Supporting Information

Exploration of Hydrazide-Based HDAC8 PROTACs for the Treatment of Hematological Malignancies and Solid Tumors

Chunlong Zhao[+], Jianqiu Zhang[+], Hangyu Zhou, Rita Setroikromo, Gerrit J. Poelarends, Frank J. Dekker[*]

Department of Chemical and Pharmaceutical Biology, Groningen Research Institute of Pharmacy (GRIP), University of Groningen, Antonius Deusinglaan 1, 9713 AV Groningen, The Netherlands

Corresponding Authors

Frank J. Dekker Department of Chemical and Pharmaceutical Biology, Groningen Research Institute of Pharmacy (GRIP), University of Groningen, Antonius Deusinglaan 1, 9713AV Groningen, The Netherlands.

Email: f.j.dekker@rug.nl

Table of Contents

S3 – S11: Supporting figures

Figure S1. The proposed binding modes of compound **6** in HDAC8, HDAC6 and HDAC2 (C, PDB Code: 4LXZ). The co-crystal complexes of thalidomide in cereblon (CRBN) and the VHL ligand/VHL E3 ligase.

Figure S2. The in vitro HDAC1/2/3/8 deacetylase inhibitory activity of **Z16**.

Figure S3. The effect of **Z16** on HDAC1 and HDAC6 levels in HCT116 cells.

Figure S4. The *in vitro* antiproliferative activity of compound **Z16**.

Figure S5. The effect of PCI-34051 on histone acetylation in Jurkat cells and the antiproliferative activity of PCI-34051 against Jurkat cells in the absence and presence of **Z16**.

Figure S6.The effects of **Z16** and SAHA on HDAC8 and histone acetylation in HCT116 cells and Jurkat cells.

Figure S7. The effects of **Z16** and **Z16**+Bortezomib cotreatment on HDAC8 and Ac-HH3 levels in A549 cells.

Figure S8 The effect of **Z16** on STAT3 level in Jurkat and HCT116 cells.

Figure S9. The apoptosis in Jurkat cells treated with indicated compounds for 24 h and 48 h.

Figure S10. The antiproliferative activities of **Z16** and SAHA in the absence or presence of various death inhibitors.
Figure S11.The lipid peroxidation level in Jurkat, HCT116, and THP-1 cells treated with indicated compounds for 24 h or 48 h.
Figure S12. Jurkat cell death determined by flow cytometry of **Z16** in the absence and presence of various cell death inhibitors.

S13 – S20: Uncropped western blots

S21 – S54: Representative NMR, HRMS and HPLC spectra

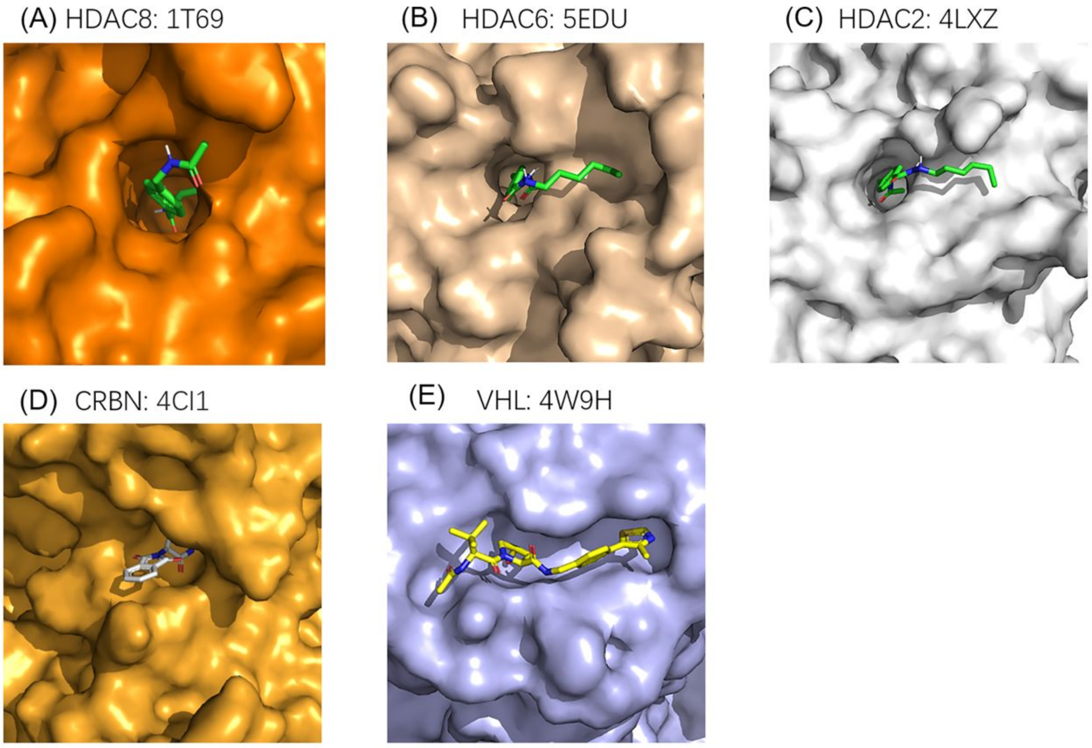

Figure S1. The proposed binding modes of compound **6** in HDAC8 (**A**, PDB Code:

1T69), HDAC6 (**B**, PDB Code: 5EDU), and HDAC2 (**C**, PDB Code: 4LXZ). The co-crystal complexes of thalidomide in cereblon (CRBN) (**D**, PDB Code: 4CL1) and the VHL ligand/VHL E3 ligase (**E**, PDB Code: 4W9H).

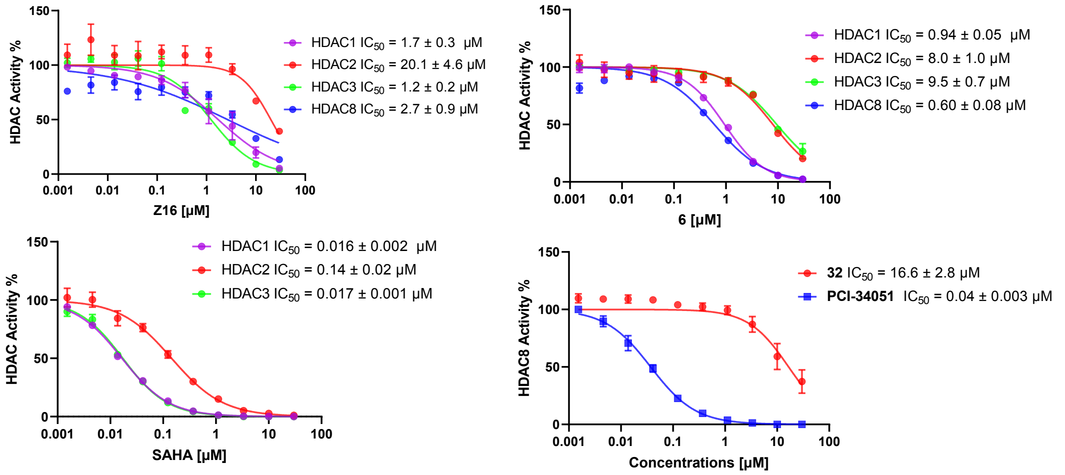

Figure S2. The in vitro HDAC1/2/3/8 deacetylase inhibitory activity of **Z16**. Nonlinear fitting was generated by GraphPad Prism and data were shown as mean ± SEM of three independent experiments.

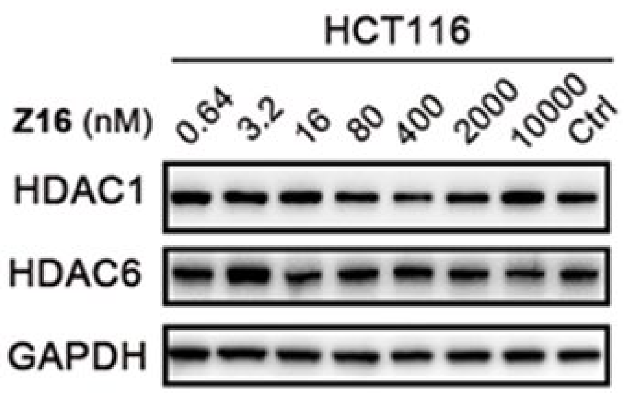

Figure S3. The effect of **Z16** on HDAC1 and HDAC6 levels in HCT116 cells. HCT116 cells were treated with **Z16** for 6 h. HDAC1 and HDAC6 levels were detected using western blot. GAPDH was used as a loading control.

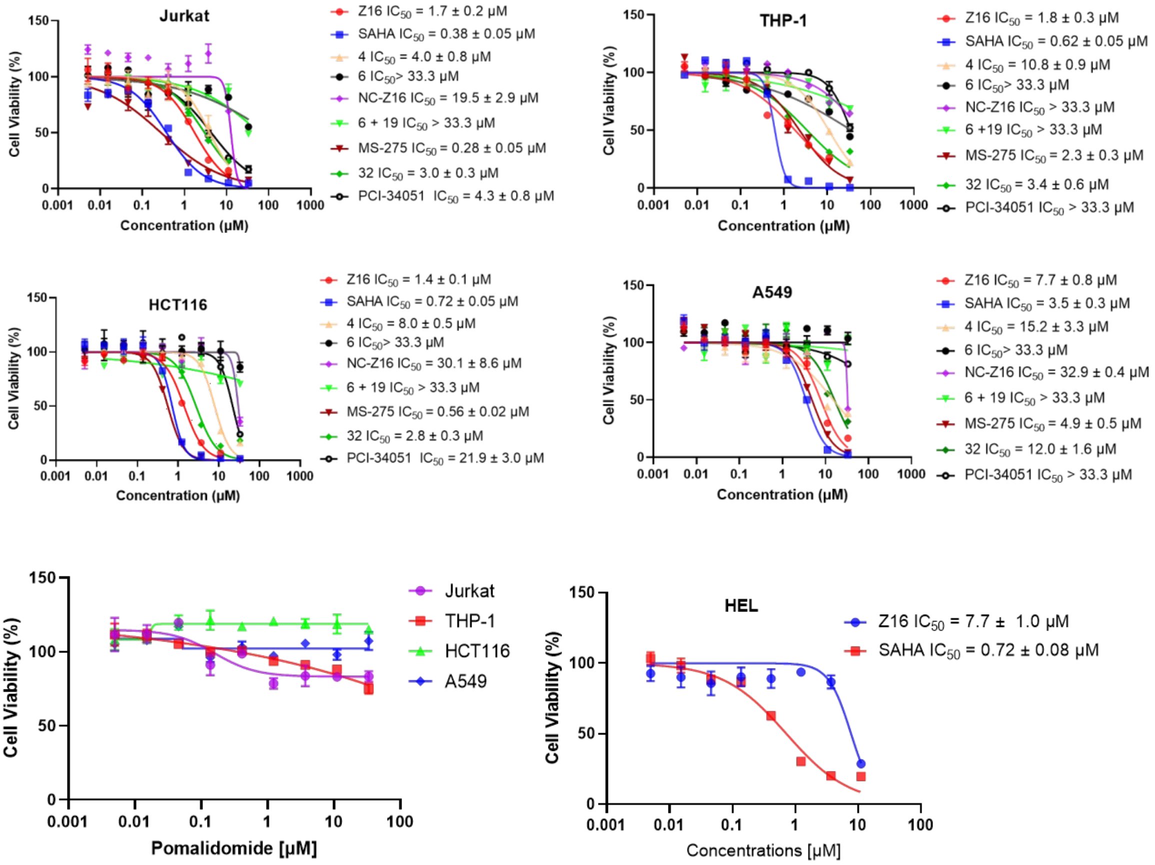

Figure S4. The antiproliferative activities against Jurkat, THP-1, HCT116, A549, and HEL cells. Cells were treated with indicated compounds for 72 h. Cell viability were determined by MTS assay. Nonlinear fitting was generated by GraphPad Prism and data were shown as mean ± SEM of three independent experiments.

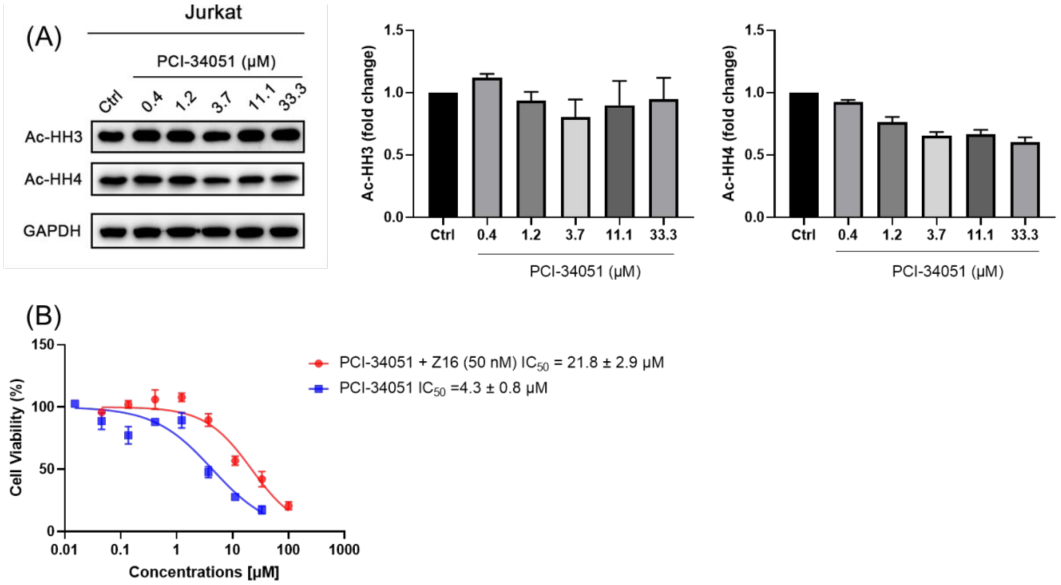

Figure S5. (A) Jurkat cells were treated with indicated concentrations of PCI-34051 for 24 h. The levels of Ac-HH3 and Ac-HH4 were detected by western blot. GAPDH

was used as a loading control. The levels of Ac-HH3 and Ac-HH4 were quantified using Image J and data are shown as mean ± SEM of two independent experiments. (B) Jurkat cells were either pre-treated with 50 nM **Z16** for 2h, followed by treatment with PCI-34051 for 72 h or treated with PCI-34051 for 72 h. ell viability were determined by MTS assay. Nonlinear fitting was generated by GraphPad Prism and data were shown as mean ± SEM of three independent experiments.

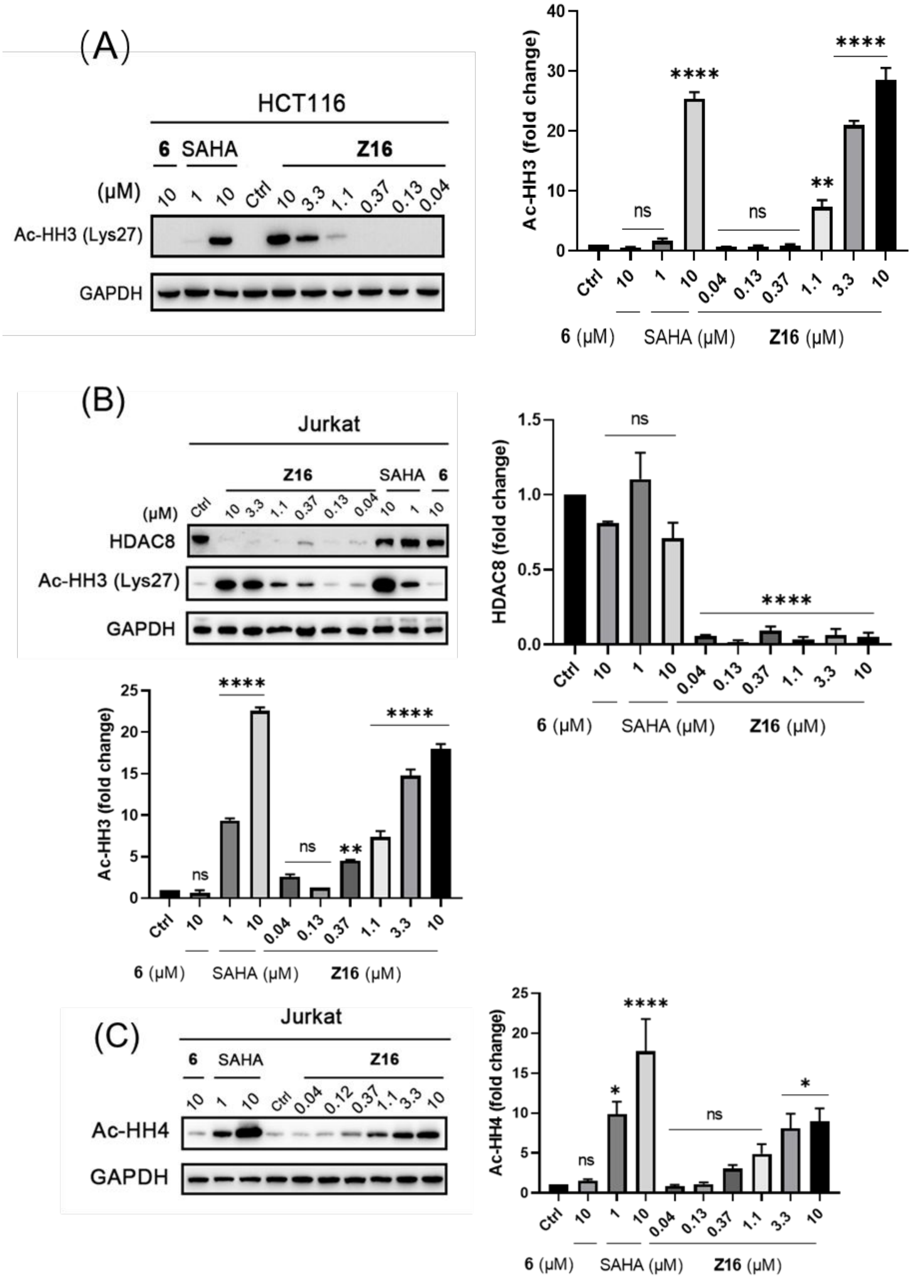

Figure S6. (A) HCT116 cells were treated with indicated concentrations of **Z16**, **6** and SAHA for 24 h. (B) and (C) Jurkat cells were treated with indicated concentrations of **Z16**, **6** and SAHA for 48 h. The levels of HDAC8, Ac-HH3, and Ac-HH4 were detected using western blot. GAPDH was used as a loading control. Data were normalized to the DMSO-treated group and the dot plots were shown as mean ± SEM of at least two independent experiments. The levels Ac-HH3 and Ac-HH4 were quantified using Image J and data are shown as mean ± SEM of two independent experiments. ns: not significant, *P<0.05, **P<0.01, ***P<0.001, and ****P<0.0001 vs DMSO-treated group, one-way analysis of variance (ANOVA).

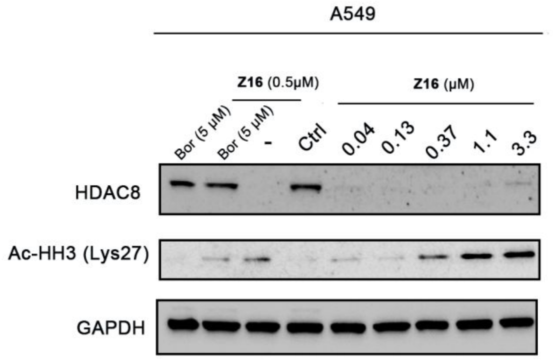

Figure S7. A549 cells were either pre-treated with 5 μM bortezomib (Bor) for 1 h, followed by treatment with 0.5 μM **Z16** or treated with indicated concentration of **Z16** for 6 h. The levels of Ac-HH3 and HDAC8 were detected using western blot. GAPDH was used as a loading control.

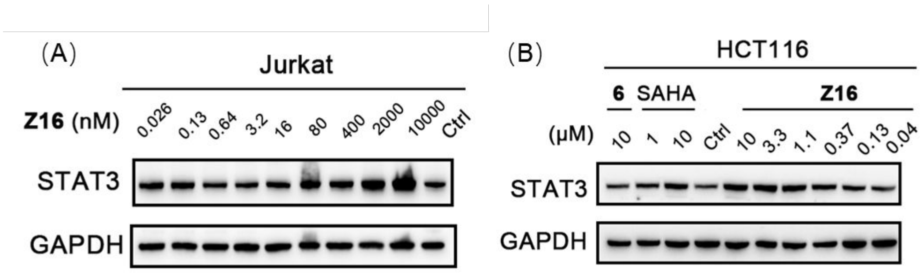

Figure S8. The effect of **Z16** on STAT3 level in Jurkat and HCT116 cells. (A) Jurkat and (B) HCT116 cells were treated with indicated compounds for 24 h. STAT3 level was detected using western blot. GAPDH was used as a loading control.

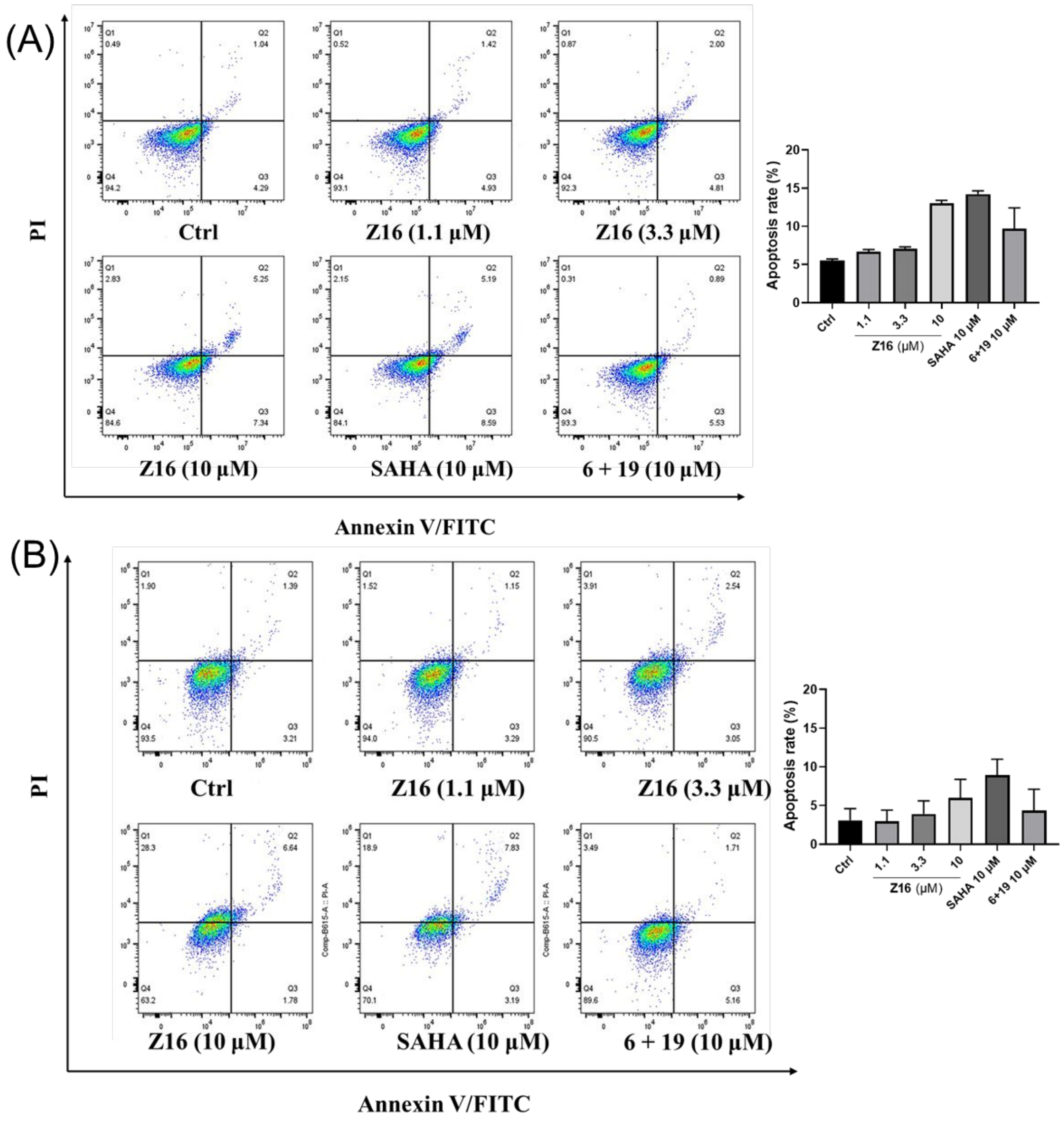

Figure S9. Jurkat cells were treated with the indicated concentrations of **Z16**, **6+19**, and SAHA for 24 h (A) and 48 h (B). Apoptosis was determined using flow cytometry. Data are shown as mean ± SEM of two independent experiments.

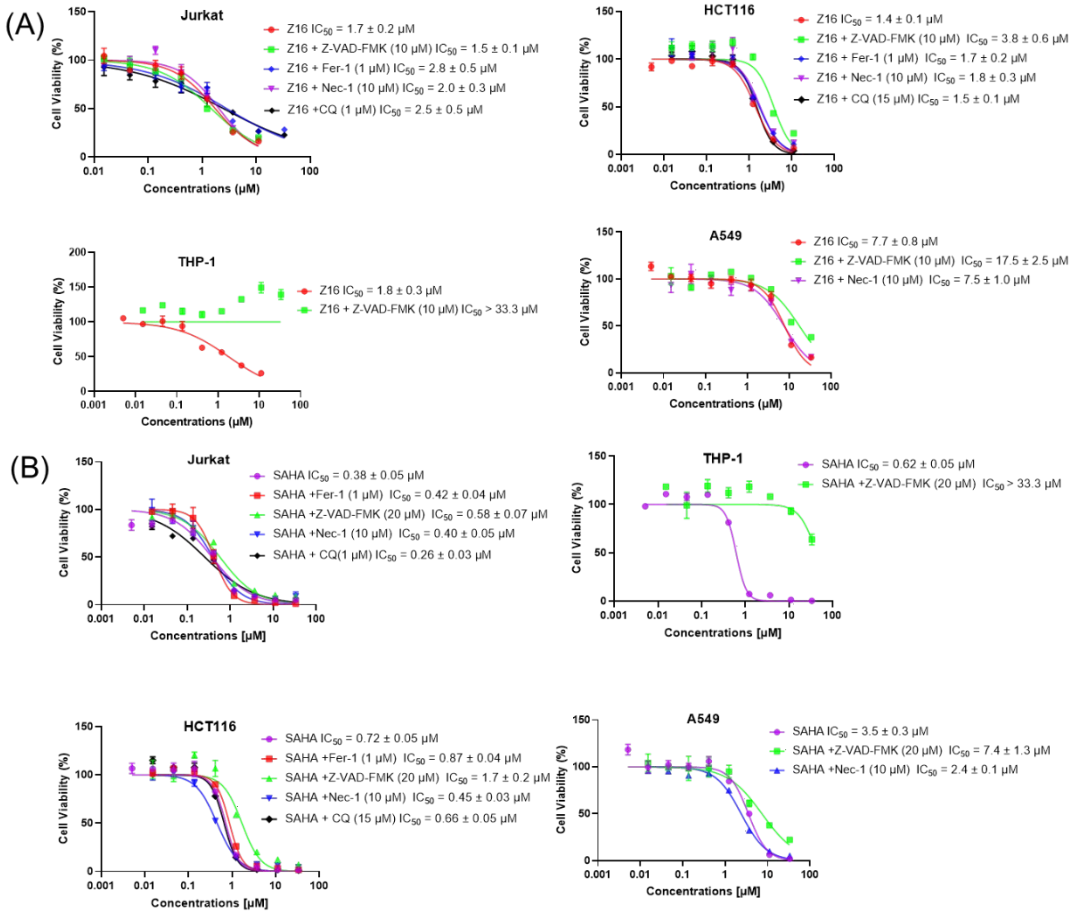

Figure S10. Cells were pre-treated with 20 μM of caspase inhibitor Z-VAD-FMK, 10 μM of necroptosis inhibitor necrostatin-1 (Nec-1) and 1 μM of ferroptosis inhibitor ferrostain-1 (Fer-1), 1μM of CQ for Jurkat and 15 μM of CQ for HCT116 for 1 h, followed by treatment of **Z16** (A) and SAHA (B) for 72 h. Data are shown as mean ± SEM of three independent experiments.

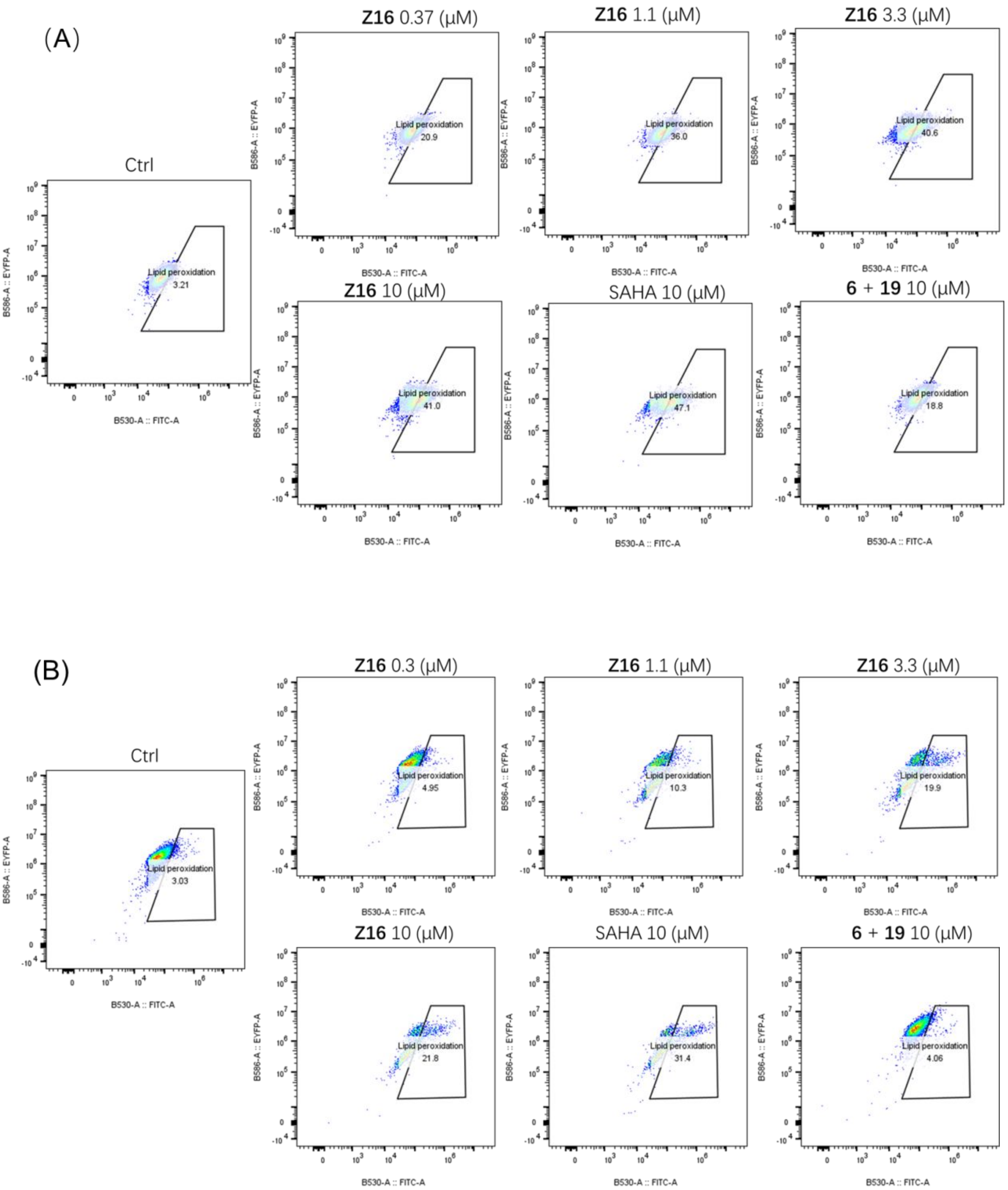
(A)
Ctrl
Lipid peroxidation
3.21
Z16 0.37 (μM)
20.9
Z16 1.1 (μM)
36.0
Z16 3.3 (μM)
40.6
Z16 10 (μM)
41.0
SAHA 10 (μM)
47.1
6 + 19 10 (μM)
18.8
(B)
Ctrl
3.03
Z16 0.3 (μM)
4.95
Z16 1.1 (μM)
10.3
Z16 3.3 (μM)
19.9
Z16 10 (μM)
21.8
SAHA 10 (μM)
31.4
6 + 19 10 (μM)
4.06
B530-A :: FITC-A
B586-A :: EYFP-A

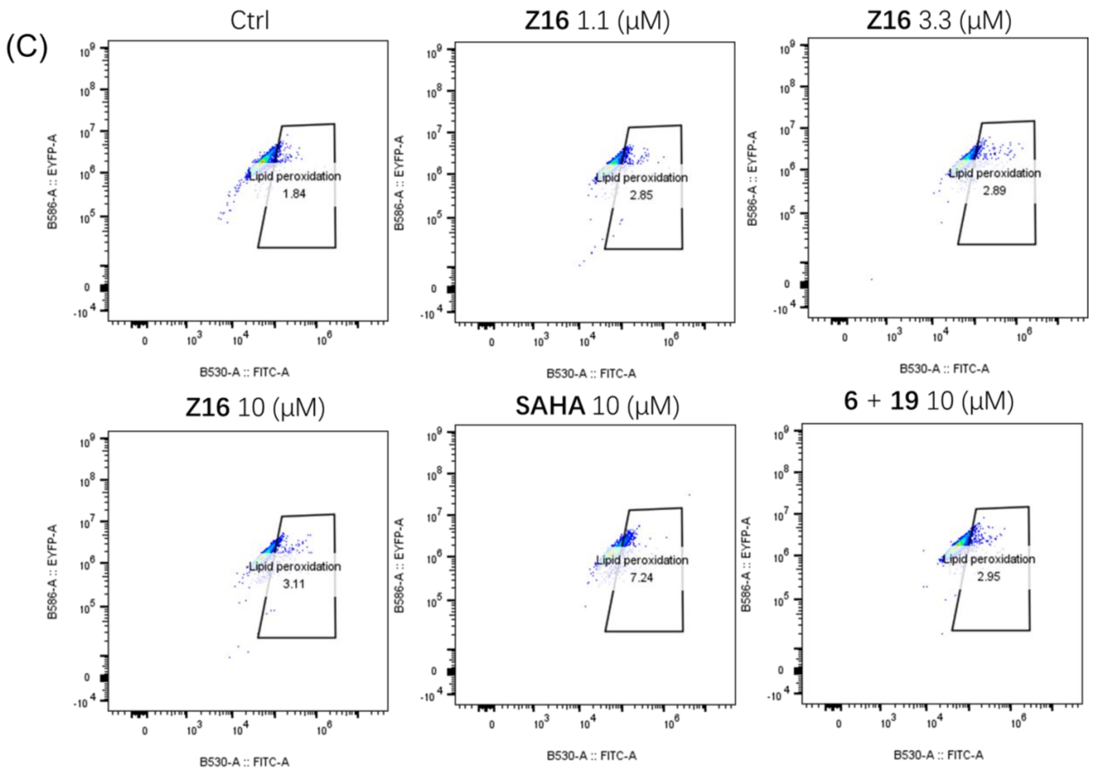

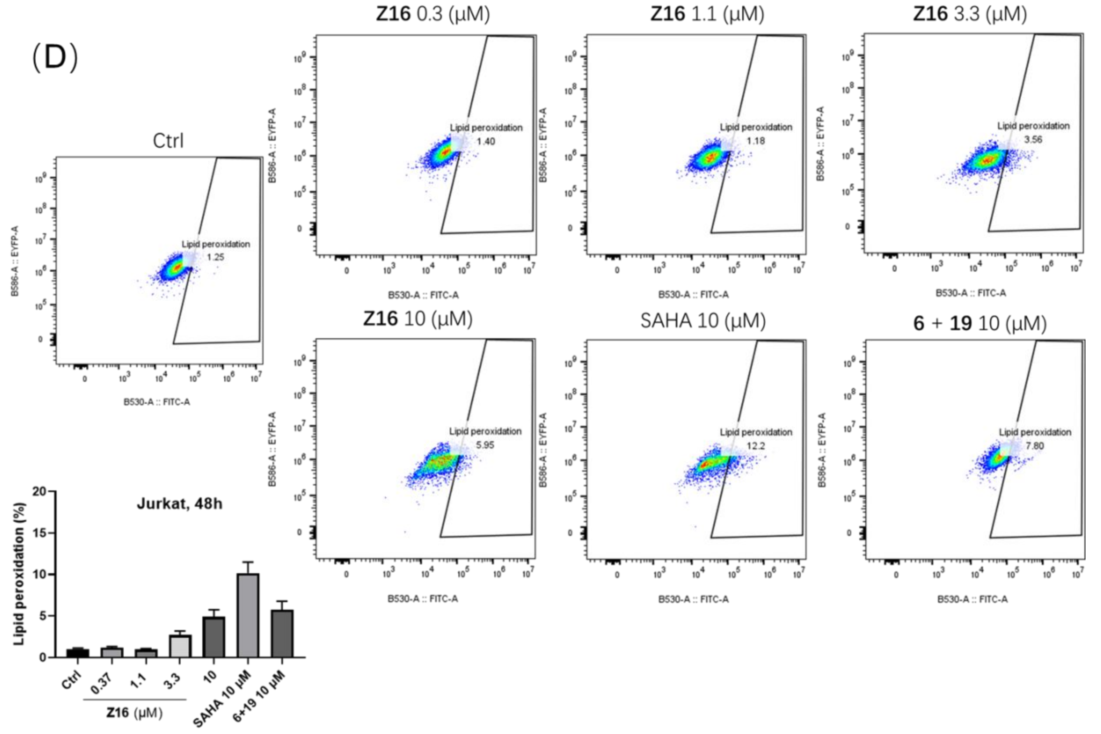

Figure S11. Jurkat (A), HCT116 (B), and THP-1(C) were treated with indicated compounds for 24 h. Jurkat (D) were treated with indicated compounds for 48 h. The lipid peroxidation level was detected by BODIPY 581/591 C11 staining determined by flow cytometry. Data are shown as mean ± SEM of two independent experiment.

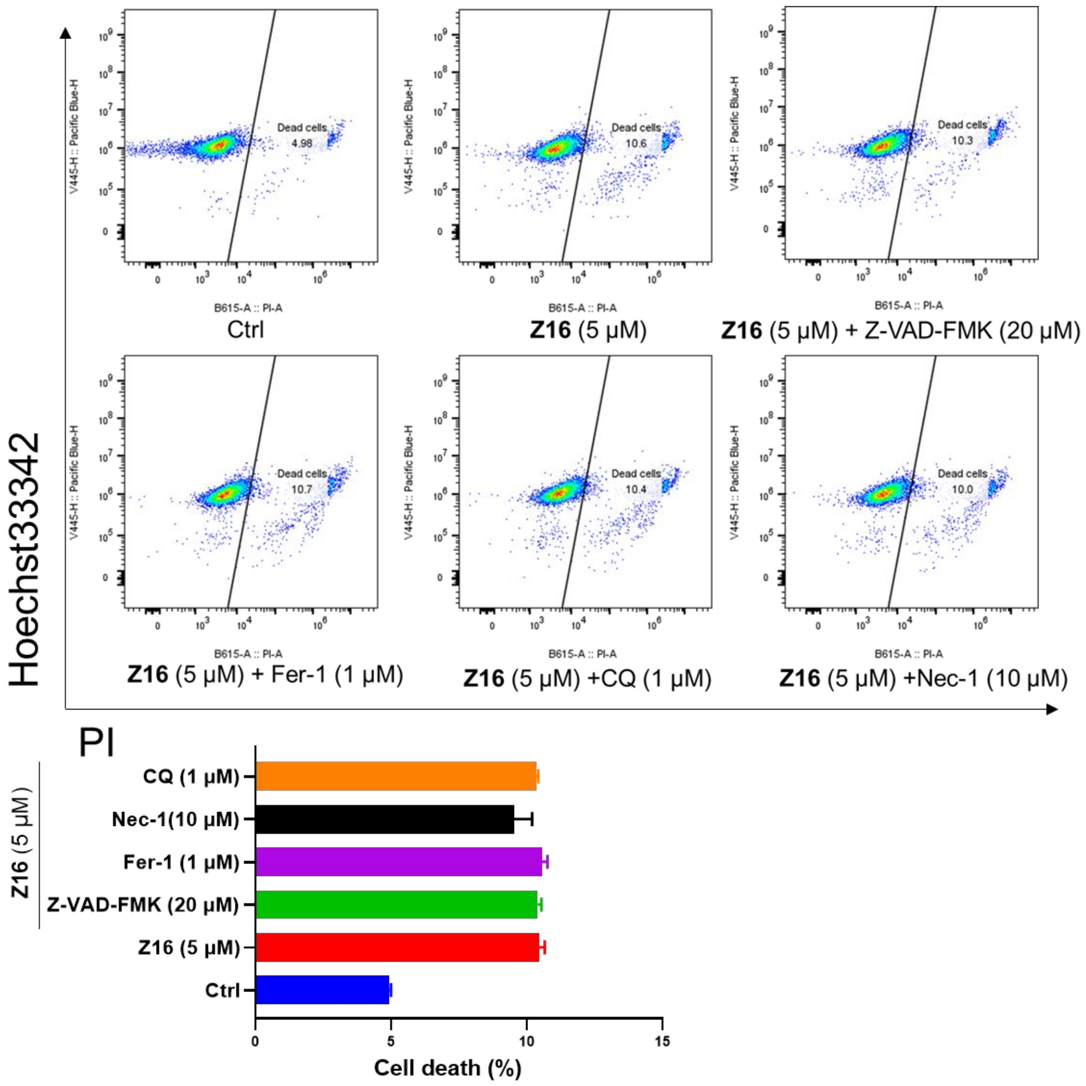

Figure S12. Jurkat cells were either pre-treated with indicated concentrations of cell death inhibitors for 1h, folowed by 5 μM **Z16** treatment for 48 or treated with 5 μM **Z16** for 48 h. Cell death was detected by flow cytometry. Cell death rates are shown as mean ± SEM of two independent experiment.

Uncropped western blots

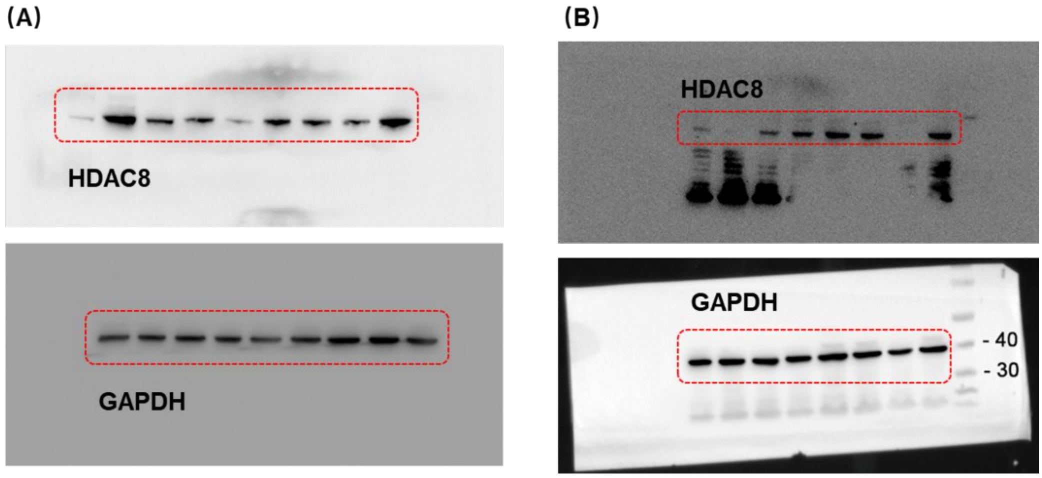

Uncropped western blot for **Figure 3**

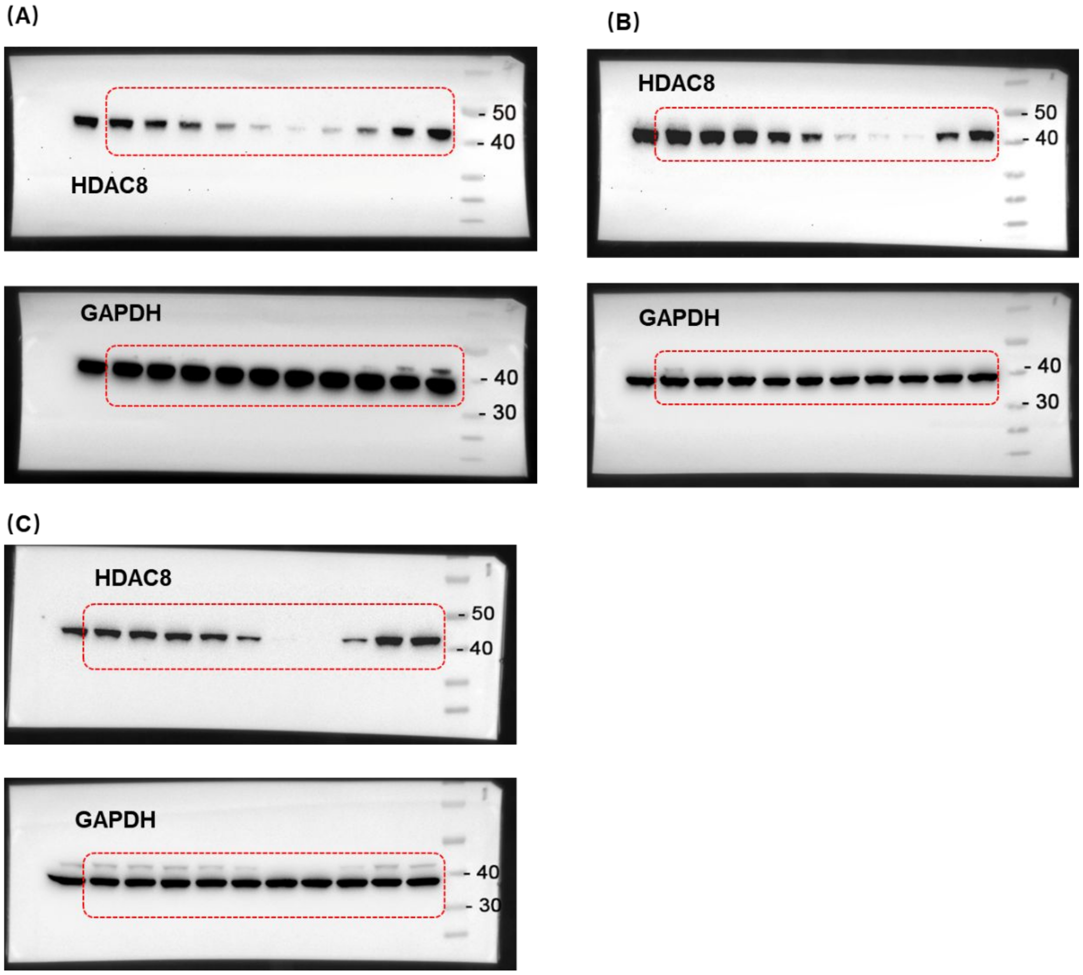

Uncropped western blot for **Figure 4**

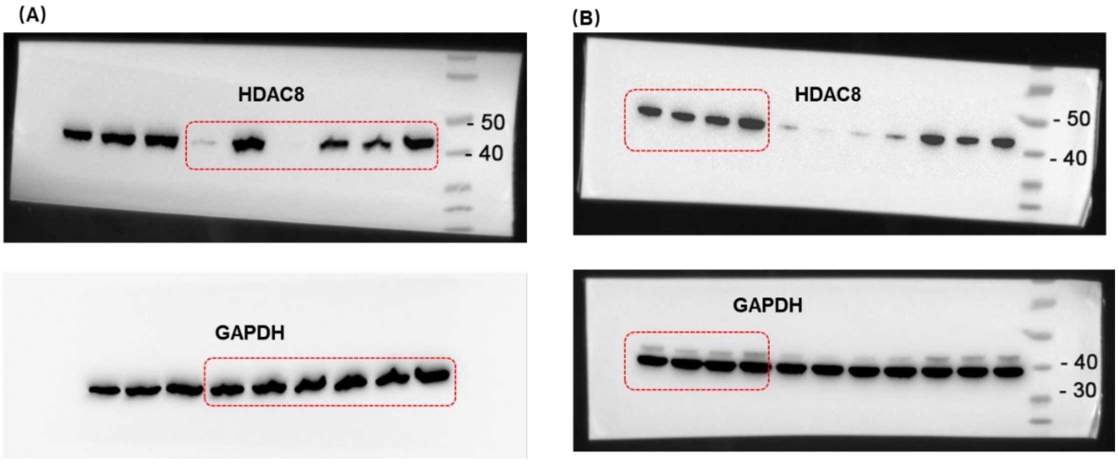

Uncropped western blot for **Figure 5**

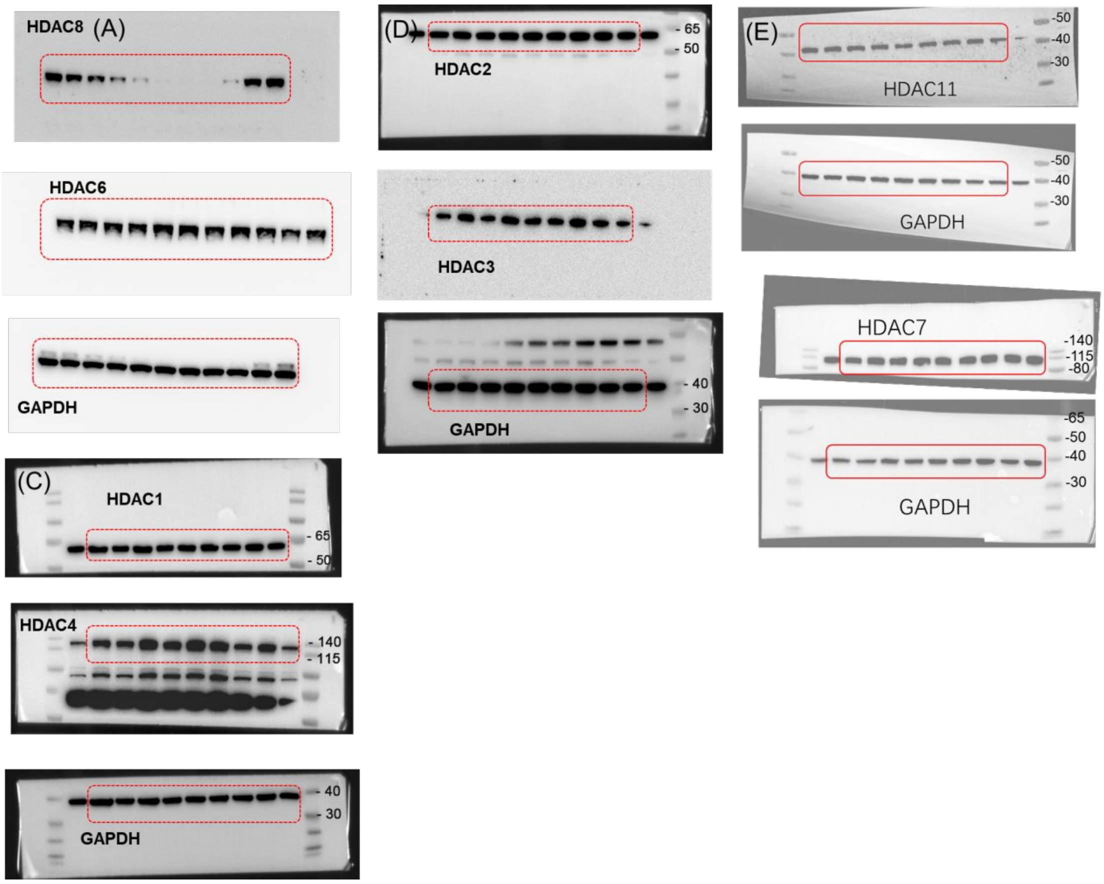

Uncropped western blot for **Figure 6**

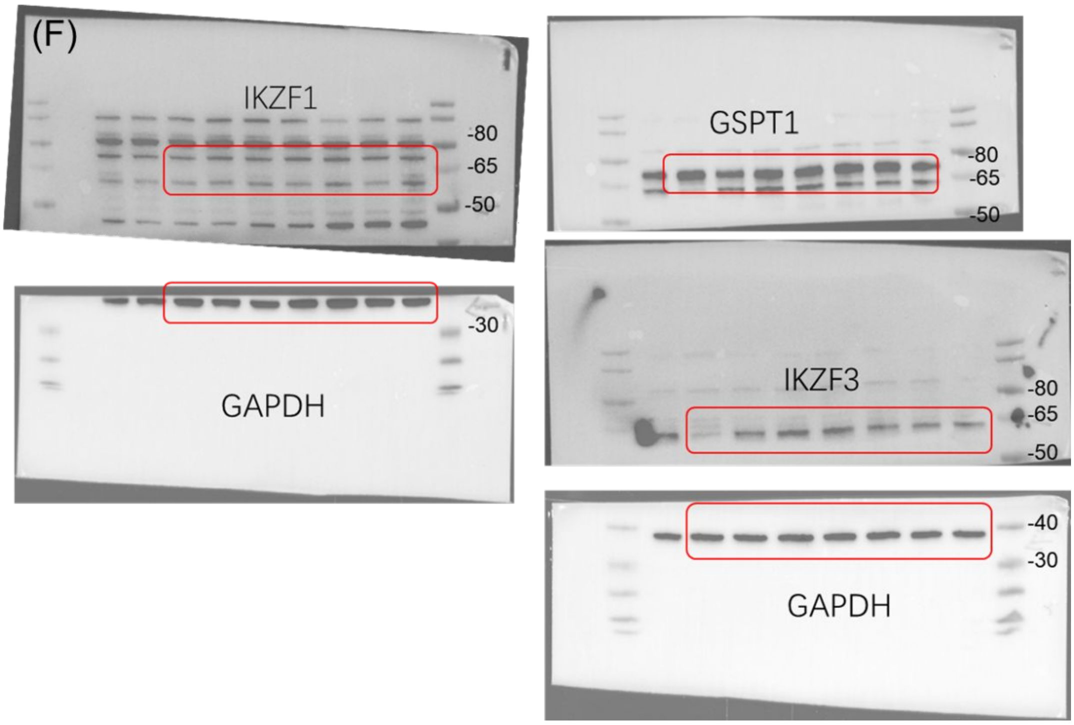

Uncropped western blot for **Figure 6**

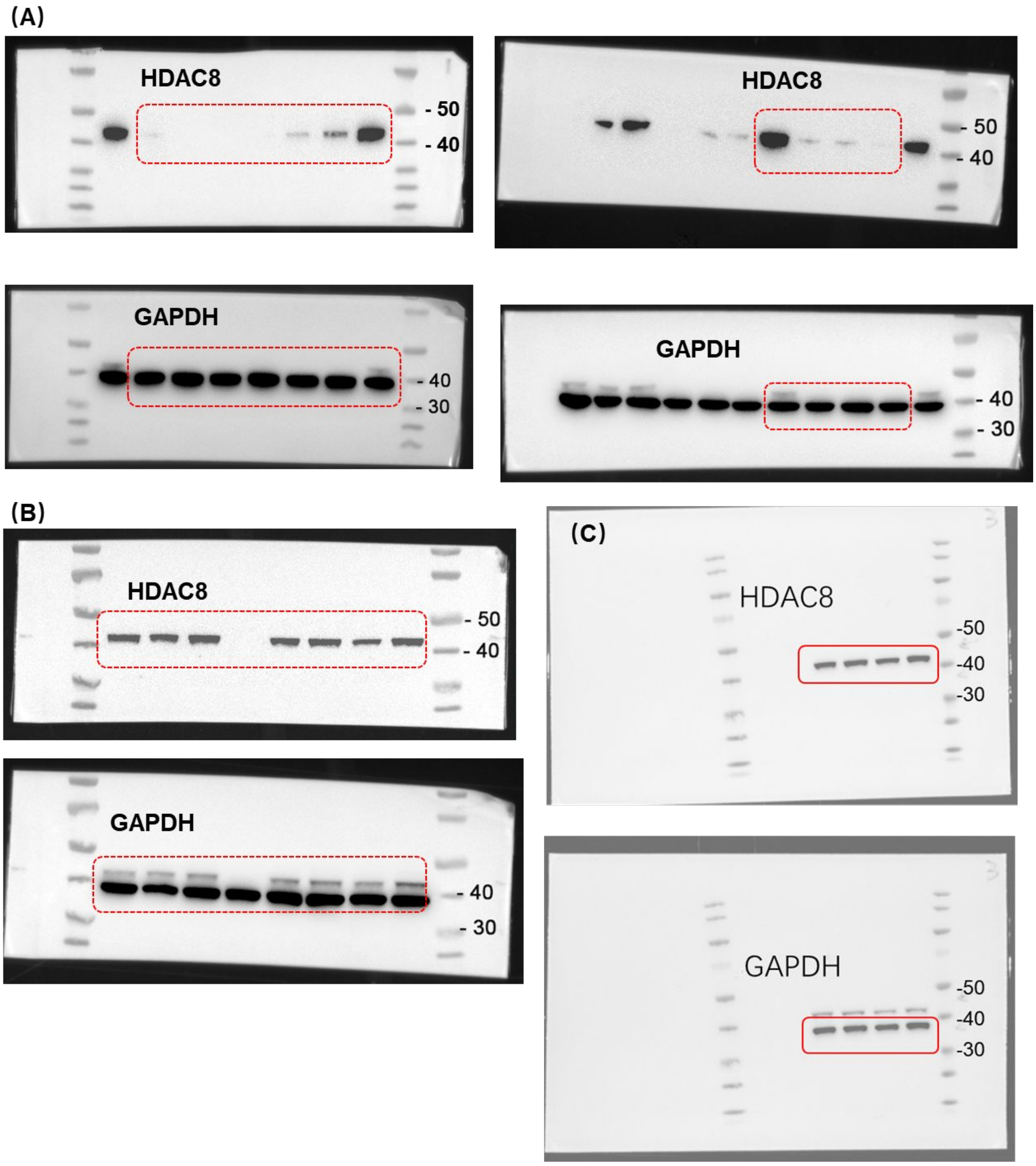

Uncropped western blot for **Figure 7**

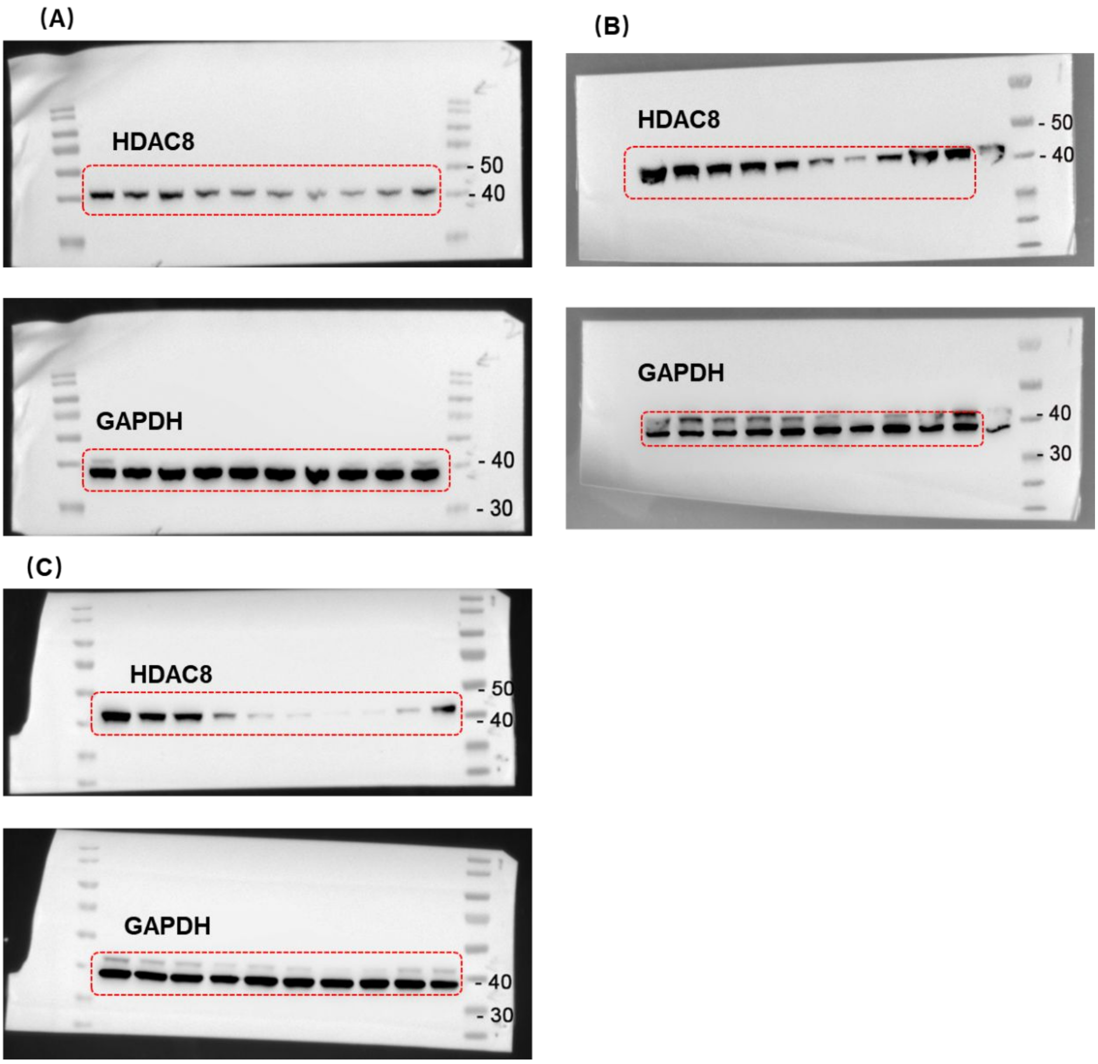

Uncropped western blot for **Figure 8**

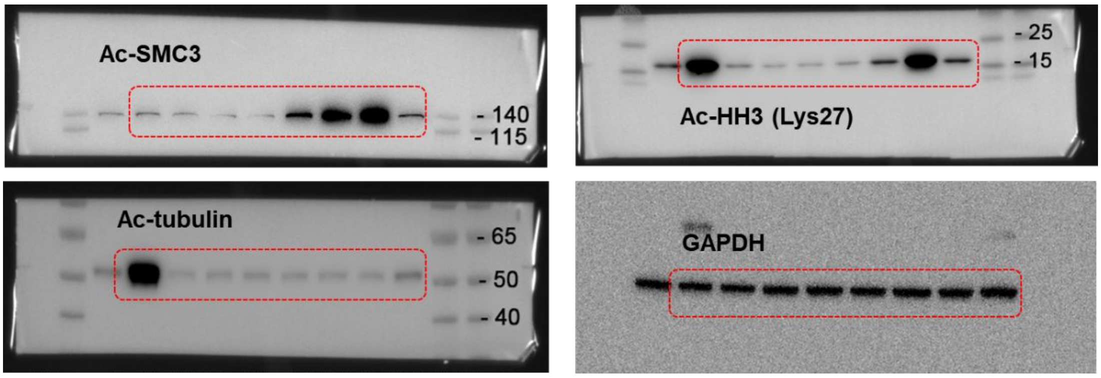

Uncropped western blot for **Figure 9**

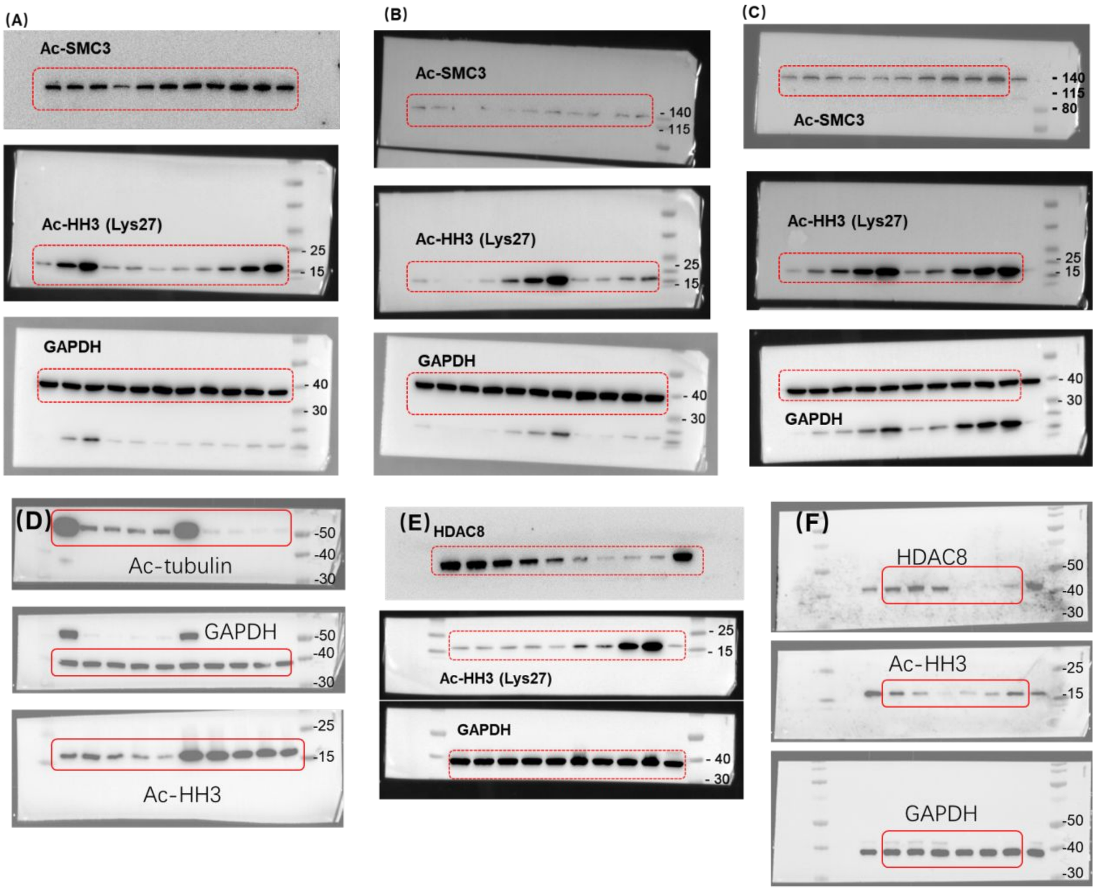

Uncropped western blot for **Figure 10**

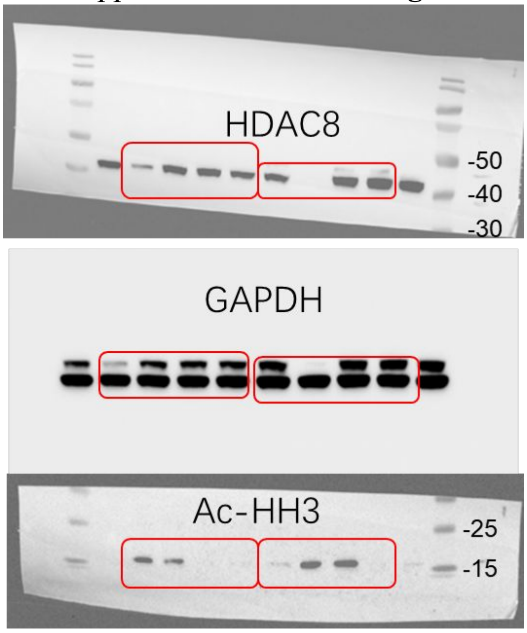

Uncropped western blot for **Figure 11**

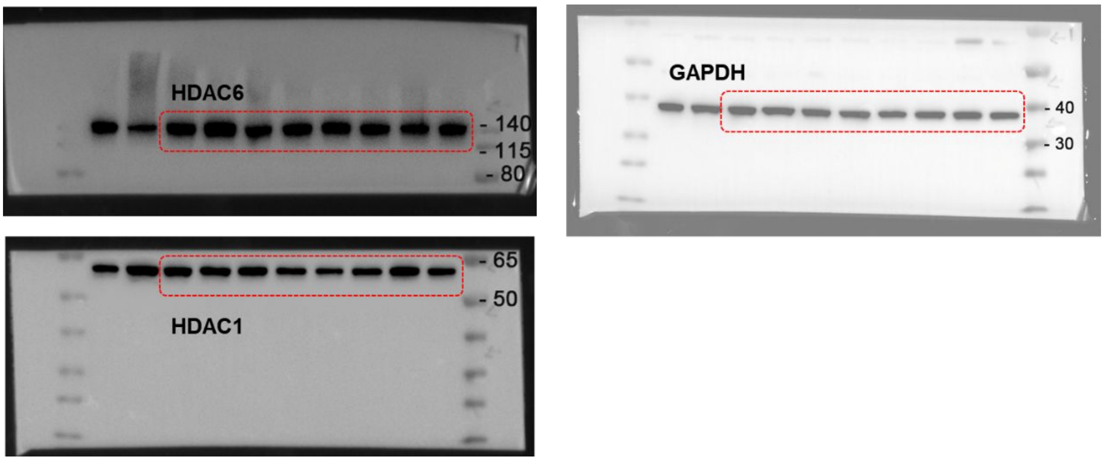

Uncropped western blot for **Figure S3**

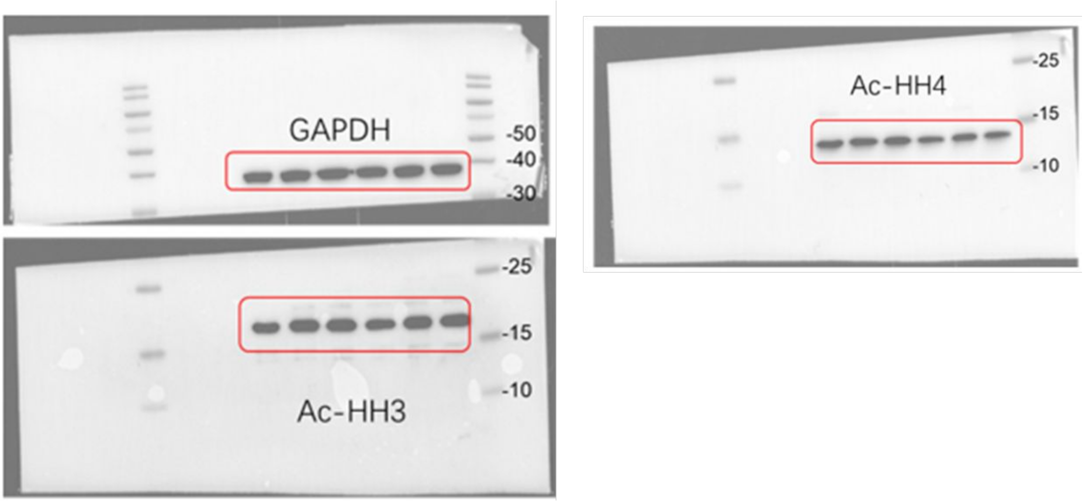

Uncropped western blot for Figure S5A

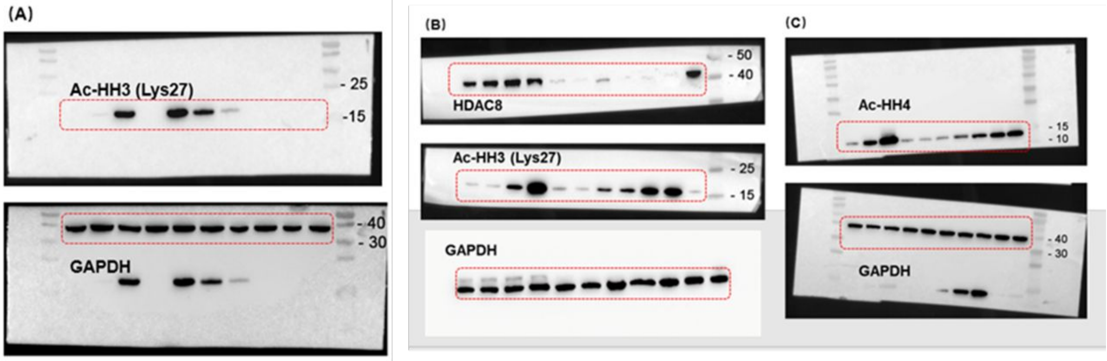

Uncropped western blot for **Figure S6**

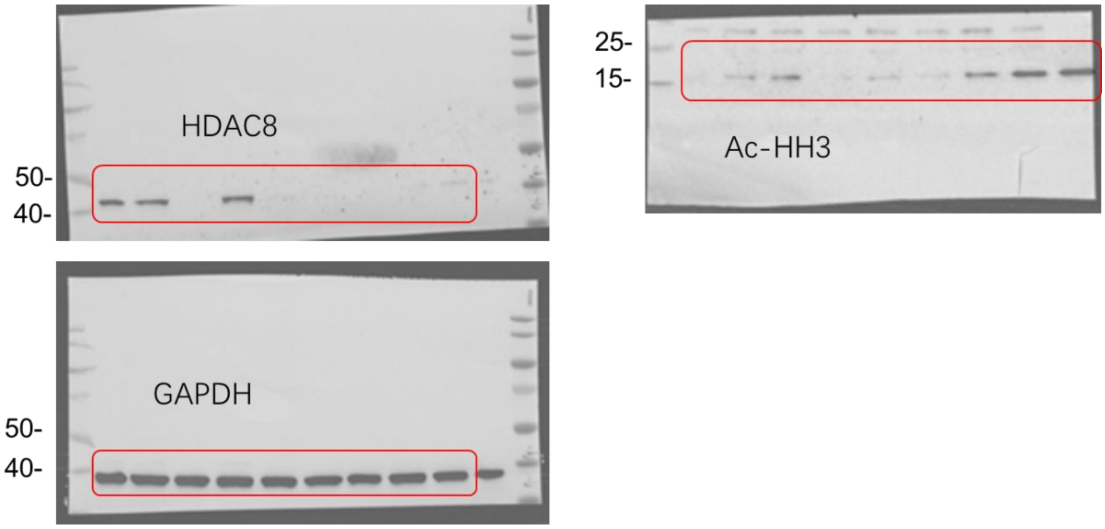

Uncropped western blot for **Figure S7**

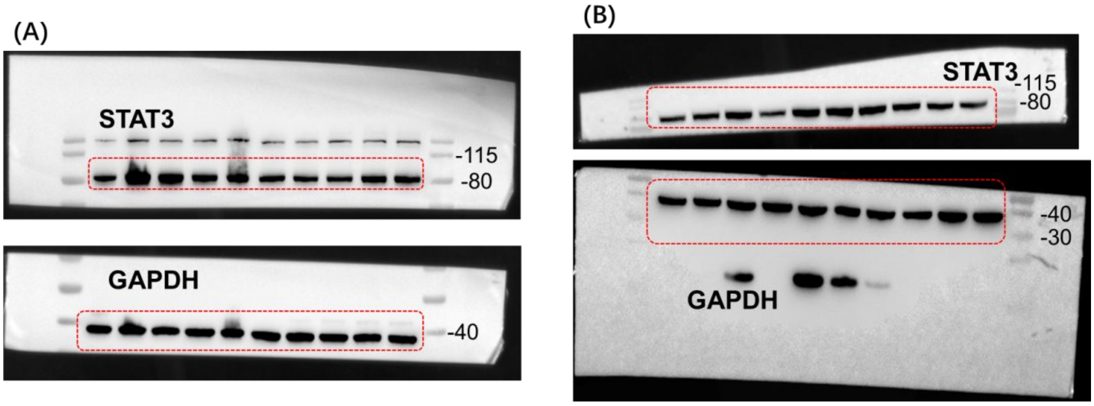

Uncropped western blot for **Figure S8**

NMR, HRMS and HPLC spectra
Representative NMR spectra

^{1}H NMR of **9** in DMSO-d_6

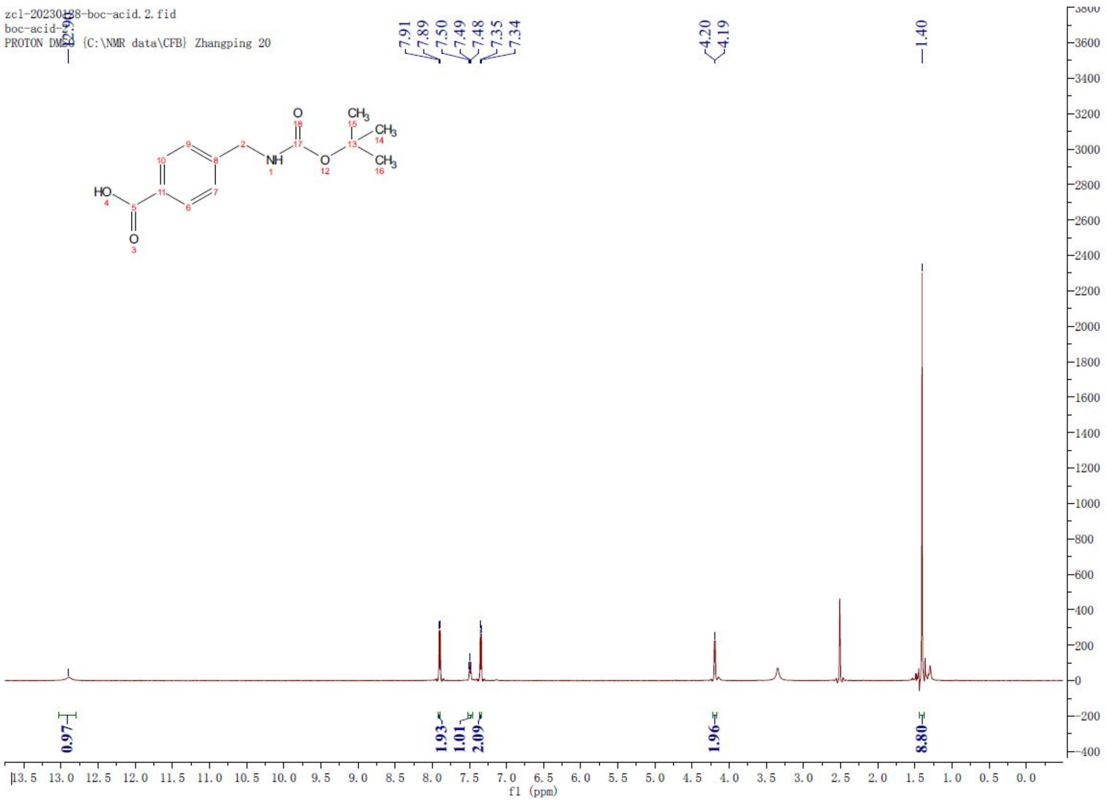

^{1}H NMR of **12** in DMSO-d_6

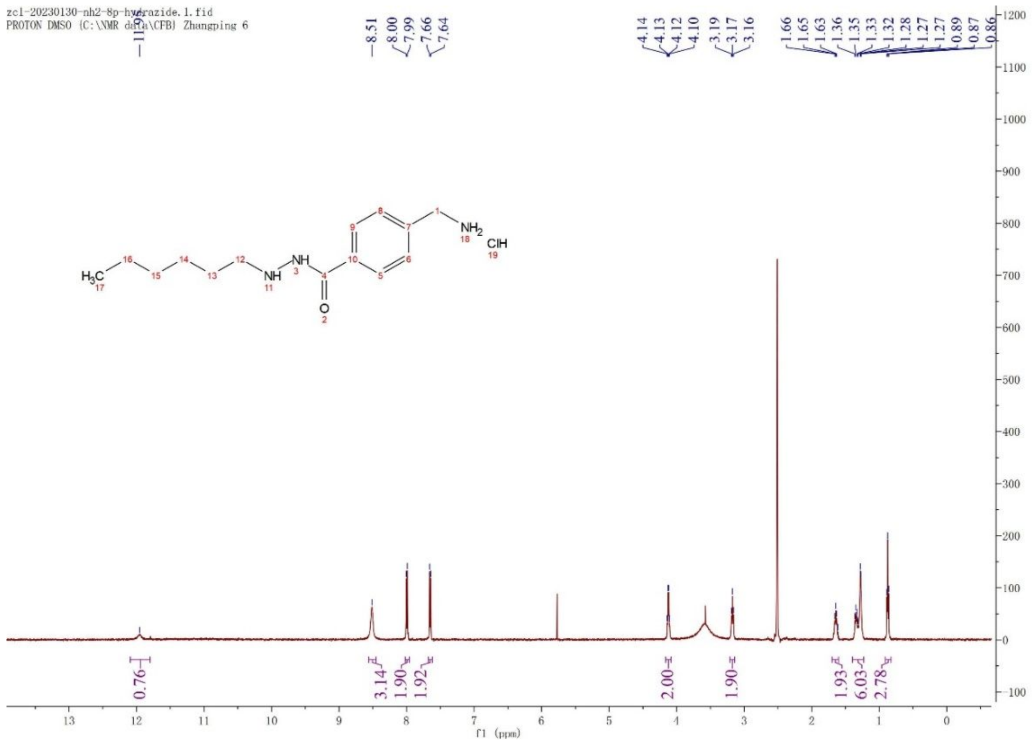

^{1}H NMR of **Z1** in DMSO-d_6

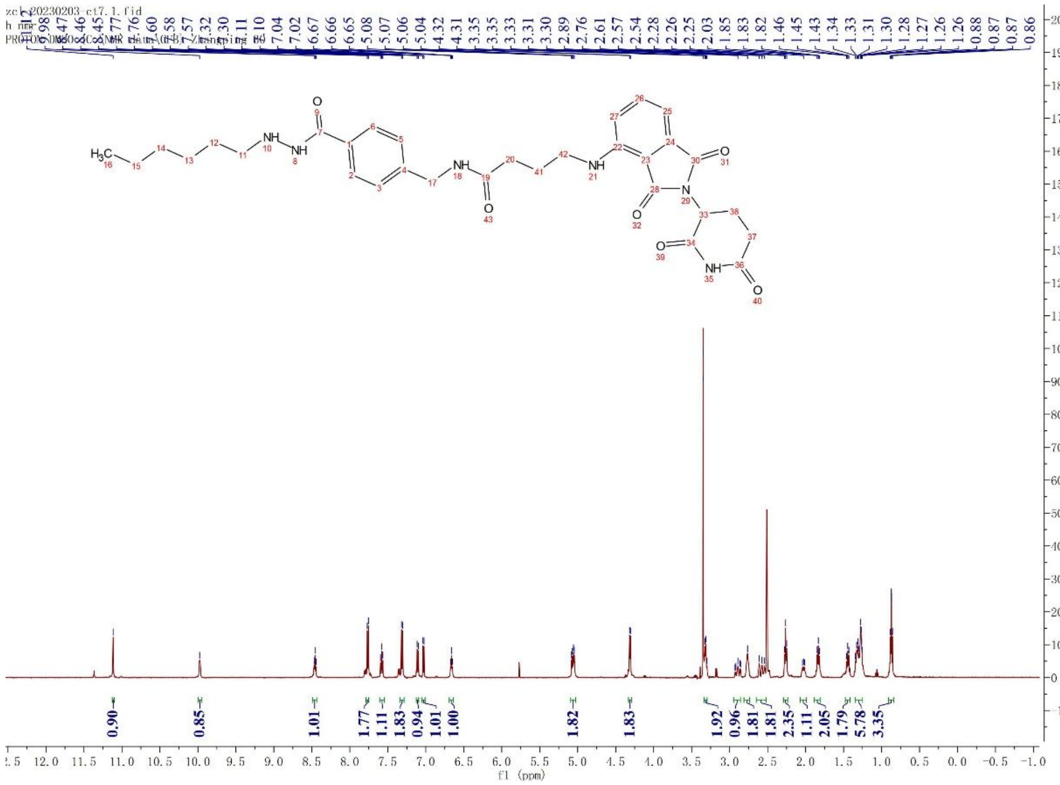

^{13}C NMR of **Z1** in DMSO-d_6

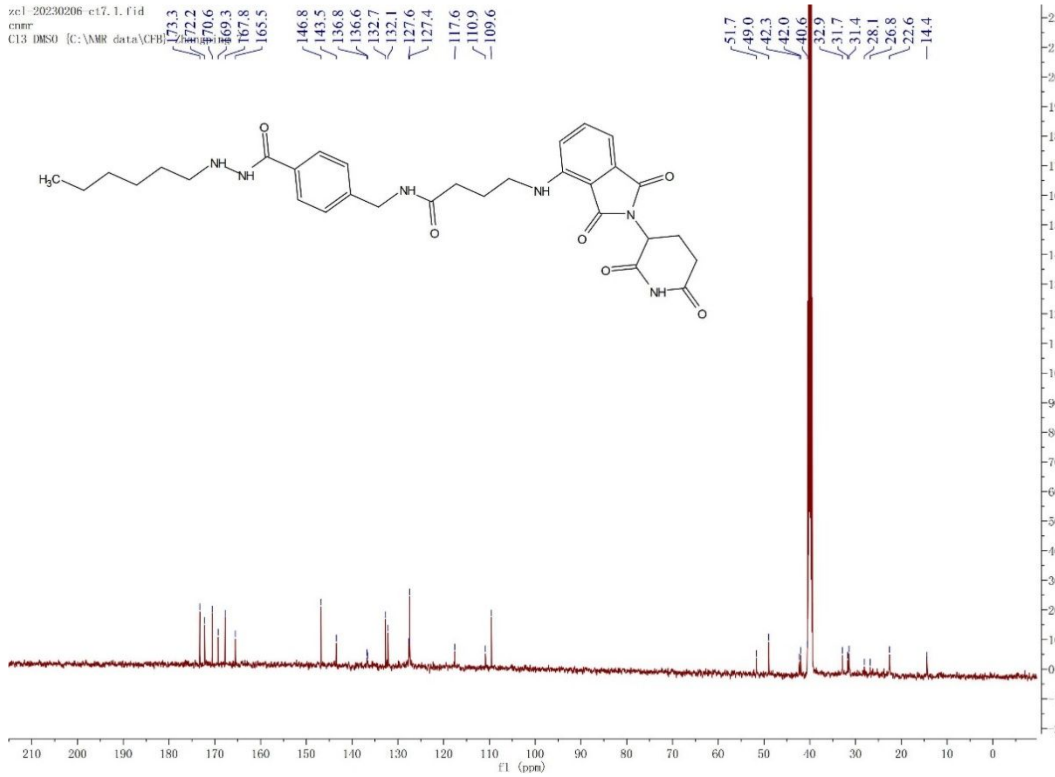

^{1}H NMR of **Z2** in DMSO-d_6

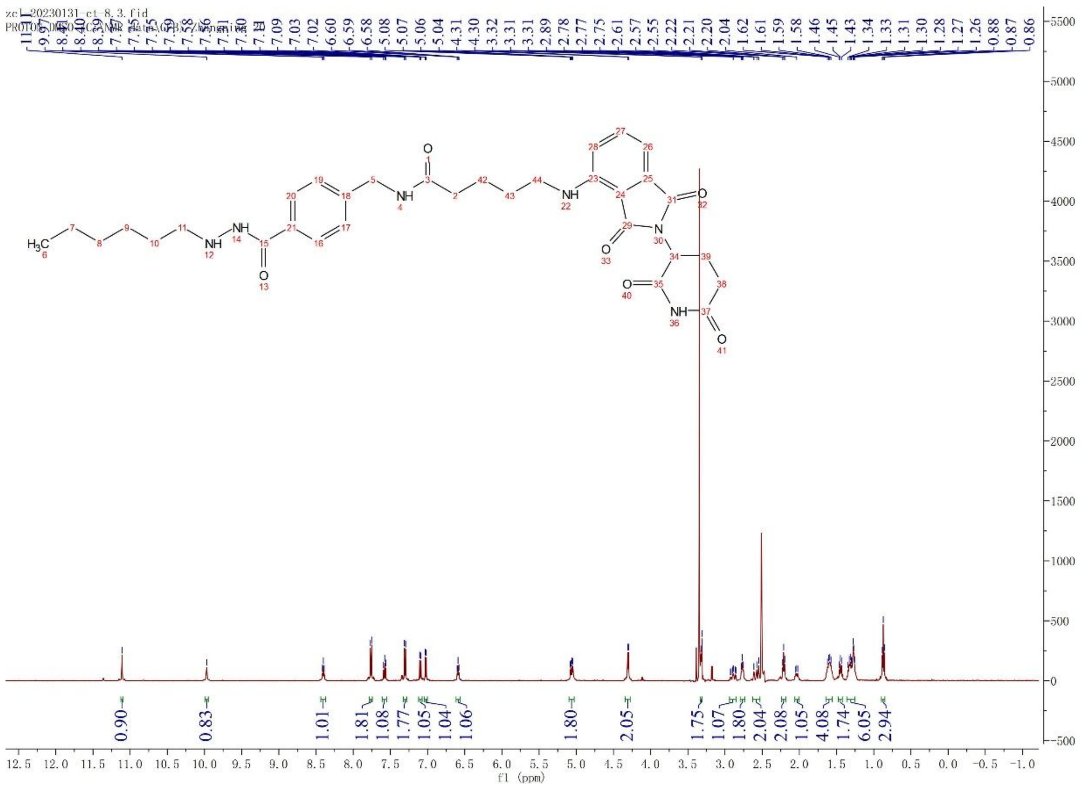

^{13}C NMR of **Z2** in DMSO-d_6

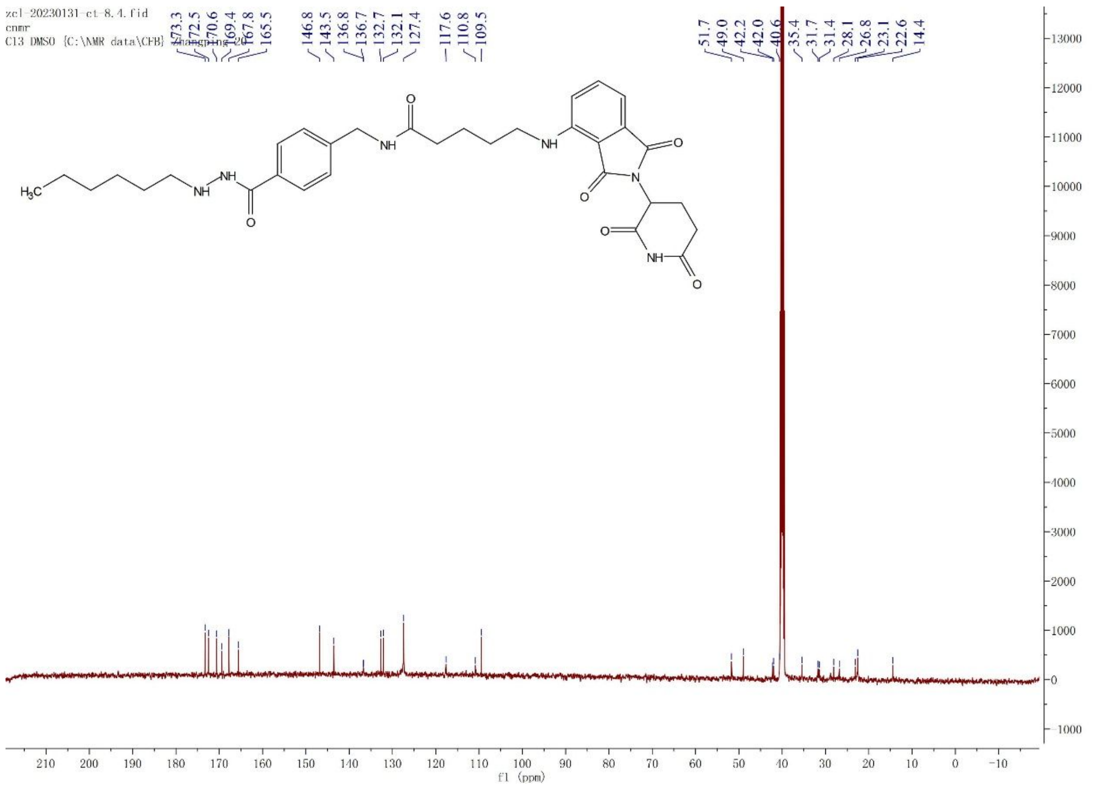

^{1}H NMR of **Z3** in DMSO-d_6

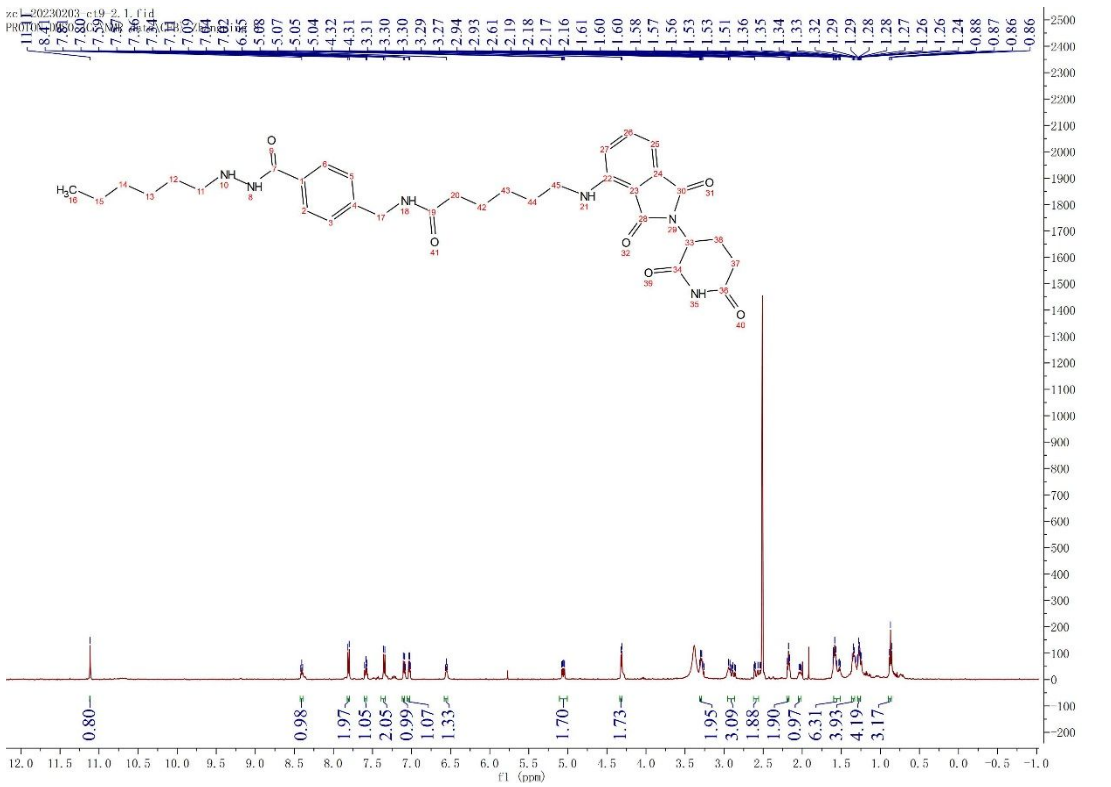

^{13}C NMR of **Z3** in DMSO-d_6

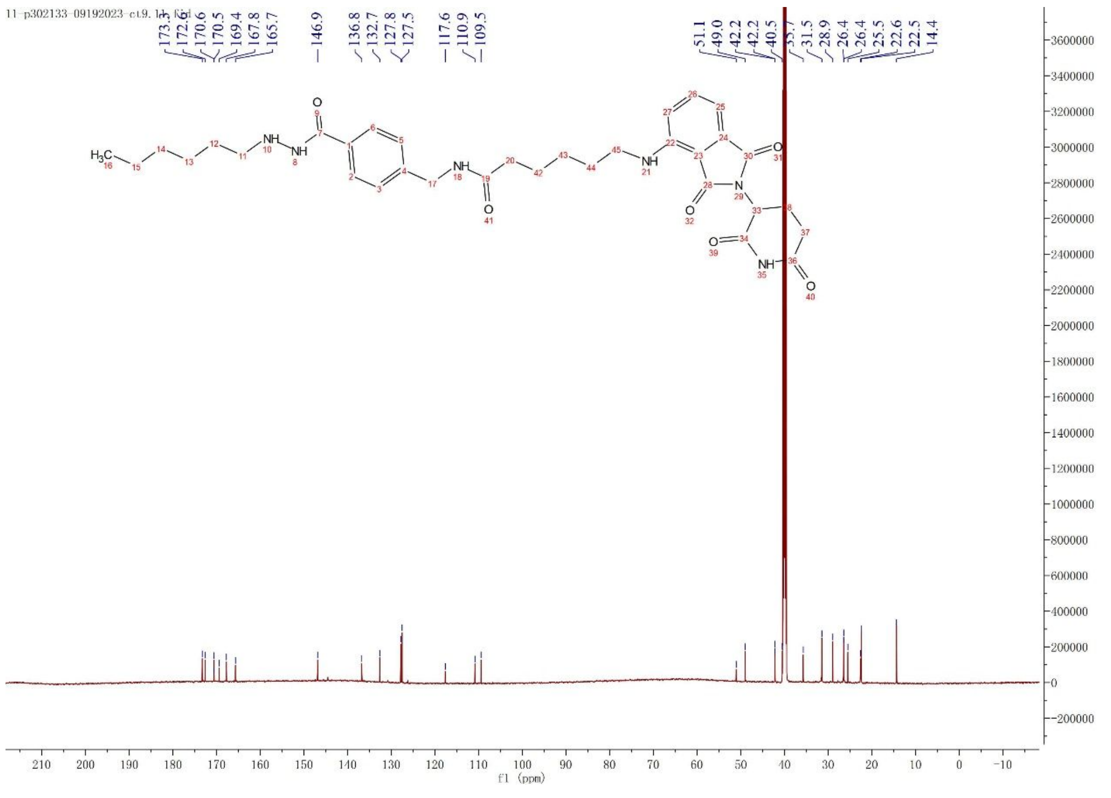

^{1}H NMR of **Z4** in DMSO-d_6

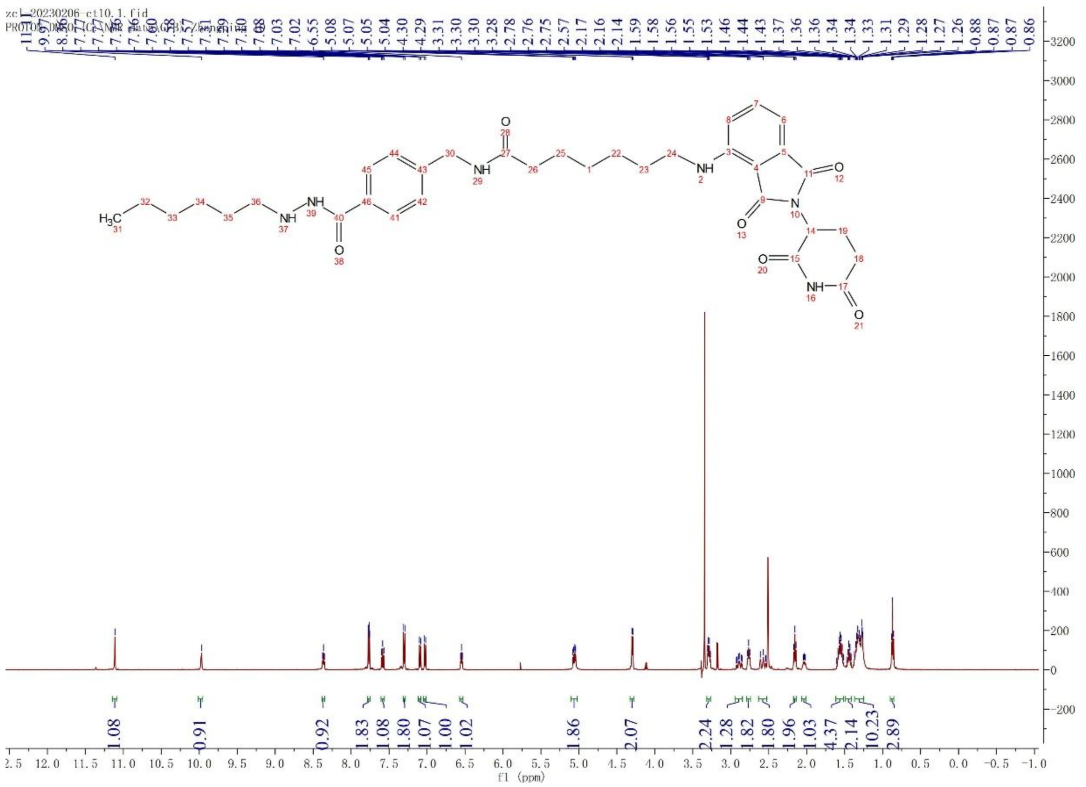

^{13}C NMR of **Z4** in DMSO-d_6

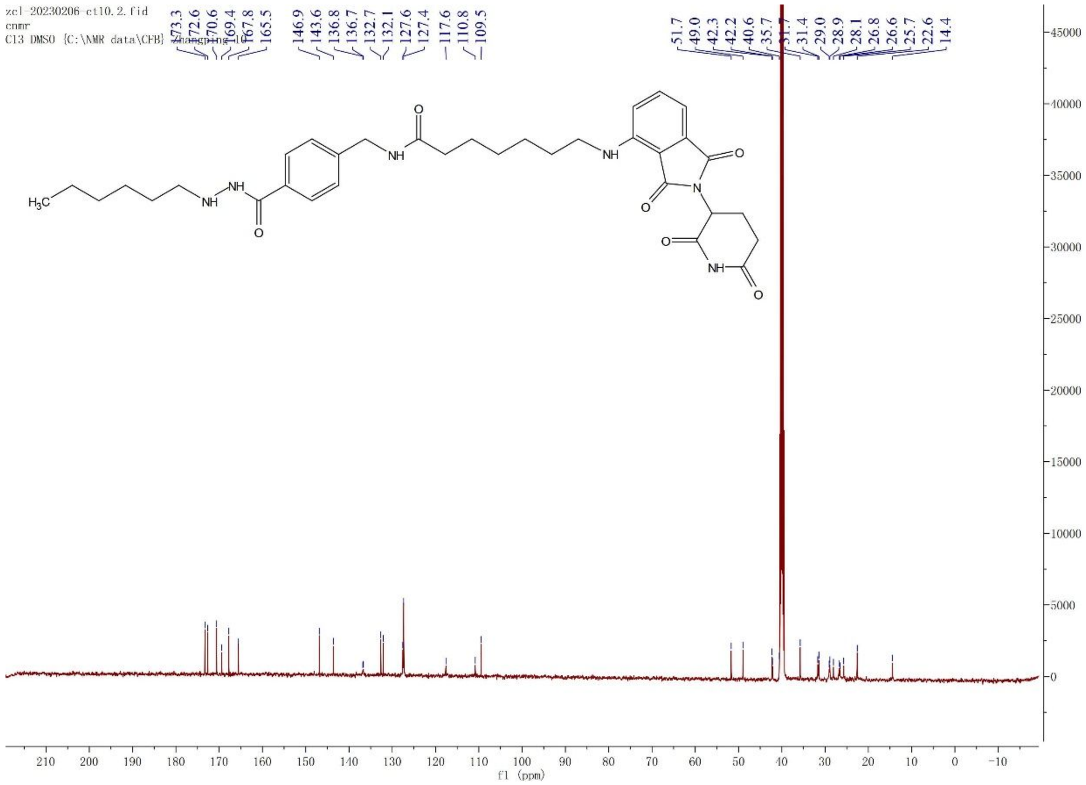

^{1}H NMR of **Z5** in DMSO-d_6

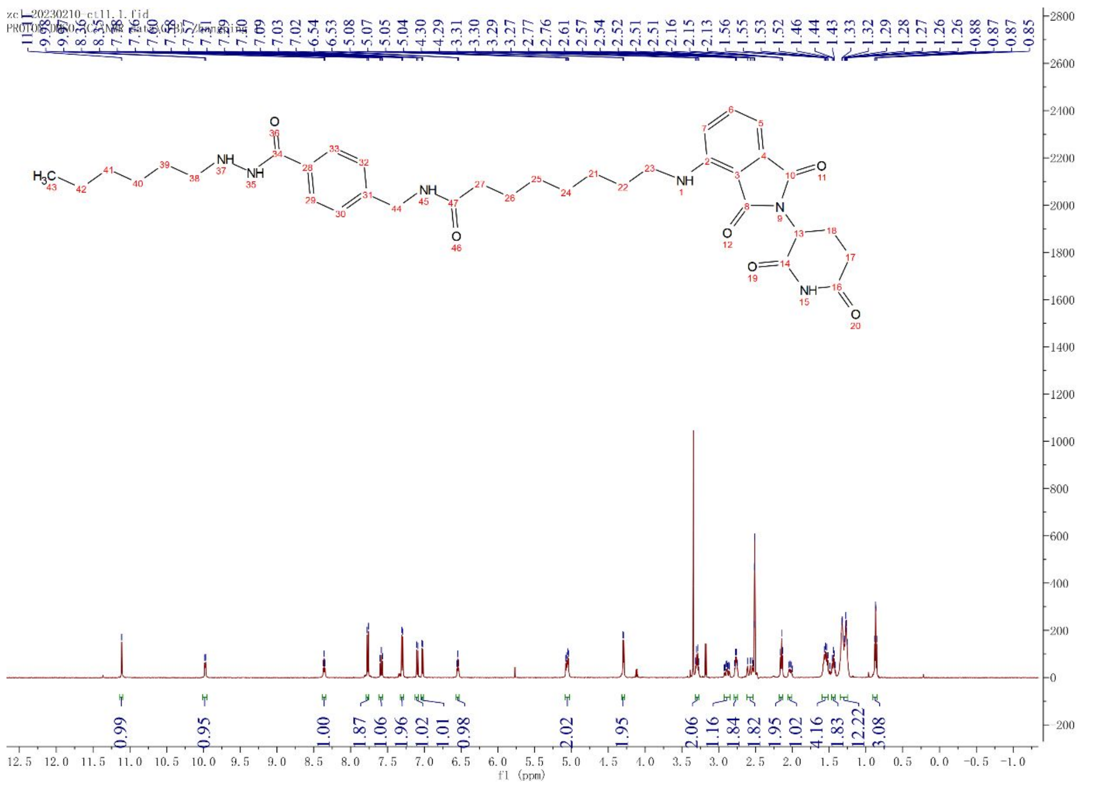

^{13}C NMR of **Z5** in DMSO-d_6

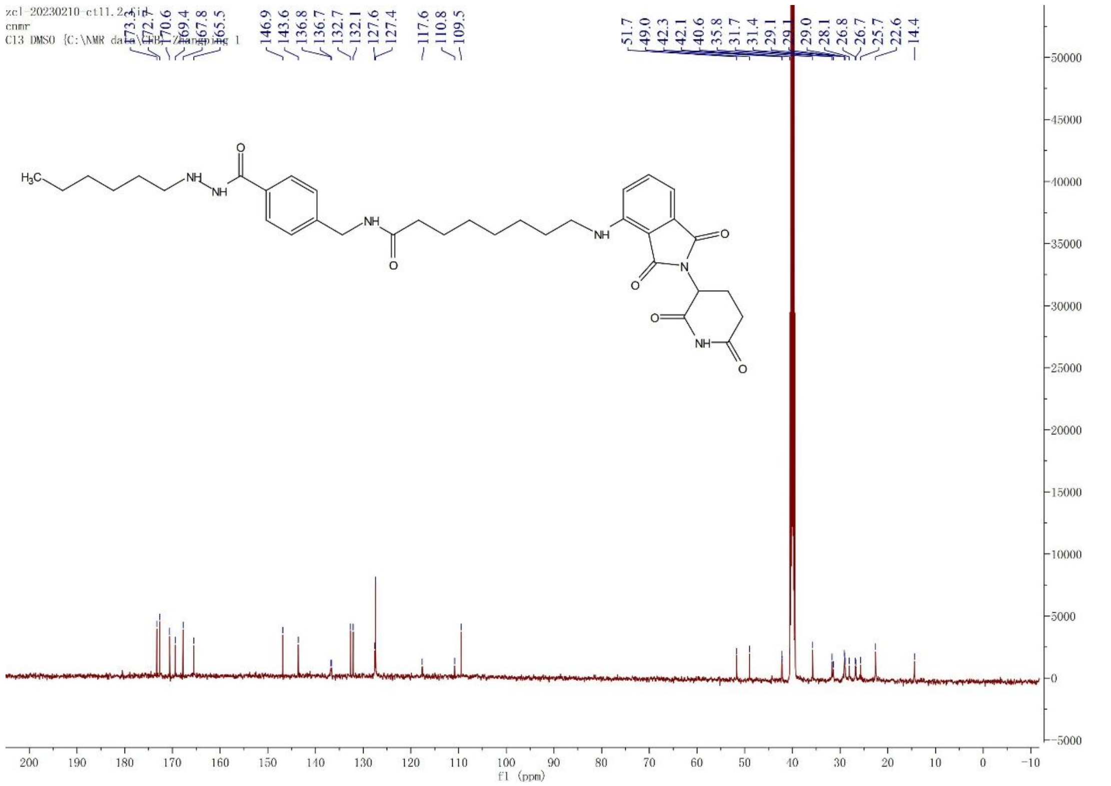

^{1}H NMR of **Z6** in DMSO-d_6

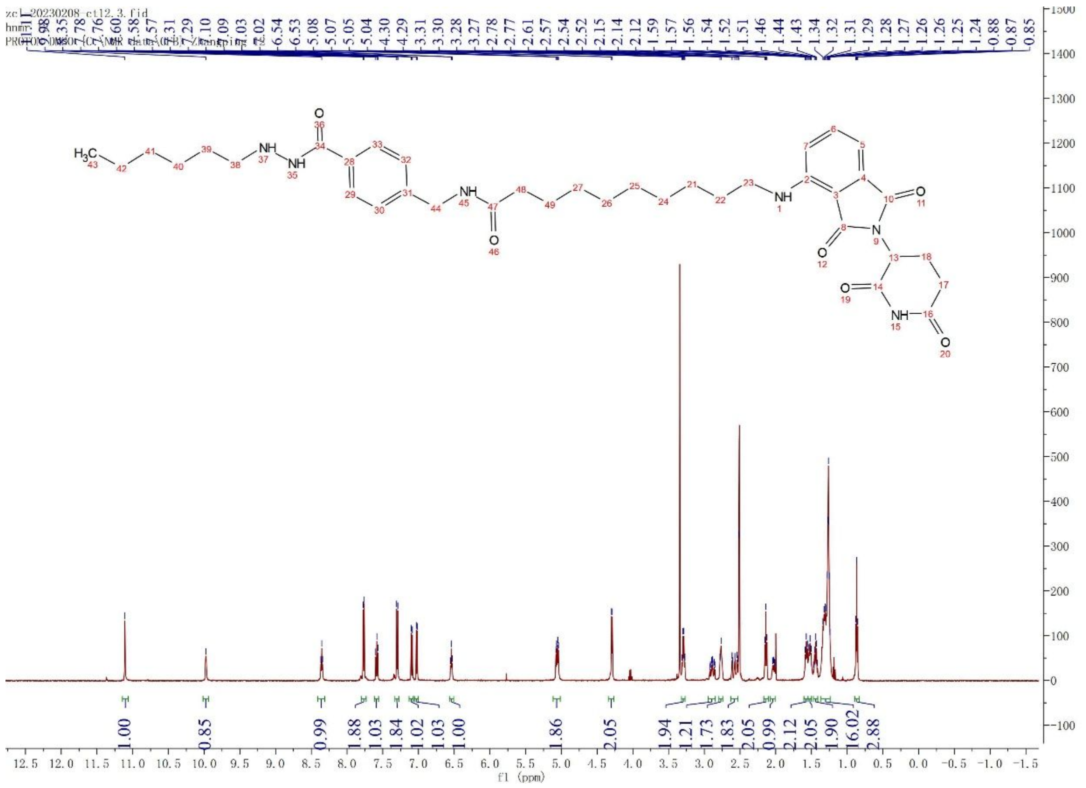

^{13}C NMR of **Z6** in DMSO-d_6

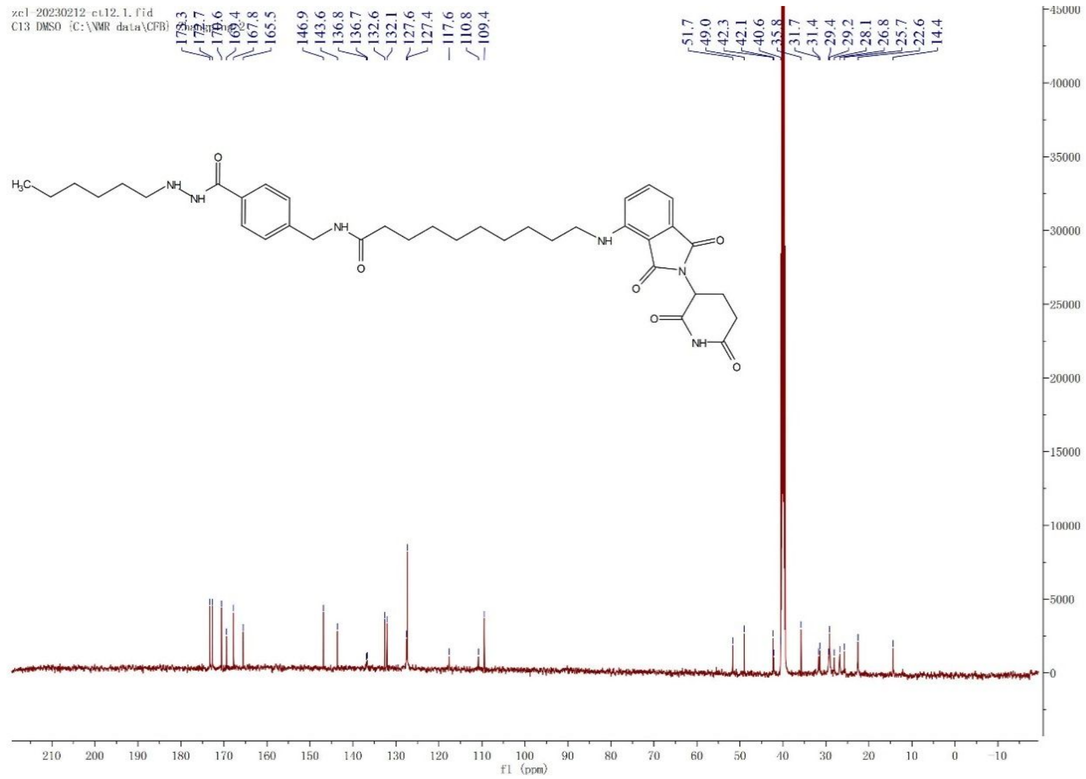

^{1}H NMR of **Z7** in DMSO-d_6

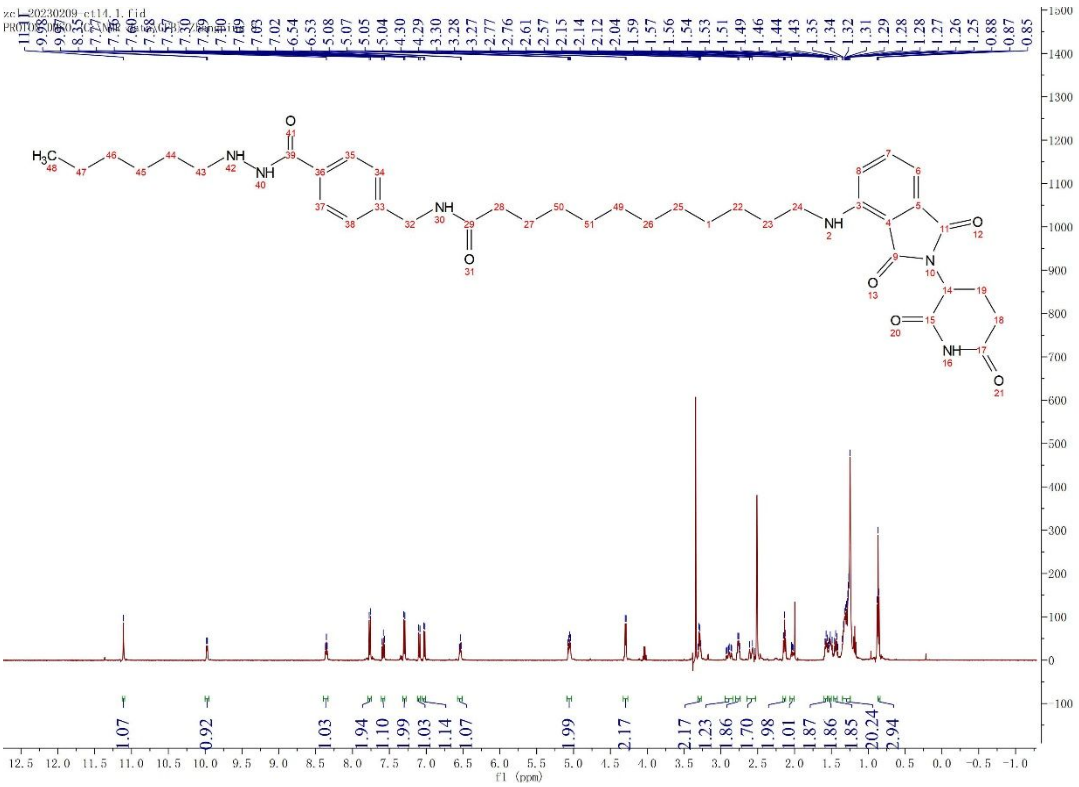

^{13}C NMR of **Z7** in DMSO-d_6

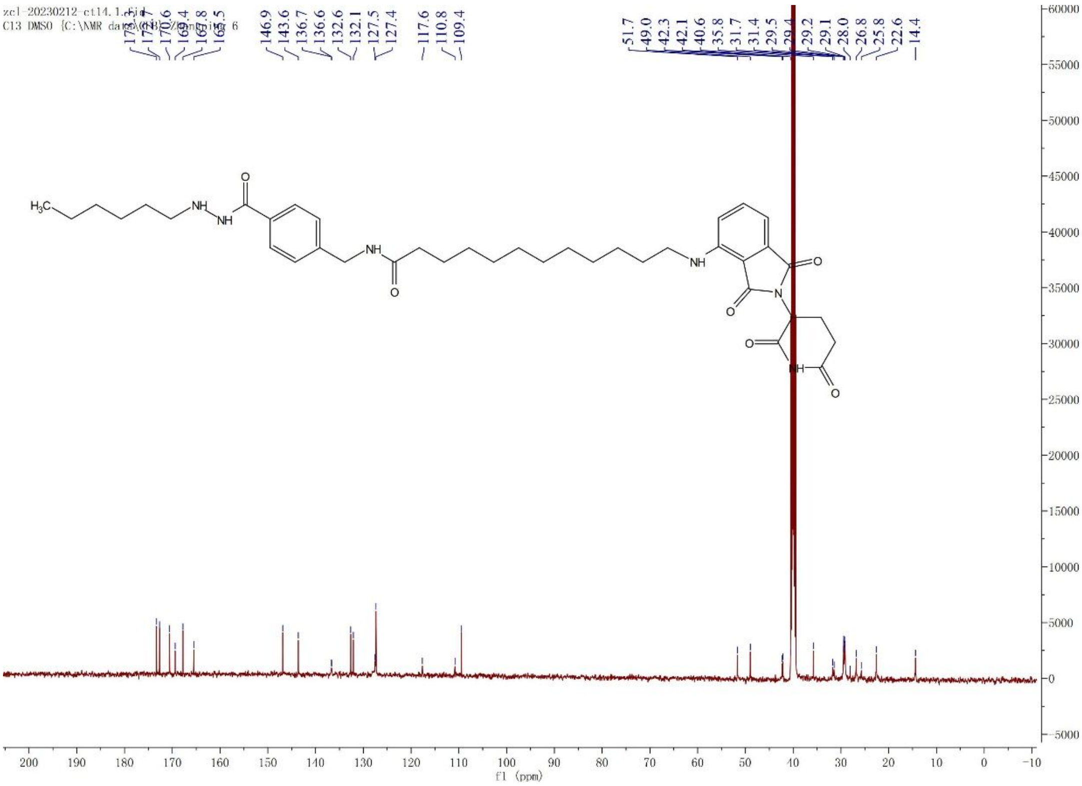

^{1}H NMR of **Z9** in DMSO-d_6

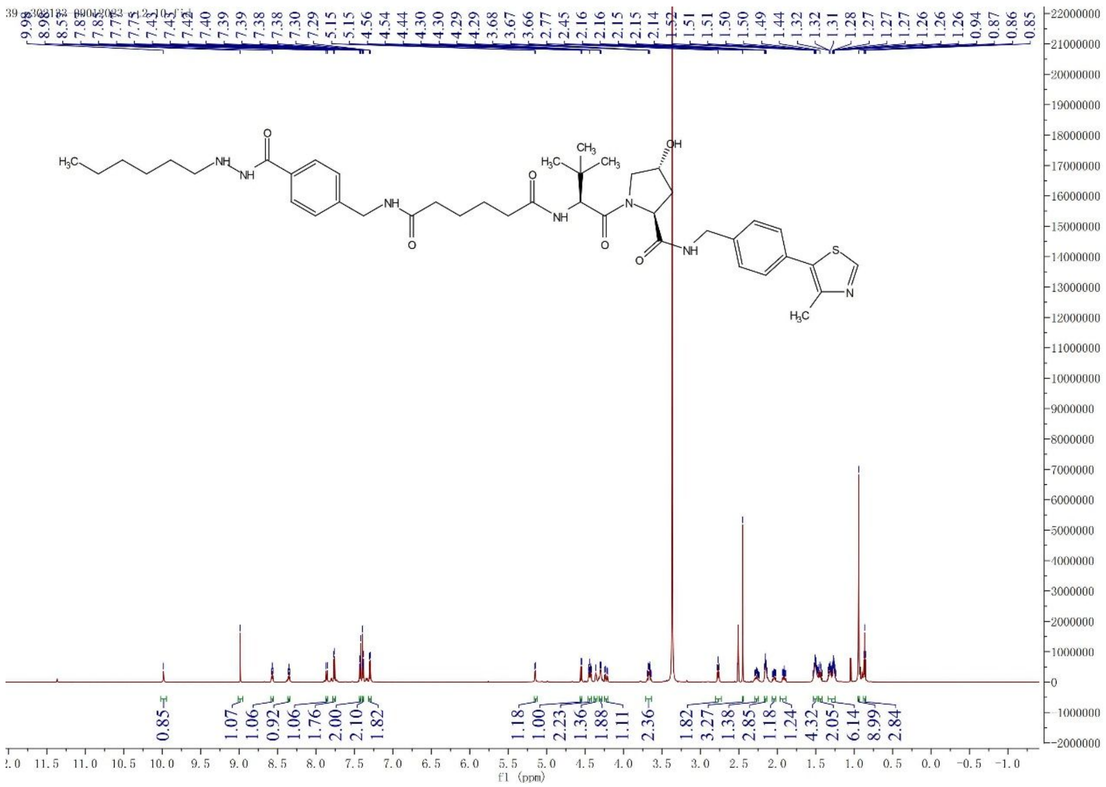

^{13}C NMR of **Z9** in DMSO-d_6

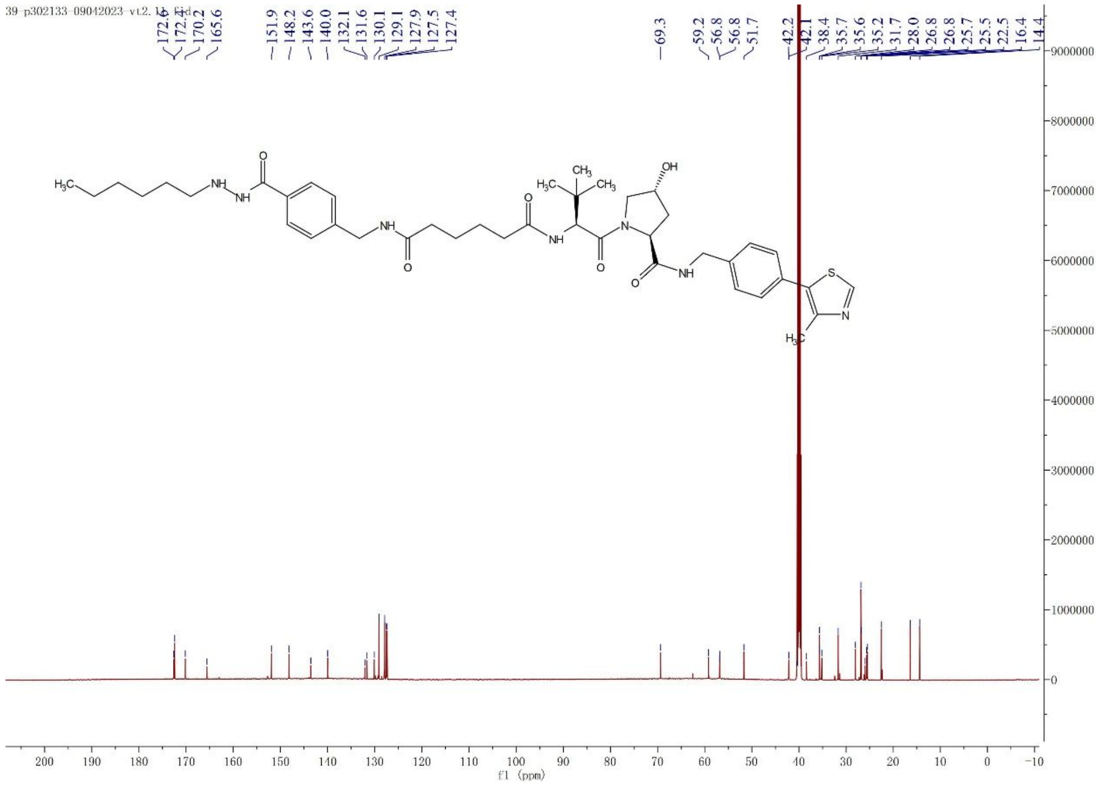

^{1}H NMR of **Z10** in DMSO-d_6

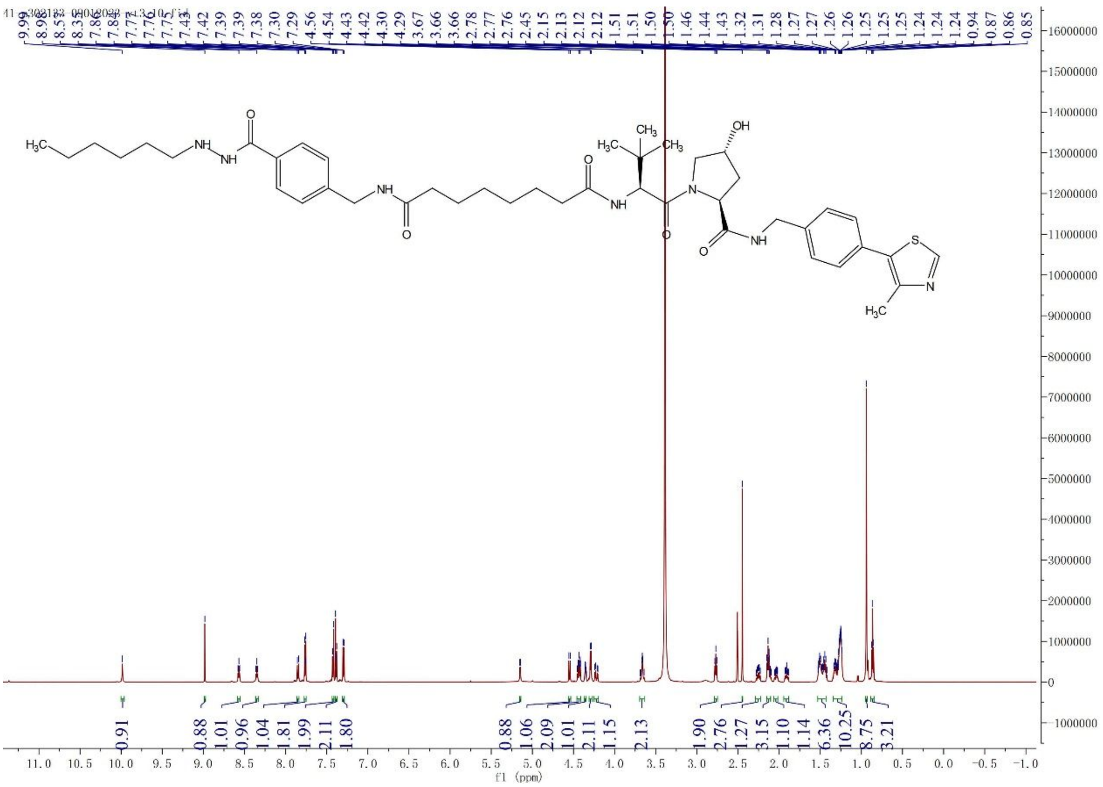

^{13}C NMR of **Z10** in DMSO-d_6

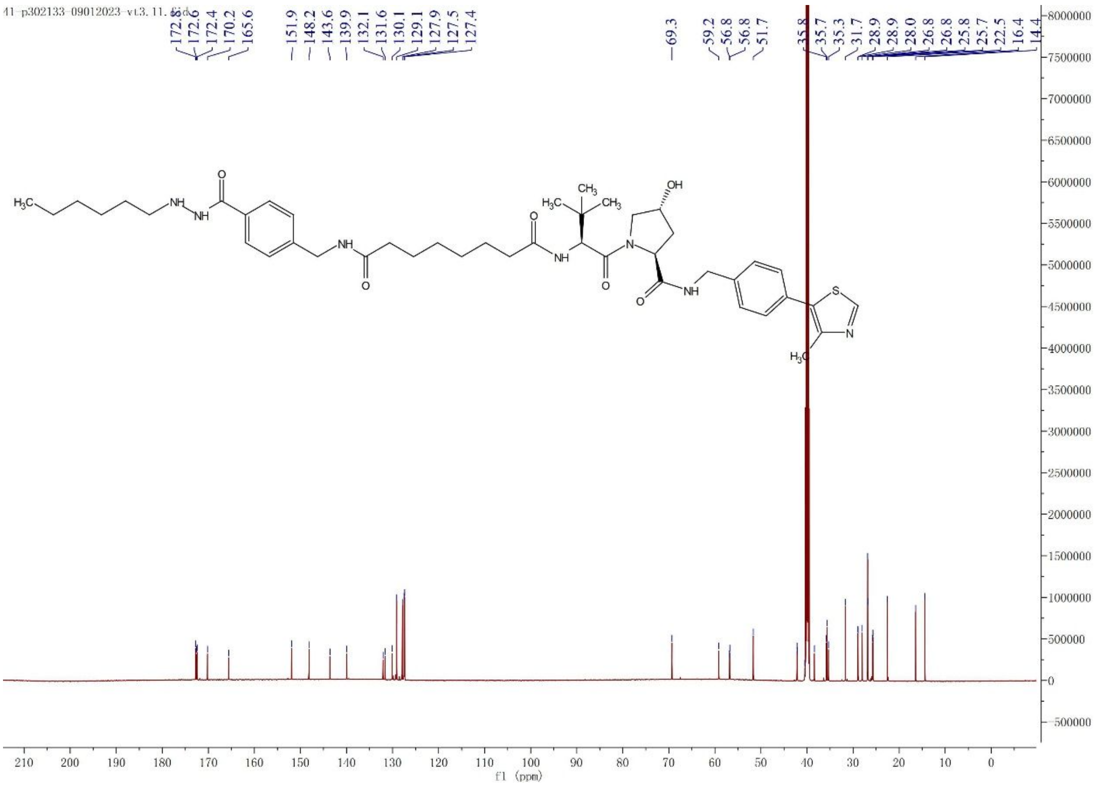

^{1}H NMR of **Z11** in DMSO-d_6

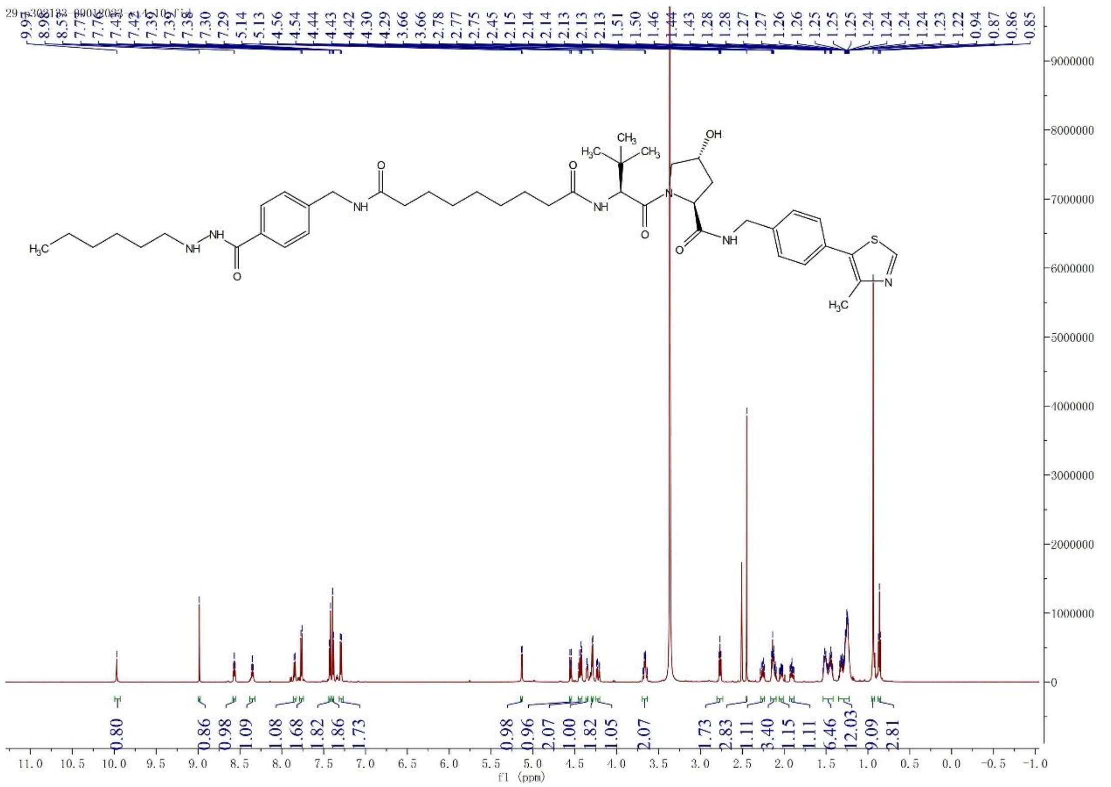

^{13}C NMR of **Z11** in DMSO-d_6

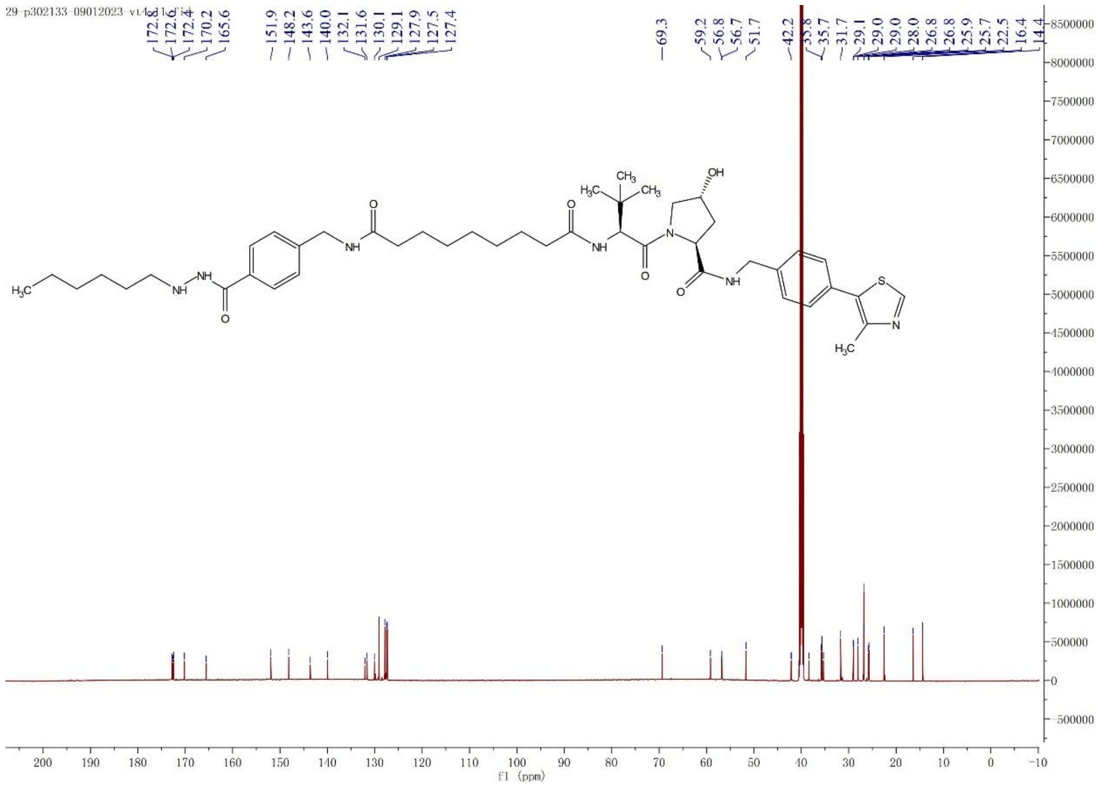

^{1}H NMR of **Z12** in DMSO-d_6

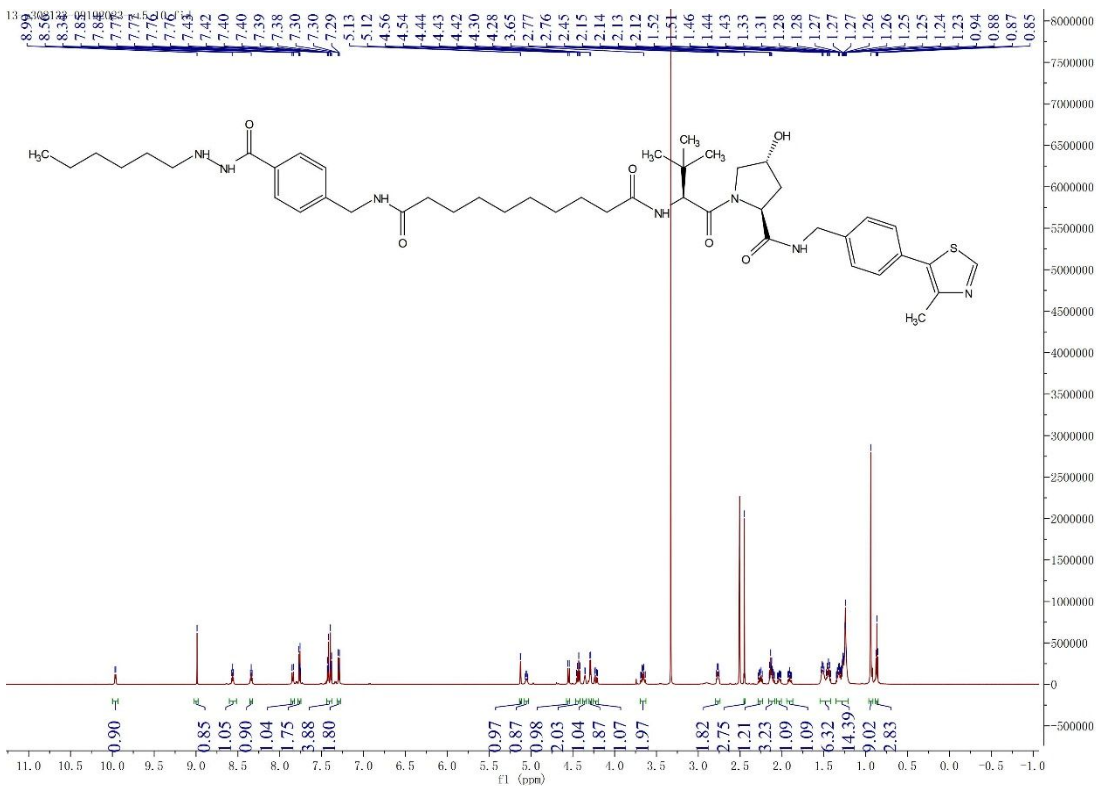

^{13}C NMR of **Z12** in DMSO-d_6

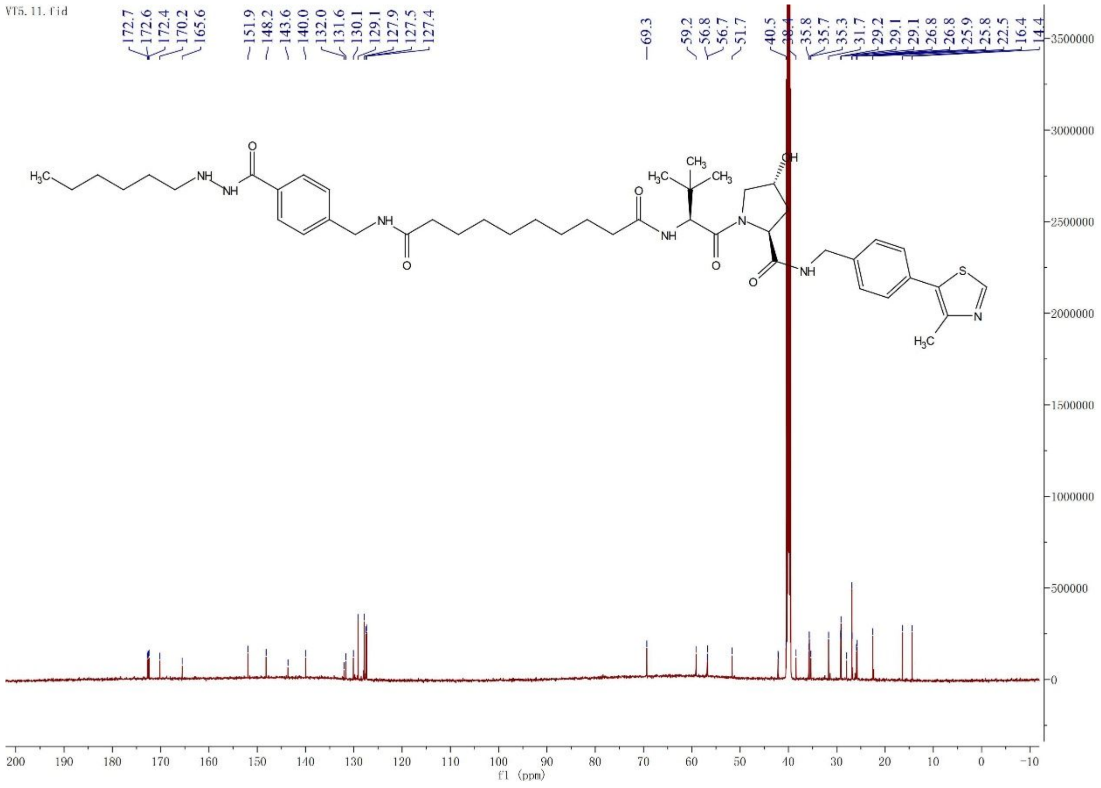

^{1}H NMR of **Z13** in DMSO-d_6

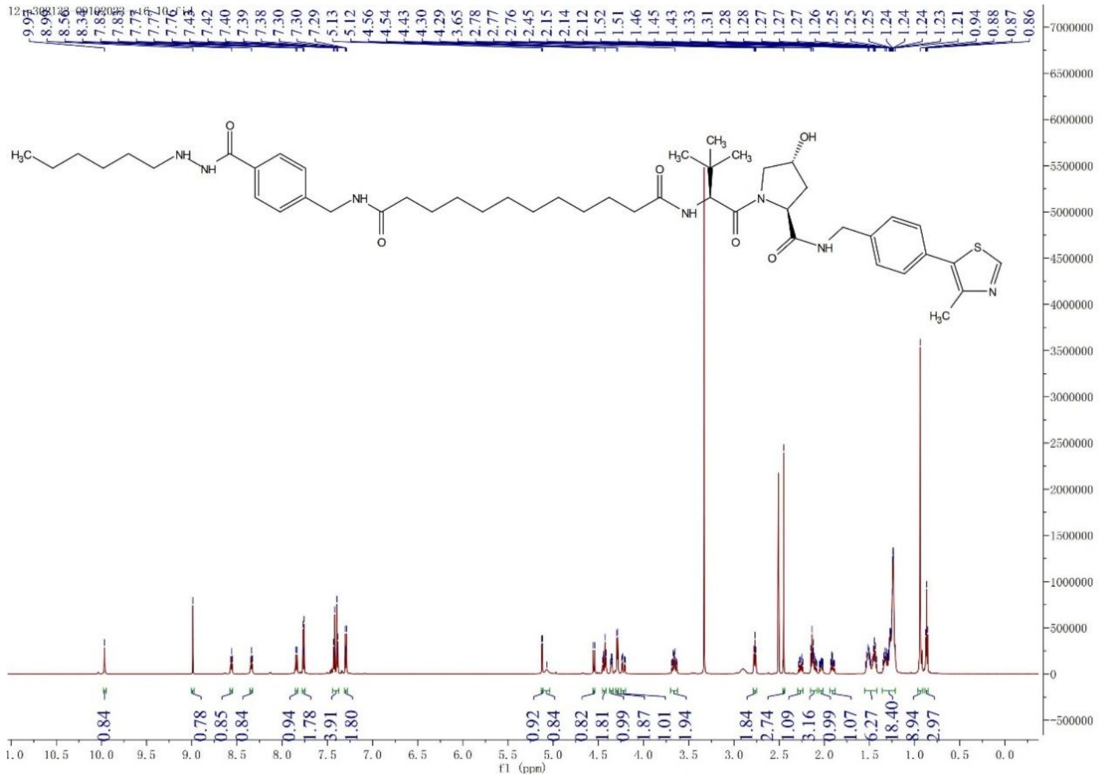

^{13}C NMR of **Z13** in DMSO-d_6

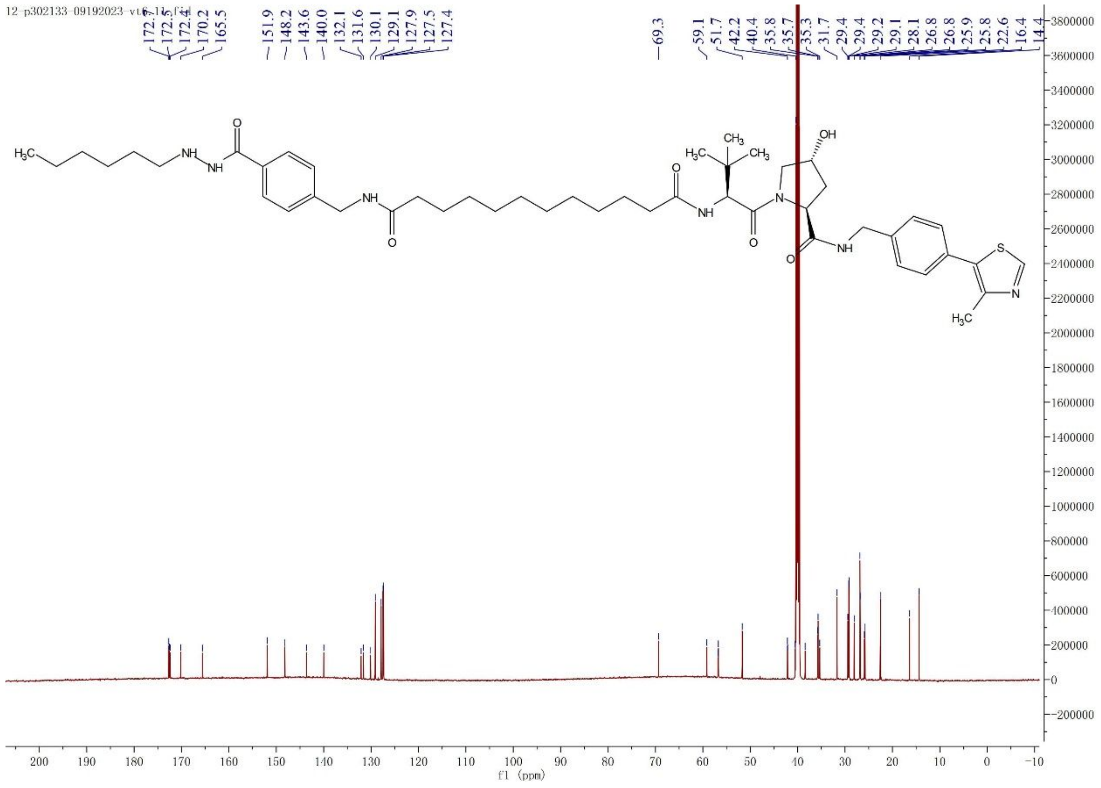

^{1}H NMR of **16a** in DMSO-d_6

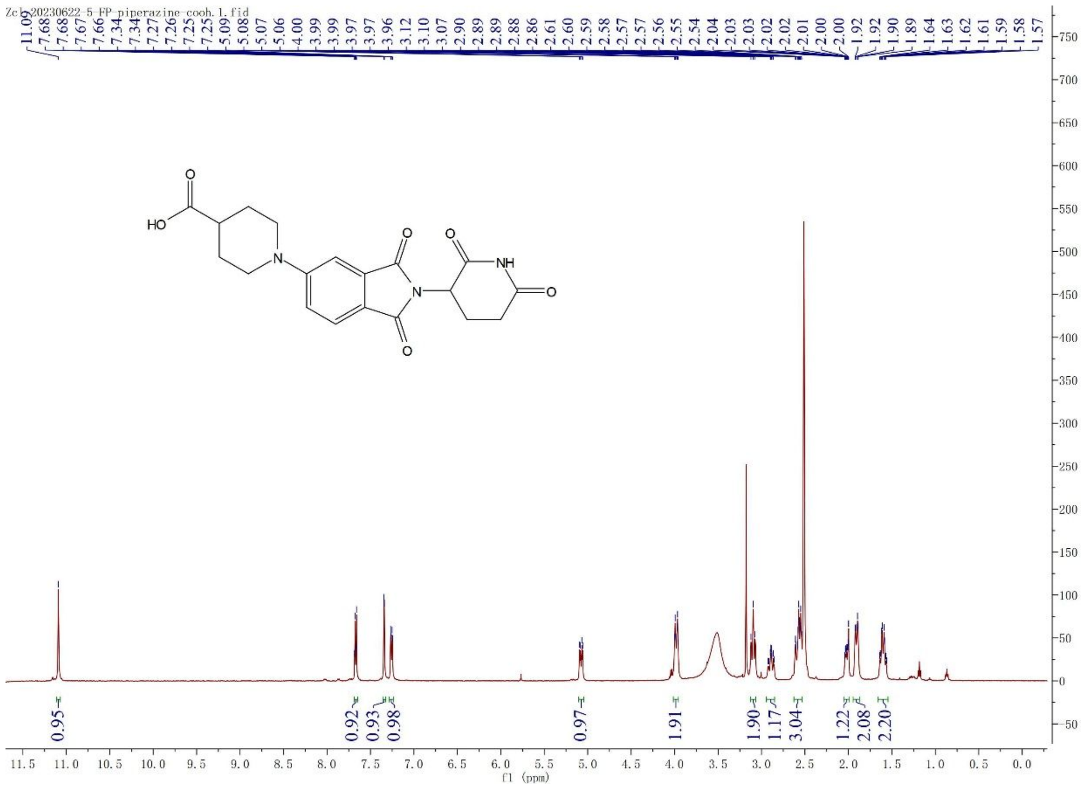

^{1}H NMR of **16b** in DMSO-d_6

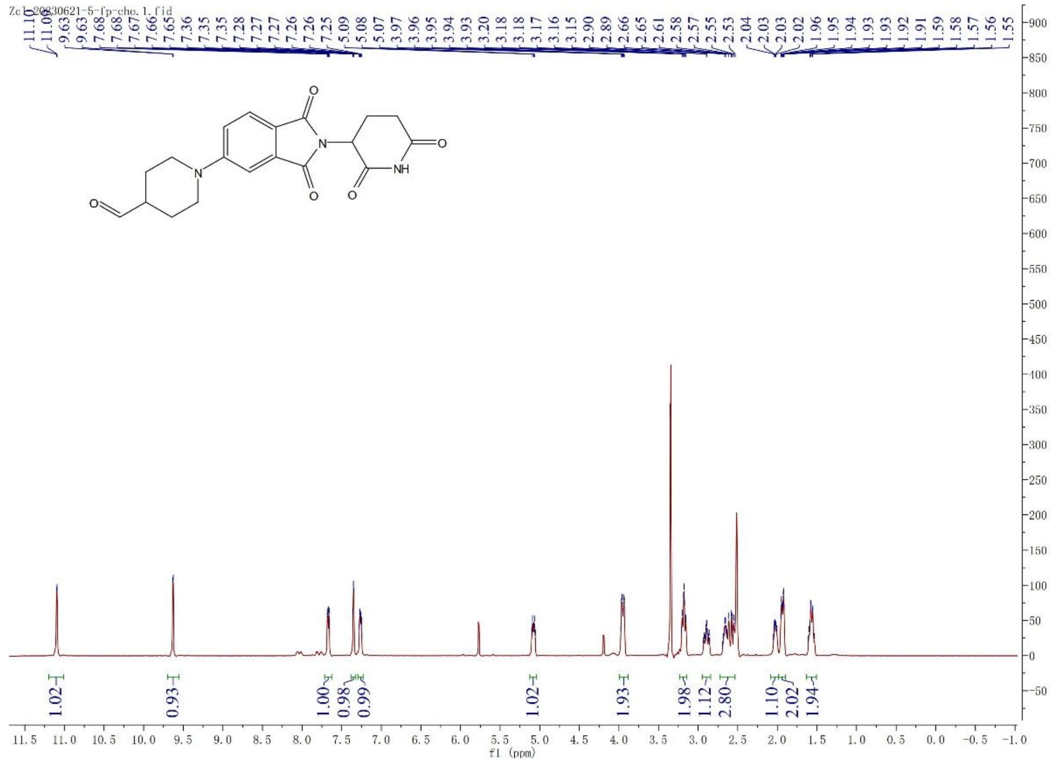

^{1}H NMR of **Z14** in DMSO-d_6

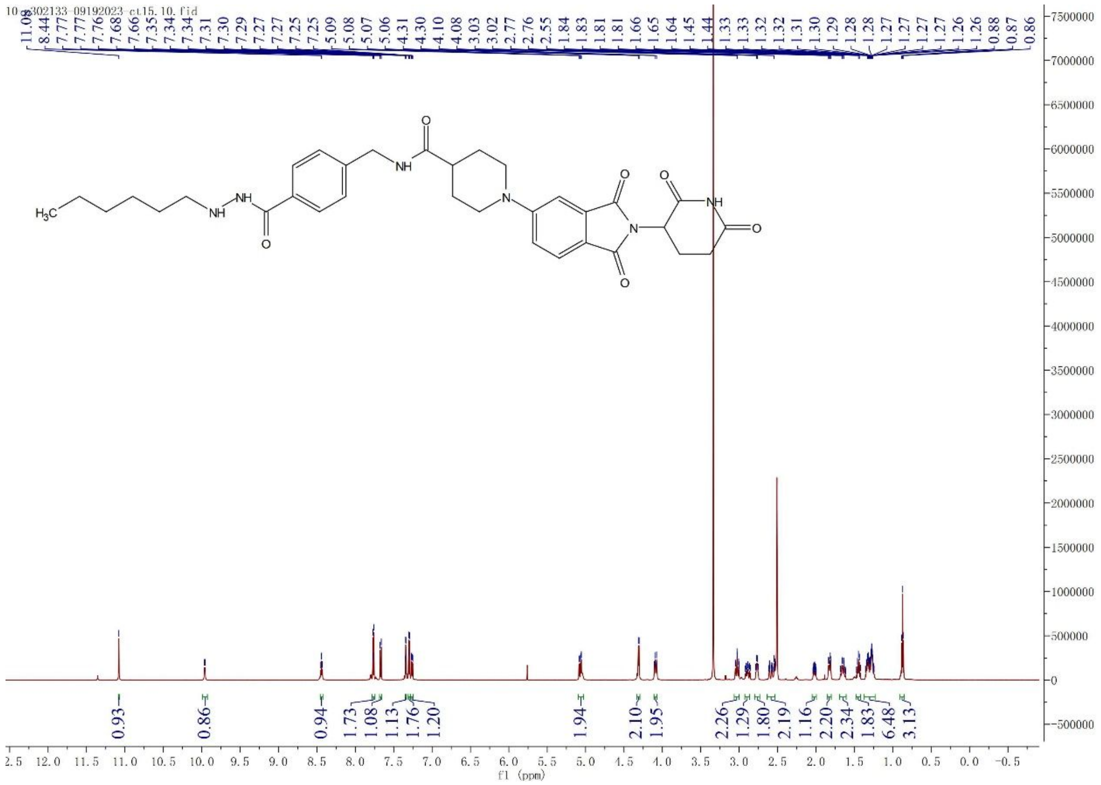

^{13}C NMR of **Z14** in DMSO-d_6

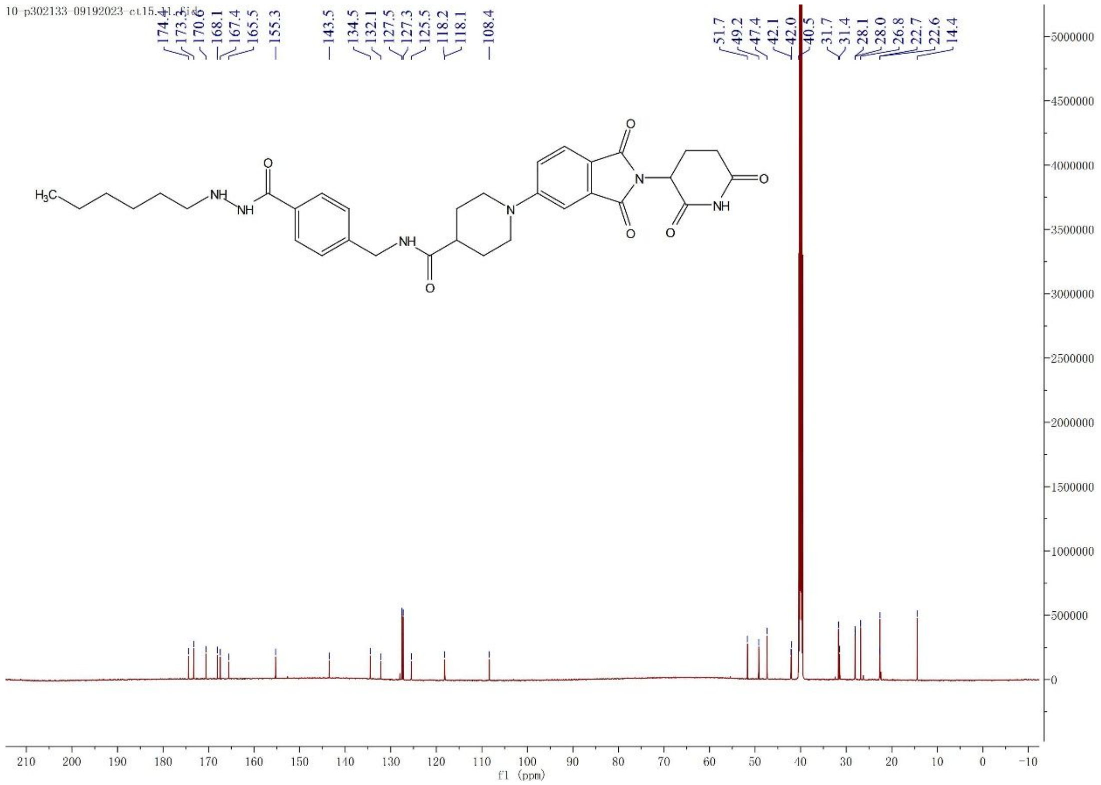

^{1}H NMR of **Z15** in DMSO-d_6

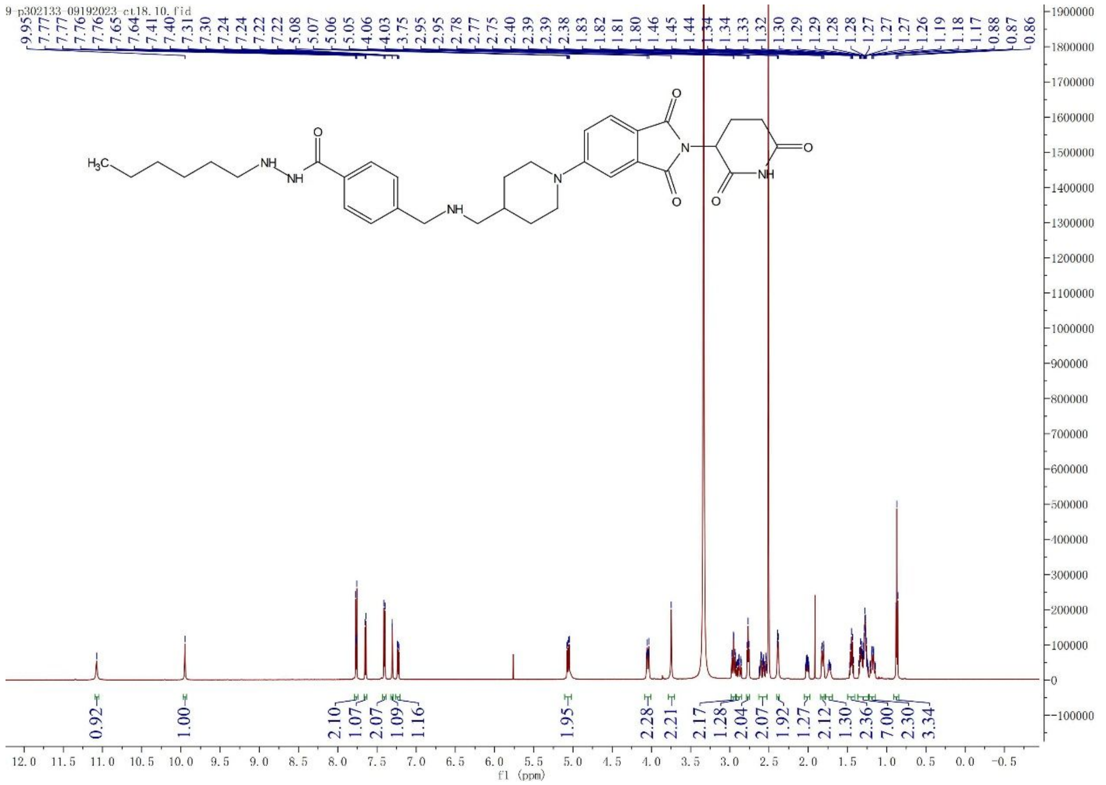

^{13}C NMR of **Z15** in DMSO-d_6

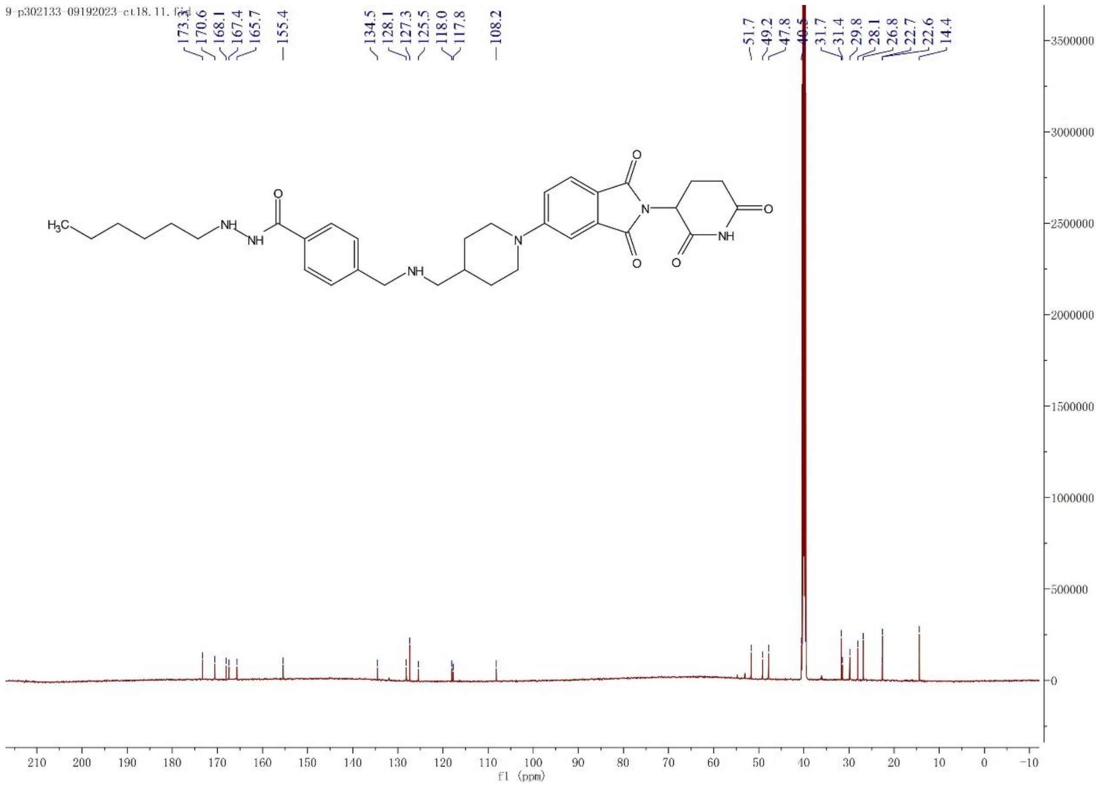

^{1}H NMR of **18** in DMSO-d_6

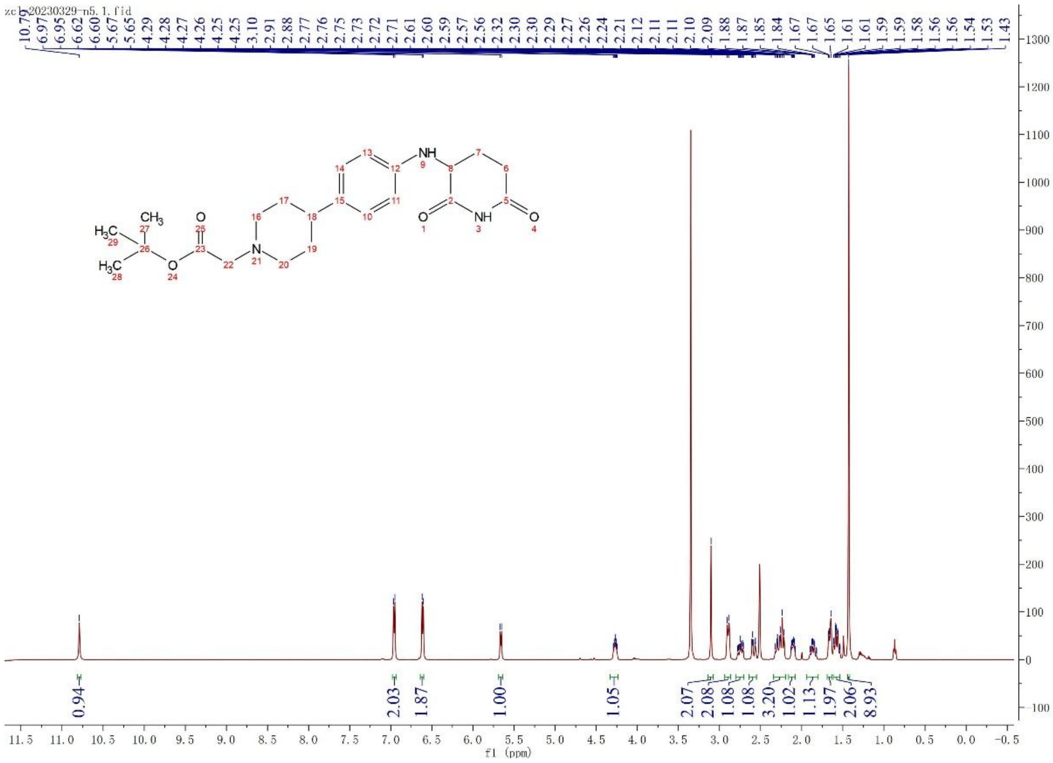

^{1}H NMR of **19** in DMSO-d_6

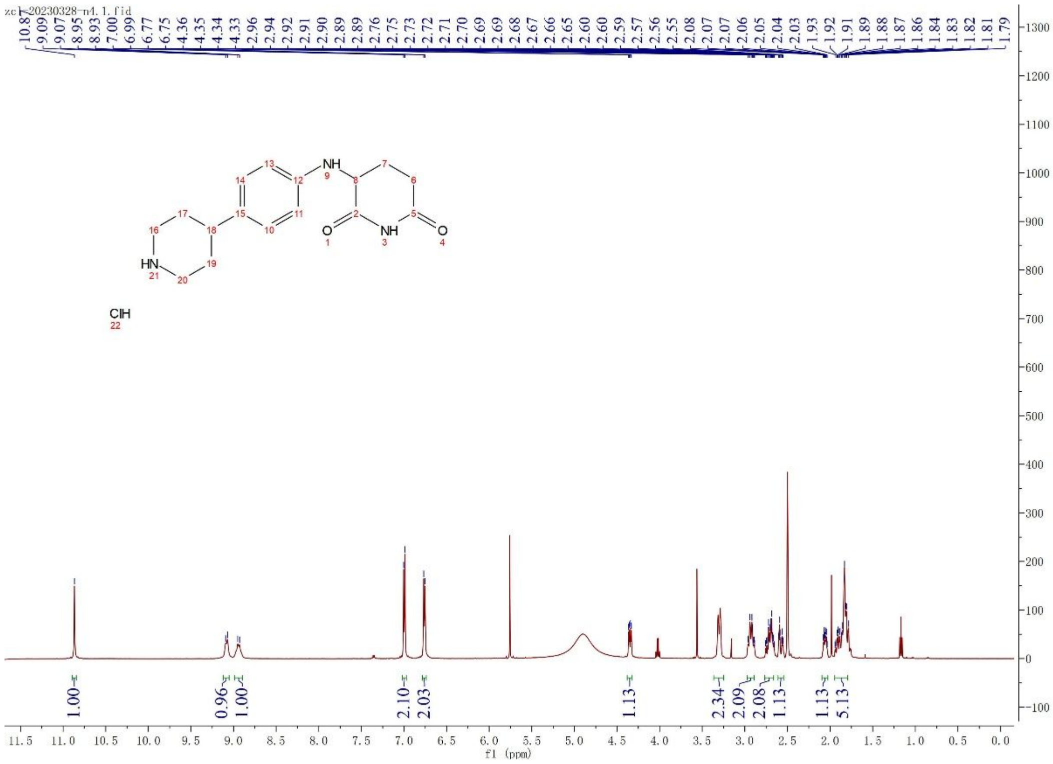

^{1}H NMR of **Z16** in DMSO-d_6

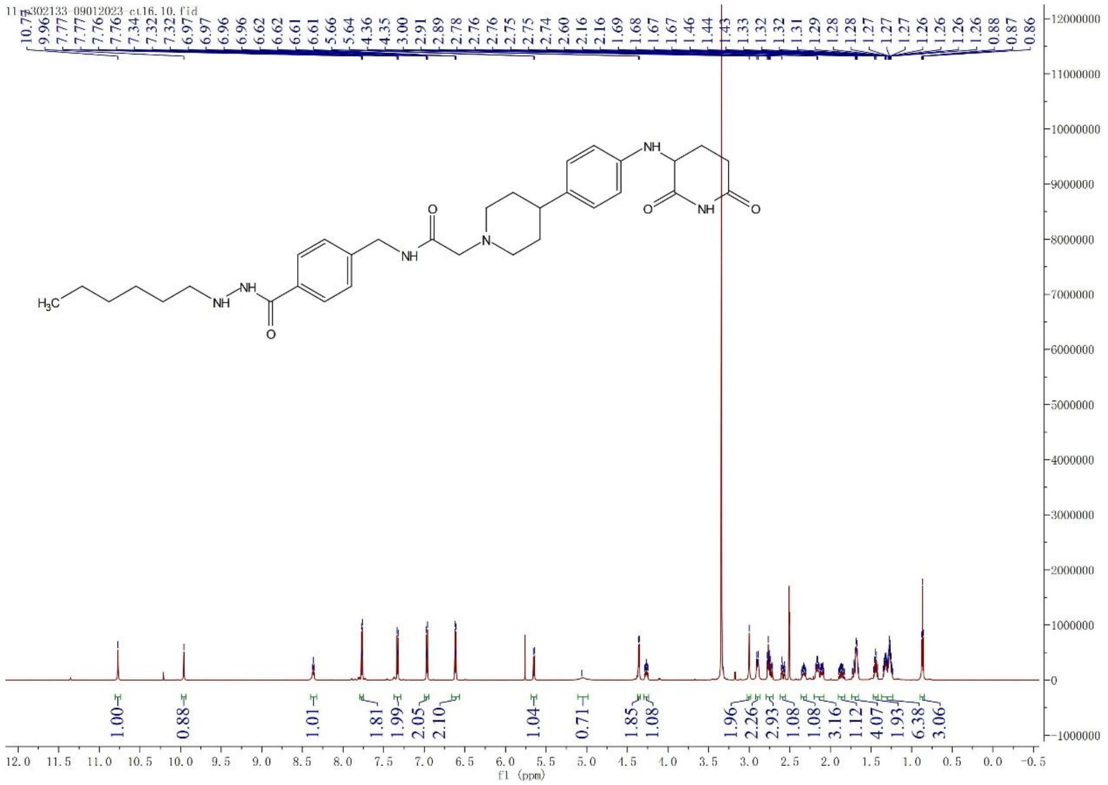

^{13}C NMR of **Z16** in DMSO-d_6

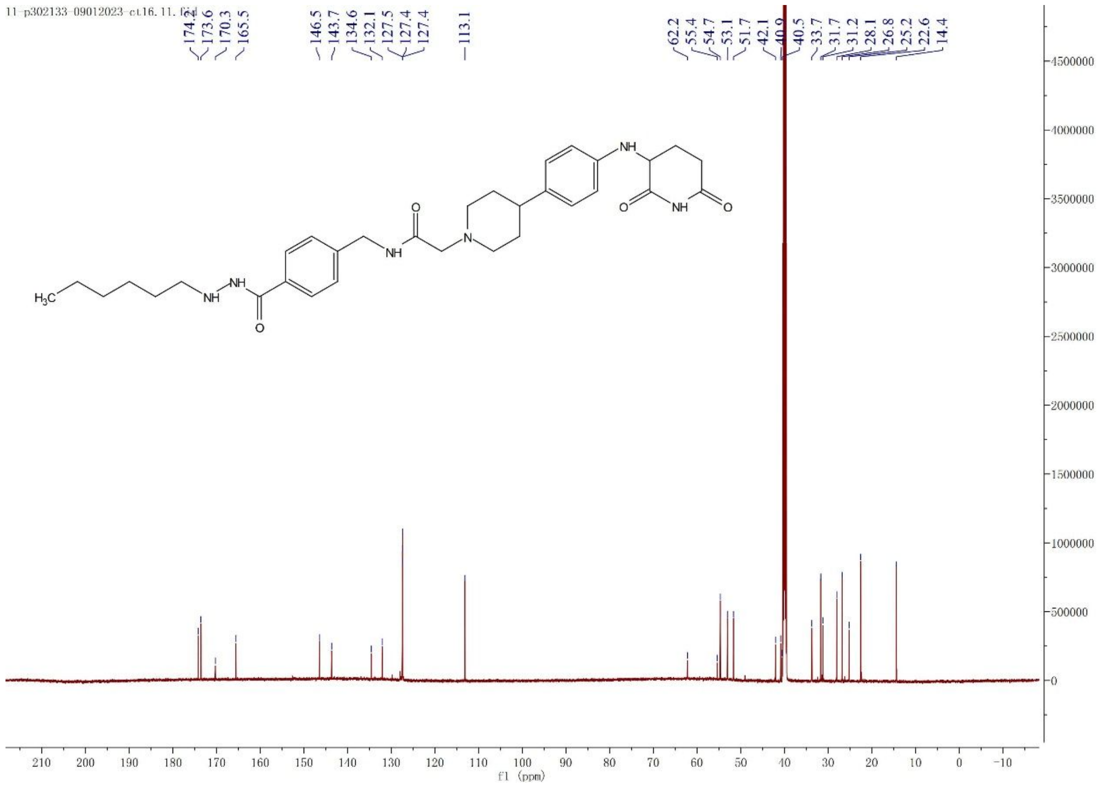

^1H NMR of **22** in DMSO-d_6

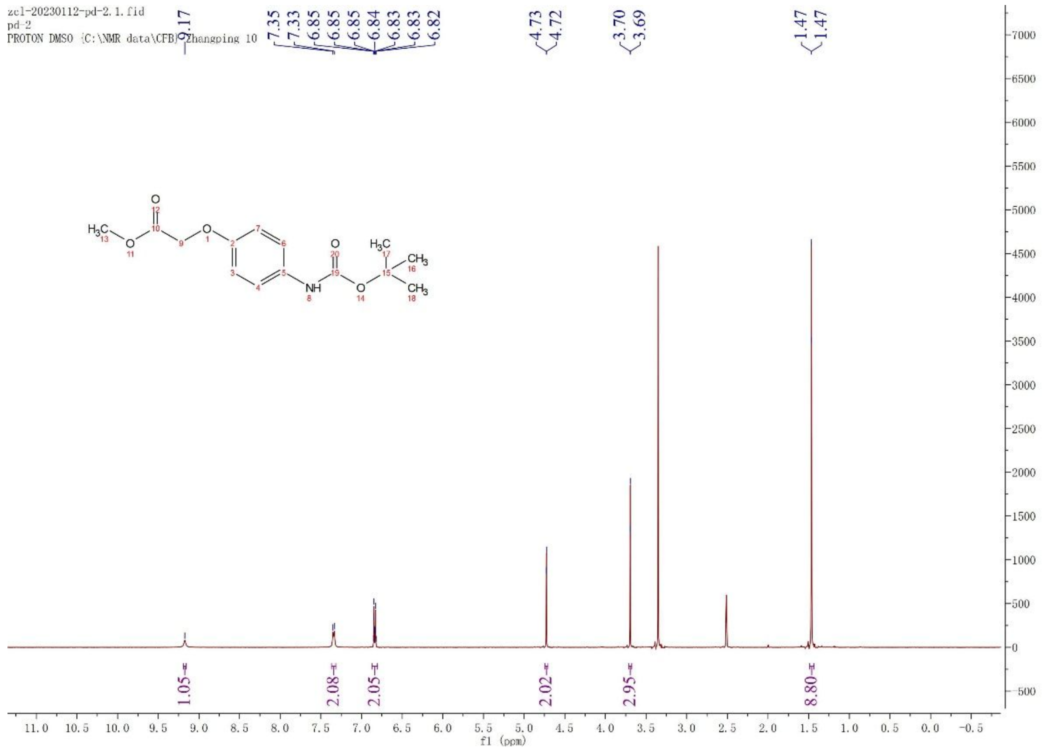

^1H NMR of **25** in DMSO-d_6

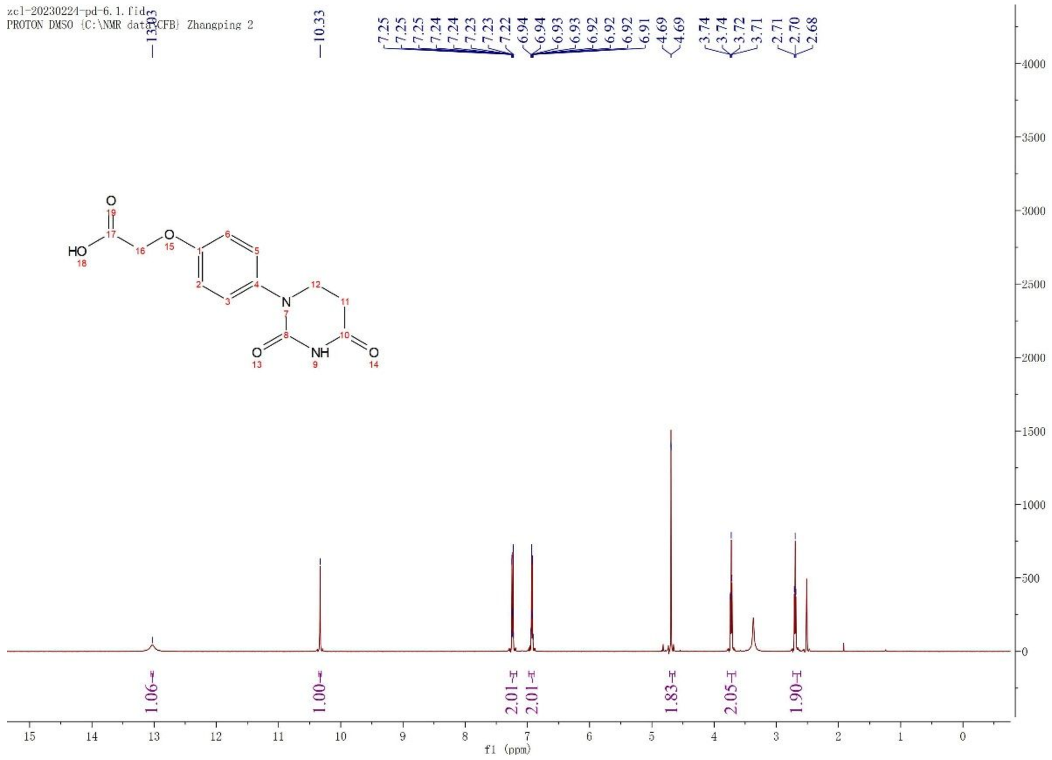

^{1}H NMR of **27** in DMSO-d_6

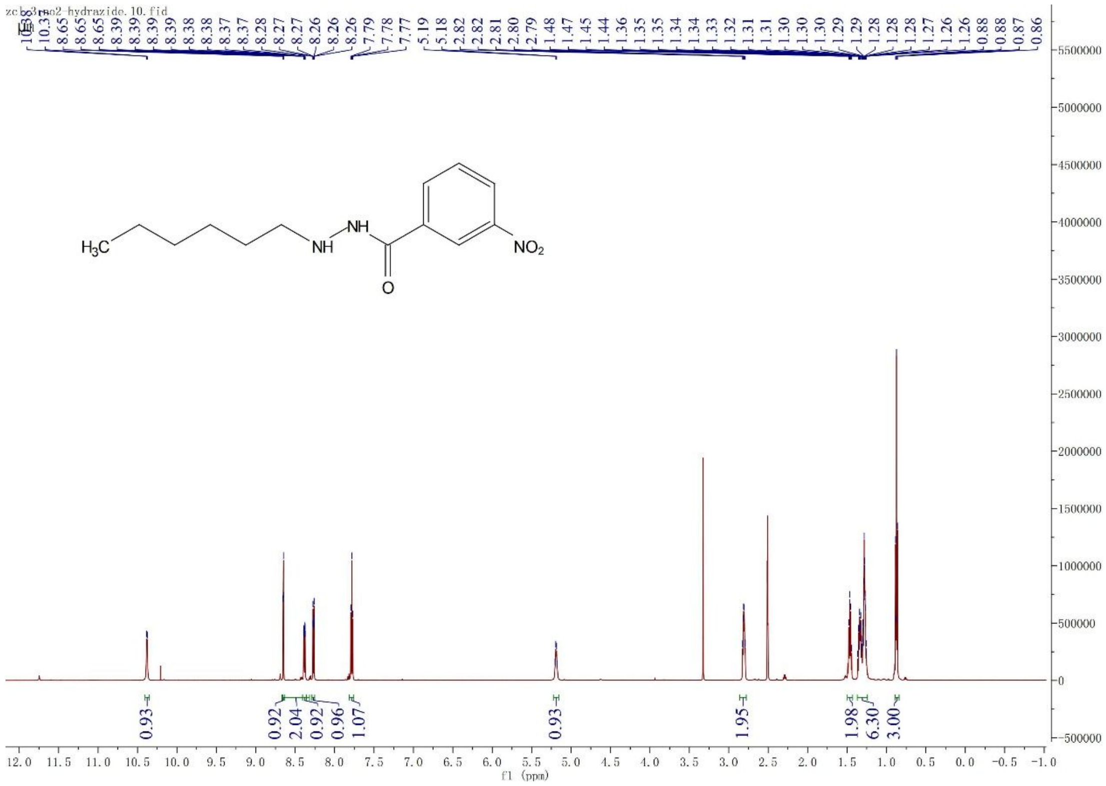

^{1}H NMR of **29** in DMSO-d_6

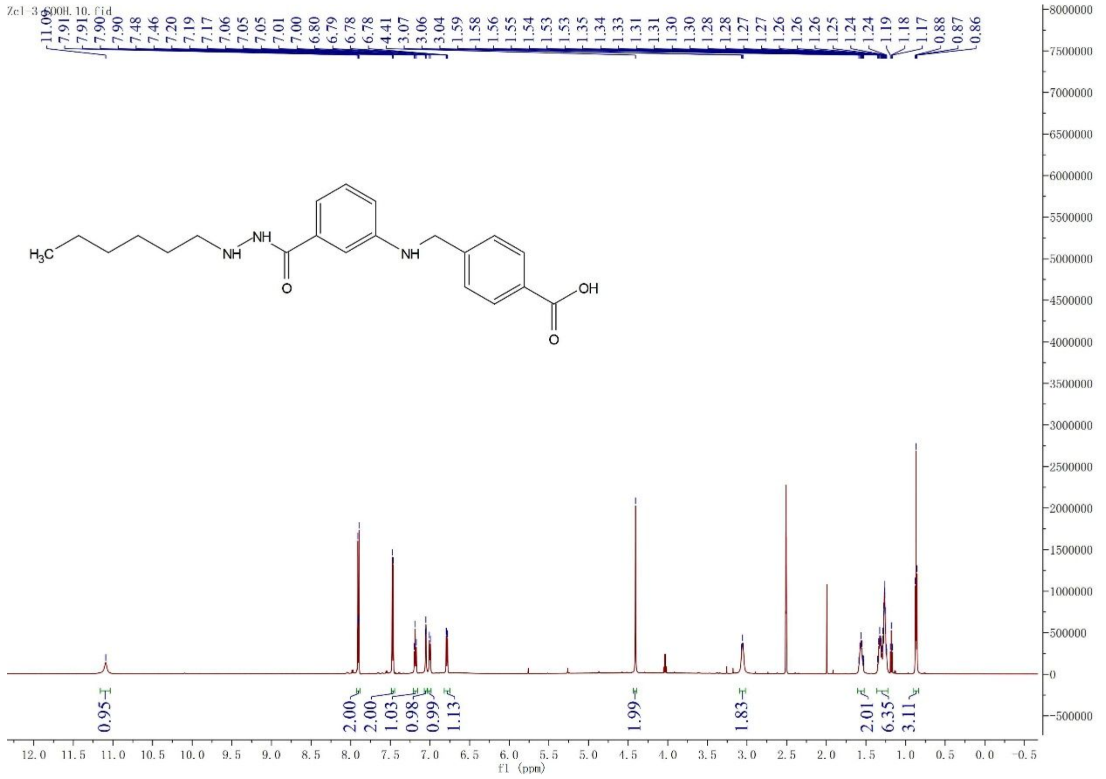

^{1}H NMR of **Z18** in DMSO-d_6

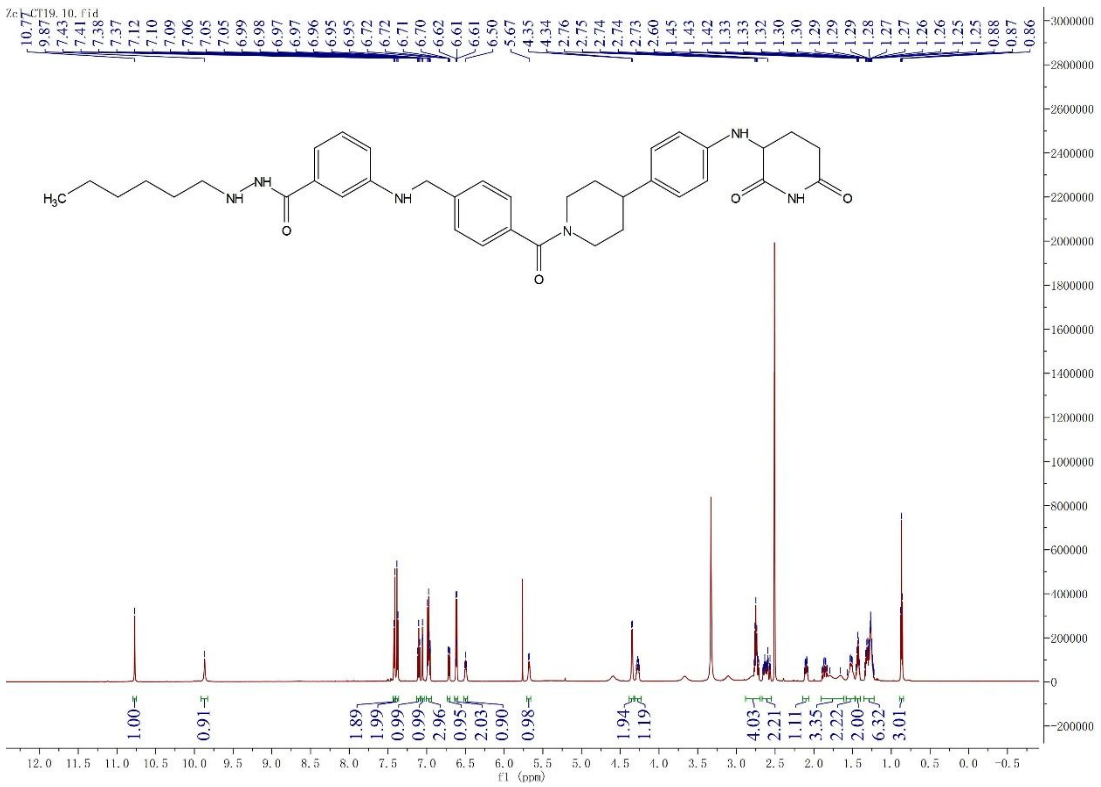

^{13}C NMR of **Z18** in DMSO-d_6

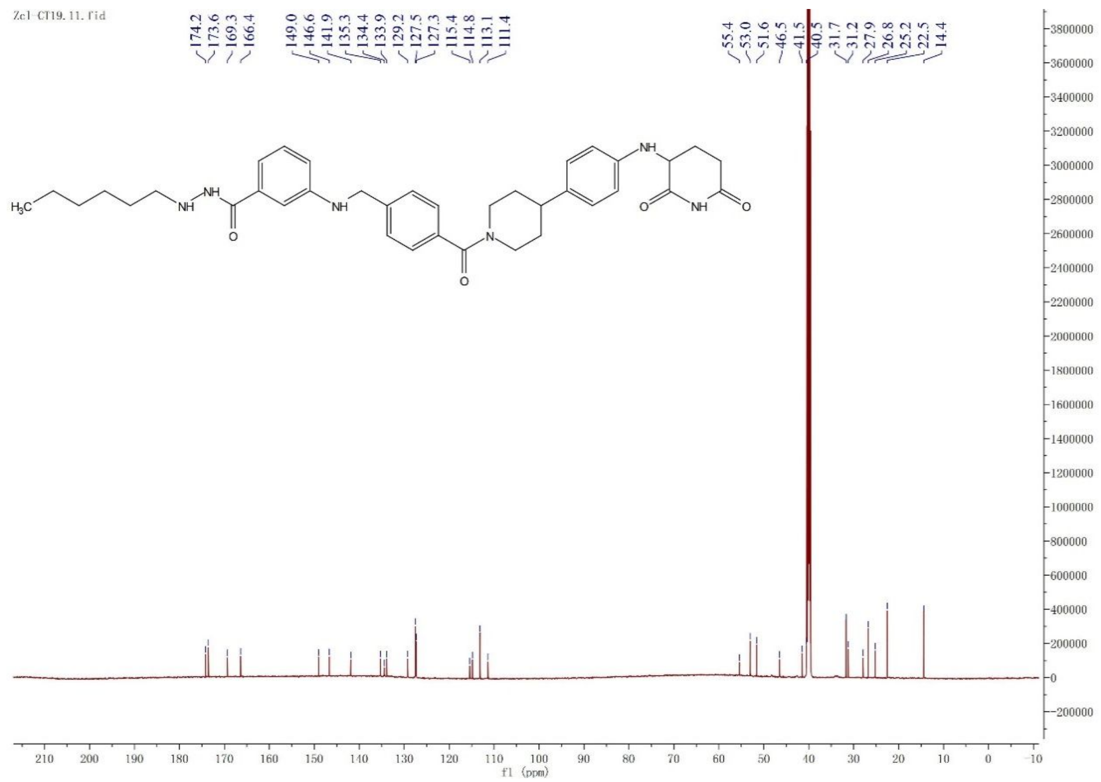

^{1}H NMR of **NC-Z16** in DMSO-d_6

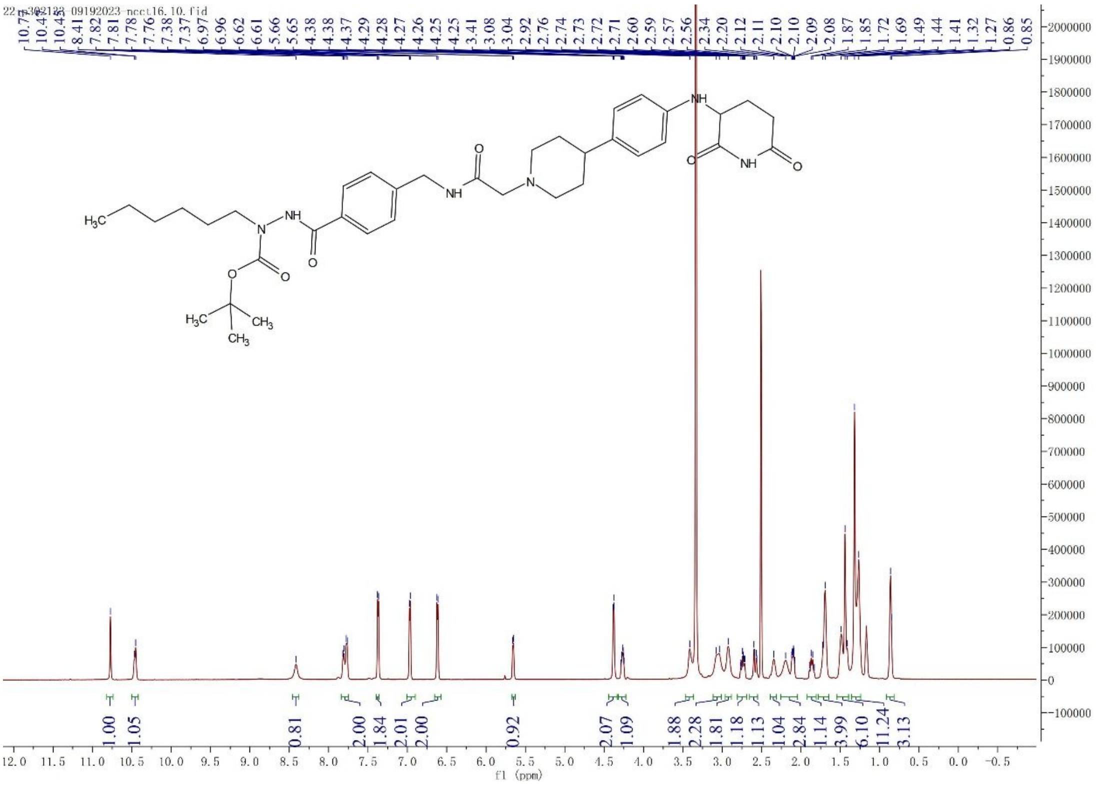

^{13}C NMR of **NC-Z16** in DMSO-d_6

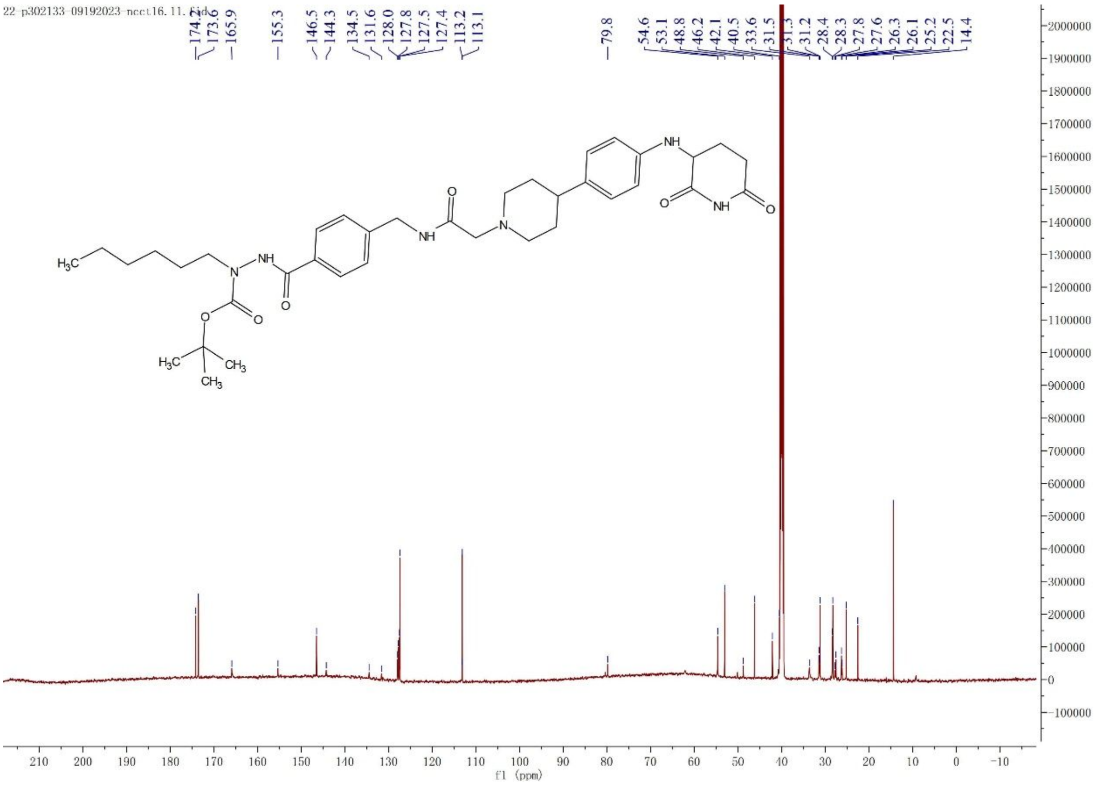

^{1}H NMR of **32** in DMSO-d_6

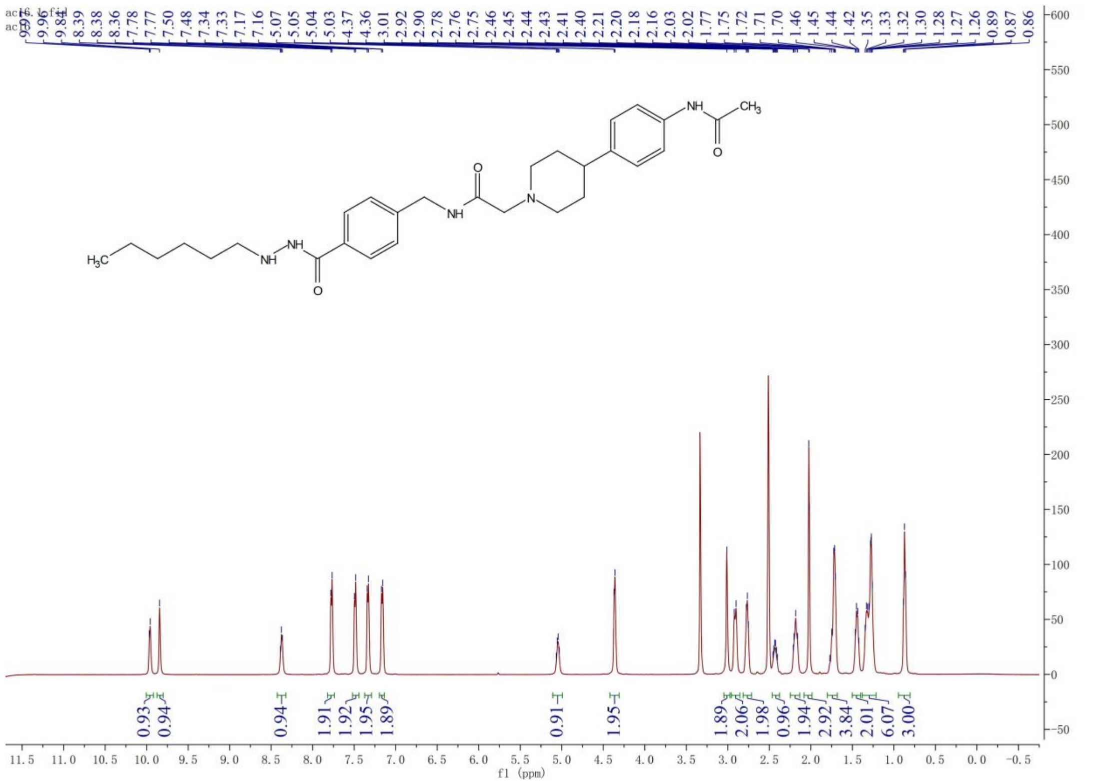

^{13}C NMR of **32** in DMSO-d_6

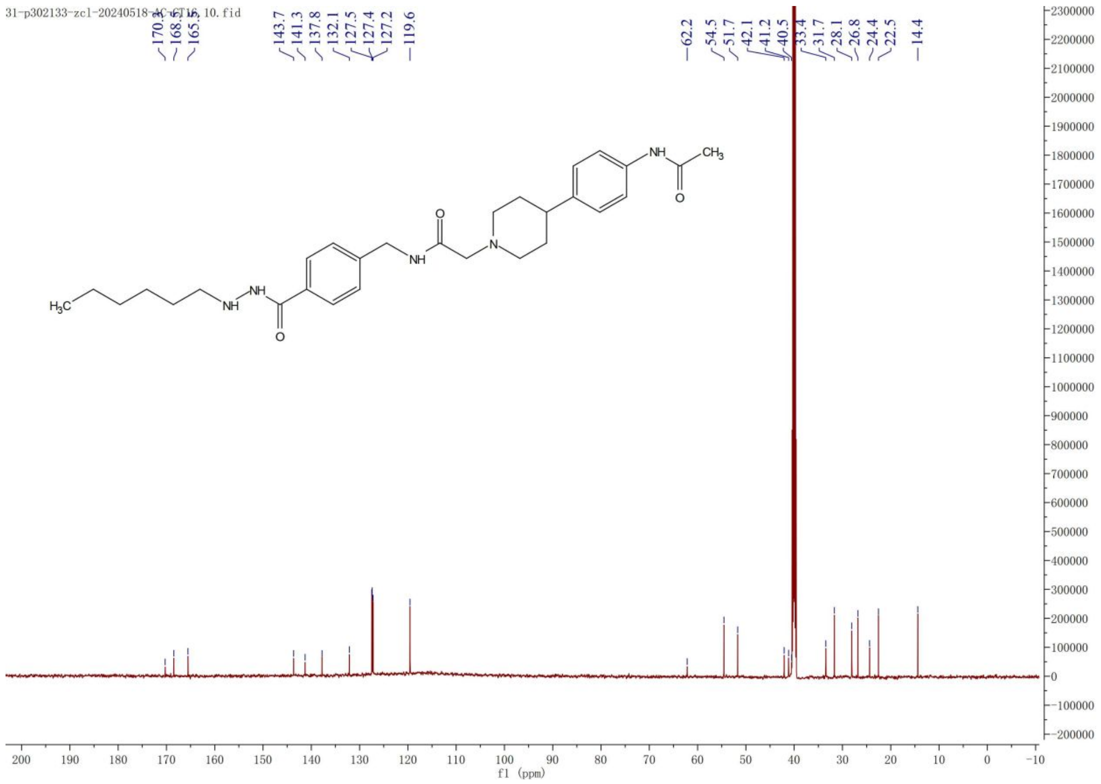

Representative HRMS Spectra

6

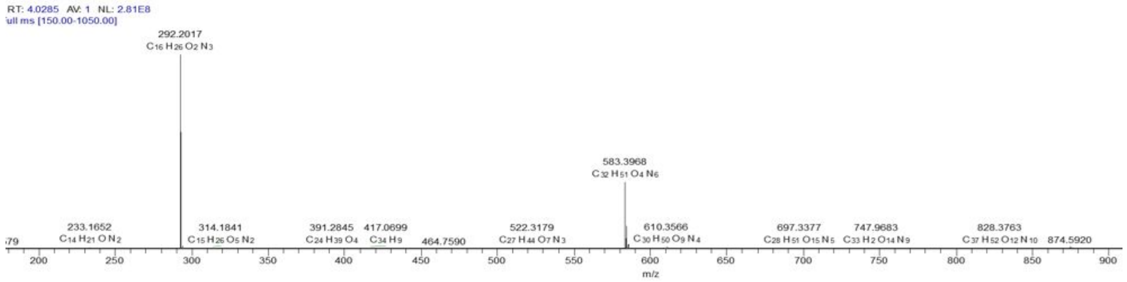

19

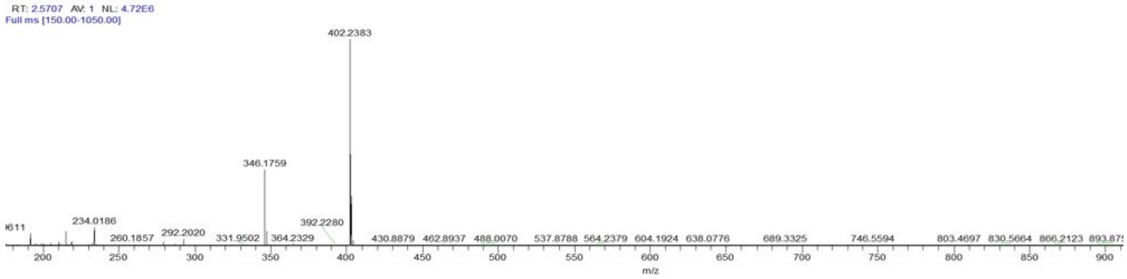

Z1

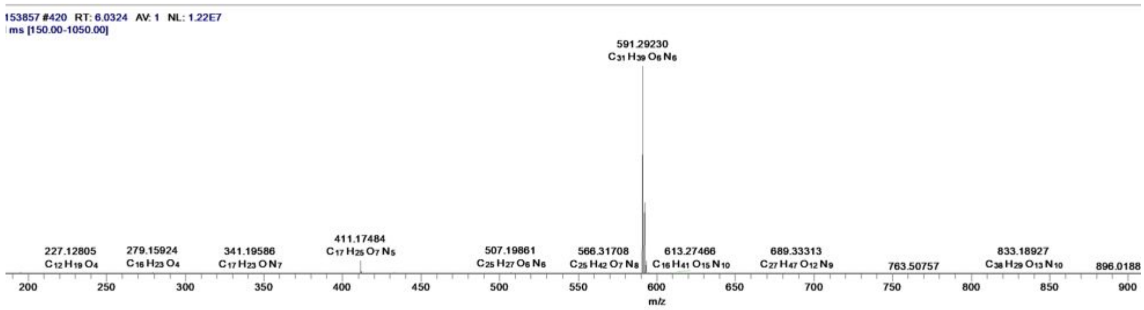

Z2

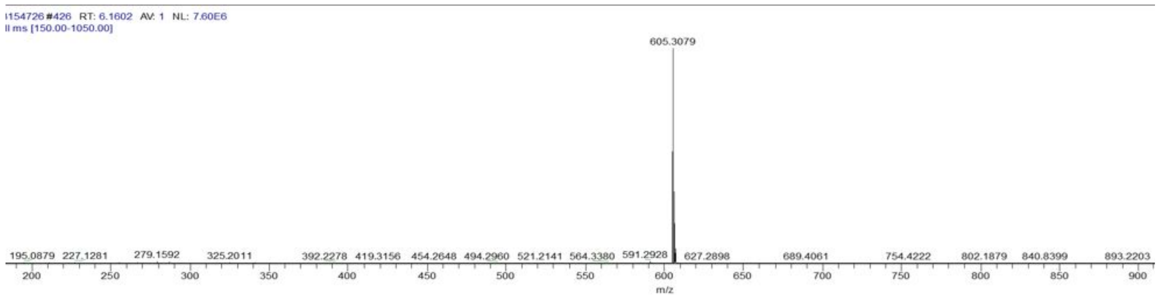

Z3

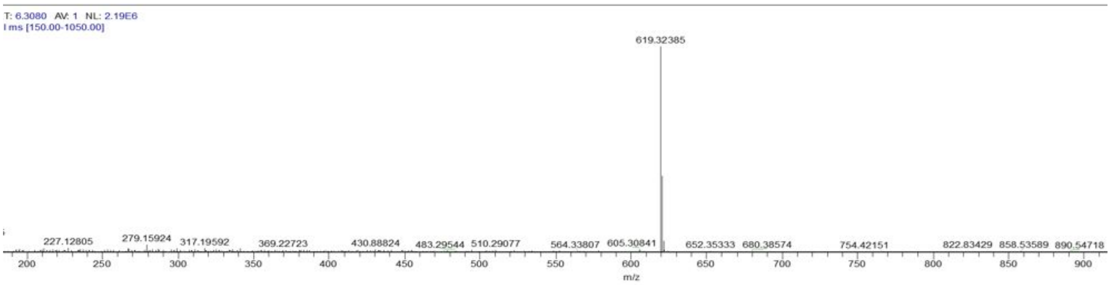
T: 6.3080 AV: 1 NL: 2.19E6
l ms [150.00-1050.00]
619.32385
227.12805 279.15924 317.19592 369.22723 430.88824 483.29544 510.29077 564.33807 605.30841 652.35333 680.38574 754.42151 822.83429 858.53589 890.54718
200 250 300 350 400 450 500 550 600 650 700 750 800 850 900
m/z

Z4

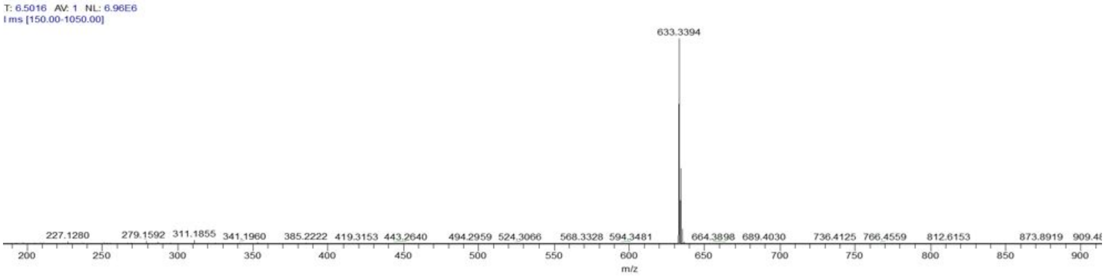
T: 6.5016 AV: 1 NL: 6.96E6
l ms [150.00-1050.00]
633.3394
227.1280 279.1592 311.1855 341.1960 385.2222 419.3153 443.2640 494.2959 524.3066 568.3328 594.3481 664.3898 689.4030 736.4125 766.4559 812.6153 873.8919
200 250 300 350 400 450 500 550 600 650 700 750 800 850 900
m/z

Z5

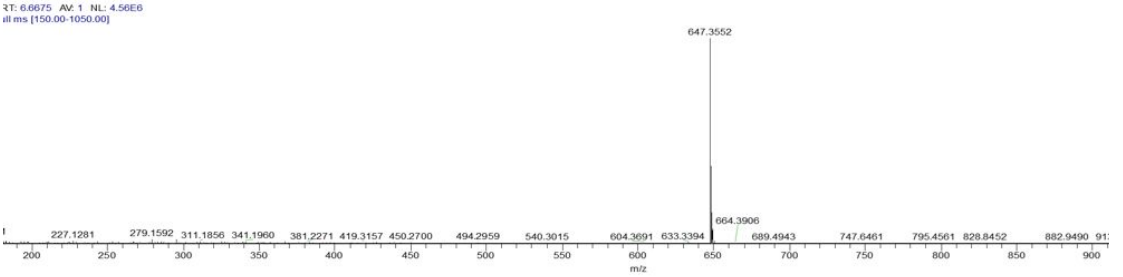
RT: 6.6675 AV: 1 NL: 4.56E6
ll ms [150.00-1050.00]
647.3552
664.3906
227.1281 279.1592 311.1856 341.1960 381.2271 419.3157 450.2700 494.2959 540.3015 604.3691 633.3394 689.4943 747.6461 795.4561 828.8452 882.9490
200 250 300 350 400 450 500 550 600 650 700 750 800 850 900
m/z

Z6

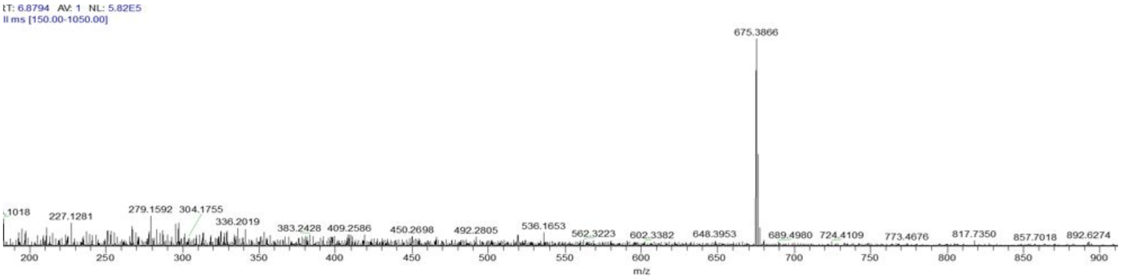
RT: 6.8794 AV: 1 NL: 5.82E5
ll ms [150.00-1050.00]
675.3866
227.1281 279.1592 304.1755 336.2019 383.2428 409.2586 450.2698 492.2805 536.1653 562.3223 602.3382 648.3953 689.4980 724.4109 773.4676 817.7350 857.7018 892.6274
200 250 300 350 400 450 500 550 600 650 700 750 800 850 900
m/z

Z7

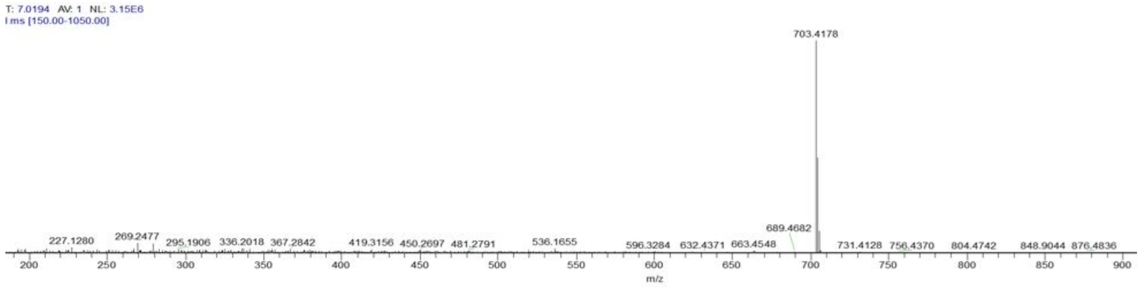
T: 7.0194 AV: 1 NL: 3.15E6
l ms [150.00-1050.00]
703.4178
689.4682
227.1280 269.2477 295.1906 336.2018 367.2842 419.3156 450.2697 481.2791 536.1655 596.3284 632.4371 663.4548 731.4128 756.4370 804.4742 848.9044 876.4836
m/z

Z8

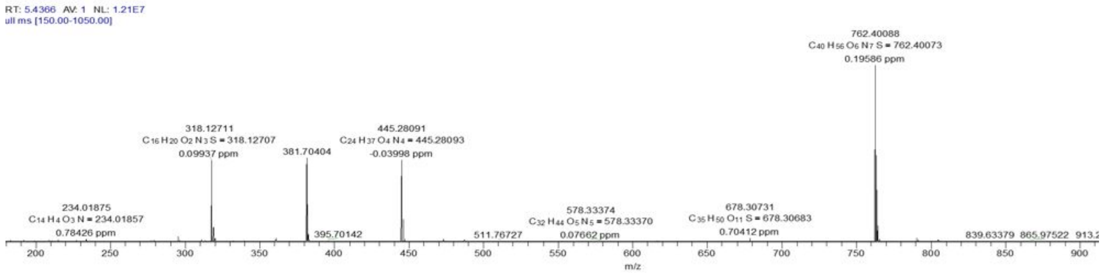
RT: 5.4366 AV: 1 NL: 1.21E7
ull ms [150.00-1050.00]
762.40088
C40 H56 O6 N7 S = 762.40073
0.19586 ppm
318.12711
C16 H20 O2 N3 S = 318.12707
0.09937 ppm
381.70404
445.28091
C24 H37 O4 N4 = 445.28093
-0.03998 ppm
234.01875
C14 H4 O3 N = 234.01857
0.78426 ppm
395.70142
511.76727
578.33374
C32 H44 O5 N5 = 578.33370
0.07662 ppm
678.30731
C35 H50 O11 S = 678.30683
0.70412 ppm
839.63379 865.97522
m/z

Z9

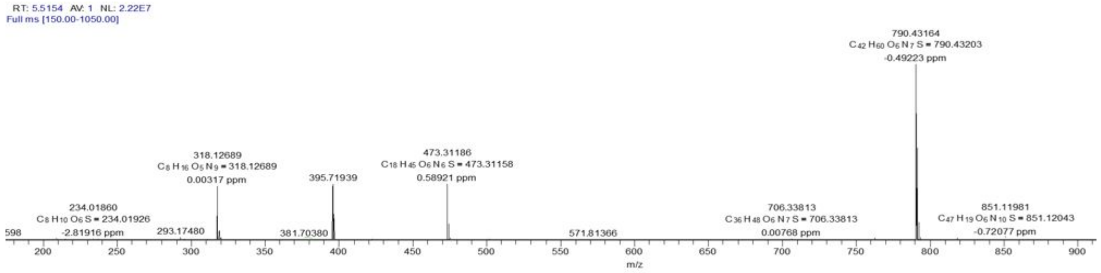
RT: 5.5154 AV: 1 NL: 2.22E7
Full ms [150.00-1050.00]
790.43164
C42 H60 O6 N7 S = 790.43203
-0.49223 ppm
318.12689
C8 H16 O5 N9 = 318.12689
0.00317 ppm
395.71939
473.31186
C18 H45 O6 N6 S = 473.31158
0.58921 ppm
234.01860
C8 H10 O6 S = 234.01926
-2.81916 ppm
293.17480
381.70380
571.81366
706.33813
C36 H48 O6 N7 S = 706.33813
0.00768 ppm
851.11981
C47 H19 O6 N10 S = 851.12043
-0.72077 ppm
m/z

Z10

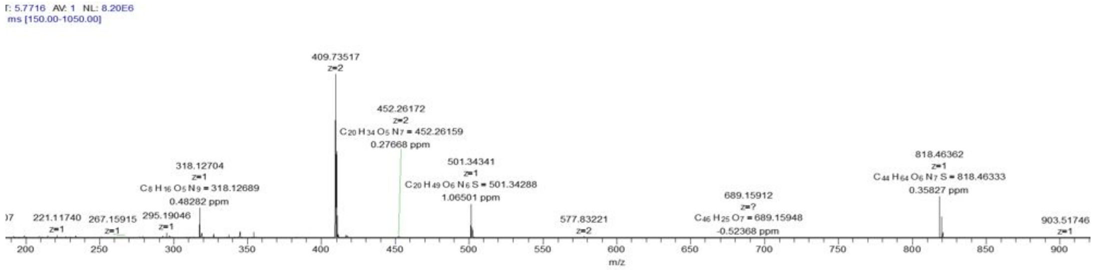
T: 5.7716 AV: 1 NL: 8.20E6
ms [150.00-1050.00]
409.73517
z=2
452.26172
z=2
C20 H34 O5 N7 = 452.26159
0.27668 ppm
501.34341
z=1
C20 H49 O6 N6 S = 501.34288
1.06501 ppm
318.12704
z=1
C8 H16 O5 N9 = 318.12689
0.48282 ppm
221.11740 z=1
267.15915 z=1
295.19046 z=1
577.83221 z=2
689.15912
z=?
C46 H25 O7 = 689.15948
-0.52368 ppm
818.46362
z=1
C44 H64 O6 N7 S = 818.46333
0.35827 ppm
903.51746 z=1
m/z

Z11

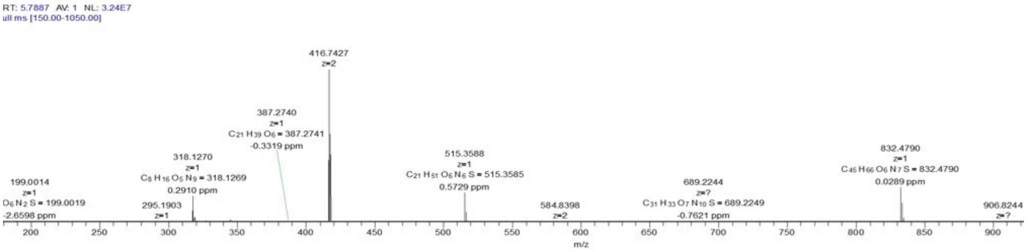

Z12

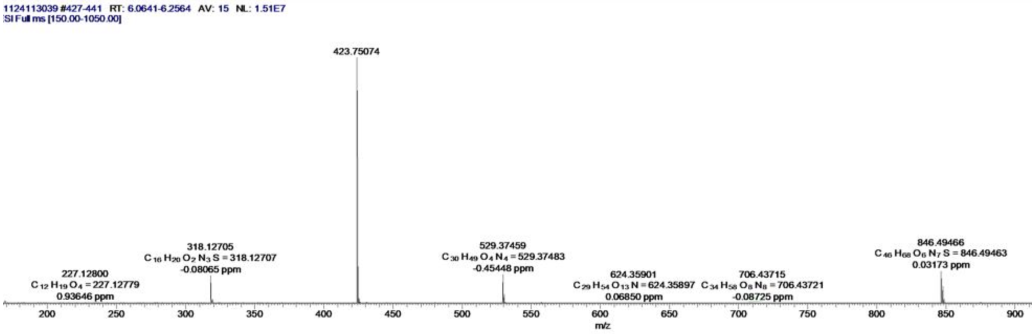

Z13

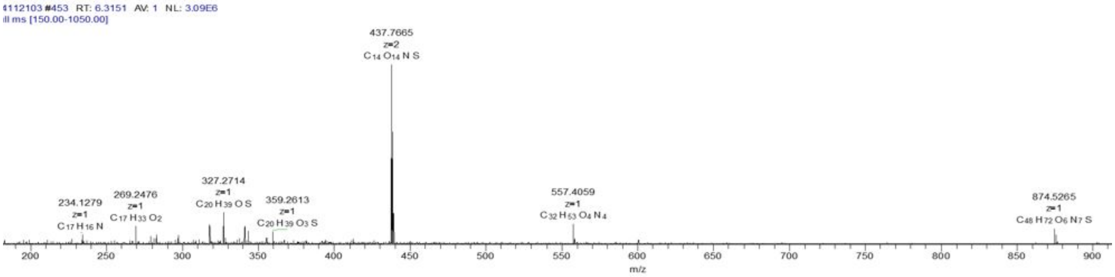

Z15

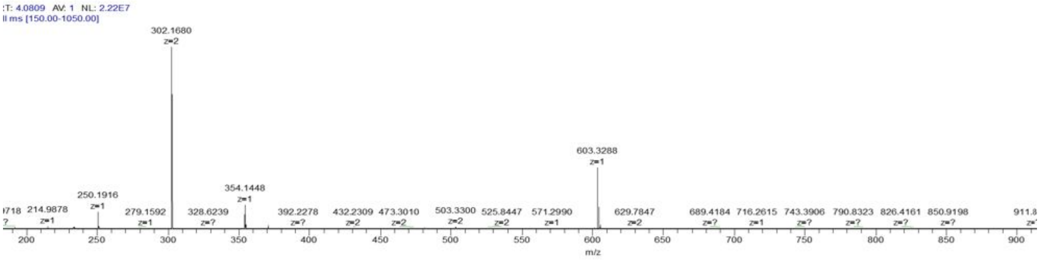

Z16

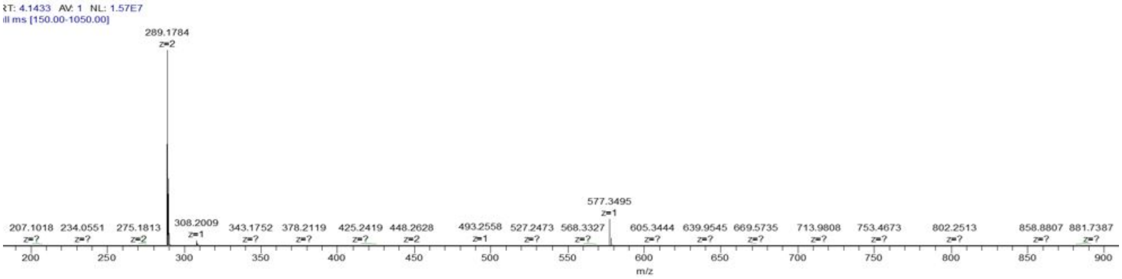

Z17

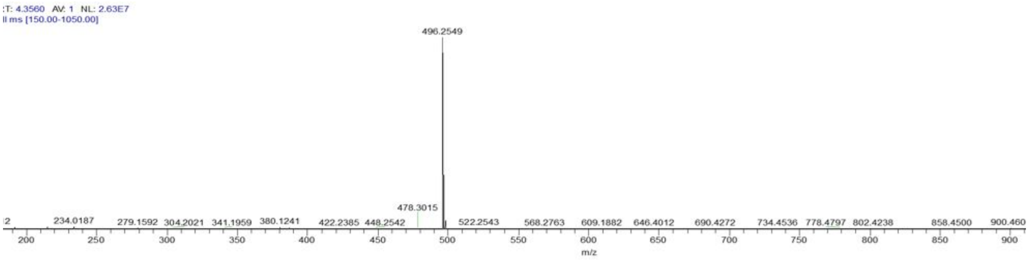

Z18

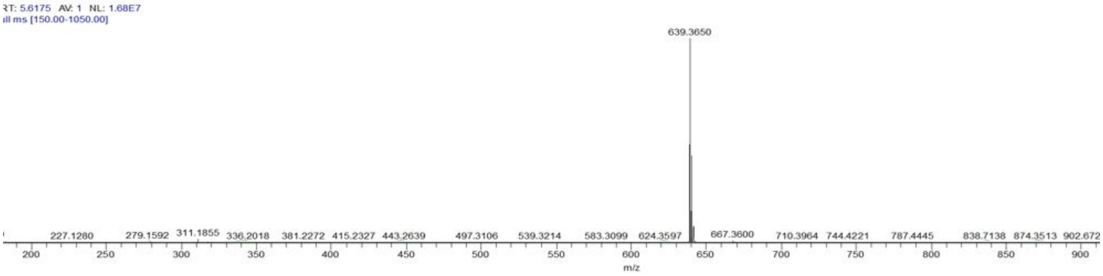

NC-Z16

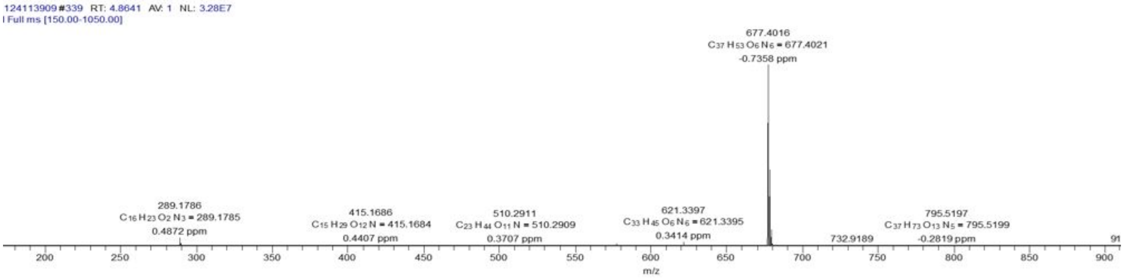

32

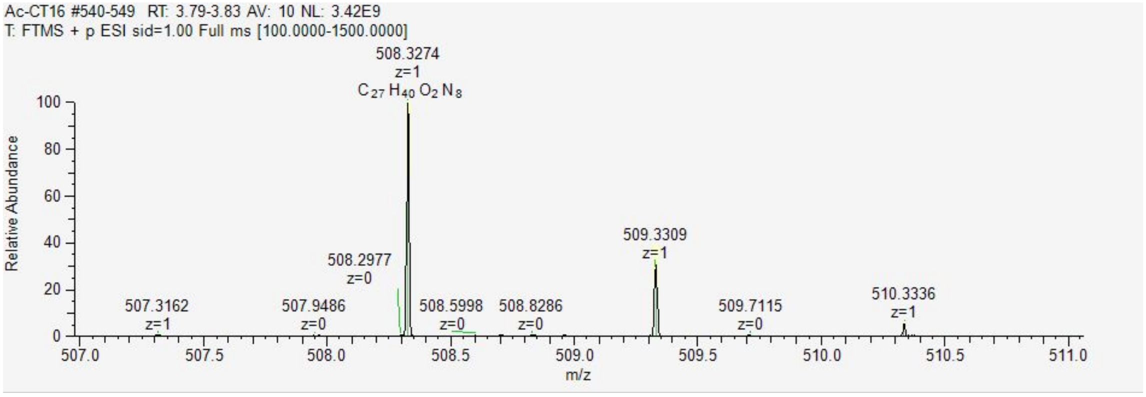

Representative HPLC traces

6

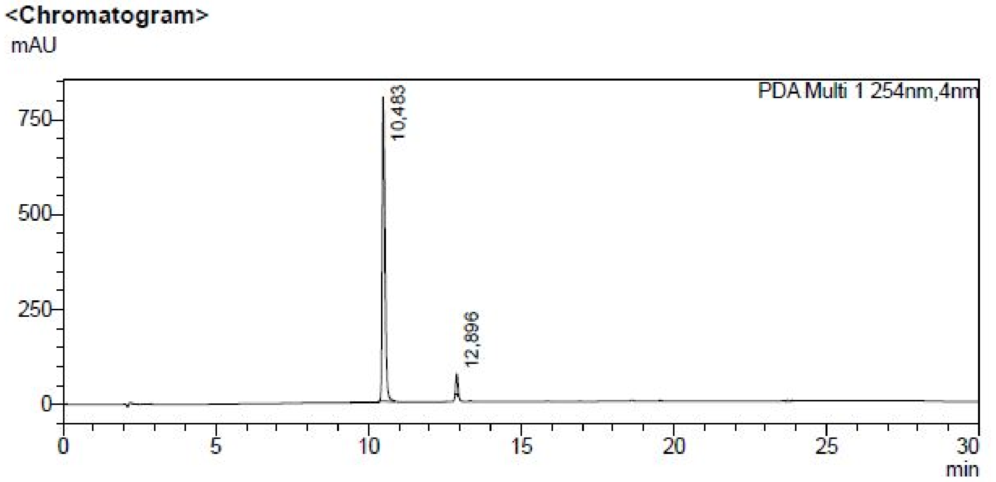

<Peak Table>

PDA Ch1 254nm

Peak#	Ret. Time	Area	Area%
1	10,483	5038295	95,896
2	12,896	215641	4,104
Total		5253937	100,000

Z4

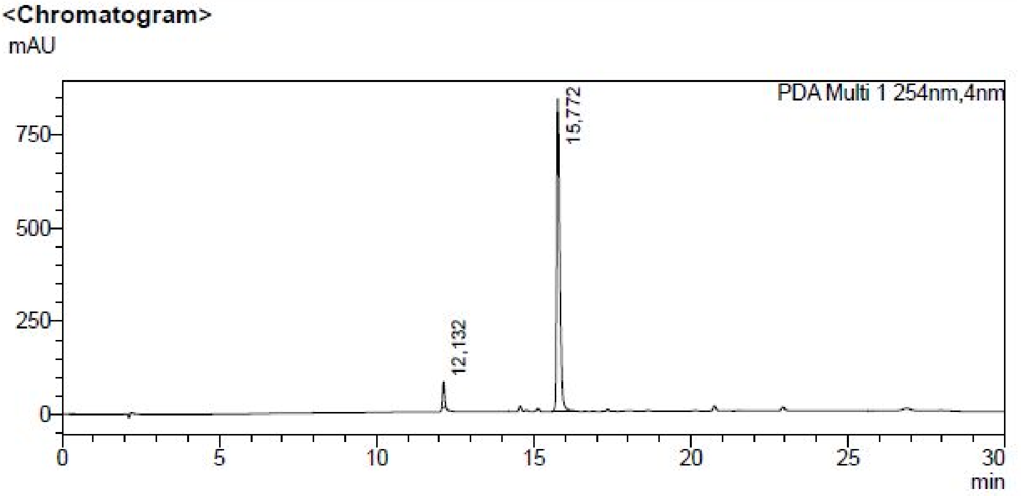

<Peak Table>

PDA Ch1 254nm

Peak#	Ret. Time	Area	Area%
1	12,132	277776	4,579
2	15,772	5788905	95,421
Total		6066682	100,000

Z5

<Chromatogram>

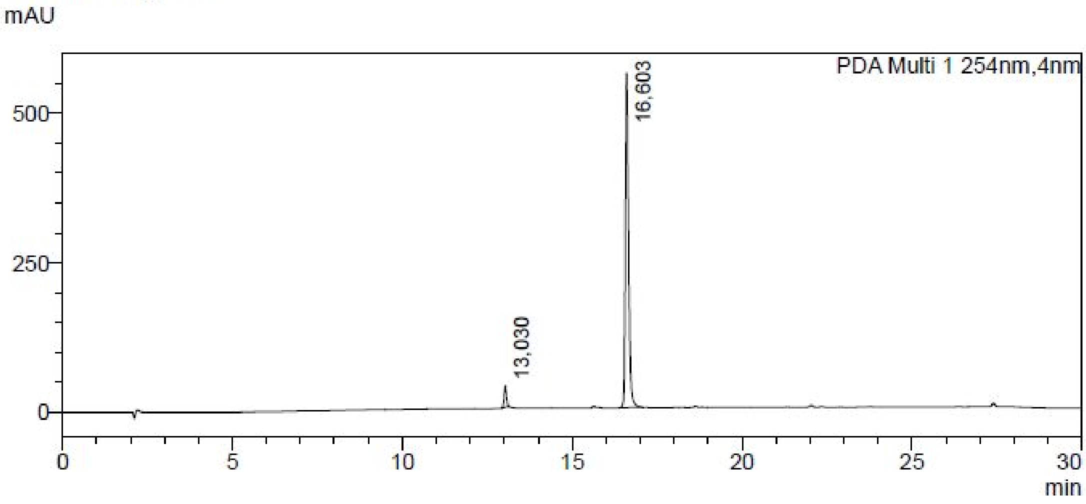

<Peak Table>

PDA Ch1 254nm

Peak#	Ret. Time	Area	Area%
1	13,030	172069	4,446
2	16,603	3698225	95,554
Total		3870293	100,000

Z6

<Chromatogram>

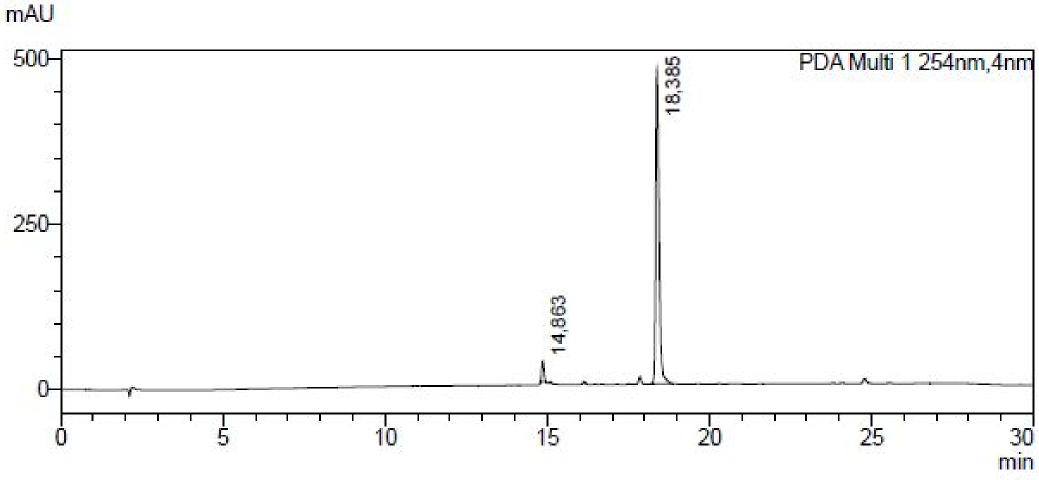

<Peak Table>

PDA Ch1 254nm

Peak#	Ret. Time	Area	Area%
1	14,863	147581	4,151
2	18,385	3407451	95,849
Total		3555032	100,000

Z10

<Chromatogram>

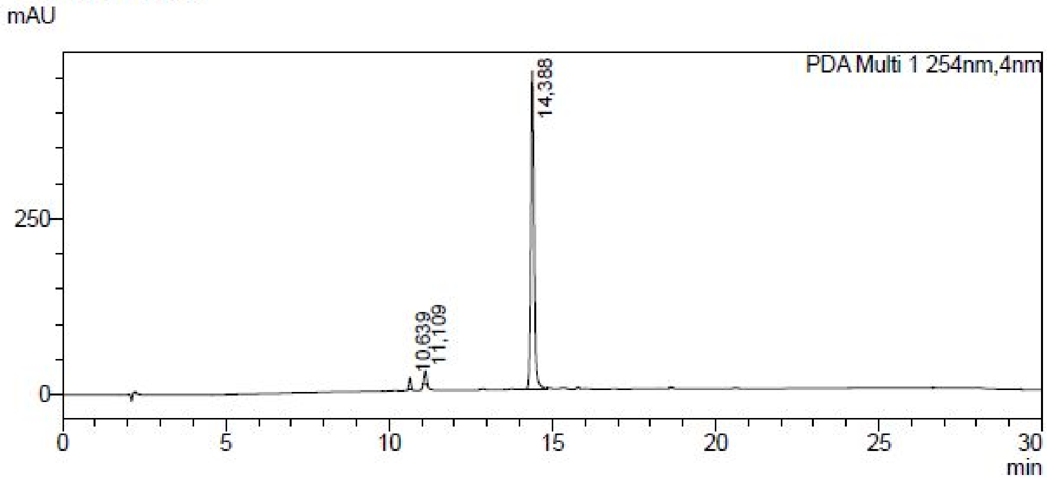

<Peak Table>

PDA Ch1 254nm

Peak#	Ret. Time	Area	Area%
1	10,639	67617	2,048
2	11,109	88521	2,681
3	14,388	3145345	95,271
Total		3301483	100,000

Z11

<Chromatogram>

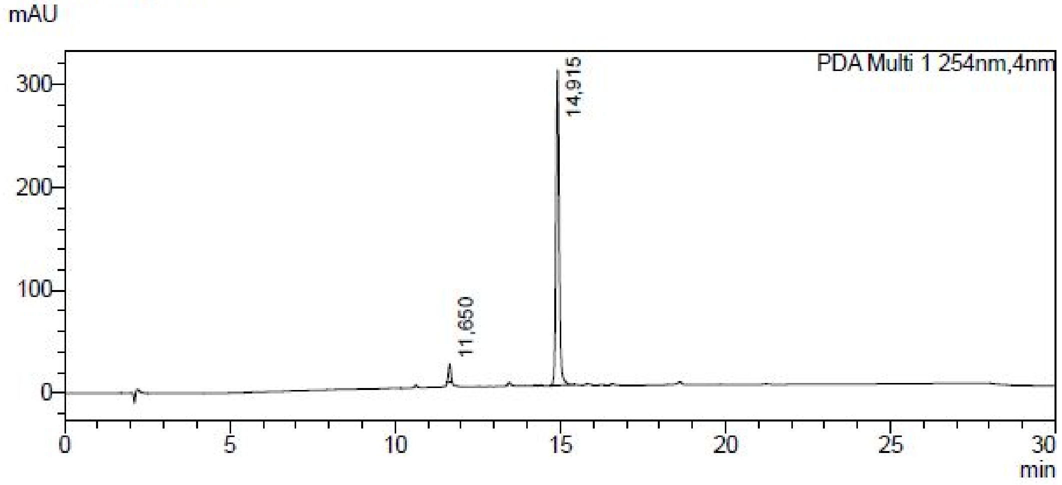

<Peak Table>

PDA Ch1 254nm

Peak#	Ret. Time	Area	Area%
1	11,650	86474	4,045
2	14,915	2051487	95,955
Total		2137961	100,000

Z15

<Chromatogram>

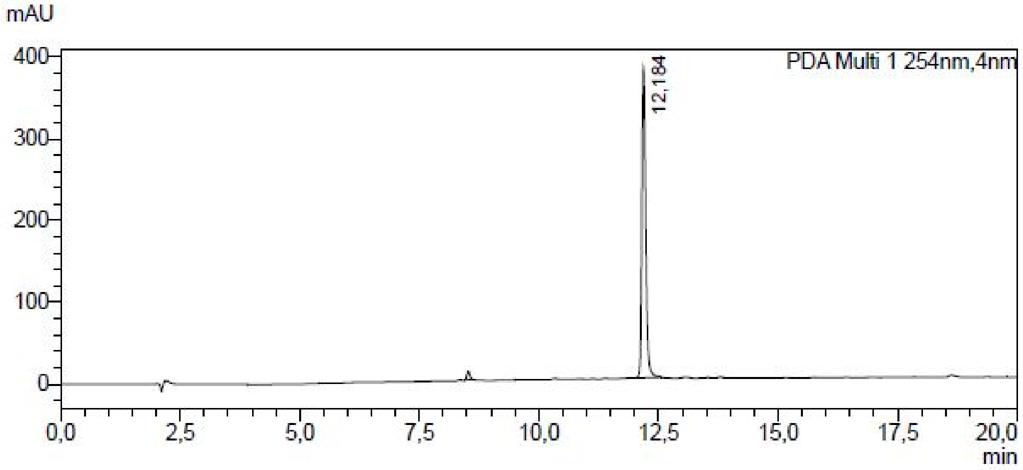

<Peak Table>

PDA Ch1 254nm

Peak#	Ret. Time	Area	Area%
1	8,523	44915	2,078
2	12,184	2116778	97,922
Total		2161693	100,000

Z16

<Chromatogram>

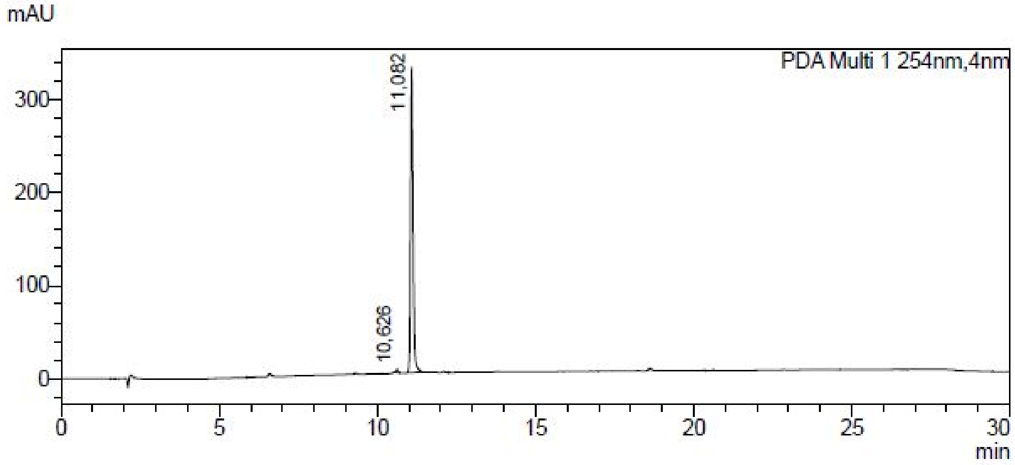

<Peak Table>

PDA Ch1 254nm

Peak#	Ret. Time	Area	Area%
1	10,626	15100	0,819
2	11,082	1828547	99,181
Total		1843647	100,000

Z18

<Chromatogram>

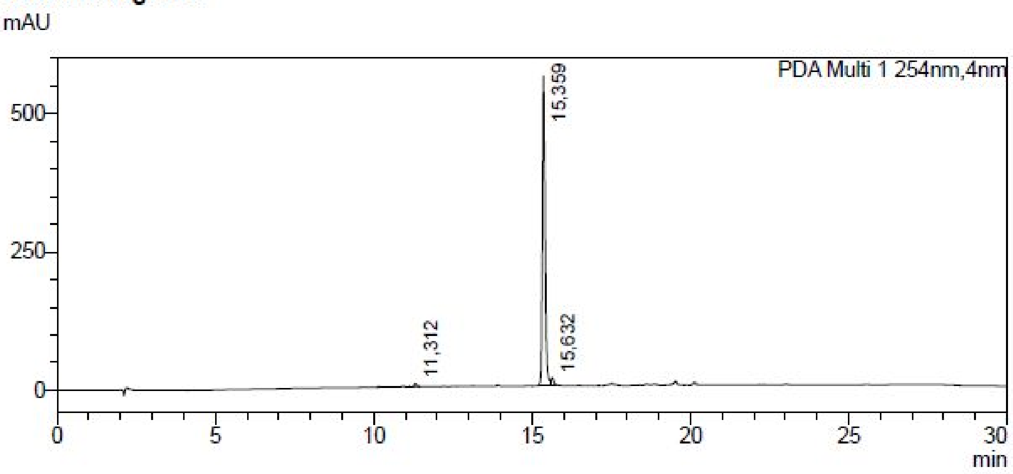

<Peak Table>

PDA Ch1 254nm

Peak#	Ret. Time	Area	Area%
1	11,312	29771	0,857
2	15,359	3360113	96,707
3	15,632	84650	2,436
Total		3474535	100,000

NC-Z16

<Chromatogram>

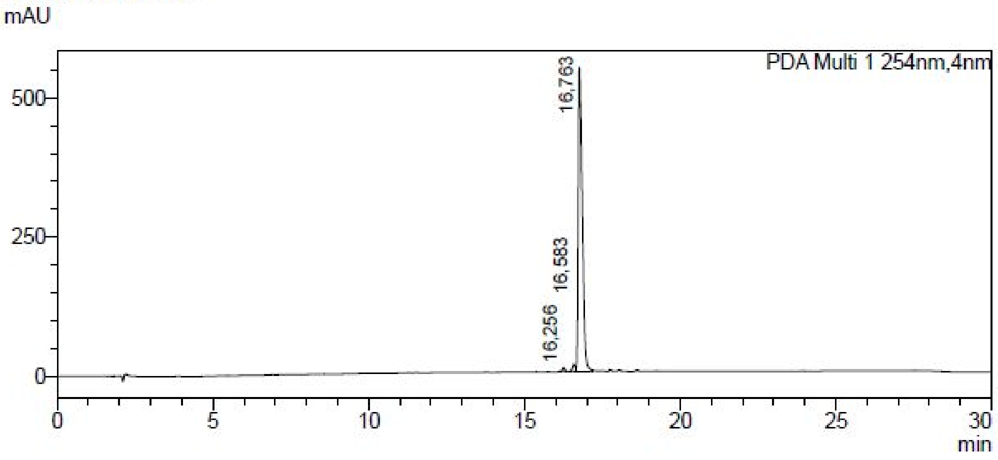

<Peak Table>

PDA Ch1 254nm

Peak#	Ret. Time	Area	Area%
1	16,256	30138	0,596
2	16,583	77687	1,535
3	16,763	4951739	97,869
Total		5059564	100,000

32

<Chromatogram>

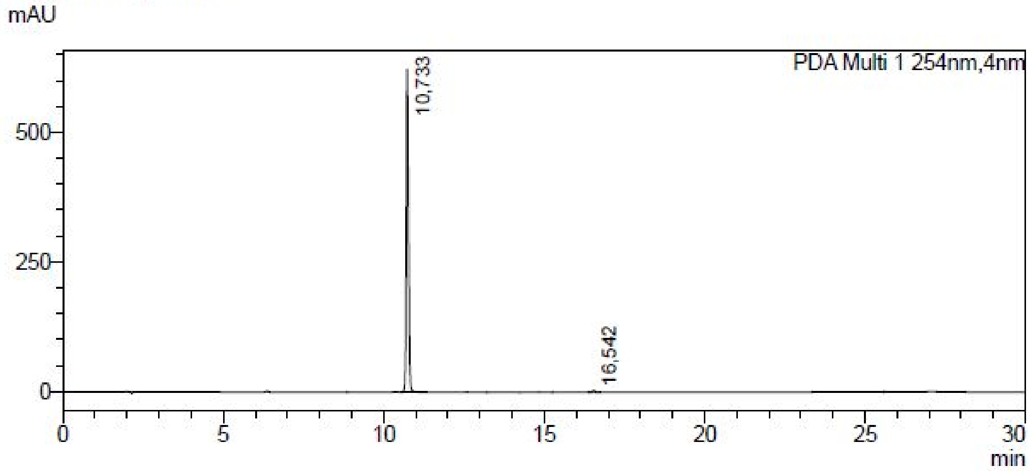

<Peak Table>

PDA Ch1 254nm

Peak#	Ret. Time	Area	Area%
1	10,733	2993199	99,368
2	16,542	19035	0,632
Total		3012234	100,000
